# Global burden, risk factor analysis, and prediction study of leukaemia from 1990 to 2030

**DOI:** 10.7189/jogh.14.04150

**Published:** 2024-08-23

**Authors:** Wenjun Wang, Donglei Zhang, Qian Liang, Xiaoyan Liu, Jun Shi, Fuling Zhou

**Affiliations:** 1 Department of Haematology, Zhongnan Hospital of Wuhan University, Wuhan, China; 2 Regenerative Medicine Clinic, State Key Laboratory of Experimental Haematology, National Clinical Research Centre for Blood Diseases, Haihe Laboratory of Cell Ecosystem, Institute of Haematology and Blood Diseases Hospital, Chinese Academy of Medical Sciences and Peking Union Medical College, Tianjin, China; 3 Zhoukou Central Hospital, Zhoukou, China

## Abstract

**Background:**

Leukaemia is a devastating disease with an incidence that progressively increases with advancing age. The World Health Organization has designated 2021–30 as the decade of healthy ageing, highlighting the need to address age-related diseases. We estimated the disease burden of leukaemia and forecasted it by 2030.

**Methods:**

Based on the Global Burden of Disease 2019 database, we systematically analysed the geographical distribution of leukaemia and its subtypes. We used Joinpoint regression and Bayesian age-period-cohort models to evaluate incidence and mortality trends from 1990 to 2019 and projections through 2030. We analysed five leukaemia subtypes and the impact of age, gender, and social development. Decomposition analysis revealed the effects of disease burden on ageing and population growth. We used frontier analysis to illustrate the potential of each country to reduce its burden based on its development levels.

**Results:**

Globally, the absolute numbers of leukaemia incidence and mortality have increased, while the age-standardised rates (ASRs) have shown a decreasing trend. The disease burden was more pronounced in men, the elderly, and regions with a high socio-demographic index (SDI), where ageing and population growth played varying roles across subtypes. From 2000 to 2006, disease burdens were most effectively controlled. Global ASRs of incidence might stabilise, while ASRs of death are expected to decrease until 2030. Frontier analysis showed that middle and high-middle SDI countries have the most improvement potential. Smoking and high body mass index were the main risk factors for leukaemia-related mortality and disability-adjusted life years.

**Conclusions:**

The absolute number of leukaemia cases has increased worldwide, but there has been a sharp decline in ASRs over the past decade, primarily driven by population growth and ageing. Countries with middle and high-middle SDI urgently need to take action to address this challenge.

Leukaemia is a malignancy of the haematological system, characterised by clonal malignant haematopoiesis of one or more cell lines. It can be categorised into acute myeloid leukaemia (AML), acute lymphoblastic leukaemia (ALL), chronic lymphocytic leukaemia (CLL), chronic myeloid leukaemia (CML), and other types of leukaemia in Global Burden of Disease (GBD) databases. The GBD 2015 study shows that leukaemia ranked eighth in terms of global cancer incidence and ninth in terms of cancer deaths [1]. The age-standardised rate (ASR) of disability-adjusted life years (DALYs) for leukaemia is around 35% of all tumours in children aged zero to 14 years and around 25–30% of all tumours in adolescents aged 15–19 years [2].

In recent years, with the improvement in diagnosis technologies and medical treatments, leukaemia has dropped from 5th in cancer-related DALYs in 2010 to 7th in 2019 [3,4]. The Surveillance, Epidemiology, and End Results database showed a significant increase in the five-year survival rate for leukaemia, from 10%, between 1950–54, to 63.8% between 2011–17, with different improvements observed for different subtypes. The approval of tyrosine kinase inhibitors in 2001 catapulted the 10-year survival rate for CML patients from <50% to >90% [5,6]. With the clinical application of rituximab in the late 1990s, the five-year survival rate for CLL patients increased from 67.5% in 1975 to 87.9% in 2007 [7,8]. The improved prognosis of ALL is due to advanced chemotherapy and breakthrough immunotherapies such as rituximab and Chimeric antigen receptor cell therapy, which have pushed the five-year survival rate to over 90% [9–13]. AML has a median survival of 8.5 months, but recent precision-targeted therapies suggest an improved outlook [4,14–18].

Although the survival of people with leukaemia has improved significantly over the past few decades, challenges remain, particularly for older patients and those in low- and middle-income countries who lack access to comprehensive treatment. With the advent of an ageing society, leukaemia is a crucial target of the third sustainable development goal, which aims to reduce the premature mortality rate from noncommunicable diseases by one-third before 2030 [19]. Therefore, a holistic view of specific epidemiological patterns and temporal trends is essential to optimising health care spending and formulating relevant policy frameworks. In this study, we systematically and comprehensively analysed the variations in disease burden and influencing factors of leukaemia and its subtypes from 1990 to 2019 and projected epidemiological trends up to 2030. We believe this study can potentially promote disease management and health decision-making improvements.

## METHODS

### Data sources

In this study, data were sourced from the GBD 2019 study. The Global Health Data Exchange tool extracted leukaemia-specific metrics, including incidence, prevalence, mortality, and DALYs. General methods for the GBD 2019 have been published previously [3,20]. The primary methods for estimating metrics by cause, age, sex, year, and location for the GBD 2019 study include the Cause of Death Ensemble model, Spatiotemporal Gaussian Process Regression, and the Bayesian meta-regression tool Disease Modelling Meta-Regression, version 2.1 (GBD Study, Institute of Health Metrics and Evaluation, University of Washington, Seattle, Washington, USA) [21]. This study follows the Guidelines for Accurate and Transparent Health Estimates Reporting (GATHER) statement.

### Data collection

Leukaemia and its subtypes are classified according to the diagnostic codes outlined in the International Classification of Diseases and Injuries, 10th edition. Specific codes are listed in Table S1 in the **Online Supplementary Document**.

### Risk factors

We used the GBD 2019 comparative risk assessment methodology in our study [22]. We also incorporated occupational exposure data from the International Labor Organization and used body mass index (BMI) benchmarks, including those set by the International Obesity Task Force [23,24].

### Statistical analysis

We obtained data sets from GBD to evaluate leukaemia and its subtypes’ epidemiological burden, including annual incidence, mortality, DALYs, and prevalence. To account for differences in age distributions between populations and periods, ASRs were calculated using the following formula:



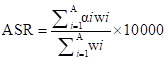



where α*_i_* is the age-specific rate for the *i* age groups, w*_i_* is the weight (or count) of individuals in the *i* age groups of a reference standard population, and A is the total number of age groups.

### Joinpoint regression analysis

Joinpoint regression analysis is particularly adept at discerning the impact of interventions or events on disease incidence or mortality rates. Joinpoint Regression Program, version 5.0.2 (Statistical Methodology and Applications Branch, National Cancer Institute, Bethesda, Maryland, USA) was used to identify trend changes and calculate annualised percentage change (APC) in leukaemia burden indicators. The average annual percentage change (AAPC), derived from APC, provided a comprehensive overview of leukaemia trends, aiding in understanding its temporal dynamics. We also analysed turning points in the year, marking the most pronounced trend deviations. We analysed multiple line segments on a logarithmic scale; the intersections are termed ‘joinpoints’. The maximum number of joinpoints is based on the number of data points in the series [25].

Pearson correlation coefficient was calculated for the AAPCs and the socio-demographic index (SDI), supplemented by Spearman’s rank-order correlation to assess the relationships between the ASRs and the SDI to elucidate associations between socioeconomic indicators and changing ASR trends.

### The age-period-cohort (APC) and Bayesian APC (BAPC) model

The unique aspect of the APC model lies in its utilisation of Bayesian methods and its comprehensive consideration of age, period, and cohort effects, providing a more holistic analytical framework for epidemiological research. The intrinsic estimator (IE) method of APC was used to overcome the inherent linear dependence between age, period, and cohort [26]. Our data stratification included categorising dimensions into five-year intervals. Mathematically, the APC model can be expressed as:



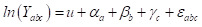



Here, *l_n_ (Y_abc_)* represents the natural logarithm of the disease incidence or mortality rate for a given group; *u* is the intercept, representing the reference level of disease risk in terms of age, period, and cohort parameters; *β_b_* and *γ_c_* are coefficients that capture the unique effects of age, period and cohort, respectively; and *ε_abc_* are represented by the error term or residual term of the APC model.

Using Bayesian methodology for estimating probability distributions, we combine prior knowledge with observed data to infer posterior distributions [27]. The BAPC model has relatively small absolute percentage biases, making it optimal for predicting leukaemia incidence and mortality before 2030 [28]. All computational analyses and projections were executed utilising the R, version 4.3.1 (R Core Team, Vienna, Austria) package ‘BAPC’ [29].

### Decomposition analysis

We used decomposition analysis to systematically separate and quantify the contributions of population ageing, population growth, and epidemiological transitions, elucidating the changing burden of leukaemia and its subtypes (AML, ALL, CLL, CML, and other leukaemia) from 1990 to 2019. Integrating the basic methodology with advanced refinements facilitated a nuanced dissection of the respective influences of these determinants on overarching changes in incidence, mortality, and DALYs [26,27].

### Frontier analysis

The frontier analysis explored the relationship between leukaemia mortality, DALYs, and SDI. We establish a nonlinear frontier, representing the lowest attainable mortality rates given a specific level of development [30]. We defined the effective difference as the gap between observed leukaemia age-standardised death rate (ASDR) and ASR-DALYs compared to this frontier, highlighting unrealised health gains relative to the region or country’s current level of development.

### Data visualisation

All analyses were performed using R, version 4.3.1, with a *P-*value threshold of <0.05 to establish statistical significance.

## RESULTS

### Global trends in the epidemiology of leukaemia and its subtypes

**Figure 1:**
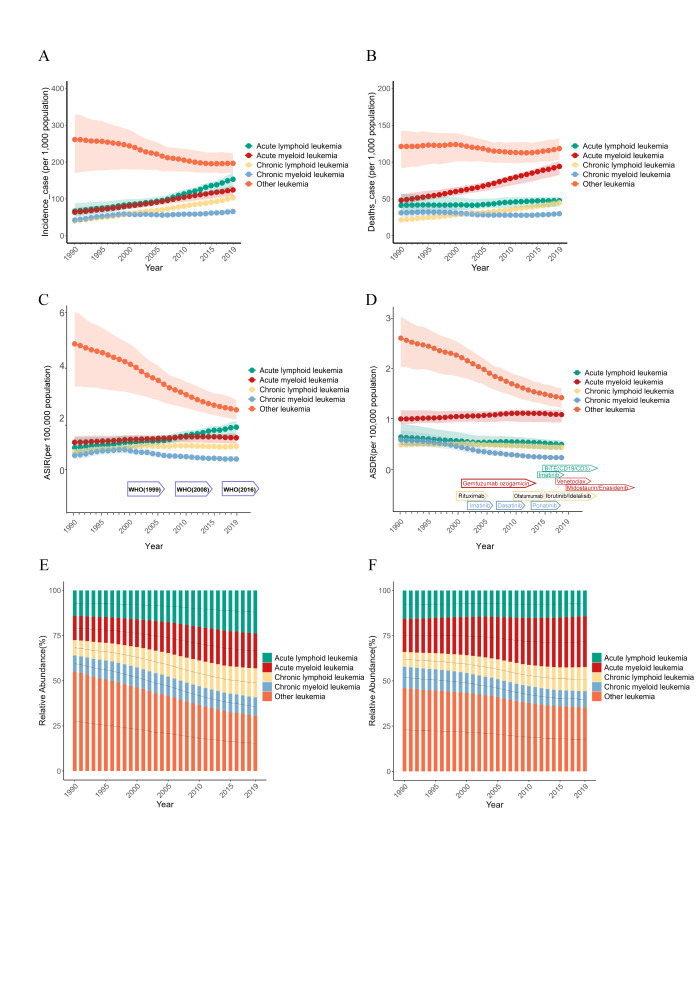
Global trends in incidence and death for leukaemia and its five subtypes from 1990 to 2019. **Panel A.** The new cases of leukaemia from 1990 to 2019. **Panel B.** The number of deaths due to leukaemia from 1990 to 2019. **Panel C.** ASIR and AAPC in leukaemia over the last 30 years. **Panel D.** ASDR and AAPC in leukaemia over the previous 30 years. **Panel E.** The proportion of new cases of leukaemia from 1990 to 2019. **Panel F.** The proportion of deaths of leukaemia from 1990 to 2019. AAPC – average annual percentage changes, ASDR – age-standardised death rate, ASIR – age-standardised incidence rate, WHO – World Health Organization.

Our analysis from 1990 to 2019 observed significant changes in leukaemia’s global incidence and mortality rates (**Table 1**, **Table 2**). In 2019, the global incidence of leukaemia was reported at 643 579 cases. For subtypes, ALL accounted for 153 315, AML for 124 333, CLL for 103 467, CML for 65 800, and other leukaemia for 196 665 cases (**Figure 1**, Panel A). Leukaemia-related deaths in 2019 were reported at 334 592 cases, with ALL at 47 645, AML at 94 056, CLL at 44 613, CML at 29 931, and other leukaemia at 118 348 (**Figure 1**, Panel B).

**Table 1:** The global and GBD regions incident cases and their AAPCs of leukaemia from 1990 to 2019

	1990		2019		
**Location**	**Incident cases, n ×10^3^ (95% CI)**	**ASIR per 100 000 population, n (95% CI)**	**Incident cases, n ×10^3^ (95% CI)**	**ASIR per 100 000 population, n (95% CI)**	**AAPC (95% CI)**
Global	474.92 (388.56, 560.55)	9.60 (8.14, 11.02)	643.58 (586.98, 699.73)	8.22 (7.50, 8.94)	–0.54 (–0.62, –0.45)
SDI					
*High*	104.37 (100.13, 106.82)	11.18 (10.74, 11.45)	188.54 (169.16, 208.21)	11.99 (10.87, 13.21)	0.24 (0.05, 0.43)
*High-middle*	114.95 (99.42, 127.24)	10.54 (9.12, 11.69)	167.72 (150.62, 183.33)	10.11 (9.05, 11.14)	–0.15 (–0.29, –0.01)
*Middle*	149.08 (113.02, 179.04)	9.01 (7.16, 10.62)	163.72 (144.03, 183.74)	6.90 (6.08, 7.74)	–0.91(–1.01, –0.81)
*Low-middle*	69.81 (46.71, 97.16)	6.28 (4.65, 8.11)	74.17 (64.99, 86.01)	4.72 (4.16, 5.45)	–0.98 (–1.06, –0.9)
*Low*	36.47 (20.05, 60.21)	6.43 (4.4, 9.37)	49.05 (37.94, 60.80)	5.10 (4.09, 6.02)	–0.81 (–0.91, –0.71)
Region					
*Andean Latin America*	2.72 (2.22, 3.53)	7.44 (6.28, 9.37)	4.42 (3.23, 5.62)	7.30 (5.33, 9.25)	–0.09 (–0.46, 0.29)
*Australasia*	2.18 (2.07, 2.28)	9.77 (9.28, 10.21)	4.72 (3.81, 5.80)	10.46 (8.44, 12.78)	0.34 (0.01, 0.67)
*Caribbean*	2.77 (2.10, 3.83)	8.22 (6.52, 10.88)	3.61 (2.84, 4.45)	7.56 (5.83, 9.49)	–0.27 (–0.41, –0.14)
*Central Asia*	6.37 (5.72, 6.84)	8.87 (8.11, 9.41)	5.25 (4.61, 6.05)	6.05 (5.35, 6.93)	–1.29 (–1.43, –1.15)
*Central Europe*	10.48 (10.18, 10.87)	7.93 (7.67, 8.23)	17.32 (15.24, 19.60)	9.59 (8.45, 10.87)	0.64 (0.55, 0.73)
*Central Latin America*	11.10 (10.54, 11.66)	7.03 (6.77, 7.30)	15.98 (13.60, 18.62)	6.58 (5.60, 7.67)	–0.19 (–0.23, –0.15)
*Central sub-Saharan Africa*	3.30 (1.49, 6.03)	5.45 (3.57, 8.22)	4.09 (3.06, 5.53)	3.89 (2.91, 4.98)	–1.17 (–1.33, –1.01)
*East Asia*	147.56 (106.05, 178.26)	12.68 (9.23, 15.23)	159.36 (131.90, 185.88)	10.41 (8.69-12.26)	–0.64 (–0.96, –0.31)
*Eastern Europe*	19.67 (18.89, 20.41)	8.18 (7.83, 8.53)	21.62 (19.53, 23.85)	7.54 (6.86, 8.29)	–0.27 (–1.06, 0.53)
*Eastern sub-Saharan Africa*	19.12 (10.21, 33.79)	8.26 (5.3, 13.2)	21.55 (14.00, 29.46)	5.85 (4.15, 7.71)	–1.20 (–1.3, –1.11)
*High-income Asia Pacific*	15.85 (14.85, 16.73)	9.03 (8.38, 9.61)	29.20 (24.93, 33.27)	10.33 (8.98, 11.7)	0.47 (0.24, 0.7)
*High-income North America*	39.06 (37.17, 40.10)	11.82 (11.26-12.14)	61.55 (53.74, 70.24)	10.69 (9.38, 12.13)	–0.39 (–0.58, –0.19)
*North Africa and the Middle East*	29.19 (20.05, 39.89)	9.51 (7.1, 11.96)	39.30 (32.62, 45.06)	7.76 (6.54, 8.84)	–0.70 (–0.81, –0.58)
*Oceania*	0.41 (0.27, 0.61)	7.11 (5.19, 9.77)	0.81 (0.53, 1.22)	6.84 (4.8-9.75)	–0.14 (–0.35, 0.07)
*South Asia*	46.31 (30.34, 64.72)	4.75 (3.57, 6.1)	59.86 (51.91, 70.10)	3.81 (3.32, 4.45)	–0.75 (–0.99, –0.52)
*Southeast Asia*	37.67 (25.23, 53.04)	8.64 (6.3, 11.33)	42.26 (35.84, 49.69)	6.81 (5.8, 7.99)	–0.80 (–0.84, –0.76)
*Southern Latin America*	3.61 (3.48, 3.74)	7.52 (7.24, 7.78)	5.39 (4.27, 6.70)	7.20 (5.71, 8.93)	–0.15 (–0.37, 0.08)
*Southern sub-Saharan Africa*	1.88 (1.61, 2.14)	4.62 (4.02, 5.11)	2.76 (2.38, 3.14)	4.34 (3.71, 4.86)	–0.09 (–0.24, 0.06)
*Tropical Latin America*	9.35 (8.71, 9.99)	6.86 (6.48, 7.23)	12.37 (11.56, 13.08)	5.53 (5.14, 5.88)	–0.74 (–0.8, –0.67)
*Western Europe*	60.01 (57.77, 61.72)	12.71 (12.25, 13.14)	118.62 (102.90, 135.44)	16.87 (14.68, 19.38)	0.90 (0.74, 1.06)
*Western sub-Saharan Africa*	6.33 (4.29, 9.21)	3.82 (2.97, 4.85)	13.56 (10.27, 17.28)	3.90 (3.14, 4.68)	0.07 (–0.03, 0.17)

AAPC – average annual percent change, ASI – age-standardised incidence rate, CI – confidence interval, GBD – global burden of disease, SDI – socio-demographic index

**Table 2:** The global and GBD region’s deaths cases and their AAPCs of leukaemia from 1990 to 2019

	1990		2019		
**Location**	**Death cases, n ×10^3^ (95% CI)**	**ASDR per 100 000 population, n (95% CI)**	**Death cases, n ×10^3^ (95% CI)**	**ASDR per 100 000 population, n (95% CI)**	**AAPC (95% CI)**
Global	263.26 (233.66, 298.70)	5.82 (5.25, 6.44)	334.59 (306.82, 360.21)	4.26 (3.91, 4.58)	–1.07 (–1.13, –1.01)
SDI					
*High*	58.95 (56.28, 60.25)	5.96 (5.70, 6.09)	85.26 (76.99, 89.68)	4.61 (4.27, 4.82)	–0.9 (–0.96, –0.84)
*High-middle*	65.41 (59.75, 69.78)	6.06 (5.55, 6.46)	75.83 (68.74, 81.31)	4.21 (3.80, 4.53)	–1.24 (–1.34, –1.13)
*Middle SDI*	77.56 (66.82, 87.91)	5.46 (4.79, 6.11)	92.24 (81.50, 103.86)	3.9 (3.45, 4.38)	–1.14 (–1.2, –1.09)
*Low-middle*	40.24 (30.71, 51.62)	4.42 (3.61, 5.34)	51.12 (45.29, 59.13)	3.45 (3.07, 3.98)	–0.84 (–0.94, –0.75)
*Low*	20.96 (13.45, 32.73)	4.78 (3.67, 6.51)	29.92 (24.14, 35.50)	3.88 (3.12, 4.56)	–0.74(–0.81, –0.66)
Region					
*Andean Latin America*	1.65 (1.44, 2.06)	5.32 (4.69, 6.50)	3.05 (2.26, 3.81)	5.15 (3.83, 6.42)	–0.17 (–0.52, 0.18)
*Australasia*	1.37 (1.31, 1.41)	6.04 (5.76, 6.25)	2.37 (2.12, 2.57)	4.83 (4.39, 5.20)	–0.73 (–0.89, –0.57)
*Caribbean*	1.71 (1.43, 2.12)	5.51 (4.77, 6.58)	2.36 (1.95, 2.81)	4.82 (3.95, 5.81)	–0.43 (–0.57, –0.28)
*Central Asia*	3.31 (3.18, 3.44)	5.21 (5.02, 5.38)	3.19 (2.85, 3.60)	3.89 (3.50, 4.37)	–0.97 (–1.16, –0.78)
*Central Europe*	7.63 (7.41, 7.92)	5.58 (5.42, 5.81)	9.59 (8.46, 10.79)	4.85 (4.29, 5.45)	–0.54 (–0.69, –0.39)
*Central Latin America*	6.51 (6.28, 6.76)	4.85 (4.68, 5.02)	10.72 (9.28, 12.35)	4.45 (3.85, 5.13)	–0.26 (–0.3, –0.21)
*Central sub-Saharan Africa*	1.64 (0.99, 2.58)	3.79 (3.05, 4.76)	2.42 (1.84, 3.06)	2.99 (2.18, 4.04)	–0.83 (–0.94, –0.72)
*East Asia*	68.81 (56.22, 79.72)	6.25 (5.17, 7.20)	62.98 (52.70, 73.47)	3.69 (3.12, 4.28)	–1.8 (–1.96, –1.64)
*Eastern Europe*	13.57 (13.20, 13.90)	5.43 (5.27, 5.57)	11.75 (10.64, 12.90)	3.9 (3.55-4.28)	–0.96 (–1.58, –0.33)
*Eastern sub-Saharan Africa*	10.24 (6.15, 17.77)	5.87 (4.19, 8.84)	12.06 (8.40, 15.92)	4.37 (3.19, 5.61)	–1 (–1.1, –0.91)
*High-income Asia Pacific*	8.59 (8.25, 8.83)	4.61 (4.40, 4.75)	12.12 (10.46, 13.03)	3.02 (2.72, 3.22)	–1.47 (–1.56, –1.38)
*High-income North America*	23.44 (22.17, 24.09)	6.8 (6.46, 6.98)	34.71 (32.03, 36.51)	5.65 (5.28, 5.92)	–0.65 (–0.83, –0.47)
*North Africa and the Middle East*	16.92 (13.27, 21.05)	6.99 (5.58, 8.19)	25.14 (21.11, 28.83)	5.41 (4.62, 6.13)	–0.88 (–0.95, –0.81)
*Oceania*	0.23 (0.18, 0.32)	5.2 (4.08, 6.71)	0.46 (0.33, 0.64)	4.79 (3.58, 6.48)	–0.31 (–0.49, –0.14)
*South Asia*	31.17 (23.27, 40.74)	3.81 (3.10, 4.62)	44.55 (38.75, 52.63)	2.98 (2.59, 3.50)	–0.84 (–1.12, –0.56)
*Southeast Asia*	20.85 (16.12, 26.13)	5.77 (4.72, 6.91)	28.50 (24.41, 32.96)	4.74 (4.05, 5.47)	–0.67 (–0.74, –0.59)
*Southern Latin America*	2.72 (2.64, 2.80)	5.82 (5.61, 6.00)	3.84 (3.59, 4.06)	4.9 (4.60, 5.18)	–0.59 (–0.71, –0.48)
*Southern sub-Saharan Africa*	1.23 (1.09, 1.36)	3.57 (3.08, 4.01)	1.96 (1.65, 2.17)	3.35 (2.78, 3.73)	–0.22 (–0.56, 0.11)
*Tropical Latin America*	5.80 (5.55, 6.07)	4.85 (4.66, 5.04)	9.07 (8.45, 9.50)	3.97 (3.69, 4.19)	–0.65 (–0.73, –0.56)
*Western Europe*	31.67 (30.30, 32.41)	5.95 (5.70, 6.08)	45.00 (39.92, 47.62)	4.91 (4.46, 5.15)	–0.67 (–0.77, –0.56)
*Western sub-Saharan Africa*	4.19 (3.11, 5.59)	3.13 (2.54, 3.73)	8.78 (6.93, 10.83)	3.12 (2.54, 3.72)	–0.01 (–0.1, 0.08)

AAPC – average annual percent change, ASDR – age-standardised death rate, CI – confidence interval, GBD – global burden of disease, SDI – socio-demographic index

In 2019, the subtype with the highest age-standardised incidence rate (ASIR) and ASDR was other leukaemia, followed by ALL and AML (**Figure 1**, Panels C and D). The ASIR for ALL, AML, and CLL were increased (AAPC = 1.62, 0.37, and 0.56). Conversely, ASIR of CML and other leukaemia were decreased (AAPC = –0.5 and –2.21). The ASDR for leukaemia and most subtypes decreased, while that for AML exhibited an upward trend (AAPC = 0.25).

In 2019, other leukaemia, while still constituting the majority, declined to represent about 30% of new cases, down from its proportion in 1990. Since 1995, other leukaemia accounted for less than half of all new diagnoses, with increasing proportions of AML, ALL, and CLL (**Figure 1**, Panel E). Regarding mortality, other leukaemia remains predominant, but their share is decreasing (**Figure 1**, Panel F). In 2019, leukaemia prevalence cases decreased (Figure S1, Panel A and Table S2 in the **Online Supplementary Document**). An increasing trend in the ASR of prevalence was observed for ALL, AML, CML, and CLL. In contrast, the prevalence of other leukaemia decreased significantly. Meanwhile, DALYs for leukaemia decreased. The ASR-DALYs for most subtypes demonstrated a decreasing trend, except AML (Figure S1, Panels B–D in the **Online Supplementary Document**).

### Epidemiological characteristics of leukaemia and its subtypes by gender and age

**Figure 2:**
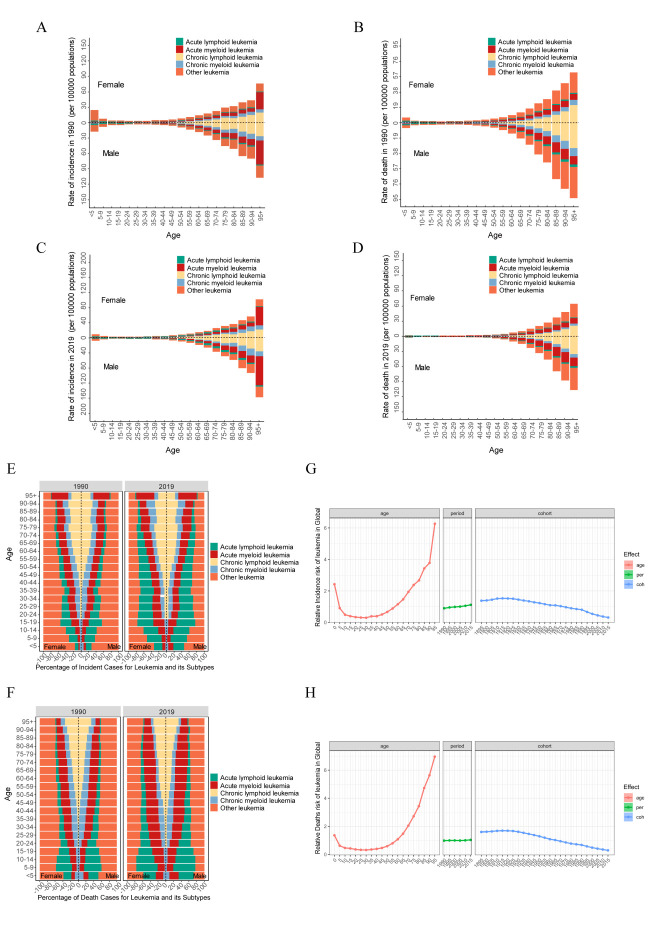
Analysis of global leukaemia incidence and mortality by age, sex, and APC-IE model. **Panel A.** Global leukaemia incidence rate by age for both sexes in 1990. **Panel B.** Global leukaemia death rates by age for both sexes in 1990. **Panel C.** Global leukaemia incidence rates by age for both sexes in 2019. **Panel D.** Global leukaemia death rates by age for both sexes in 2019. **Panel E.** The proportion of incidence of leukaemia by sex and age in 1990 and 2019. **Panel F.** The proportion of leukaemia deaths by sex and age in 1990 and 2019. **Panel G.** Age, period, and cohort effect coefficients of the APC-IE model of global leukaemia incidence risk from 1990 to 2019. **Panel H.** Age, period, and cohort effect coefficients of the APC-IE model of global leukaemia mortality risk from 1990 to 2019. APC-IE – age-period-cohort model with intrinsic estimator.

From a gender perspective, males consistently exhibit higher incidence and mortality rates related to leukaemia (**Figure 2**, Panels A and D, **Table 3**). This phenomenon stands out markedly in those aged ≥50 years. In 1990, it was evident that male leukaemia incidence and mortality rates surpassed those of females (**Figure 2**, Panels A and B), and this gap widened by 2019 (**Figure 2**, Panels C and D). Several factors, such as genetic predisposition, lifestyle choices, or hormonal fluctuations, could contribute to this disparity. Specifically, our analysis revealed male dominance in all age groups except those younger than five years (**Table 3**). In 1990, CML had the highest male-to-female incidence and mortality ratios for leukaemia subtypes, followed by CLL, AML, other leukaemia, and ALL (Table S3 in the **Online Supplementary Document**). Notably, females had higher other leukaemia incidence rates than males in both years, with ratios between 1.6:1.0 and 1.3:1.0.

**Table 3:** Incidence and mortality relative sex ratio of leukaemia in global

	Incidence		Mortality	
**Age in years**	**1990, RR (95% CI)**	**2019, RR (95% CI)**	**1990, RR (95% CI)**	**2019, RR (95% CI)**
<5	0.73 (0.79, 0.63)	0.93 (0.94, 0.93)	0.96 (1.01, 0.82)	1.30 (1.36, 1.17)
5–9	1.07 (1.12, 0.98)	1.22 (1.25, 1.15)	1.25 (1.26, 1.08)	1.37 (1.41, 1.30)
10–14	1.12 (1.13, 1.00)	1.16 (1.24, 1.10)	1.24 (1.26, 1.07)	1.28 (1.36, 1.22)
15–19	1.29 (1.29, 1.20)	1.31 (1.36, 1.24)	1.35 (1.34, 1.26)	1.39 (1.46, 1.32)
20–24	1.26 (1.25, 1.19)	1.30 (1.30, 1.23)	1.34 (1.30, 1.27)	1.40 (1.43, 1.32)
25–29	1.05 (1.01, 1.09)	1.23 (1.21, 1.19)	1.11 (1.06, 1.16)	1.35 (1.36, 1.27)
30–34	1.06 (1.03, 1.05)	1.24 (1.23, 1.21)	1.11 (1.08, 1.11)	1.37 (1.40, 1.26)
35–39	1.01 (0.99, 1.01)	1.15 (1.13, 1.11)	1.07 (1.04, 1.08)	1.28 (1.32, 1.20)
40–44	1.07 (1.08, 1.01)	1.15 (1.15, 1.13)	1.13 (1.12, 1.06)	1.23 (1.27, 1.17)
45–49	1.10 (1.11, 1.05)	1.16 (1.19, 1.12)	1.12 (1.13, 1.08)	1.21 (1.28, 1.14)
50–54	1.19 (1.20, 1.16)	1.18 (1.19, 1.15)	1.19 (1.18, 1.14)	1.23 (1.29, 1.18)
55–59	1.25 (1.25, 1.20)	1.23 (1.23, 1.20)	1.27 (1.27, 1.20)	1.30 (1.34, 1.24)
60–64	1.34 (1.37, 1.30)	1.32 (1.35, 1.28)	1.38 (1.41, 1.34)	1.40 (1.47, 1.33)
65–69	1.45 (1.47, 1.41)	1.43 (1.44, 1.41)	1.51 (1.55, 1.47)	1.52 (1.56, 1.47)
70–74	1.51 (1.54, 1.46)	1.49 (1.52, 1.48)	1.57 (1.61, 1.52)	1.57 (1.61, 1.49)
75–79	1.58 (1.59, 1.58)	1.59 (1.60, 1.61)	1.64 (1.68, 1.64)	1.65 (1.67, 1.65)
80–84	1.61 (1.60, 1.68)	1.64 (1.63, 1.75)	1.69 (1.68, 1.74)	1.72 (1.72, 1.81)
85–89	1.65 (1.64, 1.76)	1.74 (1.70, 1.88)	1.75 (1.74, 1.85)	1.87 (1.84, 2.00)
90–94	1.61 (1.58, 1.72)	1.68 (1.61, 1.86)	1.68 (1.66, 1.79)	1.82 (1.76, 1.98)
≥95	1.41 (1.41, 1.48)	1.56 (1.5, 1.63)	1.50 (1.47, 1.60)	1.65 (1.65, 1.73)
Total	1.41 (1.39, 1.45)	1.53 (1.52, 1.58)	1.55 (1.54, 1.60)	1.67 (1.67, 1.72)

CI – confidence interval, RR – relative risk

The age distribution of the population is critical to understanding the leukaemia burden. In particular, those aged ≥80 years are a high-risk group, accounting for 55% of leukaemia cases in 1990 and 63% in 2019. In terms of mortality, individuals aged ≥85 years accounted for 53% of leukaemia deaths in 1990 and 59% in 2019. For this age group, AML is the most common leukaemia subtype, while for mortality, other leukaemia leads (**Figure 2**, Panels A–F).

### Analysis of epidemiological characteristics of leukaemia and its subtypes by age-period-cohort with intrinsic estimator (APC-IE) model

We investigated the effect of age, period, and cohort on leukaemia incidence and mortality using the APC-IE model (**Figure 2**, Panels G and H, **Table 4**). The age effect for leukaemia shows a decline in incidence and mortality from zero to 30 and a significant increase from 30–79. Relative risks (RR) are lowest at ages 30–34 (RR for incidence = 0.296, RR for mortality = 0.314) and highest at ≥95 and older (RR for incidence = 6.264, RR for mortality = 6.959). These findings underscore a marked age-related increase in leukaemia incidence and mortality. The period effect from 1990 to 2019 for incidence and mortality rates increased modestly. The cohort effect showed incidence and mortality peaking between 1915 (RR = 1.528) and 1919 (RR = 1.691) and reaching a low from 2015 (RR = 0.312) to 2019 (RR = 0.289).

**Table 4:** APC-IE model of Incidence and mortality relative risks of leukaemia due to age, period, and cohort effects in global

	Incidence			Mortality		
**Factor**		**RR (95% CI)**	***P*-value**		**RR (95% CI)**	***P*-value**
Age in years						
*0*		2.42 (2.41, 2.43)	<0.01		1.36 (1.35, 1.37)	<0.01
*5*		0.91 (0.90, 0.91)	<0.01		0.62 (0.62, 0.63)	<0.01
*10*		0.49 (0.48, 0.49)	<0.01		0.46 (0.45, 0.46)	<0.01
*15*		0.40 (0.39, 0.40)	<0.01		0.43 (0.42, 0.43)	<0.01
*20*		0.34 (0.33, 0.34)	<0.01		0.36 (0.35, 0.36)	<0.01
*25*		0.31 (0.30, 0.31)	<0.01		0.32 (0.32, 0.33)	<0.01
*30*		0.30 (0.29, 0.30)	<0.01		0.31 (0.31, 0.32)	<0.01
*35*		0.39 (0.38, 0.39)	<0.01		0.35 (0.34, 0.35)	<0.01
*40*		0.39 (0.39, 0.40)	<0.01		0.39 (0.39, 0.40)	<0.01
*45*		0.50 (0.50, 0.51)	<0.01		0.46 (0.45, 0.46)	<0.01
*50*		0.66 (0.66, 0.67)	<0.01		0.59 (0.58, 0.59)	<0.01
*55*		0.88 (0.88, 0.89)	<0.01		0.79 (0.78, 0.79)	<0.01
*60*		1.16 (1.15, 1.16)	<0.01		1.08 (1.07, 1.08)	<0.01
*65*		1.46 (1.45, 1.47)	<0.01		1.48 (1.47, 1.49)	<0.01
*70*		1.94 (1.93, 1.95)	<0.01		2.05 (2.04, 2.06)	<0.01
*75*		2.38 (2.36, 2.39)	<0.01		2.72 (2.70, 2.73)	<0.01
*80*		2.67 (2.66, 2.68)	<0.01		3.44 (3.42, 3.46)	<0.01
*85*		3.43 (3.41, 3.45)	<0.01		4.73 (4.70, 4.76)	<0.01
*90*		3.78 (3.75, 3.81)	<0.01		5.63 (5.59, 5.68)	<0.01
*95*		6.26 (6.20, 6.33)	<0.01		6.96 (6.87, 7.05)	<0.01
Period						
*1990*		0.90 (0.89, 0.90)	<0.01		0.98 (0.98, 0.99)	<0.01
*1995*		0.95 (0.95, 0.96)	<0.01		1.00 (0.99, 1.00)	0.00
*2000*		0.99 (0.98, 0.99)	<0.01		1.00 (0.99, 1.00)	0.01
*2005*		1.01 (1.00, 1.01)	<0.01		0.99 (0.98, 1.00)	<0.01
*2010*		1.06 (1.05, 1.06)	<0.01		1.00 (1.00, 1.01)	0.01
*2015*		1.12 (1.11, 1.12)	<0.01		1.03 (1.03, 1.04)	<0.01
Cohort						
*1895*		1.38 (1.32, 1.44)	<0.01		1.60 (1.52, 1.67)	<0.01
*1900*		1.40 (1.37, 1.43)	<0.01		1.62 (1.58, 1.65)	<0.01
*1905*		1.44 (1.42, 1.46)	<0.01		1.64 (1.61, 1.66)	<0.01
*1910*		1.51 (1.50, 1.53)	<0.01		1.68 (1.66, 1.69)	<0.01
*1915*		1.53 (1.52, 1.54)	<0.01		1.69 (1.68, 1.70)	<0.01
*1920*		1.52 (1.51, 1.53)	<0.01		1.69 (1.67, 1.70)	<0.01
*1925*		1.51 (1.50, 1.52)	<0.01		1.67 (1.66, 1.68)	<0.01
*1930*		1.45 (1.44, 1.46)	<0.01		1.61 (1.60, 1.63)	<0.01
*1935*		1.39 (1.38, 1.40)	<0.01		1.54 (1.52, 1.55)	<0.01
*1940*		1.34 (1.33, 1.35)	<0.01		1.46 (1.45, 1.47)	<0.01
*1945*		1.28 (1.27, 1.29)	<0.01		1.38 (1.37, 1.39)	<0.01
*1950*		1.23 (1.22, 1.24)	<0.01		1.29 (1.28, 1.30)	<0.01
*1955*		1.16 (1.15, 1.17)	<0.01		1.19 (1.18, 1.20)	<0.01
*1960*		1.10 (1.09, 1.10)	<0.01		1.08 (1.07, 1.09)	<0.01
*1965*		1.09 (1.08, 1.10)	<0.01		1.02 (1.01, 1.03)	<0.01
*1970*		1.03 (1.03, 1.04)	<0.01		0.94 (0.93, 0.95)	<0.01
*1975*		0.97 (0.96, 0.97)	<0.01		0.85 (0.84, 0.86)	<0.01
*1980*		0.90 (0.89, 0.90)	<0.01		0.77 (0.76, 0.78)	<0.01
*1985*		0.85 (0.84, 0.86)	<0.01		0.72 (0.71, 0.73)	<0.01
*1990*		0.81 (0.80, 0.81)	<0.01		0.67 (0.67, 0.68)	<0.01
*1995*		0.66 (0.66, 0.67)	<0.01		0.57 (0.57, 0.58)	<0.01
*2000*		0.54 (0.54, 0.55)	<0.01		0.49 (0.48, 0.49)	<0.01
*2005*		0.45 (0.44, 0.45)	<0.01		0.41 (0.41, 0.42)	<0.01
*2010*		0.37 (0.37, 0.38)	<0.01		0.35 (0.34, 0.35)	<0.01
*2015*		0.31 (0.31, 0.32)	<0.01		0.29 (0.28, 0.30)	<0.01

APC-IE – age-period-cohort with intrinsic estimator, CI – confidence interval, RR – relative risk

The APC-IE revealed diverse patterns across leukaemia subtypes (Figure S2 and Table S4 in the **Online Supplementary Document**). In terms of age, children under five years have the highest risk for ALL, while individuals >95 years face the highest risk for AML and other leukaemia. In paediatric leukaemia and its subtypes in the group aged zero to 15 years, the incidence and mortality rates followed a descending order across age groups – zero to five years, five to 10 years, 10–15 years – with this trend being particularly pronounced in ALL (Figure S2, Panels A and F in the **Online Supplementary Document**). This phenomenon suggests that genetic factors and congenital immune system abnormalities may constitute significant risk factors for paediatric leukaemia. For CLL and CML, peak risk occurs at the age of 90 years. Period effects indicated that incidence risks of ALL and mortality risks of AML have risen particularly from 1990 to 2015 (**Figure 2**, Panels A and G). Cohort effects showed that recent birth cohorts have lower risks of leukaemia for all subtypes.

### Analysis of epidemiological trends in leukaemia and its subtypes across SDI regions – 21 GBD regions and 204 countries

Between 1990–2019, leukaemia ASIR only increased in high SDI regions, with Western Europe displaying notably (AAPC = 0.9) (**Table 5**, Figure S3, Panel A in the **Online Supplementary Document**). ALL ASIR increased in high to middle SDI regions, especially in East Asia (AAPC = 5.09). AML ASIR increased across all SDI regions but showed a notable decrease in Eastern Europe (AAPC = –1.27) (Figures S4–5, Panels A and B and Tables S4–5 in the **Online Supplementary Document**). CML ASIR increased in high and high-middle SDI regions, with Western Europe standing out (AAPC = 1.52). ASIR for CLL and other leukaemia rose in all SDI regions (Figures S6–8, Panels A and B and Tables S4–5 in the **Online Supplementary Document**).

**Table 5:** AAPC in incidence, mortality, and DALYs of leukaemia across SDI regions, GBD regions, 1990–2019

	Incidence		Mortality		DALYs	
**Location**	**AAPC (95% CI)**	***P*-value**	**AAPC (95% CI)**	***P*-value**	**AAPC (95% CI)**	***P*-value**
Global	–0.54 (–0.62, –0.45)	<0.01	–1.07 (–1.13, –1.01)	<0.01	–1.62 (–1.69, –1.54)	<0.01
SDI						
*High*	0.24 (0.05, 0.43)	0.01	–0.9 (–0.96, –0.84)	<0.01	–1.41 (–1.47, –1.35)	<0.01
*High-middle*	–0.15 (–0.29, –0.01)	0.03	–1.24 (–1.34, –1.13)	<0.01	–1.88 (–2.03, –1.74)	<0.01
*Middle*	–0.91 (–1.01, –0.81)	<0.01	–1.14 (–1.2, –1.09)	<0.01	–74 (–1.83, –1.65)	<0.01
*Low-middle*	–0.98 (–1.06, –0.9)	<0.01	–0.84 (–0.94, –0.75)	<0.01	–1.40 (–1.52, –1.28)	<0.01
*Low*	–0.81 (–0.91, –0.71)	<0.01	–0.74 (–0.81, –0.66)	<0.01	–1.19 (–1.32, –1.06)	<0.01
Region						
*Andean Latin America*	–0.09 (–0.46, 0.29)	0.65	–0.17 (–0.52, 0.18)	0.34	–0.48 (–0.81, –0.14)	<0.01
*Australasia*	0.34 (0.01, 0.67)	0.05	–0.73 (–0.89, –0.57)	<0.01	–1.2 (–1.3, –1.1)	<0.01
*Caribbean*	–0.27 (–0.41, –0.14)	<0.01	–0.43 (–0.57, –0.28)	<0.01	–0.63 (–0.76, –0.51)	<0.01
*Central Asia*	–1.29 (–1.43, –1.15)	<0.01	–0.97 (–1.16, –0.78)	<0.01	–1.6 (–1.74, –1.46)	<0.01
*Central Europe*	0.64 (0.55, 0.73)	<0.01	–0.54 (–0.69, –0.39)	<0.01	–1.15 (–1.28, –1.01)	<0.01
*Central Latin America*	–0.19 (–0.23, –0.15)	<0.01	–0.26 (–0.3, –0.21)	<0.01	–0.42 (–0.47, –0.38)	<0.01
*Central sub-Saharan Africa*	–1.17 (–1.33, –1.01)	<0.01	–0.83 (–0.94, –1.64)	<0.01	–1.36 (–1.52, –1.19)	<0.01
*East Asia*	–0.64 (–0.96, –0.31)	<0.01	–1.8 (–1.96, –1.64)	<0.01	–2.38 (–2.63, –2.14)	<0.01
*Eastern Europe*	–0.27 (–1.06, 0.53)	0.51	–0.96 (–1.58, –0.33)	<0.01	–1.75 (–2.41, –1.08)	<0.01
*Eastern sub-Saharan Africa*	–1.2 (–1.3, –1.11)	<0.01	–1.0 (–1.1, –0.91)	<0.01	–1,66 (–1.84, –1.49)	<0.01
*High-income Asia Pacific*	0.47 (0.24, 0.7)	<0.01	–1.47 (–1.56, –1.38)	<0.01	–2.17 (–2.37, –1.98)	<0.01
*High-income North America*	–0.39 (–0.58, –0.19)	<0.01	–0.65 (–0.83, –0.47)	<0.01	–1.13 (–1.27, –0.98)	<0.01
*North Africa and the Middle East*	–0.7 (–0.81, –0.58)	<0.01	–0.88 (–0.95, –0.81)	<0.01	–1.33 (–1.39, –1.27)	<0.01
*Oceania*	–0.14 (–0.35, 0.07)	0.18	–0.31 (–0.49, –0.14)	<0.01	–0.33 (–0.55, –0.10)	<0.01
*South Asia*	–0.75 (–0.99, –0.52)	<0.01	–0.84 (–1.12, –0.56)	<0.01	–1.27 (–1.52, –1.02)	<0.01
*Southeast Asia*	–0.8 (–0.84, –0.76)	<0.01	–0.67 (–0.74, –0.59)	<0.01	–1.11 (–1.16, –1.07)	<0.01
*Southern Latin America*	–0.15 (–0.37, 0.08)	0.19	–0.59 (–0.71, –0.48)	<0.01	–0.93 (–1.07, –0.78)	<0.01
*Southern sub-Saharan Africa*	–0.09 (–0.24, 0.06)	0.26	–0.22 (–0.56, 0.11)	0.19	–0.58 (–0.74, –0.42)	<0.01
*Tropical Latin America*	–0.74 (–0.8, –0.67)	<0.01	–0.65 (–0.77, –0.56)	<0.01	–1.12 (–1.2, –1.05)	<0.01
*Western Europe*	0.9 (0.74, 1.06)	<0.01	–0.67 (–0.77, –0.56)	<0.01	–1.31 (–1.44, –1.17)	<0.01
*Western sub-Saharan Africa*	0.07 (–0.03, 0.17)	0.19	–0.01 (–0.1, 0.08)	0.84	–0.2 (–0.32¸–0.08)	<0.01

AAPC – average annual percent change, CI – confidence interval, DALYs – disability-adjusted life years, SDI – socio-demographic index

The global leukaemia ASDR decreased in all SDI regions, with East Asia experiencing the most significant decrease (AAPC = –1.8) (**Table 5**, Figure S3, Panel B in the **Online Supplementary Document**). In ALL, the ASDR decreased in all SDI regions, especially Australasia (AAPC = –2.12). However, Central Latin America and Andean Latin America showed increased ASDR (AAPC = 0.54 and 0.36). AML ASDR increased in all SDI regions, especially in Andean Latin America (AAPC = 1.4). The highest increase of CLL ASDR was in Central Europe (AAPC = 1.75). CML and other leukaemia ASDR decreased in all SDI regions (Figure S4–8, Panels C and D and Table S5 in the **Online Supplementary Document**).

ASR-DALYs for leukaemia decreased globally across all SDI regions (**Table 5**, Figure S3, Panels E and F in the **Online Supplementary Document**). In ALL, despite the overall declining trend, Central Latin America, Andean Latin America, and East Asia increased (AAPC = 0.29, 0.11, and 0.06). AML showed decreasing ASR-DALYs in high and high-middle SDI regions (AAPC = –0.24 and –0.50), while other regions, especially Andean Latin America, witnessed increases (AAPC = 1.08). CLL had declining ASR-DALYs in high SDI and high-middle SDI regions (AAPC = –1.05 and –0.05) but increased in Central Sub-Saharan Africa, East Asia, and Central Europe (AAPC = 2.35, 1.72, and 1.59). CML and other leukaemia presented declining ASR-DALYs across all SDI regions (Figures S4–8, Panels E and F and Table S5 in the **Online Supplementary Document**).

**Figure 3:**
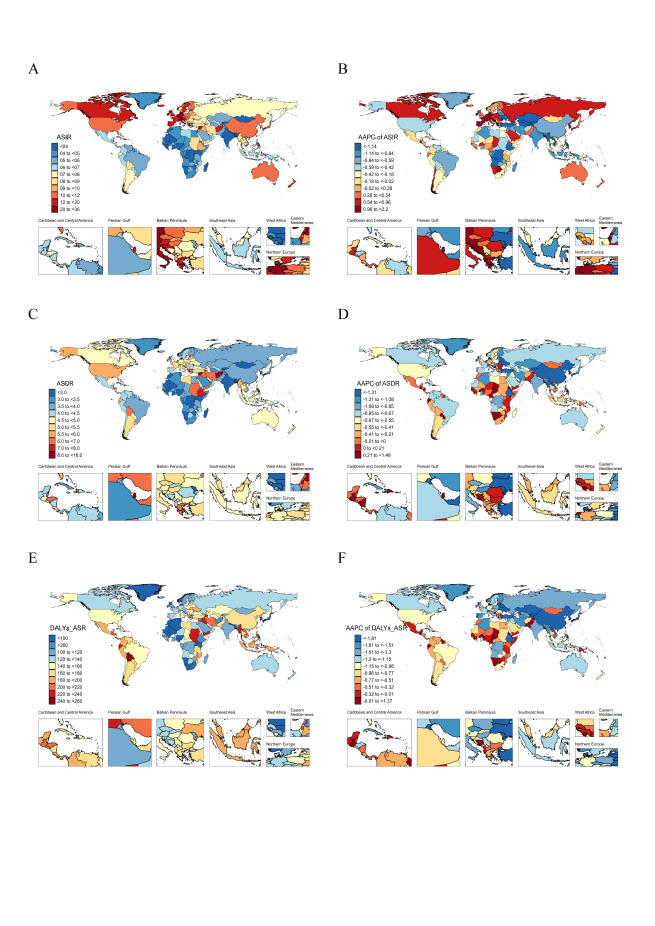
The global trends of leukaemia by countries and territories. **Panel A.** The ASIR of leukaemia in 2019. **Panel B.** The AAPC in ASIR of leukaemia from 1990 to 2019. **Panel C.** The ASDR of leukaemia in 2019. **Panel D.** The AAPC in ASDR of leukaemia from 1990 to 2019. **Panel E.** The ASR of DALYs of leukaemia in 2019. **Panel F.** The AAPC in ASR of DALYs of leukaemia from 1990 to 2019. AAPC – average annual percentage changes, ASDR – age-standardised death rate, ASIR – age-standardised incidence rate, ASR – age-standardised rate, DALYs – disability-adjusted life years.

Leukaemia epidemiology across 204 countries highlights San Marino, Monaco, and Andorra with the highest ASIRs, and the Syrian Arab Republic, Afghanistan, and Monaco with the highest ASDRs. Regarding ASR-DALYs, the Syrian Arab Republic, Afghanistan, and Haiti are the top three countries. Maldives, Ethiopia, and the Republic of Moldova showed commendable ASIR, ASDR, and DALY control (**Figure 3****,** Panels A–F). For ALL, Taiwan (province of China) and Guatemala have shown leading increases in ASIR, ASDR, and DALYs, highlighting significant epidemiological challenges. AML saw substantial challenges in Guatemala, Taiwan (province of China), El Salvador, and Thailand, all excelling in control. CLL presented challenges in Poland, Jamaica, and Estonia, whereas the Netherlands, Guam, Kyrgyzstan, and Switzerland showed effective control. The disease burden of CML was significant in Lesotho and Jamaica. At the same time, challenges associated with other leukaemia were notable in the Netherlands, Chad, and Lesotho (Figure S4–8, Panels B, D, F in the **Online Supplementary Document**).

### Decomposition analysis of deaths, incidence, and DALYs for leukaemia and its subtypes

**Figure 4:**
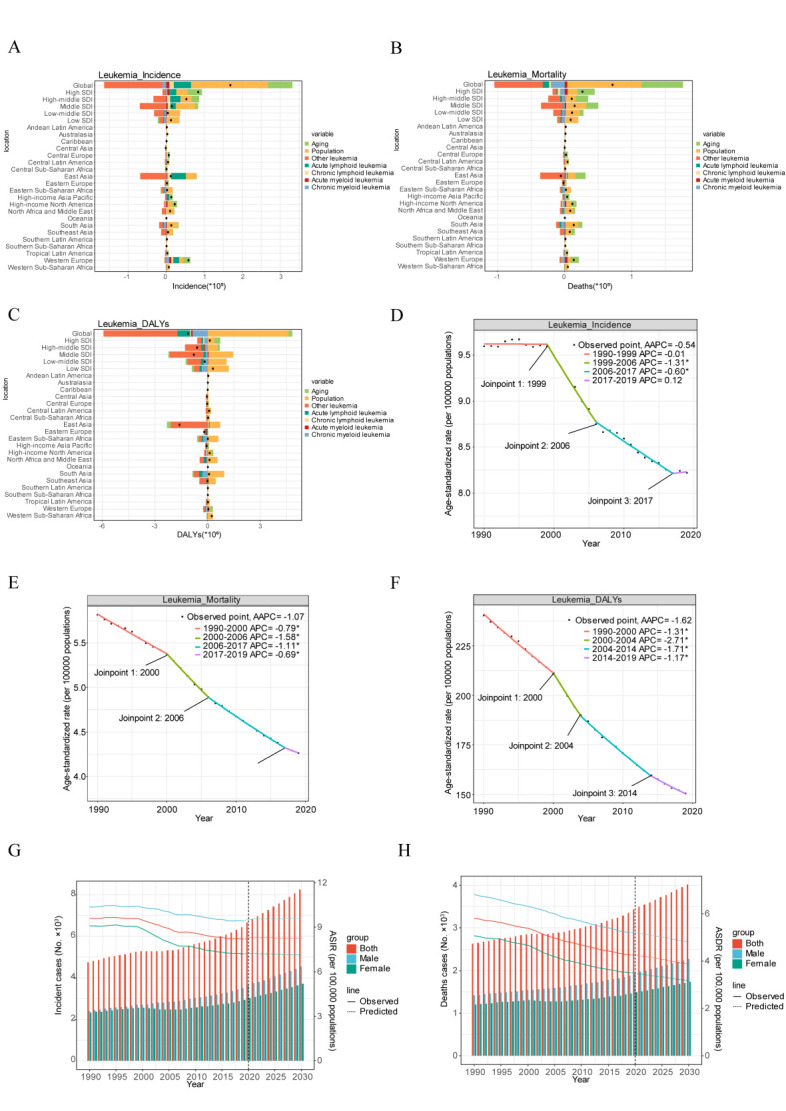
Decomposition and joinpoint analysis of global leukaemia trends in incidence, mortality, and DALYs from 1990 to 2019, with projections to 2030. **Panel A.** Decomposition analysis of leukaemia incidence. **Panel B.** Decomposition analysis of leukaemia mortality. **Panel C.** Decomposition analysis of leukaemia DALYs. These analyses are based on population-level determinants of population growth, ageing, and epidemiological change for five leukaemia subtypes from 1990 to 2019 at the global level, SDI quintile level, and 21 regional levels. The black dot represents the overall value of change contributed by all seven components. For each component, the magnitude of a positive value indicates a corresponding increase in leukaemia incidence attributed to the component, while the magnitude of a negative value indicates a corresponding decrease in leukaemia incidence attributed to the related component. **Panel D.** Joinpoint regression analysis of global leukaemia incidence. **Panel E.** Joinpoint regression analysis of global leukaemia mortality. **Panel F.** Joinpoint regression analysis of global leukaemia DALYs. (*P < 0.05, **P < 0.01, ***P < 0.001). **Panel G.** Projections of global leukaemia ASIRs in both sexes. **Panel H.** Projections of global leukaemia ASDR in both sexes. For each group, the right side of the dotted line is the observation value from 1990 to 2019. On the left of the dotted line are observation values from 1990 to 2019, and on the right of the dotted lines are predicted values from 2020 to 2030. AAPC – average annual percentage changes, ASDR – age-standardised death rate, ASIR – age-standardised incidence rate, DALYs – disability-adjusted life years.

Over the past three decades, global leukaemia incidence has risen, especially in high SDI regions, due to population growth (120.05%) and ageing (38.13%). Ageing’s impact is most pronounced in the middle (51.49%) and high (45.52%) SDI regions. Notably, Eastern sub-Saharan Africa has experienced a significant increase due to population expansion (740.53%). Leukaemia-related deaths have also increased, particularly in high SDI regions, driven by ageing (96.75%) and population growth (56.88%). Ageing predominantly affects high-middle SDI regions (180.06%). Eastern sub-Saharan Africa has experienced a notable mortality increase due to population growth (499.85%). DALYs for leukaemia have generally decreased, except in low and high SDI regions, where they have increased due to different factors (**Figure 4**, Panels A–C, Table S6 in the **Online Supplementary Document**).

Regarding subtypes, ALL incidence notably increased, especially in high and middle SDI regions, correlating with a rise in DALYs (Figure S9, Panels A–C and Table S7 in the **Online Supplementary Document**). AML significantly increased in high SDI regions, primarily due to demographic factors (Figure S9, Panels D–F and Table S7 in the **Online Supplementary Document**). Due to ageing, CLL incidence and mortality rose in high and high-middle SDI regions. The DALY surge in East Asia is attributed to disease epidemiology, indicating the necessity for enhanced medical interventions (Figure S9, Panels A–J and Table S7 in the **Online Supplementary Document**). Despite a demographic uptick in CML incidence in high SDI regions, mortality and DALYs were effectively managed (Figure S10, Panels A–C and Table S7 in the **Online Supplementary Document**). Other leukaemia globally declined, notably in the middle SDI region and East Asia, reflecting shifting epidemiological patterns (Figure S10, Panels D–F and Table S7 in the **Online Supplementary Document**).

### Joinpoint analysis of the incidence and mortality rates of leukaemia and its subtypes

Joinpoint regression analysis showed that the disease burden of leukaemia was effectively controlled between 2000–06, with a significant turning point appearing between 1999–2000. ASIR significantly decreased from 1999 to 2006 (AAPC = –1.31; *P* < 0.05), slowing down until 2017 (AAPC = –0.60; *P* < 0.05) (**Figure 4**, Panel D). ASDR also declined notably between 2000–06 (AAPC = –1.58; *P* < 0.05), continuing until 2017 (AAPC = –1.11; *P* < 0.05) (**Figure 4**, Panel E). DALYs significantly reduced between 2000–04 (AAPC = –2.17; *P* < 0.05), with a continued decrease until 2014 (AAPC = –1.71, *P* < 0.05) (**Figure 4**, Panel F). A similar pattern was observed in the ASR of prevalence (Figure S8, Panel A in the **Online Supplementary Document**).

From 2006 to 2010, the burden of ALL increased significantly (Figure S11, Panel A in the **Online Supplementary Document**). In contrast, the burden of AML significantly increased from 1997 to 2000, probably due to environmental and economic factors (Figure S11, Panel B in the **Online Supplementary Document**). CLL’s turning point around 2002 is consistent with the use of targeted drugs (Figure S11, Panel C in the **Online Supplementary Document**). The disease burden of CML shifted markedly between 2000–03, probably due to effective tyrosine kinase inhibitors treatments (Figure S11, Panel D in the **Online Supplementary Document**). Other leukaemia experienced a turning point around 2000, possibly influenced by the World Health Organization (WHO) guidelines and therapeutic advances (Figure S11, Panel E in the **Online Supplementary Document**).

### Projections of leukaemia incidence and mortality by 2030 using the BAPC model

Using the BAPC model, our projections indicate that by 2030, the global incidence of leukaemia cases will reach 721 091, with an ASIR of 8.28 per 100 000 population (**Figure 4**, Panel G, Table S8 in the **Online Supplementary Document**). Concurrently, the number of leukaemia-related deaths is expected to reach 343 713 by 2030, with the ASDR declining to 3.87 per 100 000 population (**Figure 4**, Panel H, Table S8 in the **Online Supplementary Document**). Gender bias is anticipated to persist in 2030.

### The trends and correlations between SDI and leukaemia incidence and mortality

**Figure 5:**
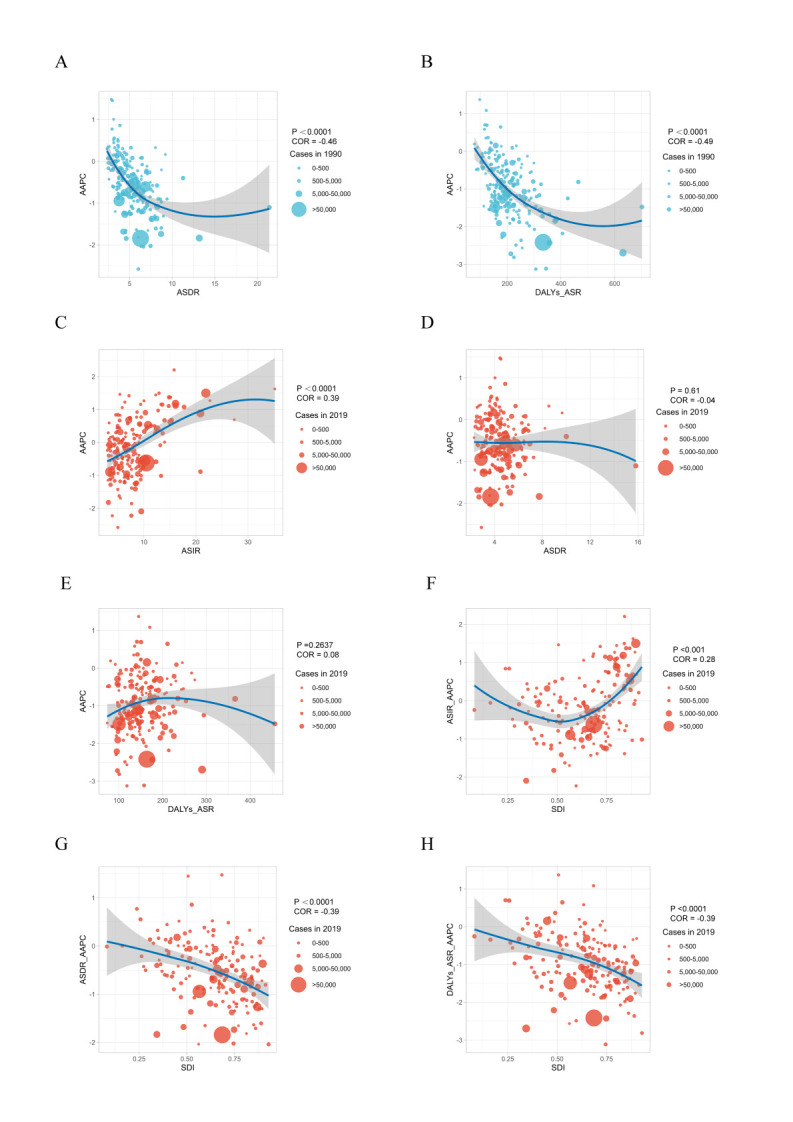
The correlation analyses of AAPCs-ASIR, AAPCs-ASDR, AAPCs-ASR of DALYs and AAPCs-SDI in 1990 and 2019. **Panel A.** The correlation between ASDR and AAPC of ASDR of 1990 in 195 countries or territories. **Panel B.** The correlation between age-standardised DALYs rate and its AAPC in 1990 in 195 countries or territories. **Panel C.** The correlation between ASIR and AAPC of ASIR of 2019 in 195 countries or territories. **Panel D.** The correlation between ASDR and AAPC of ASDR 2019 in 195 countries or territories. **Panel E.** The correlation between age-standardised DALYs rate and its AAPC in 1990 in 195 countries or territories. **Panel F.** The correlation between AAPC of ASIR and SDI of 2019 in 195 countries or territories. **Panel G.** The correlation between AAPC of ASDR and SDI of 2019 in 195 countries or territories. **Panel H.** The correlation between AAPC of age-standardised DALYs rate and SDI of 2019 in 195 countries or territories. The circle size represents the number of leukaemia patients in one country or territory. AAPC – average annual percentage change, ASDR – age-standardised death rate, ASIR – age-standardised incidence rate, DALYs – disability-adjusted life years, SDI – socio-demographic index.

Next, we analysed the relationships between SDI and leukaemia incidence, mortality, and DALYs. A negative trend was observed between ASDR and AAPC (correlation coefficient (r) = –0.46; *P* < 0.05) and the ASR-DALYs and AAPC (r = –0.49; *P* < 0.05), suggesting an overestimation of mortality in high-mortality areas in 1990. (**Figure 5**, Panels A and B). However, no correlation was observed between the ASIR and its AAPCs (Figure S12, Panel A in the **Online Supplementary Document**). In 2019, there was a positive correlation between ASIR and its AAPC (r = 0.39; *P* < 0.05), highlighting an increase in incidence rates in countries with initially higher rates (**Figure 5**, Panel C). There was no correlation between ASDR or ASR-DALYs and their AAPCs in 2019 (**Figure 5**, Panels D and E). We analysed data from 204 countries and found that SDI did not correlate significantly with the AAPC of ASIR in 2019 (**Figure 5**, Panel F). However, the AAPC of ASDR and ASR-DALY were negatively correlated with SDI (r = –0.39; *P* < 0.05) (**Figure 5**, Panels G and H). These findings suggest that countries with higher SDI levels do not necessarily experience higher leukaemia incidence rates but tend to have lower mortality and DALY rates. This underscores the intricate interplay between sociodemographic factors and the influence of advanced health care in high-SDI regions on improving leukaemia outcomes.

**Figure 6:**
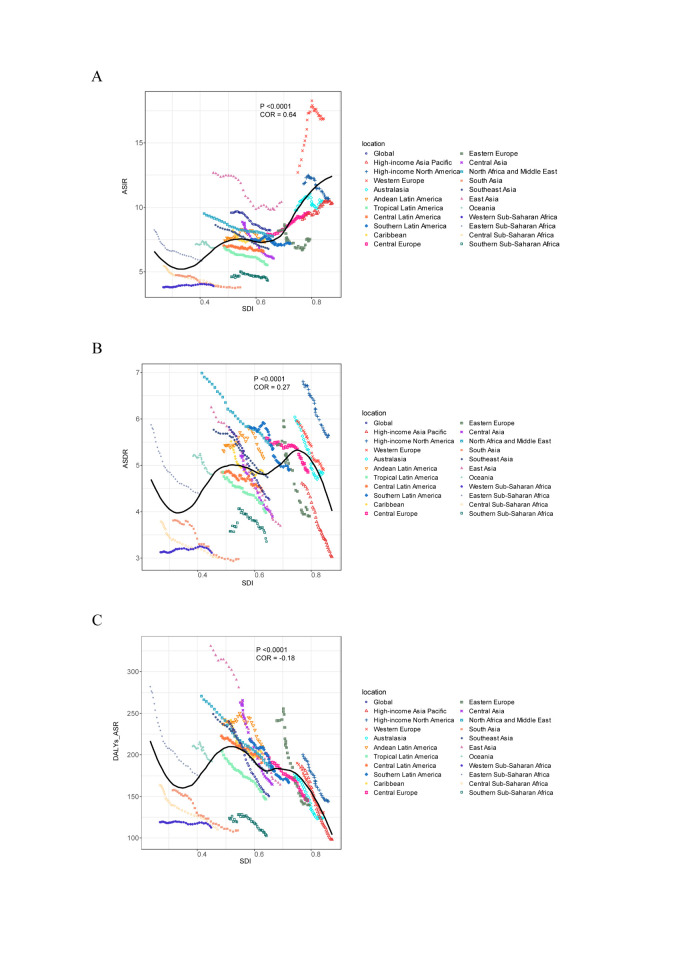
The change trends and correlation analyses of ASRs and SDI from 1990 to 2019. **Panel A.** The change trends and correlation of ASIR and SDI from 1990 to 2019 in global and 21 regions. **Panel B.** The change trends and correlation of ASDR and SDI from 1990 to 2019 in global and 21 regions. **Panel C.** The change trends and correlation of age-standardised DALY rate and SDI from 1990 to 2019 in global and 21 regions. AAPC – average annual percentage change, ASDR – age-standardised death rate, ASIR – age-standardised incidence rate, DALYs – disability-adjusted life years, SDI – socio-demographic index.

We examined the correlation between SDI and ASIR, ASDR, and ASR-DALY in 21 GBD regions from 1990 to 2019. Compared with ASDR, ASIR showed a more significant positive correlation with SDI (r = 0.64; *P* < 0.05). There was no correlation between ASDR or ASR-DALY and SDI (r = –0.18; *P* < 0.05). For example, in Western Europe, the SDI index in 1990 was 0.75, indicating that the SDI was in the medium to high range. By 2019, the SDI index had increased to 0.84, indicating a high SDI zone. At the same time, as the SDI index developed, the region’s ASIR showed significant growth, with an AAPC = 0.9. At the same time, ASDR and ASR-DALY decreased slightly (**Figure 6**, Panels A–D).

### Frontier analysis of ASDR and age-standardised DALYs rates

Frontier analysis of ASDR and ASR-DALYs for leukaemia from 1990 to 2019 revealed substantial variation in the intensity of management and control across countries with different SDI levels.

**Figure 7:**
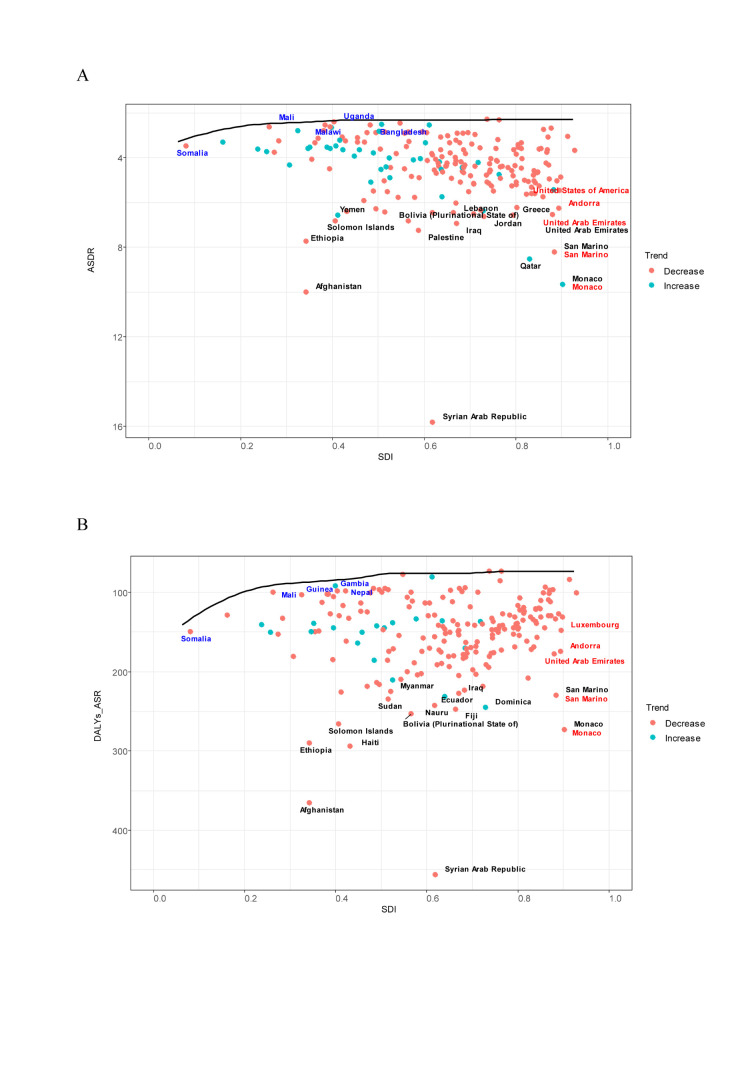
Frontier analysis of SDI, ASDR, and age-standardised DALY rate in 2019. **Panel A.** Frontier analysis of SDI and ASDR in 2019. **Panel B.** Frontier analysis of SDI and ASR of DALYs in 2019. The frontier is solid black; countries and territories are represented as dots. The top 15 countries with the largest effective difference are labelled in black; examples of frontier countries with low SDI (<0.5) and low effective difference are labelled in blue, and examples of countries and territories with high SDI (>0.85) and relatively high effective difference for their level of development are labelled in red. Red dots indicate an increase in ASR from 1990 to 2019. Blue dots indicate a decrease in ASR between 1990 and 2019. ASR – age-standardised rate, DALYs – disability-adjusted life years, SDI – socio-demographic index.

For ASDR, among countries with low-middle SDI, Somalia, Mali, Malawi, Uganda, and Bangladesh exemplify the most efficient control measures. Conversely, among high SDI countries, the United States of America, Andorra, the United Arab Emirates, San Marino, and Monaco were found to have suboptimal control. The countries with the most significant potential for progress in disease control were Yemen, Bolivia, and Qatar, among others (**Figure 7**, Panel A, Figure S13, Panel A and Table S9 in the **Online Supplementary Document**). For DALYs, countries such as Somalia, Mali, Gambia, Guinea, and Nepal, all in the low-middle SDI range, show commendable disease control. In contrast, high SDI countries such as Luxembourg, Andorra, the United Arab Emirates, San Marino, and Monaco face challenges in effective disease management (**Figure 7**, Panel B, Figure S13, Panel B and Table S9 in the **Online Supplementary Document**). This phenomenon suggests that socio-demographic progression is typically correlated with an increased effective gap, suggesting that countries or regions with a higher SDI have better prospects for burden reduction.

### The leukaemia-related mortality and DALYs attributable risk factors

**Figure 8:**
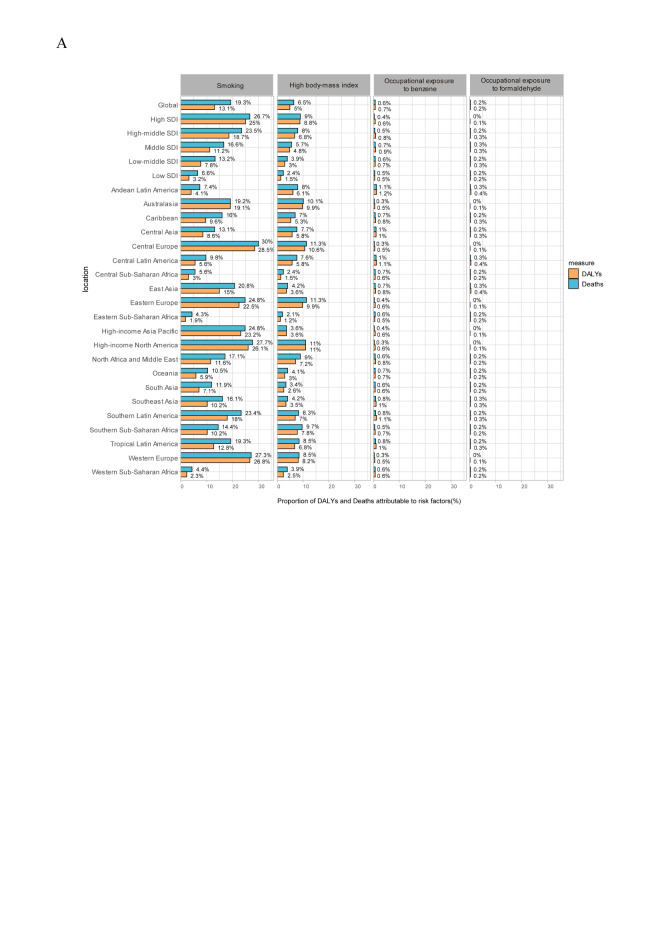
Risk factors contributing to leukaemia-related death and DALYs. **Panel A.** The four risk factors contributing to leukaemia-related death and DALYs from 1990 to 2019 in the globe and different regions. DALYs – disability-adjusted life years, SDI – socio-demographic index.

We examined potential determinants associated with leukaemia mortality and DALYs. Our analysis revealed four prominent risk factors – tobacco smoking, increased BMI, occupational benzene exposure, and formaldehyde exposure. Tobacco smoking had the most substantial impact, accounting for 13.1% of DALYs and 19.3% of deaths, while high BMI accounted for 6.5% and 5%, respectively. Notably, the detrimental effects of smoking and high BMI were more pronounced in higher SDI countries (**Figure 8**, Panel A).

## DISCUSSION

This study analysed the global burden of leukaemia and its five subtypes from 1990 to 2019, with projections to 2030, confirming leukaemia’s significant role in the global cancer burden [1,31]. Despite increases in absolute leukaemia cases and deaths, there has been a decline in AISRs and ASDRs, a trend expected to continue until 2023. Notably, the ‘other leukaemia’ category, which includes many rare subtypes, has seen marked reductions in disease burden. The significant decrease in leukaemia ASR is primarily attributed to advances in medical technology and treatment methods, alongside preventive measures like public health advocacy. This decline leads to improved patient outcomes, potentially reducing burdens on health care systems. Additionally, it may incentivise increased research in leukaemia and prevention efforts to sustain reductions in incidence.

Globally, male dominance is present in all leukaemia subtypes, which hormones, genetics, and lifestyle choices may influence. The APC-IE method helps to decompose the time trends to provide unbiased and efficient estimates. Significant increase in leukaemia cases began in the 40–45 age group and peaks in the 70+ years age group. By 2032, the global population aged 65+ years is projected to exceed 700 million, heralding a significant increase in leukaemia risk and disease burden [32]. This underscores the critical need for increased health education and prevention strategies targeted at this population. Period effects suggest an upward trend in incidence, possibly driven by unhealthy lifestyles associated with global economic progress and urbanisation [22,33]. Furthermore, advances in diagnostic technologies have improved early diagnosis through precise procedures such as immunophenotyping, cytogenetics, and molecular genetics [34]. Notably, leukaemia-related mortality has shown minimal change from 1990 to 2015. From a socioeconomic perspective, the global economic boom has supported health care reforms and medical technological advances. At the same time, public health initiatives and comprehensive epidemiological studies have forged effective countermeasures against the escalating incidence of leukaemia. The reduction in cohort effects reflects worldwide endeavours to progress health care and disseminate awareness regarding preventive health care measures.

Population growth and ageing are the primary factors contributing to the increasing burden of leukaemia, pronounced in high SDI regions. The ageing process is associated with numerous tumour-related events, including genomic instability, disruption of protein homeostasis, and metabolic dysregulation [35,36]. Among older adults, clonal haematopoiesis driver genes, such as DNA methyltransferase 3A, tet methylcytosine dioxygenase 2, and additional sex combs like 1, are mutated the most frequently and are associated with all-cause mortality and risk of leukaemia [37,38].

Our study indicates that the period between 1999–2000 marked a significant turning point in leukaemia epidemiology. This can be attributed to several factors. First, in 1999, WHO guidelines emphasised the importance of molecular genetic abnormalities in disease diagnosis and classification, leading to improved accuracy in leukaemia diagnosis. Cases previously diagnosed as other leukaemia were reclassified. For instance, refractory anaemia with excess blasts in transformation of myelodysplastic syndrome was categorised alongside AML due to clinical similarities. Diagnosis of ALL now requires ≥25% immature lymphocytes in the bone marrow; otherwise, it is classified as lymphoma. Significant cytogenetic abnormalities such as BCR-ABL fusion gene, TEL-AML1 fusion gene, MLL translocations, TCF3-PBX1 fusion gene, and MYC rearrangements are considered. Immunophenotyping has become essential for diagnosing acute leukaemia of ambiguous lineage. Second, treatment with tyrosine kinase inhibitors has notably improved the prognosis of CML patients [39,40]. Third, rituximab has been approved for CLL, enhancing clinical outcomes [41,42]. Furthermore, it is noteworthy that, in 2014, in treating ALL, the utilisation of tyrosine kinase inhibitors for Philadelphia chromosome-positive ALL and bispecific T-cell engager (CD19/CD3) monoclonal antibody therapy led to decreased ASDR. However, neither conventional chemotherapy nor advancements in targeted therapy significantly improved ASDR reduction in AML. For CLL, clinical applications of rituximab, ofatumumab, and idelalisib contributed to decreased ASDR.

Smoking and obesity emerged as prominent risk factors for leukaemia-related mortality and DALYs. Studies dating back to 1987 have consistently shown a 1.5 to 2.0-fold increased risk of leukaemia associated with smoking, which escalates with prolonged exposure [43]. Notably, parental smoking pre-conception and exposure to hydrocarbons are significant risk factors for paediatric ALL [44]. Cigarette smoke, rich in carcinogens like polycyclic aromatic hydrocarbons and benzene, induces DNA damage in hematopoietic cells and triggers chronic inflammation and immune dysregulation [45–48]. Additionally, smoking induces epigenetic alterations and adipose tissue inflammation, perturbing gene expression patterns relevant to leukemogenesis [49,50]. Similarly, obesity-associated insulin resistance and hyperinsulinemia promote cell proliferation, exacerbating leukemogenesis [51].

Economic factors and the quality of health care systems have a significant impact on the disease burden of leukaemia. Higher economic levels typically result in better access to medical resources, promoting early detection and diagnosis. High-quality health care systems can provide advanced diagnostics and effective treatments more effectively. In middle to high-middle SDI countries, strategies for leukaemia control should focus on improving early screening and diagnosis, enhancing public awareness through education programs, and providing free screening services. Investing in health care infrastructure, reducing treatment costs through governmental subsidies or insurance schemes, and promoting research and innovation are also critical. Future leukaemia research should address data gaps in developing regions with lower SDI levels, investigate emerging risk factors such as environmental pollution, occupational exposure, and lifestyle changes, and explore leukaemia biomarkers and genetic factors using advanced molecular biology techniques. We can optimise current findings by delineating these research priorities to enhance patient prognosis.

Our study provides a unique and comprehensive overview of the global burden of leukaemia, including its five subtypes, using the most recent data and projecting disease trends into the next decade. However, as with many diseases analysed in the GBD study, the accuracy of our leukaemia model is highly dependent on the quality and quantity of data collected [52–54]. Developed nations’ superior reporting and early diagnosis may inflate estimates, while low SDI countries might underestimate due to limited data reliability from lacking diagnostic tools. Underreporting and diagnostic failures, especially in underdeveloped countries, may introduce bias and underestimate hematologic malignancy estimates. Despite these challenges, our study still lays a valuable foundation for future research and developing targeted interventions and strategies.

## CONCLUSIONS

The global burden of leukaemia increased slightly from 1990 to 2019, a trend expected to persist until 2030. The period between 1999–2000 marked a turning point in mitigating the global leukaemia burden, possibly due to the WHO guidelines and advancements in targeted therapies, facilitating precise diagnosis and treatment. The rise in leukaemia burden primarily occurred in the middle to high-SDI countries, necessitating reinforced preventive measures targeting key risk factors like smoking and obesity. Future research should prioritise investigating emerging risk factors, exploring leukaemia biomarkers, and enhancing treatment efficacy. Our study has significant implications for leukaemia management and formulating pertinent strategies.

## Additional material


Online Supplementary Document
