# Supplementary material for: Global burden, risk factor analysis, and prediction study of leukaemia from 1990 to 2030
Source: J Glob Health. 2024 Aug 23;14:04150. doi: 10.7189/jogh.14.04150 (PMC11345035; doi:10.7189/jogh.14.04150)
Supplement: Online Supplementary Document [file jogh-14-04150-s001.pdf]

Supplementary Figures and Tables

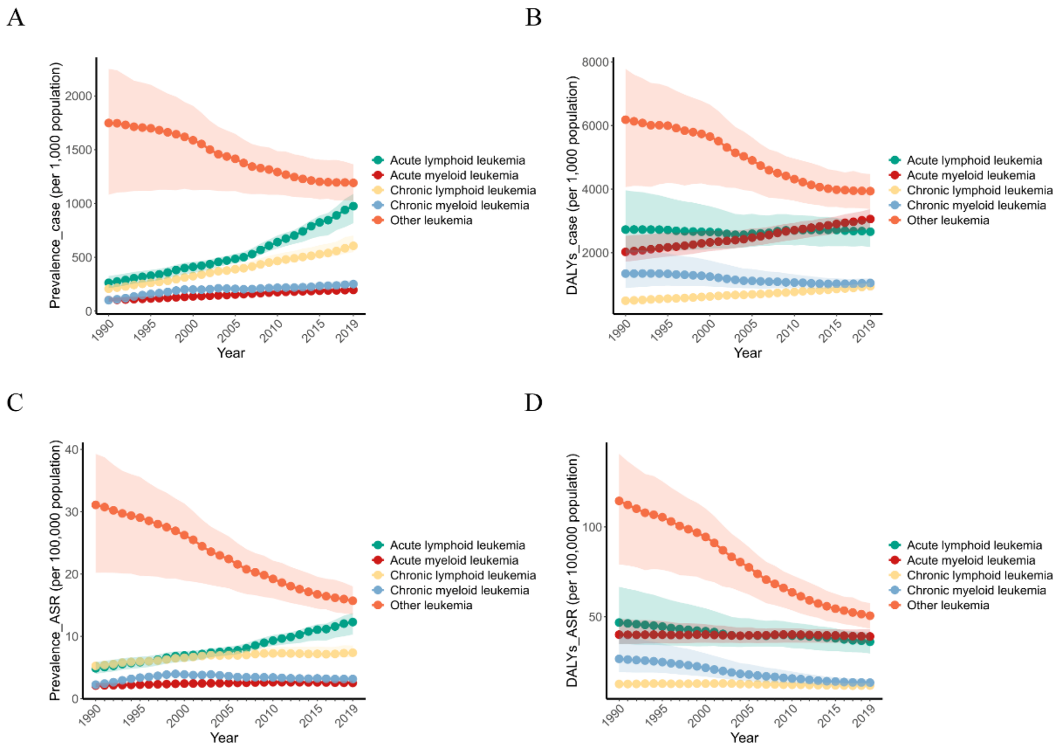

Figure S1 Global trends in prevalence and DALYs for leukemia and its five subtypes from 1990 to 2019. A The prevalence cases of leukemia from 1990 to 2019. B The number of DALYs case due to leukemia from 1990 to 2019. C ASR of prevalence and AAPC in leukemia over the last 30 years. D ASR of DALYs and AAPC in leukemia over the last 30 years. Note: ASR age standardized rate, ASIR age standardized incidence rate, ASDR age standardized death rate, AAPC average annual percentage changes, WHO World Health Organization.

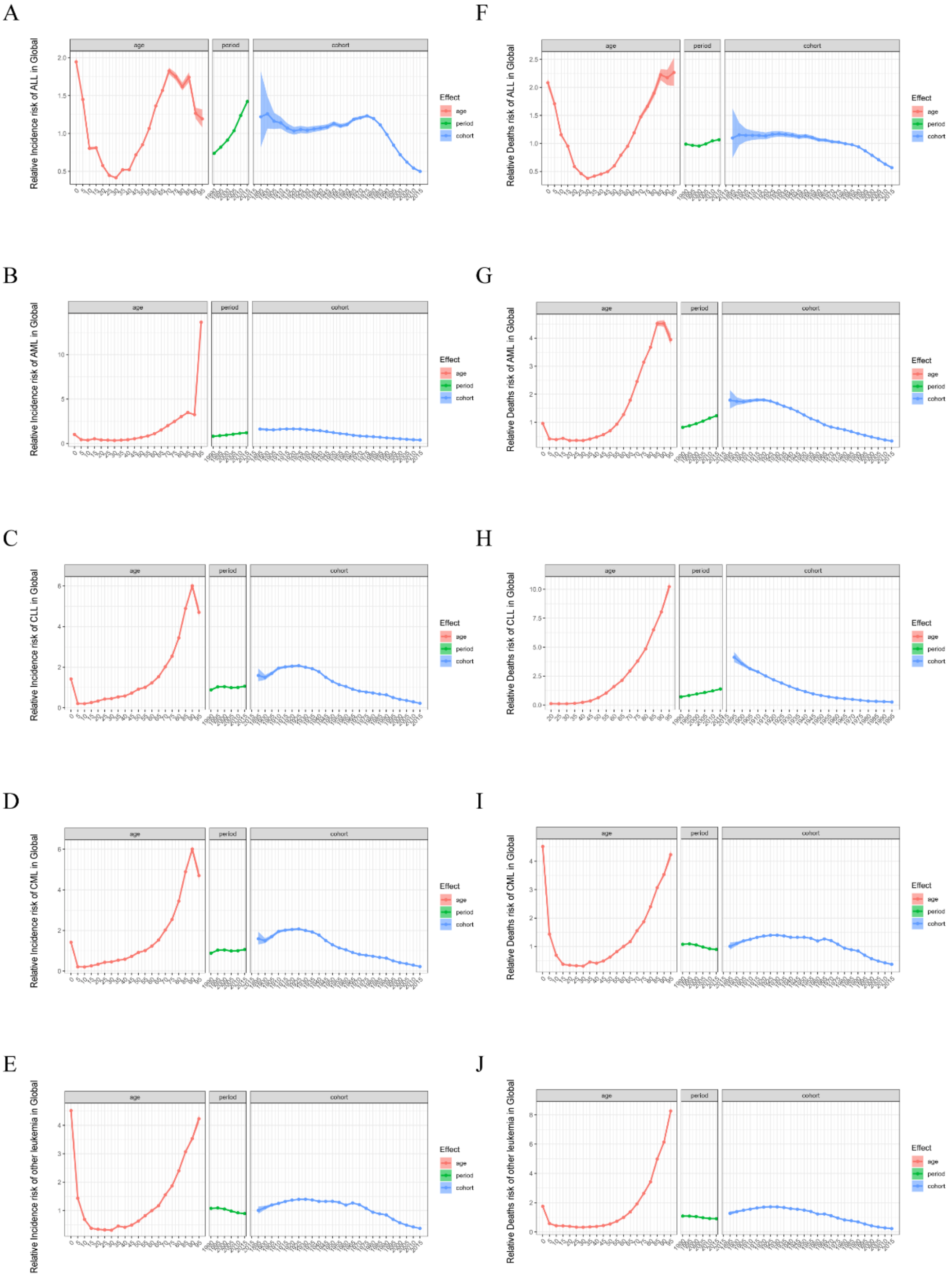

Figure S2 APC-IE analysis of incidence and mortality of global leukemia's subtypes. The age, period and cohort effect coefficients of the APC-IE model of global leukemia's incidence risk from 1990 to 2019, for ALL(A), AML(B), CLL(C), CML(D) and other leukemia(E). The age, period, and cohort effect coefficients of the APC-IE model of global leukemia mortality risk from 1990 to 2019, for ALL(F),

AML(G), CLL(H), CML(I) and other leukemia(J). APC-IE: Age-Period-Cohort model with Intrinsic Estimator.

A

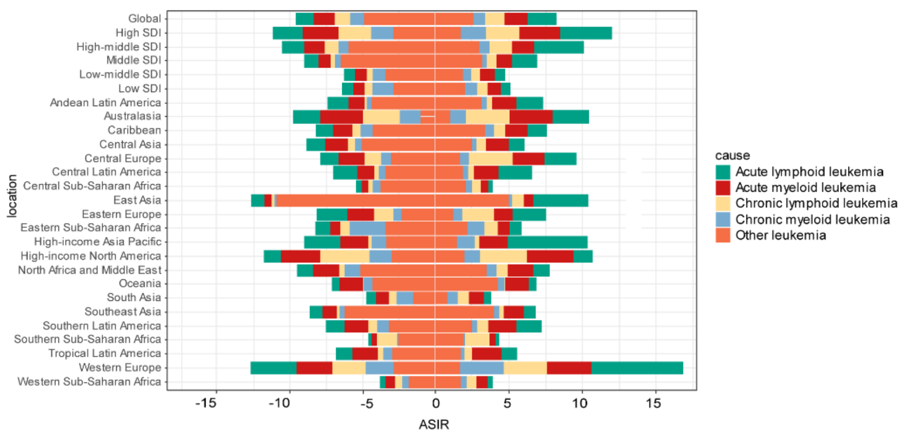

B

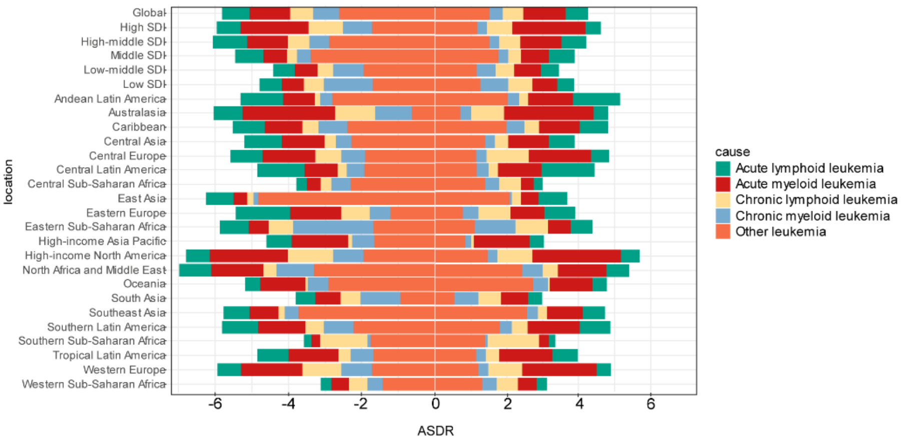

Figure S3 The ASIR and ASDR in leukemia's subtypes by regions. A The ASIR of leukemia's subtypes at a regional level in 1990 and 2019. B The ASDR of leukemia's subtypes at a regional level in 1990 and 2019. Note: ASIR age-standardized incidence rate, ASDR age standardized death rate.

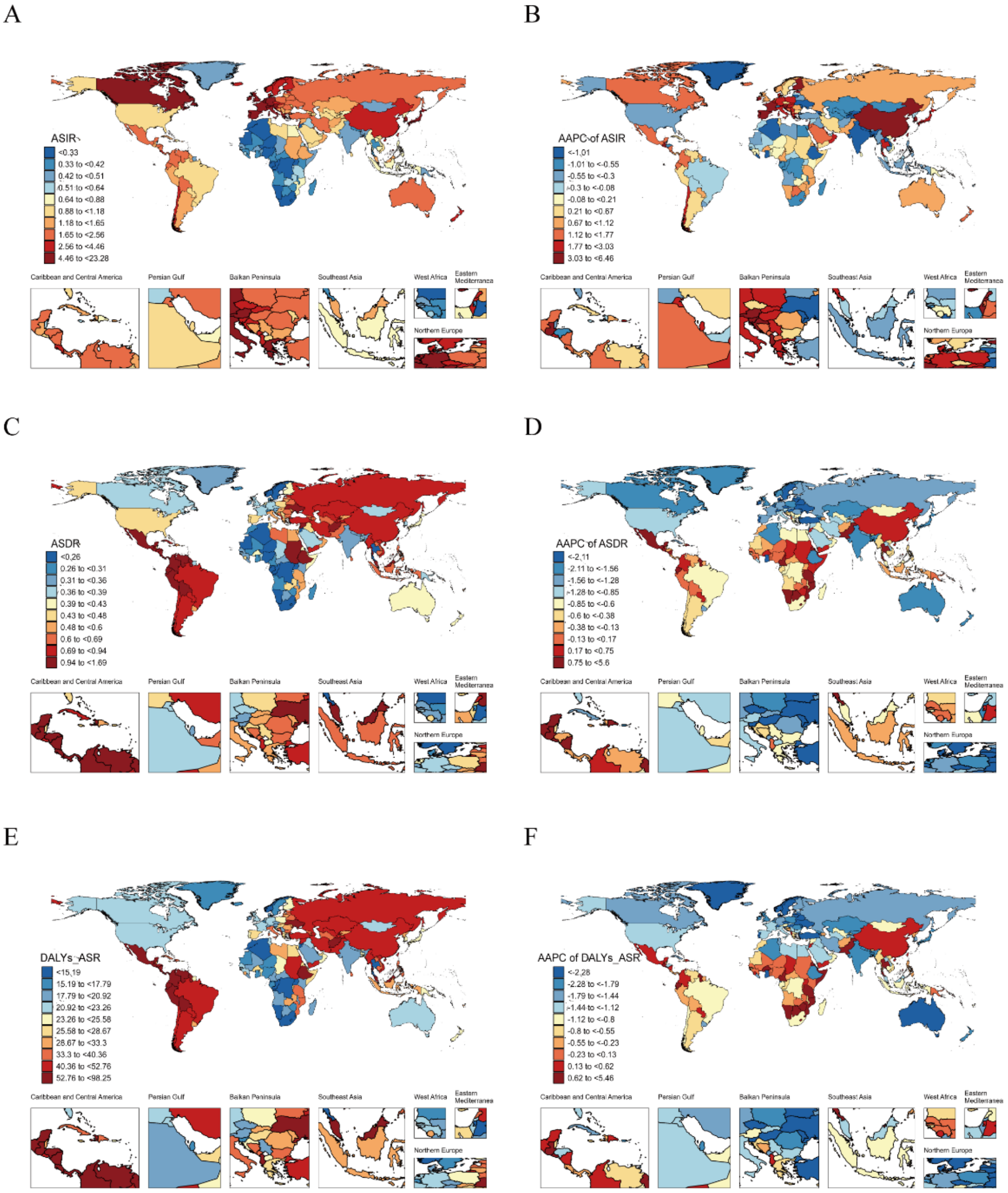

Figure S4 The global trends of ALL by countries and territories. A The ASIR of ALL in 2019. B The AAPC in ASIR of ALL from 1990 to 2019. C The ASDR of ALL in 2019. D The AAPC in ASDR of ALL from 1990 to 2019. E The ASR of DALYs of ALL in 2019. F The AAPC in ASR of DALYs of ALL from 1990 to 2019. Note: ALL acute lymphoblastic leukemia, ASR age-standardized rate, ASIR age-standardized incidence rate, ASDR age-standardized death rate, AAPC average annual percentage changes

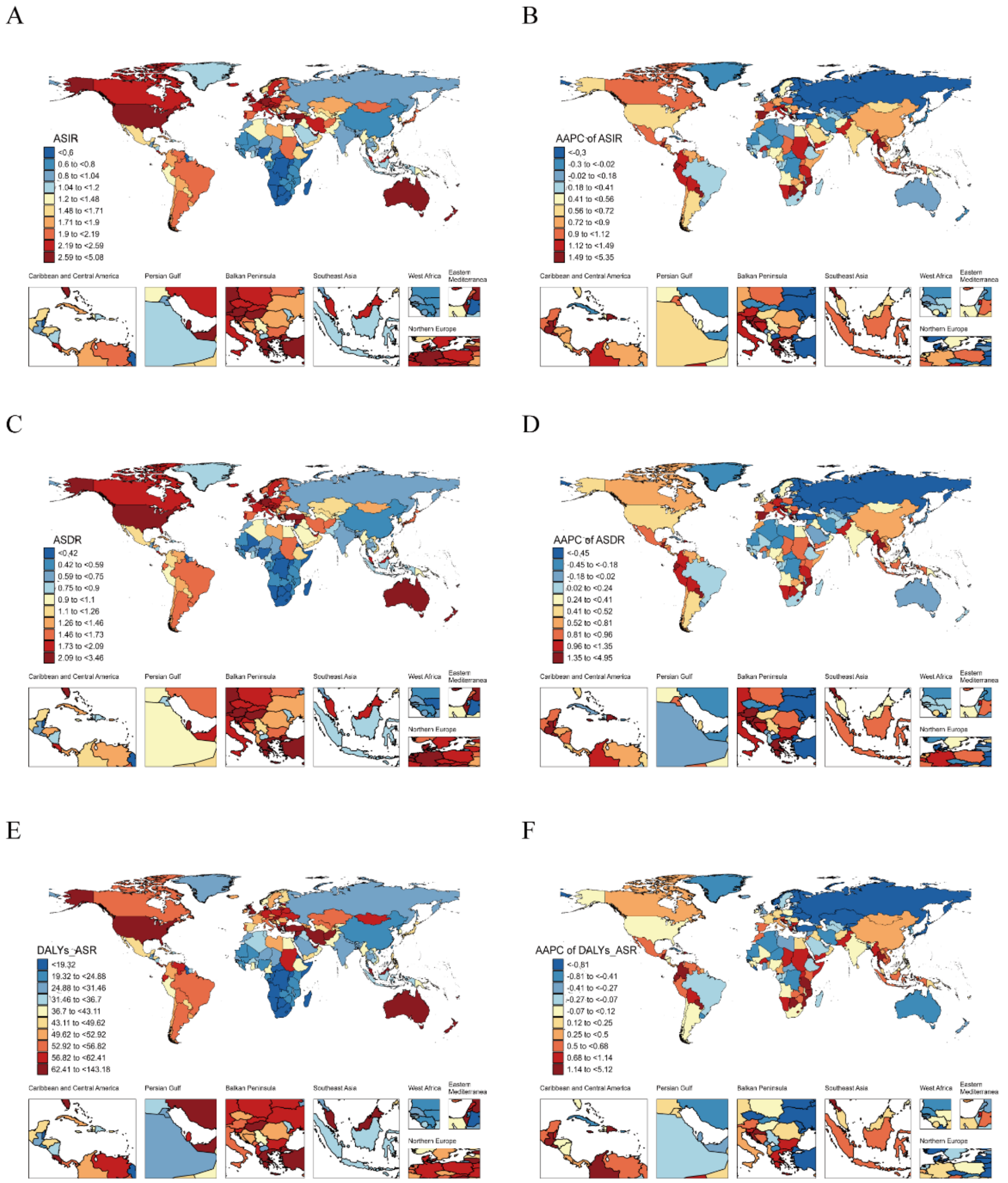

Figure S5 The global trends of AML by countries and territories. A The ASIR of AML in 2019. B The AAPC in ASIR of AML from 1990 to 2019. C The ASDR of AML in 2019. D The AAPC in ASDR of AML from 1990 to 2019. E The ASR of DALYs of AML in 2019. F The AAPC in ASR of DALYs of AML from 1990 to 2019. Note: AML acute myeloid leukemia, ASR age-standardized rate, ASIR age-standardized incidence rate, ASDR age-standardized death rate, AAPC average annual percentage changes.

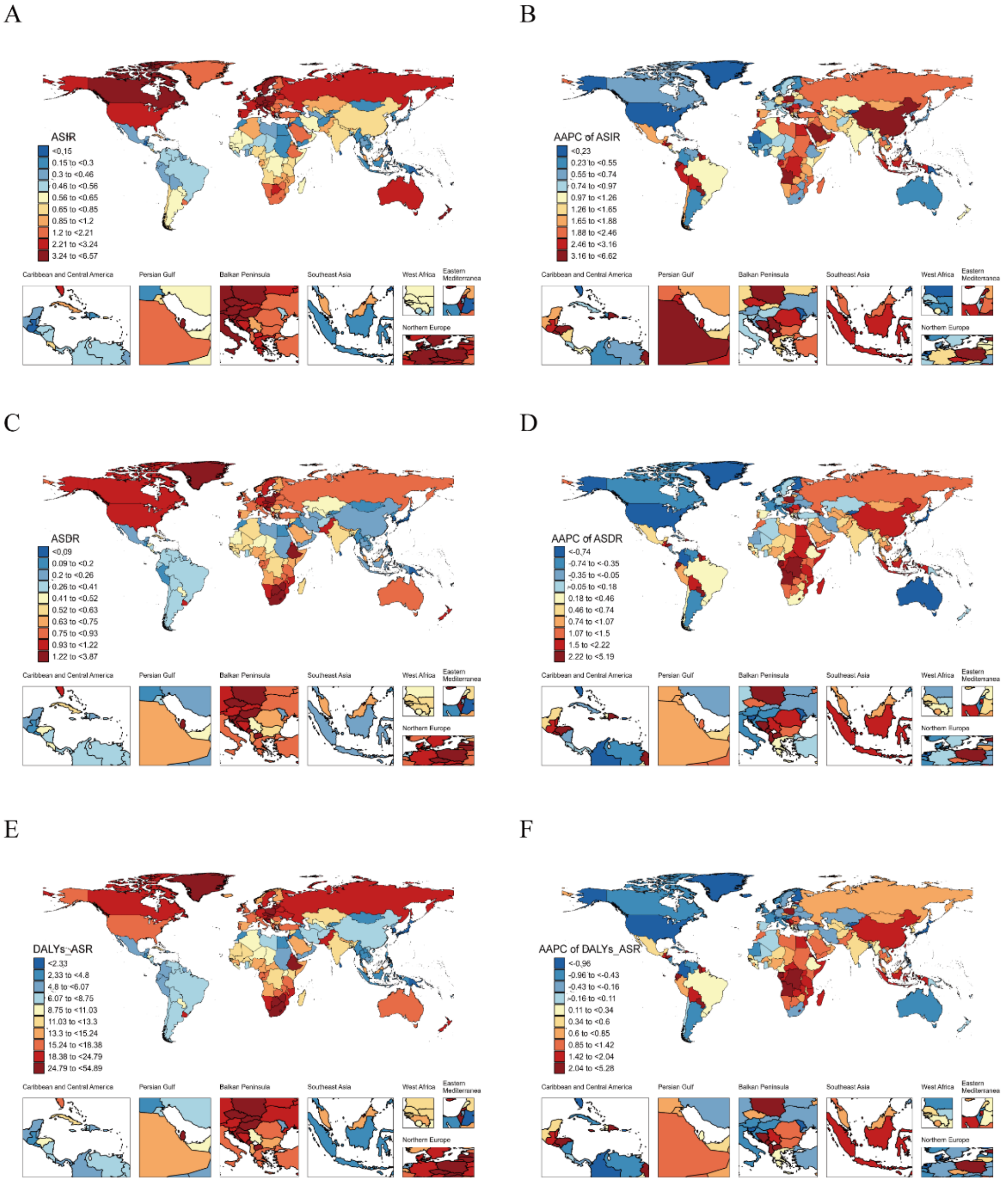

Figure S6 The global trends of CLL by countries and territories. A The ASIR of CLL in 2019. B The AAPC in ASIR of CLL from 1990 to 2019. C The ASDR of CLL in 2019. D The AAPC in ASDR of CLL from 1990 to 2019. E The ASR of DALYs of AML in 2019. F The AAPC in ASR of DALYs of AML from 1990 to 2019. Note: CLL chronic lymphocytic leukemia, ASR age-standardized rate, ASIR age-standardized incidence rate, ASDR age-standardized death rate, AAPC average annual percentage changes.

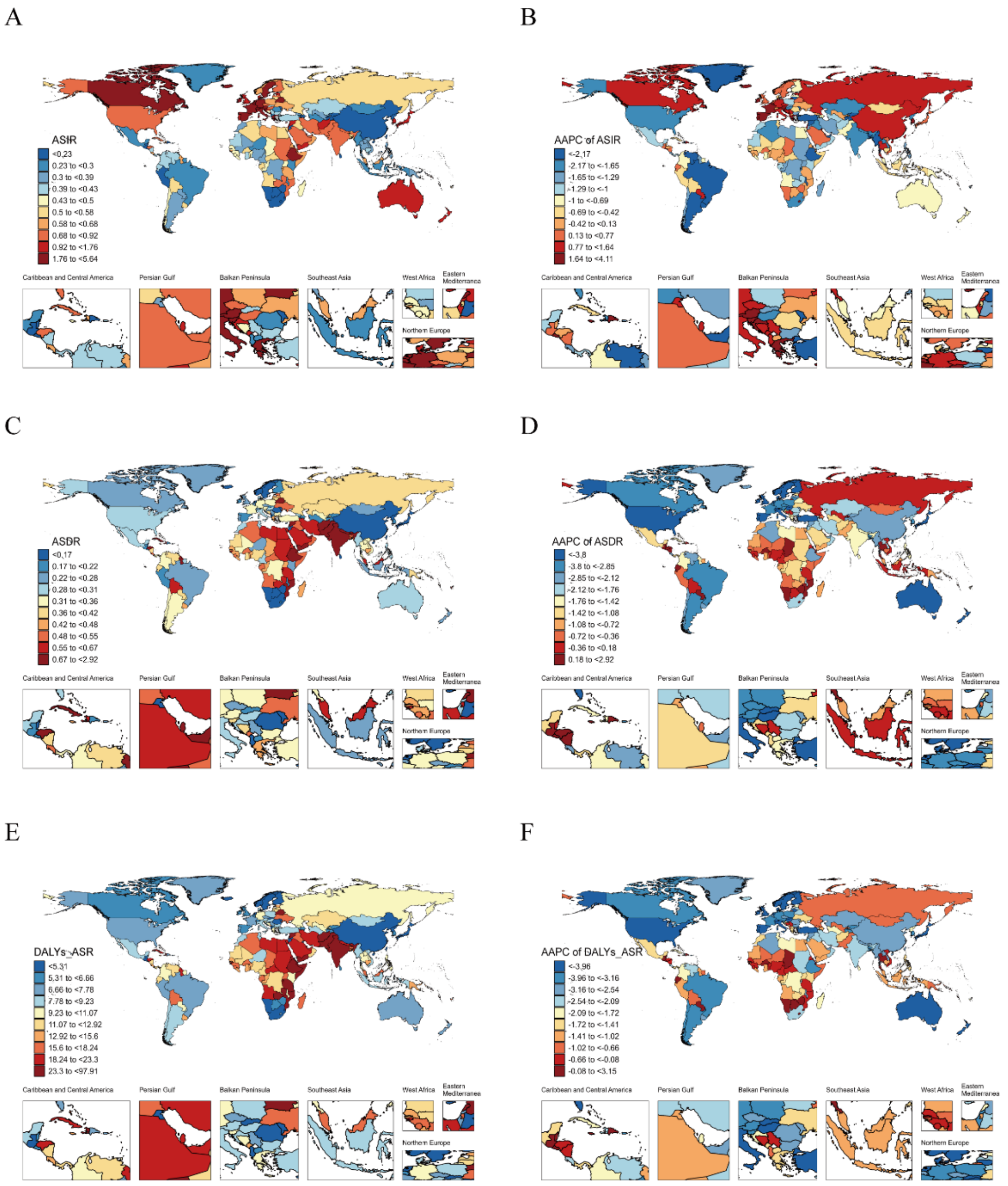

Figure S7 The global trends of CML by countries and territories. A The ASIR of CML in 2019. B The AAPC in ASIR of CML from 1990 to 2019. C The ASDR of CML in 2019. D The AAPC in ASDR of CML from 1990 to 2019. E The ASR of DALYs of CML in 2019. F The AAPC in ASR of DALYs of CML from 1990 to 2019. Note: CML chronic myeloid leukemia, ASR age-standardized rate, ASIR age-standardized incidence rate, ASDR age-standardized death rate, AAPC average annual percentage changes

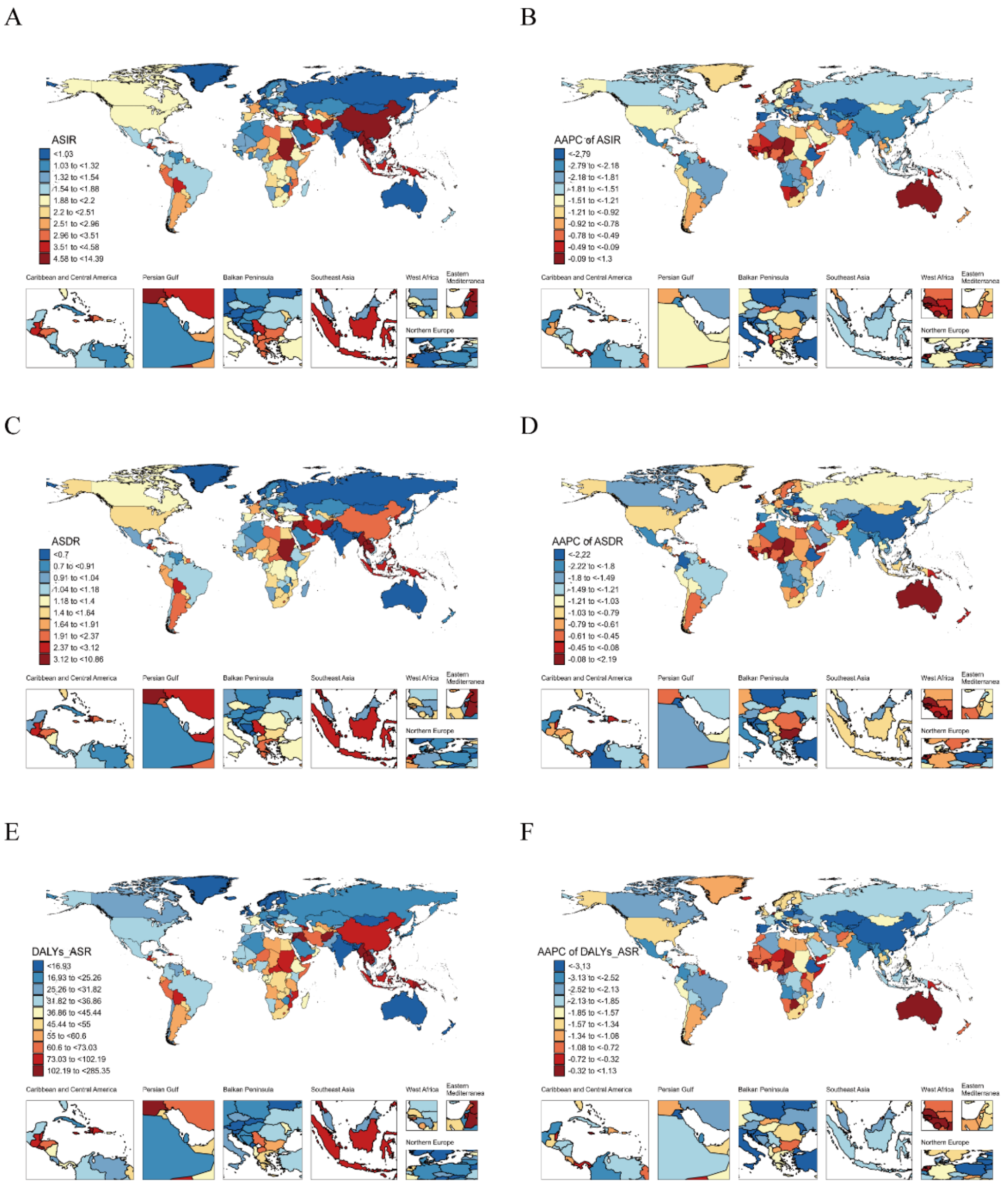

Figure S8 The global trends of other leukemias by countries and territories. A The ASIR of other leukemias in 2019. B The AAPC in ASIR of other leukemias from 1990 to 2019. C The ASDR of other leukemias in 2019. D The AAPC in ASDR of other leukemias from 1990 to 2019. E The ASR of DALYs of other leukemias in 2019. F The AAPC in ASR of DALYs of other leukemias from 1990 to 2019. Note: ASR age-standardized rate, ASIR age-standardized incidence rate, ASDR age-standardized death rate, AAPC average annual percentage changes

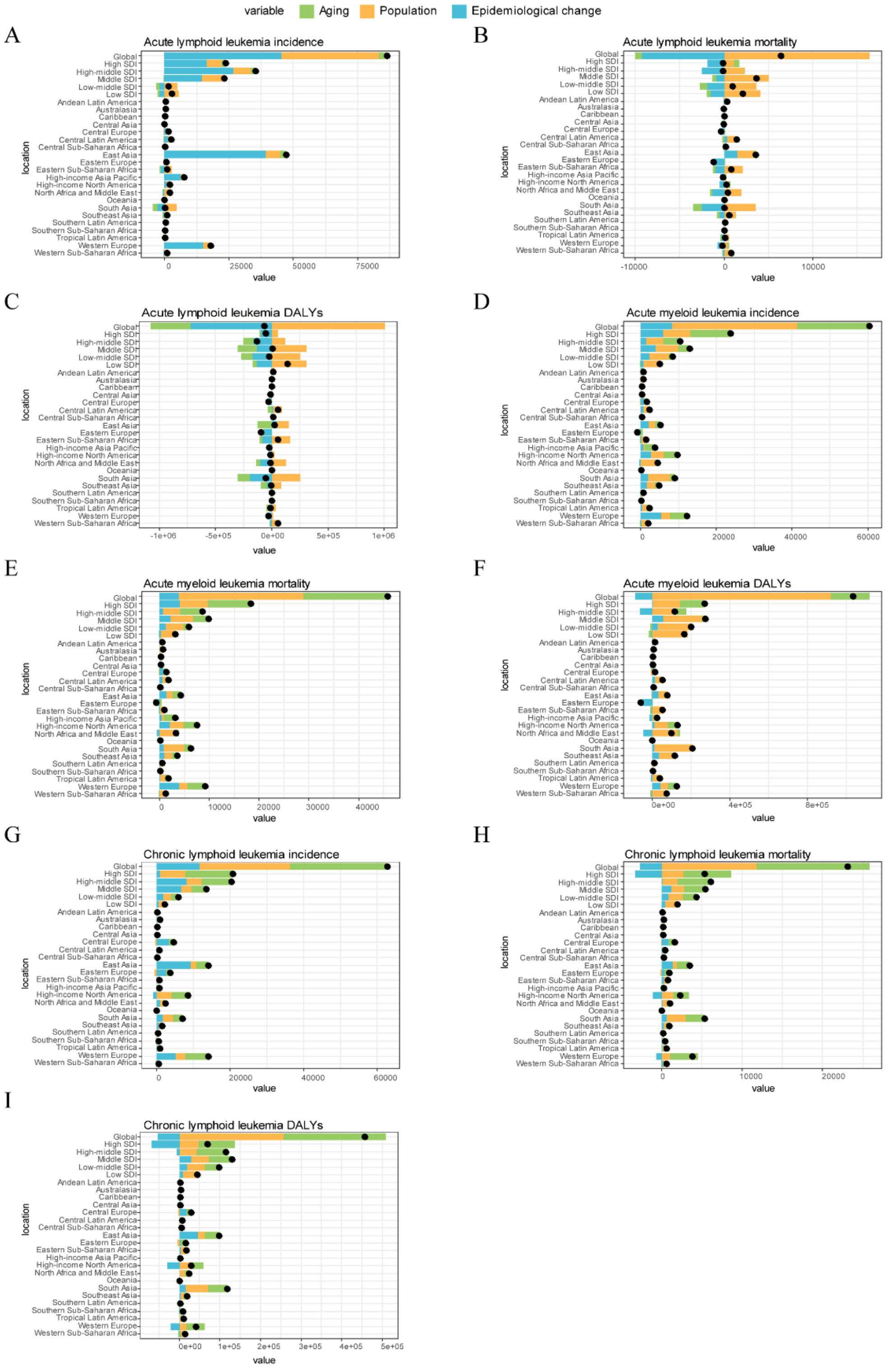

Figure S9 Decomposition analysis of global ALL, AML and CLL trends in incidence, mortality, and DALYs from 1990 to 2019. Decomposition analysis of ALL's (A) incidence, (B) mortality, (C) DALYs, AML's (A) incidence, (B) mortality, (C) DALYs, CLL's (A) incidence, (B) mortality, (C) DALYs according to population-level determinants of population growth, aging, and epidemiological

change of five leukemia subtypes from 1990 to 2019 in global, SDI quintile and 21 regions level. The black dot represents the overall value of change contributed by all seven components. For each component, the magnitude of a positive value indicates a corresponding increase in leukemia cancer incidence attributed to the component; the magnitude of a negative value indicates a corresponding decrease in leukemia incidence attributed to the related component. Note: ALL, Acute Lymphoblastic Leukemia; AML, Acute Myeloid Leukemia; CLL, Chronic Lymphocytic Leukemia; DALYs, Disability-Adjusted Life Years. SDI, Socio-Demographic Index.

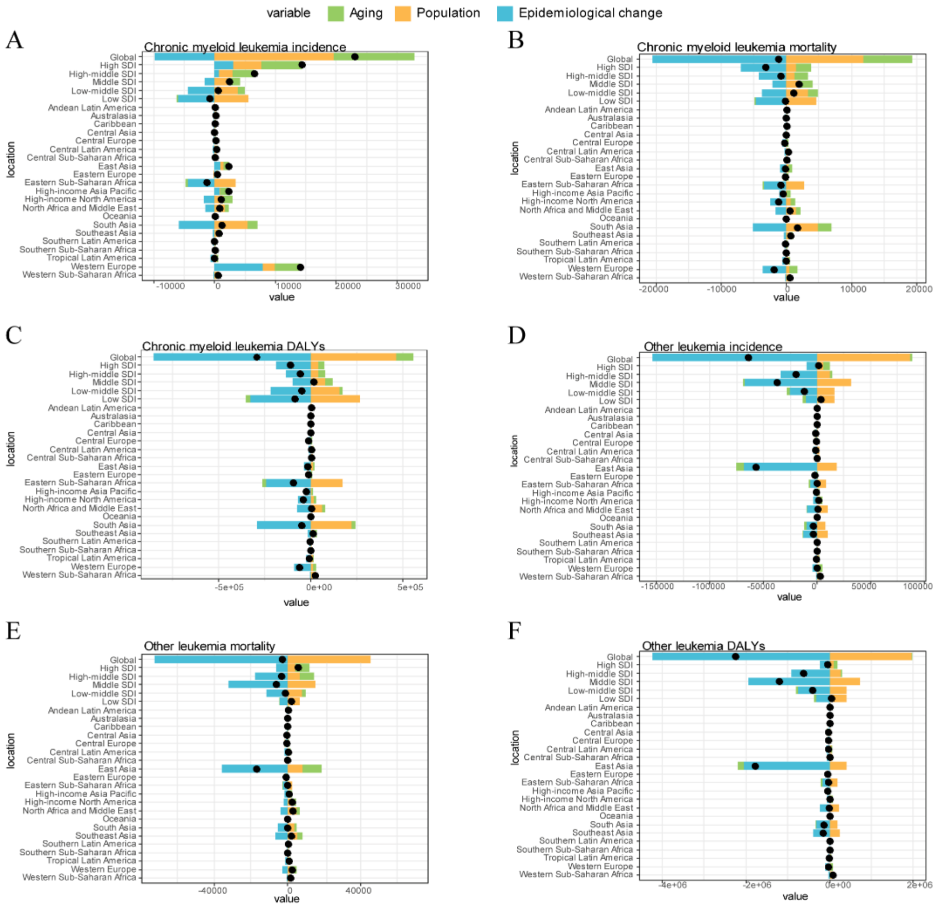

Figure S10 Decomposition analysis of global CML and other leukemia trends in incidence, mortality, and DALYs from 1990 to 2019. Decomposition analysis of CML (A) incidence, (B) mortality, (C) DALYs and other leukemia (A) incidence, (B) mortality, (C) DALYs according to population-level determinants of population growth, aging, and epidemiological change of five leukemia subtypes from 1990 to 2019 in global, SDI quintile and 21 regions level. The black dot represents the overall value of change contributed by all seven components. For each component, the magnitude of a positive value indicates a corresponding increase in leukemia cancer incidence attributed to the component; the magnitude of a negative value indicates a corresponding decrease in leukemia incidence attributed to the related component. Note: CML, Chronic Myeloid Leukemia; DALYs, Disability-Adjusted Life Years. SDI, Socio-Demographic Index.

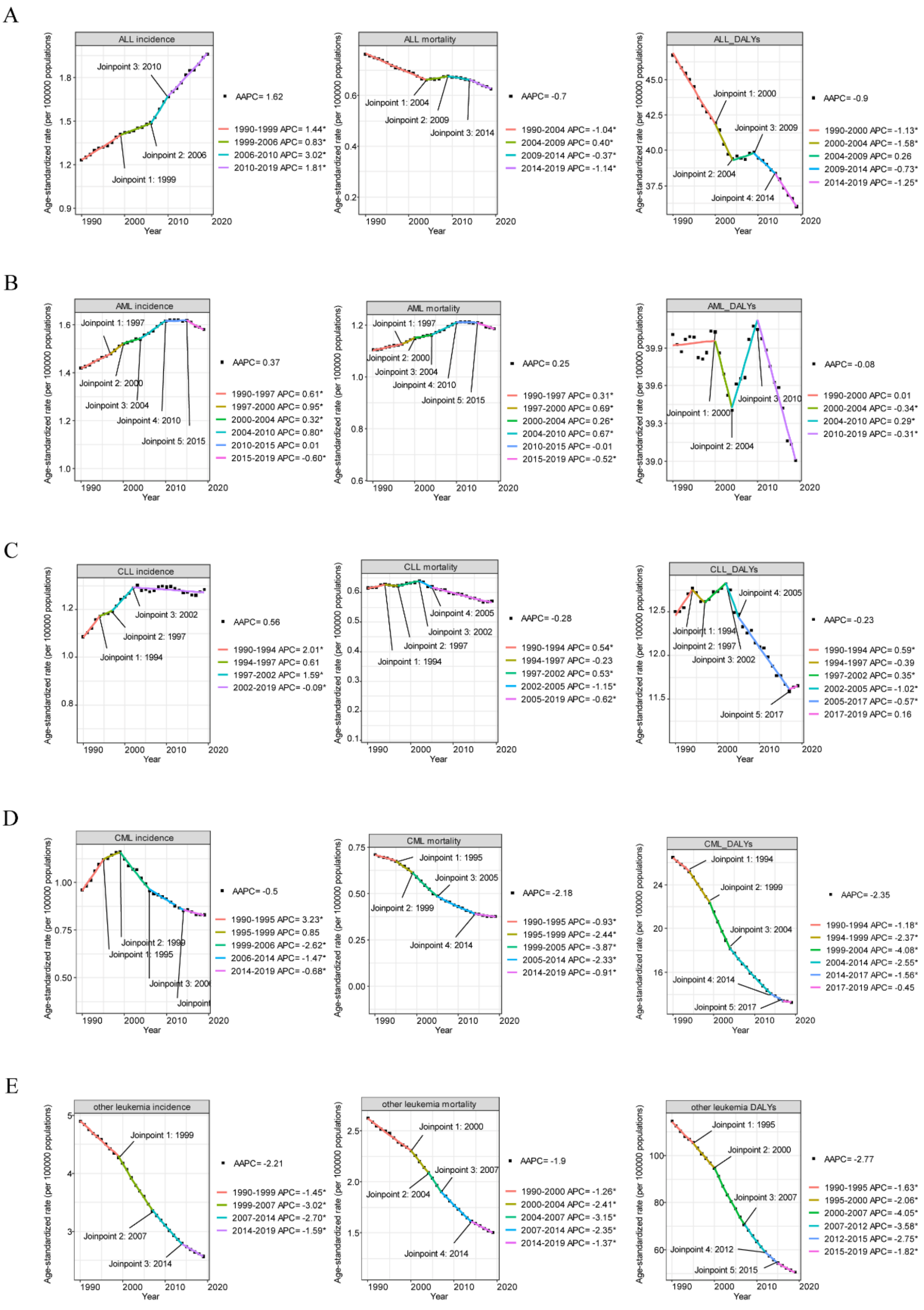

Figure S11 Joinpoint regression analysis of global leukemia's subtypes ASIR, ASDR, and DALYs-ASR from 1990 to 2019. For ALL, joinpoint regression analysis of global (A) ALL, (B) AML, (C) CLL, (D) CML, (E) other leukemia. (*p< 0.05, ** p<0.01, ***p<0.001). Note: ALL, Acute Lymphoblastic Leukemia; AML, Acute Myeloid Leukemia; CLL, Chronic Lymphocytic Leukemia; CML, Chronic Myeloid Leukemia; ASIR, Age-Standardized Incidence Rates; ASDR, Age-

Standardized Death Rates; DALYs-ASR, Disability-Adjusted Life Years Age-Standardized Rate.

A

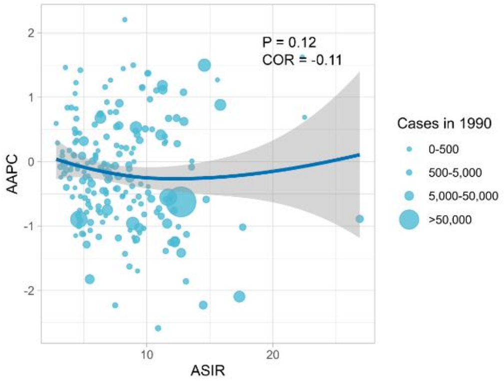

Figure S12 The correlation analyses of AAPCs-ASIR in 1990. A The correlation between ASIR and AAPC of ASIR of 1990 in 195 countries or territories. The size of circle represents the quantity of leukemia patients in one country or territory. Note: ASIR, age-standardized incidence rate; AAPC, average annual percentage change.

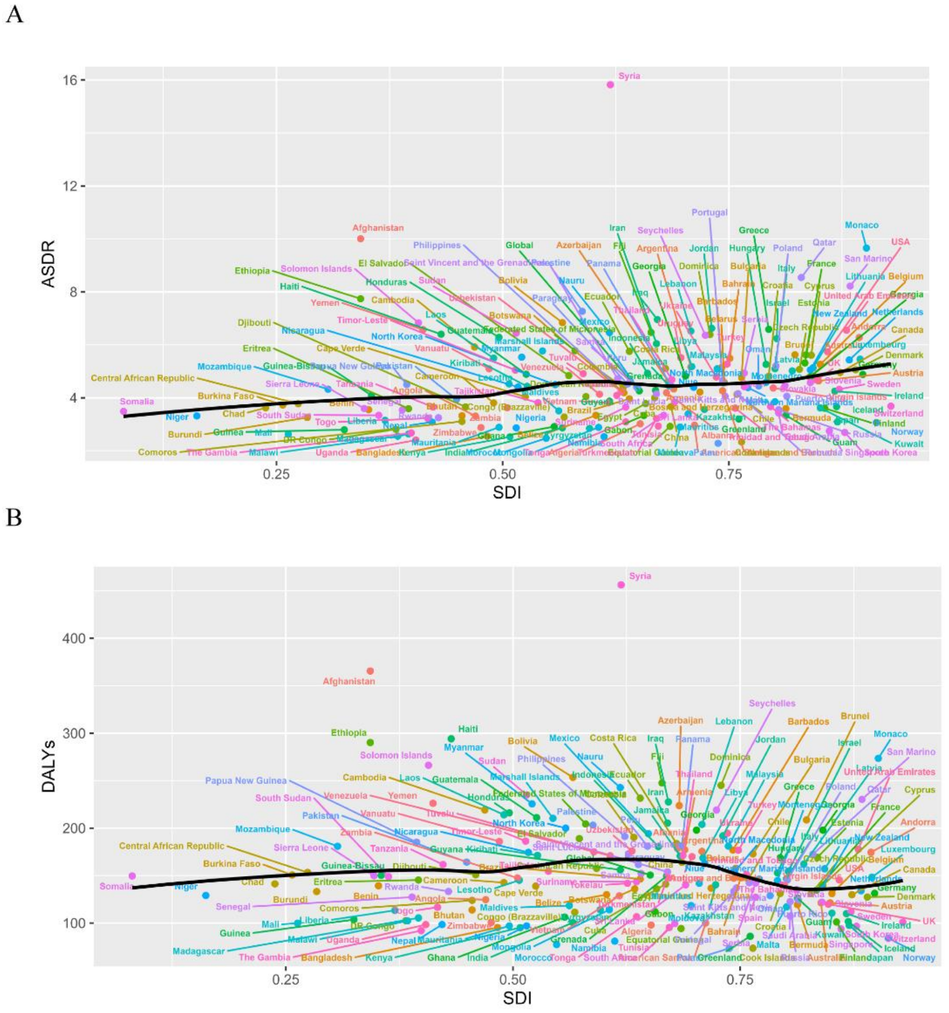

Figure S13 Frontier analysis of SDI, ASDR and age-standardized DALY rate in 2019. A The blue line represents the expected ASDRs and SDIs in 195 countries and territories. B The blue line represents the expected ASR-DALYs and SDIs in 195 countries and territories. Each point shows the observed values for a specified country in 2019. Note: ASR: age-standardized rate; DALYs: Disability-Adjusted Life Years; SDI: Socio-demographic index.

TableS1: The global and GBD regions DALYs cases, prevalence cases, and their AAPCs of leukemia from 1990 to 2019.

location	cause	DALYs					Prevalence				
		1990		2019			1990		2019		
		DALYs cases No*10^3	ASR(per 100,000 population)	DALYs cases No*10^3	ASR(per 100,000 population)	AAPC	Prevalence cases No*10^3	ASR(per 100,000 population)	Prevalence cases No*10^3	ASR(per 100,000 population)	AAPC
Global	Leukemia	12777.4	240.3	11657.5	150.5	-1.62	2423.8	45.6	3222.1	41.1	-0.37
Global	Acute lymphoid leukemia	2730.3	46.7	2661.4	36.0	-0.9	263.3	4.9	975.4	12.3	3.25
Global	Acute myeloid leukemia	2026.0	40.0	3060.1	39.0	-0.08	102.9	2.1	197.3	2.5	0.64
Global	Chronic lymphoid leukemia	492.1	12.5	948.5	11.7	-0.23	208.4	5.3	606.6	7.4	1.1
Global	Chronic myeloid leukemia	1344.4	26.5	1049.7	13.3	-2.35	101.7	2.2	251.6	3.2	1.17
Global	Other leukemia	6184.7	114.5	3938.0	50.6	-2.77	1747.5	31.1	1191.3	15.7	-2.34
High SDI	Leukemia	1704.3	190.8	1832.3	126.5	-1.41	475.3	53.3	949.8	66.8	0.75
High SDI	Acute lymphoid leukemia	276.1	36.7	218.7	22.6	-1.66	105.2	13.2	306.7	27.6	2.68
High SDI	Acute myeloid leukemia	539.2	59.2	811.2	55.3	-0.24	41.9	4.6	78.5	5.1	0.37
High SDI	Chronic lymphoid leukemia	194.5	18.5	263.3	13.6	-1.05	137.2	13.0	279.0	15.0	0.51
High SDI	Chronic myeloid leukemia	224.0	23.7	110.2	7.3	-4.01	50.3	5.2	155.3	9.3	2.01
High SDI	Other leukemia	470.6	52.7	428.9	27.6	-2.2	140.7	17.4	130.2	9.8	-1.96
High-middle SDI	Leukemia	2936.1	262.3	2340.6	150.9	-1.88	585.2	53.3	932.6	58.5	0.34
High-middle SDI	Acute lymphoid leukemia	630.6	56.4	496.7	39.9	-1.19	67.3	6.0	379.8	24.7	5.03
High-middle SDI	Acute myeloid leukemia	521.0	46.0	637.9	39.9	-0.5	21.1	1.9	33.6	2.2	0.48
High-middle SDI	Chronic lymphoid leukemia	140.3	12.9	254.5	12.7	-0.05	51.4	4.6	190.1	9.4	2.49
High-middle SDI	Chronic myeloid leukemia	196.5	17.4	136.3	7.6	-2.82	15.1	1.4	58.4	3.1	2.87
High-middle SDI	Other leukemia	1447.8	129.7	815.2	50.7	-3.23	430.3	39.5	270.8	19.1	-2.46
Low SDI	Leukemia	1347.3	213.1	1647.1	150.4	-1.19	168.4	23.7	211.3	18.0	-0.97
Low SDI	Acute lymphoid leukemia	331.7	41.3	471.8	31.9	-0.92	16.0	2.0	23.7	1.6	-0.8
Low SDI	Acute myeloid leukemia	162.5	27.3	329.6	29.3	0.24	6.7	1.1	17.6	1.5	1.13
Low SDI	Chronic lymphoid leukemia	26.7	11.2	71.3	13.9	0.72	2.5	1.0	9.6	1.8	2.04
Low SDI	Chronic myeloid leukemia	350.6	59.3	262.9	28.1	-2.55	13.5	2.3	10.7	1.2	-2.33
Low SDI	Other leukemia	475.9	73.9	511.4	47.2	-1.53	129.8	17.3	149.7	11.9	-1.3
Low-middle SDI	Leukemia	2371.1	197.6	2201.3	131.5	-1.4	344.3	26.4	287.6	17.4	-1.43

Low-middle SDI	Acute lymphoid leukemia	569.2	39.4	547.7	30.2	-0.94	27.8	1.9	34.2	1.9	-0.04
Low-middle SDI	Acute myeloid leukemia	316.3	27.5	517.8	30.5	0.37	13.0	1.1	27.8	1.6	1.33
Low-middle SDI	Chronic lymphoid leukemia	56.9	9.2	155.6	11.2	0.8	6.1	1.0	30.0	2.1	2.8
Low-middle SDI	Chronic myeloid leukemia	359.7	33.4	310.0	18.9	-1.94	14.0	1.3	13.1	0.8	-1.74
Low-middle SDI	Other leukemia	1069.0	88.2	670.2	40.8	-2.63	283.3	21.1	182.6	11.0	-2.22
Middle SDI	Leukemia	4411.5	254.0	3628.0	152.7	-1.74	849.4	47.1	838.9	35.3	-0.98
Middle SDI	Acute lymphoid leukemia	921.1	47.9	924.7	41.5	-0.49	46.9	2.4	230.7	9.5	4.91
Middle SDI	Acute myeloid leukemia	485.8	28.7	761.4	31.6	0.33	20.1	1.2	39.6	1.7	1.27
Middle SDI	Chronic lymphoid leukemia	73.5	6.3	203.4	8.0	0.89	11.1	0.9	97.6	3.7	4.93
Middle SDI	Chronic myeloid leukemia	212.9	14.0	229.5	9.0	-1.51	8.6	0.6	13.9	0.5	-0.2
Middle SDI	Other leukemia	2718.3	157.2	1508.9	62.6	-3.13	762.7	42.0	457.1	19.9	-2.56
Andean Latin America	Leukemia	94.5	237.2	130.1	208.3	-0.48	13.1	32.1	17.9	28.9	-0.38
Andean Latin America	Acute lymphoid leukemia	34.4	73.3	48.9	76.3	0.11	1.7	3.7	3.5	5.4	1.32
Andean Latin America	Acute myeloid leukemia	13.7	35.9	30.3	48.7	1.08	0.6	1.5	1.6	2.6	2.03
Andean Latin America	Chronic lymphoid leukemia	0.9	3.7	3.0	5.3	1.27	0.1	0.4	0.9	1.6	4.59
Andean Latin America	Chronic myeloid leukemia	3.6	11.5	6.0	9.8	-0.53	0.1	0.5	0.3	0.5	-0.08
Andean Latin America	Other leukemia	41.9	112.8	42.0	68.2	-1.79	10.6	26.1	11.6	18.7	-1.16
Australasia	Leukemia	38.2	177.0	49.8	123.9	-1.2	8.3	37.7	22.1	52.4	1.15
Australasia	Acute lymphoid leukemia	8.3	43.8	6.1	22.4	-2.3	2.1	10.3	6.2	18.5	2.07
Australasia	Acute myeloid leukemia	15.9	72.3	25.9	63.5	-0.42	0.7	3.3	1.4	3.3	0
Australasia	Chronic lymphoid leukemia	4.9	20.7	8.5	16.8	-0.78	3.6	14.8	9.8	20.0	1.04
Australasia	Chronic myeloid leukemia	5.8	25.7	3.0	7.2	-4.32	0.7	3.2	2.4	5.0	1.63
Australasia	Other leukemia	3.2	14.5	6.3	13.9	-0.1	1.3	6.1	2.3	5.7	-0.12
Caribbean	Leukemia	85.9	241.9	93.2	200.5	-0.63	12.9	35.8	15.6	33.5	-0.19
Caribbean	Acute lymphoid leukemia	22.1	56.4	22.4	51.8	-0.29	1.3	3.4	2.5	5.3	1.53
Caribbean	Acute myeloid leukemia	15.6	44.8	21.8	45.7	0.09	0.7	1.8	1.1	2.4	0.93
Caribbean	Chronic lymphoid leukemia	2.3	8.7	4.3	8.4	-0.1	0.6	2.3	1.9	3.7	1.69

Caribbean	Chronic myeloid leukemia	8.9	27.6	8.3	17.0	-1.61	0.4	1.2	0.6	1.2	-0.11
Caribbean	Other leukemia	37.0	104.4	36.4	77.6	-0.99	9.9	27.0	9.5	20.9	-0.83
Central Asia	Leukemia	191.4	262.9	149.5	164.4	-1.6	33.2	42.8	22.8	25.3	-1.78
Central Asia	Acute lymphoid leukemia	49.6	63.5	39.0	41.7	-1.39	2.7	3.4	3.1	3.3	-0.03
Central Asia	Acute myeloid leukemia	41.6	58.4	47.5	50.9	-0.44	1.7	2.3	2.4	2.6	0.43
Central Asia	Chronic lymphoid leukemia	4.9	9.4	7.3	9.2	-0.05	1.0	1.9	2.7	3.3	2.01
Central Asia	Chronic myeloid leukemia	10.7	16.9	8.2	9.2	-2.07	0.4	0.7	0.4	0.5	-1.39
Central Asia	Other leukemia	84.6	114.8	47.6	53.4	-2.6	27.4	34.4	14.2	15.6	-2.69
Central Europe	Leukemia	259.5	201.8	233.1	145.0	-1.15	39.1	30.7	81.7	48.0	1.59
Central Europe	Acute lymphoid leukemia	57.2	50.1	28.3	27.4	-2.1	5.1	4.3	20.4	15.3	4.49
Central Europe	Acute myeloid leukemia	70.6	54.3	86.3	54.9	0.01	2.8	2.2	4.5	2.9	0.98
Central Europe	Chronic lymphoid leukemia	23.7	16.0	53.3	25.1	1.59	8.0	5.3	38.6	18.4	4.41
Central Europe	Chronic myeloid leukemia	27.6	20.1	13.5	7.8	-3.32	1.4	1.0	3.9	2.2	2.76
Central Europe	Other leukemia	80.5	61.3	51.7	29.8	-2.45	21.8	17.9	14.3	9.3	-2.12
Central Latin America	Leukemia	381.9	222.7	470.1	190.6	-0.42	51.3	28.9	61.1	25.0	-0.47
Central Latin America	Acute lymphoid leukemia	159.9	80.3	212.3	86.3	0.29	8.6	4.3	21.1	8.5	2.41
Central Latin America	Acute myeloid leukemia	65.4	40.3	121.0	48.7	0.69	2.8	1.7	6.5	2.6	1.64
Central Latin America	Chronic lymphoid leukemia	4.6	5.1	12.6	5.4	0.14	0.8	0.9	4.6	1.9	2.93
Central Latin America	Chronic myeloid leukemia	19.9	15.8	24.1	9.7	-1.69	0.8	0.7	1.4	0.5	-0.76
Central Latin America	Other leukemia	132.1	81.2	99.9	40.4	-2.39	38.2	21.4	27.5	11.4	-2.12
Central Sub-Saharan Africa	Leukemia	106.9	163.8	132.0	110.3	-1.36	18.9	25.2	20.3	15.3	-1.72
Central Sub-Saharan Africa	Acute lymphoid leukemia	16.8	20.1	27.4	16.0	-0.79	0.8	1.0	1.3	0.8	-0.78
Central Sub-Saharan Africa	Acute myeloid leukemia	10.0	16.3	18.4	15.0	-0.29	0.4	0.6	1.0	0.8	0.51
Central Sub-Saharan Africa	Chronic lymphoid leukemia	1.5	6.5	6.8	12.7	2.35	0.1	0.6	0.8	1.4	3.17
Central Sub-Saharan Africa	Chronic myeloid leukemia	12.5	21.1	13.6	13.4	-1.56	0.5	0.8	0.5	0.6	-1.46
Central Sub-Saharan Africa	Other leukemia	66.1	99.8	65.8	53.2	-2.16	17.0	22.1	16.6	11.8	-2.17

East Asia	Leukemia	4001.4	331.0	2399.0	164.1	-2.38	934.5	78.4	1025.5	69.3	-0.39
East Asia	Acute lymphoid leukemia	561.1	45.4	587.7	47.1	0.06	29.3	2.3	425.0	27.1	8.95
East Asia	Acute myeloid leukemia	229.4	19.1	308.7	20.8	0.34	9.3	0.8	15.6	1.1	1.3
East Asia	Chronic lymphoid leukemia	52.1	4.7	150.2	7.7	1.72	8.6	0.8	114.1	5.7	7.1
East Asia	Chronic myeloid leukemia	63.8	5.5	44.2	2.6	-2.66	2.7	0.2	17.3	1.0	5.12
East Asia	Other leukemia	3095.0	256.3	1308.3	85.9	-3.7	884.6	74.3	453.5	34.4	-2.64
Eastern Europe	Leukemia	549.3	241.1	352.3	140.0	-1.75	73.1	31.4	103.6	37.2	0.58
Eastern Europe	Acute lymphoid leukemia	183.9	90.3	86.5	45.1	-2.38	14.6	7.0	32.6	13.9	2.32
Eastern Europe	Acute myeloid leukemia	147.2	64.2	90.2	37.2	-1.88	5.8	2.6	4.5	1.9	-1.06
Eastern Europe	Chronic lymphoid leukemia	57.2	20.1	73.5	21.6	0.3	18.1	6.3	43.9	12.9	2.29
Eastern Europe	Chronic myeloid leukemia	49.3	19.5	37.6	12.9	-1.28	2.3	0.9	4.9	1.6	2.08
Eastern Europe	Other leukemia	111.7	47.0	64.6	23.2	-2.23	32.2	14.6	17.8	6.9	-2.55
Eastern Sub-Saharan Africa	Leukemia	719.6	282.0	727.9	173.0	-1.66	91.3	30.9	99.8	21.1	-1.32
Eastern Sub-Saharan Africa	Acute lymphoid leukemia	179.8	56.8	234.6	41.2	-1.15	8.7	2.7	11.6	2.0	-1.05
Eastern Sub-Saharan Africa	Acute myeloid leukemia	55.0	23.9	107.4	25.8	0.27	2.3	1.0	5.7	1.3	1.13
Eastern Sub-Saharan Africa	Chronic lymphoid leukemia	9.7	13.5	26.6	17.5	0.9	0.9	1.2	3.4	2.1	2.11
Eastern Sub-Saharan Africa	Chronic myeloid leukemia	235.0	102.0	141.5	40.3	-3.15	9.1	4.0	5.7	1.7	-2.92
Eastern Sub-Saharan Africa	Other leukemia	240.0	85.8	217.8	48.2	-1.99	70.4	22.0	73.3	13.9	-1.6
High-income Asia Pacific	Leukemia	326.7	186.0	267.3	98.2	-2.17	77.2	45.9	156.3	65.1	1.2
High-income Asia Pacific	Acute lymphoid leukemia	65.2	41.0	42.1	23.8	-1.92	29.7	16.9	94.7	44.5	3.41
High-income Asia Pacific	Acute myeloid leukemia	106.4	58.4	132.7	46.1	-0.82	4.3	2.4	7.9	2.5	0.1
High-income Asia Pacific	Chronic lymphoid leukemia	4.5	2.2	7.6	1.9	-0.67	2.8	1.4	8.3	2.2	1.6
High-income Asia Pacific	Chronic myeloid leukemia	40.6	21.3	15.1	5.1	-4.84	5.8	3.0	22.7	7.0	3.01
High-income Asia Pacific	Other leukemia	110.0	63.0	69.8	21.4	-3.69	34.6	22.3	22.6	8.9	-3.17
High-income North America	Leukemia	624.6	200.4	739.0	143.8	-1.13	166.8	52.3	265.8	49.0	-0.2

High-income North America	Acute lymphoid leukemia	89.8	34.4	80.2	22.5	-1.41	15.3	5.8	30.5	8.1	1.31
High-income North America	Acute myeloid leukemia	200.7	64.0	330.1	63.8	0	10.7	3.3	23.0	4.3	0.89
High-income North America	Chronic lymphoid leukemia	88.3	25.0	118.1	18.4	-1.06	74.8	21.2	132.1	20.8	-0.07
High-income North America	Chronic myeloid leukemia	80.3	25.1	36.6	7.0	-4.31	14.9	4.5	28.1	4.8	0.29
High-income North America	Other leukemia	165.5	52.0	173.9	32.1	-1.63	51.0	17.5	52.1	10.9	-1.59
North Africa and Middle East	Leukemia	902.0	270.7	1011.6	183.4	-1.33	140.3	38.0	171.9	32.0	-0.58
North Africa and Middle East	Acute lymphoid leukemia	220.6	52.6	211.0	34.7	-1.39	11.3	2.7	26.9	4.6	1.9
North Africa and Middle East	Acute myeloid leukemia	186.8	57.9	286.9	50.3	-0.5	7.7	2.3	14.8	2.6	0.39
North Africa and Middle East	Chronic lymphoid leukemia	15.4	8.2	39.3	8.8	0.22	2.4	1.2	17.9	3.9	4.02
North Africa and Middle East	Chronic myeloid leukemia	102.9	35.8	104.0	18.8	-2.2	4.2	1.5	6.1	1.1	-0.91
North Africa and Middle East	Other leukemia	376.3	116.2	370.3	70.8	-1.7	114.7	30.2	106.1	19.7	-1.48
Oceania	Leukemia	13.2	210.0	24.6	192.0	-0.33	2.0	28.9	4.0	29.0	-0.01
Oceania	Acute lymphoid leukemia	2.0	25.5	3.7	24.2	-0.19	0.1	1.3	0.2	1.2	-0.18
Oceania	Acute myeloid leukemia	3.3	52.8	6.5	50.4	-0.18	0.1	2.2	0.3	2.6	0.65
Oceania	Chronic lymphoid leukemia	0.1	1.5	0.1	1.4	-0.25	0.0	0.2	0.0	0.2	0.45
Oceania	Chronic myeloid leukemia	1.0	19.6	1.6	14.4	-1.1	0.0	0.8	0.1	0.6	-1.04
Oceania	Other leukemia	6.9	110.6	12.7	101.7	-0.33	1.7	24.5	3.4	24.3	-0.04
South Asia	Leukemia	1768.8	157.6	1847.8	109.2	-1.27	175.2	14.7	182.8	11.1	-0.96
South Asia	Acute lymphoid leukemia	515.7	36.5	461.7	25.0	-1.36	24.8	1.8	24.8	1.3	-1
South Asia	Acute myeloid leukemia	300.7	27.9	507.6	29.6	0.21	12.3	1.1	27.6	1.6	1.23
South Asia	Chronic lymphoid leukemia	63.9	11.4	182.6	13.0	0.55	6.7	1.1	33.4	2.3	2.57
South Asia	Chronic myeloid leukemia	450.9	43.4	400.8	23.7	-2.11	17.5	1.8	17.0	1.0	-1.88
South Asia	Other leukemia	437.6	38.3	295.2	17.9	-2.63	113.9	8.9	80.0	4.9	-2.09
Southeast Asia	Leukemia	1164.7	249.5	1156.8	178.9	-1.11	208.1	42.3	190.0	30.0	-1.18
Southeast Asia	Acute lymphoid leukemia	238.5	43.5	229.7	36.2	-0.6	11.6	2.1	15.4	2.4	0.43

Southeast Asia	Acute myeloid leukemia	138.2	31.6	255.1	38.5	0.7	5.7	1.3	13.3	2.0	1.65
Southeast Asia	Chronic lymphoid leukemia	9.5	3.6	29.0	4.9	1.05	1.3	0.5	8.5	1.4	3.74
Southeast Asia	Chronic myeloid leukemia	49.9	12.7	62.6	9.3	-1.04	2.0	0.5	3.0	0.5	-0.49
Southeast Asia	Other leukemia	728.5	158.1	580.4	90.0	-1.91	187.6	38.0	149.8	23.7	-1.6
Southern Latin America	Leukemia	107.6	218.7	117.9	166.9	-0.93	13.1	26.6	20.9	29.1	0.29
Southern Latin America	Acute lymphoid leukemia	31.3	61.1	30.4	49.0	-0.75	1.8	3.5	5.8	8.6	3.23
Southern Latin America	Acute myeloid leukemia	25.9	52.5	37.1	52.1	-0.04	1.1	2.1	2.0	2.8	0.86
Southern Latin America	Chronic lymphoid leukemia	4.4	9.6	6.8	8.1	-0.61	0.9	2.0	2.9	3.5	2.02
Southern Latin America	Chronic myeloid leukemia	11.7	24.6	6.9	8.9	-3.45	0.5	1.1	0.6	0.8	-1.25
Southern Latin America	Other leukemia	34.3	71.0	36.8	48.8	-1.24	8.8	18.0	9.8	13.5	-0.87
Southern Sub-Saharan Africa	Leukemia	55.8	123.7	71.8	103.0	-0.58	9.2	19.5	12.1	17.6	-0.33
Southern Sub-Saharan Africa	Acute lymphoid leukemia	7.8	12.6	9.8	11.8	-0.3	0.4	0.6	0.5	0.6	0.0
Southern Sub-Saharan Africa	Acute myeloid leukemia	6.0	11.8	10.5	13.2	0.3	0.2	0.5	0.5	0.7	1.2
Southern Sub-Saharan Africa	Chronic lymphoid leukemia	8.4	28.7	17.1	29.7	0.2	1.2	4.0	3.4	5.7	1.3
Southern Sub-Saharan Africa	Chronic myeloid leukemia	1.8	4.0	2.2	2.9	-1.2	0.1	0.2	0.1	0.1	-1.1
Southern Sub-Saharan Africa	Other leukemia	31.7	66.7	32.3	45.3	-1.4	7.3	14.3	7.6	10.5	-1.0
Tropical Latin America	Leukemia	304.6	204.3	322.9	146.7	-1.1	38.7	25.5	41.1	18.8	-1.1
Tropical Latin America	Acute lymphoid leukemia	98.6	56.0	88.0	44.3	-0.8	5.2	3.0	7.1	3.5	0.6
Tropical Latin America	Acute myeloid leukemia	84.1	57.4	123.7	54.4	-0.2	3.5	2.4	6.6	3.0	0.8
Tropical Latin America	Chronic lymphoid leukemia	6.2	6.8	17.0	7.1	0.2	1.0	1.1	5.5	2.2	2.6
Tropical Latin America	Chronic myeloid leukemia	26.1	20.8	17.7	7.3	-3.6	1.1	0.9	0.9	0.4	-2.9
Tropical Latin America	Other leukemia	89.6	63.3	76.6	33.5	-2.2	27.8	18.2	21.0	9.7	-2.2
Western Europe	Leukemia	850.4	190.0	893.1	130.5	-1.3	291.5	67.1	650.5	104.6	1.5
Western Europe	Acute lymphoid leukemia	131.7	39.3	100.5	24.3	-1.7	85.5	23.5	236.5	51.6	2.8
Western Europe	Acute myeloid leukemia	247.4	54.6	376.0	55.5	0.0	27.6	6.1	50.1	7.1	0.5

Western Europe	Chronic lymphoid leukemia	120.2	20.8	161.5	17.1	-0.7	74.5	13.0	170.4	19.5	1.4
Western Europe	Chronic myeloid leukemia	119.0	24.5	56.4	7.5	-4.0	36.2	7.3	133.8	17.2	3.0
Western Europe	Other leukemia	232.1	50.9	198.7	26.2	-2.3	67.8	17.1	59.7	9.2	-2.1
Western Sub-Saharan Africa	Leukemia	230.6	119.3	467.6	113.0	-0.2	26.0	13.3	56.3	13.8	0.1
Western Sub-Saharan Africa	Acute lymphoid leukemia	55.9	20.0	111.1	18.3	-0.3	2.8	1.0	5.5	0.9	-0.3
Western Sub-Saharan Africa	Acute myeloid leukemia	62.2	25.7	136.5	26.5	0.1	2.5	1.0	6.9	1.3	0.9
Western Sub-Saharan Africa	Chronic lymphoid leukemia	9.5	10.8	23.1	12.6	0.6	1.0	1.1	3.3	1.7	1.7
Western Sub-Saharan Africa	Chronic myeloid leukemia	23.2	15.1	45.8	13.5	-0.4	0.9	0.6	1.8	0.6	-0.3
Western Sub-Saharan Africa	Other leukemia	79.9	47.7	151.1	42.0	-0.4	18.9	9.6	38.8	9.3	-0.1

Note: ASR age-standardized rate, AAPC average annual percentage change, DALYs disability-adjusted life years, SDI socio-demographic index.

TableS2: Incidence and Mortality relative sex ratio of leukemia's subtypes in global.

Cause	Age	Incidence						Deaths					
		1990			2019			1990			2019		
		Male/Female Ratio	Upper	Lower	Male/Female Ratio	Upper	Lower	Male/Female Ratio	Upper	Lower	Male/Female Ratio	Upper	Lower
	total	1.31	1.15	1.33	1.34	1.30	1.38	1.48	1.24	1.53	1.53	1.48	1.59
	<5 years	1.17	0.83	1.02	1.23	1.20	1.15	1.22	0.81	1.09	1.56	1.34	1.44
	5-9 years	1.28	1.12	1.27	1.34	1.35	1.31	1.37	1.17	1.36	1.44	1.45	1.39
	10-14 years	1.28	1.10	1.26	1.28	1.28	1.31	1.37	1.12	1.38	1.41	1.38	1.45
	15-19 years	1.54	1.19	1.59	1.53	1.50	1.56	1.75	1.29	1.84	1.71	1.67	1.80
	20-24 years	1.39	1.11	1.47	1.43	1.38	1.48	1.74	1.22	1.92	1.78	1.70	1.91
	25-29 years	1.07	0.83	1.21	1.19	1.12	1.23	1.30	0.87	1.56	1.43	1.31	1.48
	30-34 years	1.22	1.00	1.34	1.26	1.19	1.27	1.54	1.06	1.79	1.68	1.63	1.81
	35-39 years	1.00	0.84	1.15	1.04	0.99	1.06	1.33	0.91	1.52	1.47	1.38	1.55
	40-44 years	0.97	0.87	1.00	1.11	1.06	1.14	1.32	0.97	1.38	1.39	1.33	1.46
Acute lymphoid leukemia	45-49 years	0.98	0.89	1.04	1.10	1.08	1.13	1.27	0.97	1.34	1.37	1.35	1.43
	50-54 years	1.07	1.01	1.10	1.10	1.06	1.15	1.33	1.05	1.39	1.36	1.28	1.46
	55-59 years	1.03	0.96	1.07	1.07	1.03	1.16	1.23	1.02	1.33	1.22	1.19	1.28
	60-64 years	1.10	1.05	1.12	1.12	1.09	1.17	1.30	1.14	1.39	1.30	1.24	1.36
	65-69 years	1.22	1.18	1.26	1.30	1.26	1.35	1.41	1.24	1.44	1.45	1.40	1.59
	70-74 years	1.33	1.29	1.30	1.37	1.35	1.45	1.49	1.41	1.51	1.46	1.43	1.50
	75-79 years	1.39	1.35	1.40	1.52	1.48	1.49	1.45	1.34	1.42	1.52	1.49	1.55
	80-84	1.51	1.49	1.53	1.60	1.53	1.70	1.53	1.47	1.51	1.53	1.49	1.61
	85-89	1.56	1.54	1.57	1.69	1.64	1.74	1.63	1.66	1.67	1.74	1.70	1.78
	90-94	1.66	1.60	1.69	1.44	1.39	1.61	1.69	1.69	1.77	1.54	1.50	1.72
	95+ years	1.65	1.60	1.75	1.61	1.59	1.65	1.72	1.70	1.82	1.73	1.71	1.76
	total	1.44	1.46	1.49	1.60	1.62	1.59	1.52	1.57	1.54	1.71	1.72	1.70
	<5 years	1.26	0.84	1.04	1.41	1.37	1.30	1.33	0.88	1.07	1.65	1.60	1.51
	5-9 years	1.16	0.98	1.13	1.10	1.21	1.05	1.23	1.05	1.21	1.23	1.37	1.18
	10-14 years	1.01	0.92	1.01	0.94	1.06	0.92	1.07	0.98	1.08	1.06	1.19	1.03
	15-19 years	1.04	0.97	1.01	1.00	1.12	0.96	1.10	1.02	1.08	1.12	1.26	1.08
	20-24 years	1.08	0.99	1.05	0.98	1.08	0.96	1.13	1.03	1.12	1.08	1.19	1.06
	25-29 years	1.04	0.98	1.10	1.03	1.11	1.01	1.08	1.00	1.13	1.14	1.24	1.13
	30-34 years	1.01	0.94	1.00	1.05	1.09	1.02	1.05	0.98	1.05	1.16	1.20	1.10
	35-39 years	1.00	0.95	0.99	1.03	1.11	0.98	1.05	0.98	1.02	1.14	1.23	1.08
	40-44 years	1.03	1.00	0.96	1.04	1.11	1.03	1.08	1.04	1.01	1.16	1.24	1.12
Acute myeloid leukemia	45-49 years	0.95	0.94	0.93	0.95	1.01	0.93	0.99	0.97	0.97	1.06	1.10	1.03
	50-54 years	1.12	1.14	1.08	1.16	1.20	1.09	1.18	1.20	1.13	1.28	1.36	1.26
	55-59 years	1.25	1.29	1.21	1.29	1.34	1.23	1.30	1.35	1.24	1.40	1.46	1.31
	60-64 years	1.31	1.35	1.21	1.37	1.42	1.27	1.35	1.38	1.28	1.46	1.51	1.33
	65-69 years	1.43	1.50	1.39	1.46	1.49	1.37	1.46	1.53	1.43	1.54	1.56	1.47
	70-74 years	1.54	1.59	1.47	1.59	1.63	1.48	1.55	1.60	1.49	1.64	1.65	1.54
	75-79 years	1.58	1.63	1.59	1.62	1.62	1.52	1.59	1.63	1.59	1.68	1.69	1.59
	80-84	1.60	1.70	1.65	1.74	1.75	1.75	1.64	1.75	1.68	1.81	1.81	1.82
	85-89	1.58	1.62	1.64	1.76	1.74	1.77	1.66	1.73	1.68	1.89	1.85	1.89
	90-94	1.74	1.96	1.84	2.04	2.01	2.19	1.72	1.94	1.82	2.05	2.02	2.28
	95+ years	1.40	1.40	1.51	1.58	1.62	1.62	1.42	1.42	1.55	1.59	1.62	1.63
	total	1.44	1.44	1.42	1.55	1.62	1.56	1.49	1.48	1.50	1.57	1.64	1.60
	<5 years												
	5-9 years	NA						NA					
	10-14 years												
	15-19 years												
	20-24 years	1.18	1.14	1.05	1.38	1.36	1.28	1.40	1.28	1.27	1.57	1.59	1.43
	25-29 years	0.83	0.71	0.91	1.07	1.02	1.00	0.91	0.77	1.05	1.27	1.24	1.22
	30-34 years	0.87	0.80	0.88	1.09	1.01	1.07	0.99	0.87	1.03	1.32	1.28	1.25
	35-39 years	0.87	0.78	0.88	1.03	0.98	1.02	0.90	0.80	0.92	1.09	1.08	1.10
Chronic	40-44 years	1.14	1.10	1.09	1.03	1.01	1.05	1.08	1.04	1.02	0.96	0.95	0.99

lymphoid leukemia	45-49 years	1.29	1.25	1.25	1.07	1.08	1.10	1.09	1.08	1.07	0.95	0.96	0.96
	50-54 years	1.38	1.35	1.30	1.04	1.07	1.04	1.20	1.16	1.16	0.88	0.88	0.91
	55-59 years	1.42	1.39	1.36	1.18	1.21	1.18	1.27	1.22	1.26	0.99	1.00	1.02
	60-64 years	1.42	1.43	1.35	1.28	1.36	1.26	1.31	1.29	1.27	1.03	1.07	1.06
	65-69 years	1.50	1.53	1.40	1.38	1.48	1.34	1.46	1.45	1.39	1.22	1.25	1.23
	70-74 years	1.48	1.48	1.36	1.45	1.52	1.42	1.48	1.46	1.42	1.29	1.31	1.32
	75-79 years	1.43	1.45	1.36	1.46	1.55	1.43	1.42	1.42	1.36	1.21	1.31	1.22
	80-84	1.47	1.46	1.40	1.53	1.62	1.55	1.52	1.50	1.45	1.43	1.52	1.43
	85-89	1.51	1.50	1.55	1.65	1.70	1.71	1.59	1.58	1.61	1.63	1.71	1.71
	90-94	1.48	1.49	1.53	1.71	1.75	1.77	1.57	1.57	1.61	1.72	1.77	1.77
	95+ years	1.36	1.37	1.37	1.63	1.72	1.67	1.44	1.43	1.49	1.72	1.81	1.76
Chronic myeloid leukemia	total	1.55	1.52	1.59	1.43	1.42	1.53	1.75	1.76	1.79	1.93	1.95	2.02
	<5 years	1.51	1.93	0.94	1.69	2.01	1.38	1.47	1.88	0.90	1.70	1.99	1.43
	5-9 years	0.89	1.00	0.76	0.93	0.97	0.88	0.90	1.01	0.74	1.04	1.08	1.06
	10-14 years	1.05	1.17	0.88	1.10	1.14	1.07	1.08	1.19	0.92	1.26	1.27	1.26
	15-19 years	0.99	1.00	0.98	1.03	1.05	1.01	0.98	1.00	1.00	1.06	1.05	1.08
	20-24 years	1.04	0.97	1.15	1.24	1.27	1.21	1.02	0.95	1.13	1.27	1.28	1.26
	25-29 years	1.13	1.03	1.31	1.45	1.50	1.45	1.10	0.99	1.35	1.64	1.67	1.72
	30-34 years	1.17	1.09	1.31	1.33	1.35	1.33	1.15	1.04	1.32	1.47	1.48	1.47
	35-39 years	1.19	1.09	1.31	1.27	1.27	1.26	1.19	1.06	1.33	1.42	1.44	1.43
	40-44 years	1.22	1.17	1.26	1.24	1.26	1.23	1.28	1.22	1.35	1.38	1.40	1.38
	45-49 years	1.19	1.16	1.19	1.26	1.27	1.25	1.29	1.28	1.30	1.53	1.59	1.54
	50-54 years	1.24	1.21	1.24	1.20	1.19	1.20	1.32	1.30	1.33	1.32	1.32	1.37
	55-59 years	1.27	1.25	1.26	1.34	1.33	1.32	1.43	1.42	1.39	1.66	1.63	1.62
	60-64 years	1.36	1.35	1.32	1.38	1.43	1.39	1.58	1.62	1.52	1.86	1.94	1.78
	65-69 years	1.50	1.48	1.44	1.51	1.49	1.50	1.76	1.81	1.65	2.03	2.03	1.96
	70-74 years	1.62	1.62	1.61	1.49	1.50	1.52	1.83	1.89	1.73	1.98	1.99	1.88
	75-79 years	1.81	1.75	1.81	1.67	1.66	1.75	2.07	2.12	2.01	2.36	2.39	2.35
	80-84	1.77	1.68	1.81	1.54	1.57	1.63	2.06	2.09	2.02	2.29	2.35	2.35
	85-89	1.71	1.64	1.74	1.59	1.59	1.70	1.97	1.97	1.96	2.25	2.30	2.40
	90-94	1.66	1.61	1.79	1.38	1.31	1.59	1.91	1.89	2.00	2.12	2.12	2.34
	95+ years	1.39	1.40	1.45	1.23	1.25	1.32	1.56	1.58	1.70	1.57	1.61	1.68
Other leukemia	total	1.34	1.33	1.44	1.57	1.55	1.63	1.55	1.55	1.62	1.69	1.69	1.74
	<5 years	0.63	0.77	0.68	0.74	0.77	0.82	0.75	0.94	0.84	0.89	0.94	1.05
	5-9 years	1.00	1.12	1.11	1.16	1.08	1.18	1.19	1.34	1.38	1.36	1.26	1.49
	10-14 years	1.09	1.08	1.15	1.16	1.09	1.18	1.20	1.21	1.32	1.27	1.23	1.36
	15-19 years	1.27	1.24	1.41	1.36	1.26	1.44	1.26	1.23	1.44	1.34	1.26	1.42
	20-24 years	1.32	1.27	1.33	1.42	1.35	1.40	1.33	1.28	1.38	1.41	1.37	1.41
	25-29 years	1.04	0.96	1.20	1.39	1.30	1.42	1.07	0.97	1.26	1.40	1.32	1.40
	30-34 years	1.01	0.96	1.08	1.36	1.31	1.33	1.04	0.97	1.13	1.37	1.32	1.34
	35-39 years	0.98	0.94	1.05	1.32	1.23	1.29	1.00	0.96	1.10	1.29	1.24	1.28
	40-44 years	1.07	1.07	1.06	1.26	1.22	1.24	1.08	1.06	1.10	1.21	1.19	1.21
	45-49 years	1.14	1.15	1.16	1.37	1.37	1.37	1.09	1.10	1.11	1.24	1.27	1.20
	50-54 years	1.19	1.20	1.21	1.33	1.34	1.30	1.13	1.11	1.15	1.23	1.23	1.22
	55-59 years	1.23	1.25	1.23	1.32	1.30	1.33	1.22	1.25	1.27	1.29	1.31	1.31
	60-64 years	1.37	1.40	1.37	1.50	1.50	1.50	1.38	1.42	1.42	1.49	1.52	1.49
	65-69 years	1.47	1.50	1.44	1.55	1.51	1.60	1.51	1.54	1.48	1.58	1.57	1.60
	70-74 years	1.50	1.53	1.46	1.56	1.52	1.53	1.54	1.57	1.50	1.60	1.60	1.59
	75-79 years	1.67	1.74	1.63	1.76	1.77	1.80	1.70	1.77	1.67	1.79	1.83	1.80
	80-84	1.68	1.70	1.66	1.74	1.73	1.83	1.72	1.74	1.71	1.78	1.81	1.87
	85-89	1.82	1.83	1.88	1.96	1.93	2.09	1.84	1.85	1.89	1.97	1.95	2.08
	90-94	1.65	1.64	1.71	1.72	1.69	1.83	1.68	1.67	1.74	1.73	1.72	1.83
	95+ years	1.49	1.49	1.57	1.60	1.63	1.65	1.55	1.54	1.65	1.64	1.66	1.64

Table S3 APC-IE Incidence and mortality relative risks of leukemia's subtypes due to age, period, and cohort effects in global.

Factor	Incidence										Mortality									
	ALL		AML		CLL		CML		other leukemia		ALL		AML		CLL		CML		other leukemia	
**Age(years)**	*p*	RR	*p*	RR	*p*	RR	*p*	RR	*p*	RR	*p*	RR	*p*	RR	*p*	RR	*p*	RR	*p*	RR
age_0	0.0	1.9	0.0	1.0	0.0	1.4	0.0	1.4	0.0	4.5	0.0	2.1	0.0	1.0	NA	NA	0.0	4.5	0.0	1.8
age_5	0.0	1.4	0.0	0.4	0.0	0.2	0.0	0.2	0.0	1.4	0.0	1.7	0.0	0.4	NA	NA	0.0	1.4	0.0	0.6
age_10	0.0	0.8	0.0	0.4	0.0	0.2	0.0	0.2	0.0	0.7	0.0	1.2	0.0	0.4	NA	NA	0.0	0.7	0.0	0.4
age_15	0.0	0.8	0.0	0.5	0.0	0.3	0.0	0.3	0.0	0.4	0.0	1.0	0.0	0.4	NA	NA	0.0	0.4	0.0	0.4
age_20	0.0	0.6	0.0	0.4	0.0	0.3	0.0	0.3	0.0	0.3	0.0	0.6	0.0	0.3	0.0	0.1	0.0	0.3	0.0	0.4
age_25	0.0	0.4	0.0	0.4	0.0	0.4	0.0	0.4	0.0	0.3	0.0	0.5	0.0	0.4	0.0	0.1	0.0	0.3	0.0	0.3
age_30	0.0	0.4	0.0	0.3	0.0	0.5	0.0	0.5	0.0	0.3	0.0	0.4	0.0	0.3	0.0	0.1	0.0	0.3	0.0	0.3
age_35	0.0	0.5	0.0	0.4	0.0	0.5	0.0	0.5	0.0	0.5	0.0	0.4	0.0	0.4	0.0	0.1	0.0	0.5	0.0	0.3
age_40	0.0	0.5	0.0	0.4	0.0	0.6	0.0	0.6	0.0	0.4	0.0	0.5	0.0	0.5	0.0	0.2	0.0	0.4	0.0	0.4
age_45	0.0	0.7	0.0	0.5	0.0	0.7	0.0	0.7	0.0	0.5	0.0	0.5	0.0	0.6	0.0	0.4	0.0	0.5	0.0	0.4
age_50	0.0	0.9	0.0	0.7	0.0	0.9	0.0	0.9	0.0	0.6	0.0	0.6	0.0	0.7	0.0	0.6	0.0	0.6	0.0	0.5
age_55	0.0	1.1	0.0	0.9	0.2	1.0	0.2	1.0	0.0	0.8	0.0	0.8	0.0	0.9	0.0	1.0	0.0	0.8	0.0	0.7
age_60	0.0	1.4	0.0	1.1	0.0	1.2	0.0	1.2	0.9	1.0	0.0	0.9	0.0	1.3	0.0	1.6	0.9	1.0	0.2	1.0
age_65	0.0	1.6	0.0	1.5	0.0	1.5	0.0	1.5	0.0	1.2	0.0	1.2	0.0	1.8	0.0	2.1	0.0	1.2	0.0	1.4
age_70	0.0	1.8	0.0	2.0	0.0	2.0	0.0	2.0	0.0	1.6	0.0	1.5	0.0	2.5	0.0	2.9	0.0	1.6	0.0	1.9
age_75	0.0	1.8	0.0	2.5	0.0	2.5	0.0	2.5	0.0	1.9	0.0	1.7	0.0	3.1	0.0	3.8	0.0	1.9	0.0	2.6
age_80	0.0	1.6	0.0	3.0	0.0	3.4	0.0	3.4	0.0	2.4	0.0	1.9	0.0	3.7	0.0	4.8	0.0	2.4	0.0	3.4
age_85	0.0	1.7	0.0	3.5	0.0	4.9	0.0	4.9	0.0	3.1	0.0	2.2	0.0	4.5	0.0	6.5	0.0	3.1	0.0	5.0
age_90	0.0	1.3	0.0	3.2	0.0	6.0	0.0	6.0	0.0	3.5	0.0	2.2	0.0	4.5	0.0	8.0	0.0	3.5	0.0	6.1
age_95	0.0	1.2	0.0	13.7	0.0	4.7	0.0	4.7	0.0	4.2	0.0	2.3	0.0	3.9	0.0	10.2	0.0	4.2	0.0	8.3
**Period**																				
period_1990	0.0	0.7	0.0	0.8	0.0	0.9	0.0	0.9	0.0	1.1	0.0	1.0	0.0	0.8	0.0	0.7	0.0	1.1	0.0	1.1
period_1995	0.0	0.8	0.0	0.9	0.0	1.0	0.0	1.0	0.0	1.1	0.0	1.0	0.0	0.9	0.0	0.8	0.0	1.1	0.0	1.1
period_2000	0.0	0.9	0.0	1.0	0.0	1.0	0.0	1.0	0.0	1.1	0.0	1.0	0.0	1.0	0.0	1.0	0.0	1.1	0.0	1.1
period_2005	0.0	1.0	0.0	1.0	0.1	1.0	0.1	1.0	0.0	1.0	0.0	1.0	0.0	1.0	0.0	1.1	0.0	1.0	0.0	1.0
period_2010	0.0	1.2	0.0	1.1	0.4	1.0	0.4	1.0	0.0	0.9	0.0	1.0	0.0	1.1	0.0	1.2	0.0	0.9	0.0	0.9
period_2015	0.0	1.4	0.0	1.2	0.0	1.1	0.0	1.1	0.0	0.9	0.0	1.1	0.0	1.2	0.0	1.4	0.0	0.9	0.0	0.9
**Cohort**																				
cohort_1895	0.3	1.2	0.0	1.6	0.0	1.6	0.0	1.6	0.9	1.0	0.6	1.1	0.0	1.8	0.0	4.1	0.9	1.0	0.0	1.3
cohort_1900	0.0	1.3	0.0	1.6	0.0	1.5	0.0	1.5	0.0	1.1	0.1	1.2	0.0	1.7	0.0	3.6	0.0	1.1	0.0	1.4
cohort_1905	0.0	1.2	0.0	1.5	0.0	1.7	0.0	1.7	0.0	1.2	0.0	1.1	0.0	1.7	0.0	3.1	0.0	1.2	0.0	1.5
cohort_1910	0.0	1.1	0.0	1.6	0.0	2.0	0.0	2.0	0.0	1.3	0.0	1.1	0.0	1.8	0.0	2.9	0.0	1.3	0.0	1.6
cohort_1915	0.0	1.1	0.0	1.6	0.0	2.0	0.0	2.0	0.0	1.3	0.0	1.1	0.0	1.8	0.0	2.5	0.0	1.3	0.0	1.6
cohort_1920	0.2	1.0	0.0	1.6	0.0	2.0	0.0	2.0	0.0	1.4	0.0	1.1	0.0	1.8	0.0	2.2	0.0	1.4	0.0	1.7
cohort_1925	0.0	1.1	0.0	1.6	0.0	2.1	0.0	2.1	0.0	1.4	0.0	1.2	0.0	1.8	0.0	1.9	0.0	1.4	0.0	1.7
cohort_1930	0.0	1.0	0.0	1.6	0.0	2.0	0.0	2.0	0.0	1.4	0.0	1.2	0.0	1.7	0.0	1.6	0.0	1.4	0.0	1.7
cohort_1935	0.0	1.1	0.0	1.5	0.0	1.9	0.0	1.9	0.0	1.4	0.0	1.2	0.0	1.6	0.0	1.4	0.0	1.4	0.0	1.7
cohort_1940	0.0	1.1	0.0	1.4	0.0	1.8	0.0	1.8	0.0	1.3	0.0	1.1	0.0	1.5	0.0	1.2	0.0	1.3	0.0	1.6
cohort_1945	0.0	1.1	0.0	1.3	0.0	1.5	0.0	1.5	0.0	1.3	0.0	1.1	0.0	1.4	0.0	1.0	0.0	1.3	0.0	1.6
cohort_1950	0.0	1.1	0.0	1.2	0.0	1.3	0.0	1.3	0.0	1.3	0.0	1.1	0.0	1.3	0.0	0.8	0.0	1.3	0.0	1.5
cohort_1955	0.0	1.1	0.0	1.1	0.0	1.1	0.0	1.1	0.0	1.3	0.0	1.1	0.0	1.1	0.0	0.7	0.0	1.3	0.0	1.4
cohort_1960	0.0	1.1	0.0	1.0	0.0	1.1	0.0	1.1	0.0	1.2	0.0	1.1	0.0	1.0	0.0	0.6	0.0	1.2	0.0	1.2
cohort_1965	0.0	1.2	0.0	0.9	0.0	0.9	0.0	0.9	0.0	1.3	0.0	1.1	0.0	0.9	0.0	0.5	0.0	1.3	0.0	1.2
cohort_1970	0.0	1.2	0.0	0.8	0.0	0.8	0.0	0.8	0.0	1.2	0.0	1.0	0.0	0.8	0.0	0.5	0.0	1.2	0.0	1.1
cohort_1975	0.0	1.2	0.0	0.8	0.0	0.8	0.0	0.8	0.0	1.1	0.0	1.0	0.0	0.8	0.0	0.4	0.0	1.1	0.0	1.0
cohort_1980	0.0	1.2	0.0	0.8	0.0	0.7	0.0	0.7	0.0	0.9	0.5	1.0	0.0	0.7	0.0	0.3	0.0	0.9	0.0	0.8
cohort_1985	0.0	1.1	0.0	0.7	0.0	0.7	0.0	0.7	0.0	0.9	0.0	1.0	0.0	0.7	0.0	0.3	0.0	0.9	0.0	0.8
cohort_1990	0.0	1.0	0.0	0.6	0.0	0.6	0.0	0.6	0.0	0.8	0.0	0.9	0.0	0.6	0.0	0.3	0.0	0.8	0.0	0.7
cohort_1995	0.0	0.8	0.0	0.6	0.0	0.5	0.0	0.5	0.0	0.7	0.0	0.9	0.0	0.5	0.0	0.3	0.0	0.7	0.0	0.5
cohort_2000	0.0	0.7	0.0	0.5	0.0	0.4	0.0	0.4	0.0	0.6	0.0	0.8	0.0	0.5	NA	NA	0.0	0.6	0.0	0.4

cohort_2005	0.0	0.6	0.0	0.5	0.0	0.4	0.0	0.4	0.0	0.5	0.0	0.7	0.0	0.4	NA	NA	0.0	0.5	0.0	0.3
cohort_2010	0.0	0.5	0.0	0.4	0.0	0.3	0.0	0.3	0.0	0.4	0.0	0.6	0.0	0.4	NA	NA	0.0	0.4	0.0	0.3
cohort_2015	0.0	0.5	0.0	0.4	0.0	0.2	0.0	0.2	0.0	0.4	0.0	0.6	0.0	0.3	NA	NA	0.0	0.4	0.0	0.2

Note: APC-IE Age-Period-Cohort model with Intrinsic Estimator, ALL acute lymphoblastic leukemia, AML acute myeloid leukemia, CLL chronic lymphocytic leukemia,CML chronic myeloid leukemia.

TableS4: AAPC in incidence, prevalence, mortality, and DALYs of leukemia's subtypes across SDI regions, GBD regions, 204 countries, 1990-2019.

Causes	location	Incidence		Deaths		DALYs	
		p	AAPC_95CI	*p*	AAPC_95CI	*p*	AAPC_95CI
	Global	0	1.62(1.49 to 1.76)	0	-0.7(-0.77 to -0.63)	0	-0.9(-1 to -0.81)
	SDI regions						
	High SDI	0	1.93(1.74 to 2.13)	0	-1.5(-1.59 to -1.4)	0	-1.66(-1.71 to -1.61)
	High-middle SDI	0	2.86(2.66 to 3.07)	0	-0.99(-1.21 to -0.77)	0	-1.19(-1.38 to -0.99)
	Middle SDI	0	2.04(1.72 to 2.36)	0.011	-0.14(-0.25 to -0.03)	0	-0.49(-0.6 to -0.39)
	Low-middle SDI	0	-0.42(-0.62 to -0.22)	0	-0.62(-0.83 to -0.42)	0	-0.94(-1.19 to -0.69)
	Low SDI	0	-0.76(-0.9 to -0.63)	0	-0.76(-0.86 to -0.65)	0	-0.92(-1.07 to -0.78)
	Regions						
	Andean Latin America	0	0.67(0.42 to 0.92)	0.001	0.36(0.14 to 0.57)	0.371	0.11(-0.14 to 0.37)
	Australasia	0.004	0.96(0.3 to 1.62)	0	-2.12(-2.32 to -1.93)	0	-2.3(-2.45 to -2.14)
	Caribbean	0	0.4(0.18 to 0.61)	0.017	-0.26(-0.48 to -0.05)	0	-0.29(-0.44 to -0.13)
	Central Asia	0	-0.79(-1.01 to -0.58)	0	-1.14(-1.37 to -0.91)	0	-1.39(-1.63 to -1.15)
	Central Europe	0	1.85(1.55 to 2.16)	0	-1.9(-2.02 to -1.78)	0	-2.1(-2.22 to -1.98)
	Central Latin America	0	1.25(1.15 to 1.36)	0	0.54(0.33 to 0.76)	0.017	0.29(0.05 to 0.53)
	Central Sub-Saharan Africa	0	-0.71(-0.96 to -0.45)	0	-0.62(-0.81 to -0.43)	0	-0.79(-1.06 to -0.51)
	East Asia	0	5.09(4.54 to 5.64)	0	0.4(0.23 to 0.58)	0.65	0.06(-0.2 to 0.33)
	Eastern Europe	0.61	0.15(-0.41 to 0.71)	0	-1.87(-2.13 to -1.61)	0	-2.38(-2.63 to -2.13)
	Eastern Sub-Saharan Africa	0	-1.02(-1.17 to -0.87)	0	-0.98(-1.12 to -0.83)	0	-1.15(-1.33 to -0.97)
	High-income Asia Pacific	0	2.69(2.08 to 3.31)	0	-1.83(-1.97 to -1.69)	0	-1.92(-2.06 to -1.77)
	High-income North America	0	0.4(0.26 to 0.54)	0	-1.08(-1.22 to -0.93)	0	-1.41(-1.6 to -1.23)
	North Africa and Middle East	0.52	-0.05(-0.21 to 0.11)	0	-1.12(-1.21 to -1.02)	0	-1.39(-1.54 to -1.24)
	Oceania	0.179	-0.21(-0.51 to 0.09)	0.118	-0.27(-0.6 to 0.07)	0.261	-0.19(-0.53 to 0.14)
	South Asia	0	-1.04(-1.45 to -0.62)	0	-1.19(-1.63 to -0.76)	0	-1.36(-1.82 to -0.89)
	Southeast Asia	0.001	-0.14(-0.23 to -0.06)	0	-0.38(-0.49 to -0.27)	0	-0.6(-0.73 to -0.47)
	Southern Latin America	0	1.02(0.69 to 1.36)	0	-0.46(-0.64 to -0.27)	0	-0.75(-0.95 to -0.55)
	Southern Sub-Saharan Africa	0.234	-0.13(-0.35 to 0.09)	0.048	-0.18(-0.36 to 0)	0	-0.31(-0.47 to -0.15)
	Tropical Latin America	0.012	-0.2(-0.36 to -0.04)	0	-0.58(-0.7 to -0.45)	0	-0.82(-0.99 to -0.64)
	Western Europe	0	2.37(2.12 to 2.62)	0	-1.6(-1.74 to -1.46)	0	-1.65(-1.82 to -1.48)
	Western Sub-Saharan Africa	0	-0.28(-0.4 to -0.15)	0	-0.25(-0.38 to -0.13)	0	-0.3(-0.42 to -0.18)
	Countries						
	Afghanistan	0.01	-0.35(-0.62 to -0.08)	0.033	-0.3(-0.58 to -0.02)	0.005	-0.47(-0.8 to -0.14)
	Albania	0	3.13(2.59 to 3.66)	0.969	-0.01(-0.63 to 0.61)	0.498	0.26(-0.49 to 1.01)
	Algeria	0	-1.15(-1.34 to -0.95)	0	-1.78(-2.01 to -1.55)	0	-2.03(-2.3 to -1.77)
	American Samoa	0.043	-0.56(-1.09 to -0.02)	0.015	-0.7(-1.25 to -0.14)	0.042	-0.54(-1.06 to -0.02)
	Andorra	0	2.41(2.2 to 2.62)	0	-1.28(-1.35 to -1.22)	0	-1.22(-1.39 to -1.05)
	Angola	0.122	-0.48(-1.09 to 0.13)	0.183	-0.41(-1 to 0.19)	0.002	-0.52(-0.84 to -0.19)
	Antigua and Barbuda	0.01	0.67(0.16 to 1.18)	0.064	0.35(-0.02 to 0.72)	0.254	0.3(-0.21 to 0.81)
	Argentina	0.002	0.4(0.15 to 0.65)	0.001	-0.4(-0.63 to -0.17)	0	-0.64(-0.74 to -0.53)
	Armenia	0	-1.4(-1.89 to -0.9)	0	-2.67(-3.2 to -2.14)	0	-2.71(-3.08 to -2.34)
	Australia	0	1.01(0.56 to 1.46)	0	-2.11(-2.25 to -1.96)	0	-2.34(-2.59 to -2.09)
	Austria	0	3.41(3.02 to 3.81)	0	-0.96(-1.17 to -0.74)	0	-0.97(-1.21 to -0.73)
	Azerbaijan	0.824	-0.07(-0.64 to 0.51)	0.268	-0.29(-0.8 to 0.22)	0.195	-0.5(-1.26 to 0.26)

Bahamas	0.721	-0.11(-0.73 to 0.51)	0.365	-0.3(-0.95 to 0.35)	0.276	-0.42(-1.16 to 0.33)
Bahrain	0.342	0.36(-0.38 to 1.12)	0	-1.46(-2.03 to -0.89)	0	-1.6(-2.17 to -1.03)
Bangladesh	0	-1.79(-2.14 to -1.45)	0	-1.76(-2.11 to -1.4)	0	-1.94(-2.3 to -1.58)
Barbados	0.848	0.07(-0.6 to 0.74)	0.123	-0.53(-1.2 to 0.14)	0.013	-0.8(-1.42 to -0.17)
Belarus	0.418	-0.31(-1.06 to 0.44)	0	-3.54(-4.21 to -2.86)	0	-3.97(-4.48 to -3.45)
Belgium	0	2.52(2.1 to 2.94)	0	-2.11(-2.3 to -1.93)	0	-2.19(-2.34 to -2.04)
Belize	0.141	-0.36(-0.84 to 0.12)	0.106	-0.39(-0.85 to 0.08)	0.052	-0.61(-1.22 to 0.01)
Benin	0.918	-0.01(-0.19 to 0.18)	0.906	0.01(-0.17 to 0.19)	0.64	0.06(-0.18 to 0.29)
Bermuda	0	3.14(2.84 to 3.45)	0	-1.45(-1.65 to -1.25)	0	-1.22(-1.48 to -0.97)
Bhutan	0.045	-0.53(-1.05 to -0.01)	0.055	-0.47(-0.95 to 0.01)	0.037	-0.67(-1.3 to -0.04)
Bolivia (Plurinational State of)	0.329	-0.1(-0.29 to 0.1)	0.862	0.02(-0.16 to 0.19)	0	-0.45(-0.64 to -0.26)
Bosnia and Herzegovina	0	1.64(0.88 to 2.41)	0.299	-0.44(-1.26 to 0.39)	0.378	-0.53(-1.7 to 0.65)
Botswana	0	1.24(0.92 to 1.57)	0	1.06(0.75 to 1.38)	0	1.37(1.13 to 1.61)
Brazil	0.012	-0.22(-0.39 to -0.05)	0	-0.62(-0.76 to -0.48)	0	-0.86(-1.04 to -0.67)
Brunei Darussalam	0	2.03(1.62 to 2.44)	0	1.16(0.76 to 1.56)	0	0.97(0.54 to 1.41)
Bulgaria	0.004	0.6(0.19 to 1)	0	-0.79(-1.14 to -0.45)	0	-1.13(-1.56 to -0.7)
Burkina Faso	0	0.67(0.45 to 0.89)	0	0.64(0.43 to 0.86)	0	0.84(0.57 to 1.11)
Burundi	0.604	0.09(-0.24 to 0.42)	0.387	0.15(-0.19 to 0.49)	0.971	0.01(-0.33 to 0.34)
Cabo Verde	0.624	0.1(-0.31 to 0.51)	0.73	0.07(-0.35 to 0.5)	0	-0.61(-0.93 to -0.29)
Cambodia	0	-0.55(-0.71 to -0.4)	0	-0.46(-0.59 to -0.32)	0	-0.9(-1.2 to -0.61)
Cameroon	0	1.1(0.58 to 1.62)	0	1.07(0.57 to 1.57)	0	1.31(0.71 to 1.92)
Canada	0	1.77(1.32 to 2.21)	0	-1.58(-1.83 to -1.33)	0	-1.62(-2.02 to -1.21)
Central African Republic	0.004	-0.25(-0.42 to -0.08)	0.003	-0.26(-0.43 to -0.09)	0.01	-0.23(-0.41 to -0.05)
Chad	0.001	0.58(0.24 to 0.92)	0.001	0.59(0.24 to 0.94)	0.003	0.61(0.21 to 1.02)
Chile	0	2.24(1.76 to 2.72)	0.017	-0.54(-0.97 to -0.1)	0.001	-0.85(-1.32 to -0.37)
China	0	5.11(4.57 to 5.65)	0	0.4(0.22 to 0.58)	0.211	0.17(-0.1 to 0.45)
Colombia	0	1.59(1.27 to 1.91)	0.029	0.37(0.04 to 0.7)	0.057	0.35(-0.01 to 0.72)
Comoros	0.528	0.52(-1.08 to 2.15)	0.497	0.54(-1.01 to 2.12)	0.534	0.52(-1.11 to 2.18)
Congo	0.001	-0.75(-1.17 to -0.32)	0	-0.72(-1.12 to -0.32)	0.001	-0.79(-1.25 to -0.34)
Cook Islands	0	-0.29(-0.39 to -0.19)	0	-1.34(-1.41 to -1.26)	0	-1.71(-1.82 to -1.59)
Costa Rica	0.015	1.23(0.24 to 2.24)	0.44	-0.23(-0.82 to 0.36)	0.286	-0.49(-1.39 to 0.41)
Croatia	0	4.04(3.48 to 4.61)	0.056	-0.48(-0.97 to 0.01)	0.006	-0.79(-1.36 to -0.22)
Cuba	0.166	0.26(-0.11 to 0.63)	0	-1.31(-1.67 to -0.94)	0	-1.6(-1.95 to -1.25)
Cyprus	0	5.93(4.78 to 7.09)	0	-1.17(-1.45 to -0.89)	0	-0.76(-1.14 to -0.37)
Czechia	0.244	0.58(-0.39 to 1.55)	0	-4.59(-5 to -4.17)	0	-4.32(-4.71 to -3.94)
C么te d'Ivoire	0.039	-0.25(-0.48 to -0.01)	0.038	-0.24(-0.47 to -0.01)	0.045	-0.2(-0.39 to 0)
Democratic People's Republic of Korea	0.002	-0.2(-0.33 to -0.07)	0.023	-0.12(-0.23 to -0.02)	0	-0.51(-0.7 to -0.33)
Democratic Republic of the Congo	0	-0.81(-1.18 to -0.45)	0	-0.72(-1.07 to -0.36)	0	-0.92(-1.34 to -0.5)
Denmark	0	1.8(1.02 to 2.58)	0	-2.64(-3.17 to -2.12)	0	-2.83(-3.31 to -2.35)
Djibouti	0	1.25(0.78 to 1.73)	0	1.18(0.82 to 1.54)	0	1.28(0.92 to 1.63)
Dominica	0.001	0.65(0.28 to 1.02)	0	0.62(0.36 to 0.88)	0.001	0.6(0.25 to 0.96)
Dominican Republic	0.002	0.75(0.26 to 1.24)	0.002	0.76(0.28 to 1.26)	0.196	0.33(-0.17 to 0.84)
Ecuador	0	1.43(1.09 to 1.78)	0	1.15(0.78 to 1.52)	0	0.99(0.63 to 1.35)
Egypt	0.019	-0.6(-1.1 to -0.1)	0	-0.73(-1.09 to -0.37)	0	-1.17(-1.54 to -0.8)
El Salvador	0	2.73(2.34 to 3.13)	0	2.39(2.01 to 2.76)	0	1.87(1.51 to 2.24)
Equatorial Guinea	0	-1.28(-1.85 to -0.71)	0	-1.21(-1.74 to -0.69)	0	-1.5(-2.13 to -0.87)
Eritrea	0	1.05(0.81 to 1.3)	0	1.01(0.76 to 1.26)	0	1.06(0.83 to 1.3)

	Estonia	0	3.28(2.84 to 3.72)	0	-1.41(-1.76 to -1.06)	0	-1.74(-2.29 to -1.2)
	Eswatini	0	1.11(0.87 to 1.35)	0	1.05(0.81 to 1.28)	0	1.18(1.02 to 1.34)
	Ethiopia	0	-1.63(-1.94 to -1.33)	0	-1.62(-1.92 to -1.32)	0	-1.85(-2.22 to -1.49)
	Fiji	0	-0.97(-1.2 to -0.74)	0	-0.96(-1.14 to -0.79)	0	-0.94(-1.22 to -0.67)
	Finland	0	3.25(2.99 to 3.52)	0	-1.55(-2.01 to -1.1)	0	-1.55(-2.16 to -0.94)
	France	0	3.79(3.62 to 3.97)	0	-1.47(-1.64 to -1.29)	0	-1.35(-1.52 to -1.18)
	Gabon	0.005	-0.63(-1.06 to -0.19)	0.007	-0.59(-1.01 to -0.16)	0.001	-0.8(-1.26 to -0.33)
	Gambia	0.802	0.13(-0.85 to 1.11)	0.675	0.2(-0.75 to 1.16)	0.966	0.02(-0.95 to 1)
	Georgia	0.001	-1.42(-2.25 to -0.59)	0	-1.61(-2.3 to -0.91)	0	-2.01(-2.72 to -1.28)
	Germany	0	2.64(2.28 to 2.99)	0	-1.44(-1.63 to -1.24)	0	-1.47(-1.61 to -1.34)
	Ghana	0	-2.73(-2.98 to -2.49)	0	-2.53(-2.75 to -2.31)	0	-2.97(-3.26 to -2.67)
	Global	0	1.62(1.49 to 1.76)	0	-0.7(-0.77 to -0.63)	0	-0.9(-1 to -0.81)
	Greece	0	2.41(2.1 to 2.72)	0.001	-0.73(-1.15 to -0.32)	0	-0.72(-0.9 to -0.54)
	Greenland	0	-1.63(-2.31 to -0.93)	0	-2.07(-2.4 to -1.74)	0	-2.46(-3.18 to -1.74)
	Grenada	0	-1.47(-1.82 to -1.13)	0	-1.58(-1.93 to -1.23)	0	-1.86(-2.24 to -1.48)
	Guam	0.025	-0.98(-1.82 to -0.13)	0	-1.32(-1.87 to -0.78)	0	-1.29(-1.72 to -0.86)
	Guatemala	0	5.65(5.25 to 6.05)	0	5.6(5.18 to 6.02)	0	5.46(5.1 to 5.82)
	Guinea	0.638	0.09(-0.29 to 0.47)	0.474	0.14(-0.24 to 0.53)	0.624	0.12(-0.35 to 0.59)
	Guinea-Bissau	0.017	-0.71(-1.28 to -0.13)	0.024	-0.62(-1.16 to -0.08)	0	-0.63(-0.9 to -0.37)
	Guyana	0.101	0.81(-0.16 to 1.78)	0.081	0.86(-0.11 to 1.84)	0.262	0.63(-0.47 to 1.73)
	Haiti	0.061	-0.25(-0.52 to 0.01)	0.131	-0.2(-0.45 to 0.06)	0.112	-0.23(-0.51 to 0.05)
	Honduras	0	-0.61(-0.74 to -0.48)	0	-0.34(-0.4 to -0.28)	0	-1.32(-1.51 to -1.13)
	Hungary	0	1.77(1.3 to 2.24)	0	-2.17(-2.64 to -1.71)	0	-2.33(-2.83 to -1.83)
	Iceland	0	1.3(0.67 to 1.94)	0	-1.26(-1.82 to -0.7)	0	-1.49(-2.1 to -0.87)
	India	0	-1.52(-2.2 to -0.83)	0	-1.67(-2.39 to -0.94)	0	-1.93(-2.77 to -1.09)
	Indonesia	0	-0.48(-0.61 to -0.36)	0	-0.34(-0.44 to -0.25)	0	-0.86(-1.01 to -0.72)
	Iran (Islamic Republic of)	0.247	0.27(-0.19 to 0.73)	0	-1.28(-1.48 to -1.09)	0	-1.56(-1.74 to -1.38)
Acute lymphoid leukemia	Iraq	0.001	-0.46(-0.73 to -0.2)	0	-0.76(-0.99 to -0.52)	0	-1.35(-1.62 to -1.07)
	Ireland	0	3.1(2.84 to 3.36)	0	-2.55(-2.85 to -2.24)	0	-2.12(-2.62 to -1.62)
	Israel	0	3.63(3.28 to 3.97)	0	-1.84(-2.07 to -1.61)	0	-1.72(-2.02 to -1.41)
	Italy	0	2.25(1.98 to 2.51)	0	-1.15(-1.46 to -0.84)	0	-1.23(-1.56 to -0.9)
	Jamaica	0.785	-0.12(-0.99 to 0.76)	0.62	-0.21(-1.05 to 0.63)	0.194	-0.59(-1.47 to 0.3)
	Japan	0	2.31(1.91 to 2.72)	0	-1.85(-2.1 to -1.6)	0	-1.87(-2.21 to -1.52)
	Jordan	0	1.66(1.51 to 1.81)	0.007	0.19(0.05 to 0.32)	0.104	0.19(-0.04 to 0.42)
	Kazakhstan	0	-0.81(-1.14 to -0.48)	0	-1.62(-1.88 to -1.36)	0	-2.07(-2.46 to -1.68)
	Kenya	0.581	0.08(-0.2 to 0.36)	0.02	0.31(0.05 to 0.58)	0.452	0.12(-0.2 to 0.44)
	Kiribati	0.008	-0.35(-0.61 to -0.09)	0.004	-0.38(-0.64 to -0.12)	0.052	-0.26(-0.52 to 0)
	Kuwait	0	2.92(1.43 to 4.43)	0.038	-1.2(-2.32 to -0.07)	0.009	-1.44(-2.5 to -0.36)
	Kyrgyzstan	0	-1.26(-1.67 to -0.86)	0	-1.3(-1.69 to -0.9)	0	-1.93(-2.43 to -1.42)
	Lao People's Democratic Republic	0	-1.02(-1.19 to -0.86)	0	-0.93(-1.08 to -0.79)	0	-1.29(-1.49 to -1.08)
	Latvia	0.468	-0.24(-0.88 to 0.41)	0	-2.52(-3.35 to -1.69)	0	-2.92(-3.55 to -2.28)
	Lebanon	0	3.07(2.84 to 3.31)	0	-1.04(-1.11 to -0.96)	0	-1.07(-1.21 to -0.93)
	Lesotho	0	1.7(1.56 to 1.83)	0	1.68(1.55 to 1.81)	0	1.71(1.5 to 1.91)
	Liberia	0	-1.37(-1.75 to -0.98)	0	-1.2(-1.68 to -0.72)	0	-1.47(-1.89 to -1.05)
	Libya	0.015	-0.31(-0.56 to -0.06)	0	-0.71(-0.91 to -0.51)	0	-1.16(-1.57 to -0.75)
	Lithuania	0	-1.64(-2.44 to -0.85)	0	-3.16(-3.92 to -2.38)	0	-3.49(-4.2 to -2.77)
	Luxembourg	0	3.26(2.84 to 3.68)	0	-2.02(-2.2 to -1.84)	0	-1.82(-2.06 to -1.59)
	Madagascar	0	-0.84(-1.23 to -0.45)	0	-0.71(-1.08 to -0.34)	0.001	-0.92(-1.45 to -0.39)
	Malawi	0.001	-0.62(-0.97 to -0.26)	0.002	-0.5(-0.82 to -0.19)	0.001	-0.7(-1.09 to -0.3)
	Malaysia	0.142	-0.26(-0.61 to 0.09)	0.001	-0.76(-1.18 to -0.33)	0	-1.16(-1.6 to -0.71)
	Maldives	0.005	-0.73(-1.23 to -0.22)	0	-2.13(-2.59 to -1.67)	0	-2.43(-2.92 to -1.93)
	Mali	0.37	-0.22(-0.7 to 0.26)	0.562	-0.15(-0.64 to 0.35)	0.484	-0.21(-0.78 to 0.37)
	Malta	0	3.48(2.9 to 4.08)	0	-1.56(-1.69 to -1.44)	0	-0.97(-1.29 to -0.66)

Marshall Islands	0	-0.14(-0.22 to -0.06)	0	-0.18(-0.26 to -0.09)	0.081	-0.06(-0.14 to 0.01)
Mauritania	0	-0.44(-0.55 to -0.34)	0	-0.47(-0.56 to -0.37)	0.001	-0.59(-0.93 to -0.26)
Mauritius	0.023	1.23(0.17 to 2.31)	0.207	0.69(-0.38 to 1.78)	0.271	0.61(-0.48 to 1.72)
Mexico	0	1.27(1.13 to 1.4)	0	0.76(0.52 to 1)	0	0.48(0.27 to 0.7)
Micronesia (Federated States of)	0	-0.43(-0.49 to -0.36)	0	-0.43(-0.49 to -0.37)	0	-0.54(-0.63 to -0.45)
Monaco	0	1.3(1.07 to 1.53)	0	-0.99(-1.09 to -0.89)	0	-1.1(-1.21 to -0.99)
Mongolia	0	-0.82(-1.24 to -0.41)	0	-0.84(-1.24 to -0.44)	0	-1.09(-1.59 to -0.59)
Montenegro	0.001	1.31(0.57 to 2.06)	0	-1.3(-1.89 to -0.7)	0.005	-1.73(-2.92 to -0.52)
Morocco	0.277	-0.3(-0.84 to 0.24)	0.399	-0.21(-0.68 to 0.27)	0.014	-0.74(-1.33 to -0.15)
Mozambique	0.021	0.7(0.1 to 1.3)	0.008	0.76(0.2 to 1.32)	0.037	0.73(0.04 to 1.42)
Myanmar	0	-0.43(-0.57 to -0.29)	0	-0.39(-0.52 to -0.27)	0	-0.68(-0.95 to -0.42)
Namibia	0.002	1.04(0.39 to 1.7)	0.001	0.96(0.39 to 1.54)	0.015	0.95(0.19 to 1.72)
Nauru	0	-0.17(-0.26 to -0.08)	0.002	-0.23(-0.38 to -0.09)	0.001	-0.29(-0.47 to -0.11)
Nepal	0	-1.54(-1.82 to -1.27)	0	-1.35(-1.56 to -1.14)	0	-1.84(-2.35 to -1.33)
Netherlands	0	1.44(0.77 to 2.12)	0	-1.71(-2.37 to -1.05)	0	-1.77(-2.44 to -1.08)
New Zealand	0.001	1.32(0.56 to 2.1)	0	-2.1(-2.45 to -1.75)	0	-2.08(-2.58 to -1.57)
Nicaragua	0	1.39(1.14 to 1.63)	0	1.01(0.73 to 1.29)	0.001	0.4(0.16 to 0.65)
Niger	0.998	0(-0.42 to 0.42)	0.876	0.03(-0.39 to 0.45)	0.624	0.12(-0.36 to 0.6)
Nigeria	0.558	-0.05(-0.21 to 0.11)	0.644	-0.05(-0.27 to 0.16)	0.049	-0.15(-0.3 to 0)
Niue	0.001	0.2(0.08 to 0.31)	0	-0.45(-0.5 to -0.39)	0	-0.4(-0.46 to -0.33)
North Macedonia	0	1.43(0.91 to 1.95)	0	-0.98(-1.48 to -0.48)	0.001	-1.1(-1.74 to -0.45)
Northern Mariana Islands	0	-1.64(-1.88 to -1.39)	0	-2.41(-2.66 to -2.16)	0	-2.4(-2.69 to -2.12)
Norway	0	0.96(0.63 to 1.29)	0	-2.06(-2.34 to -1.79)	0	-2.33(-2.59 to -2.06)
Oman	0	1.86(1.51 to 2.21)	0	-0.6(-0.74 to -0.46)	0	-0.83(-1.09 to -0.57)
Pakistan	0	1.12(0.83 to 1.4)	0	0.96(0.7 to 1.22)	0	1.07(0.67 to 1.47)
Palau	0	0.38(0.28 to 0.47)	0.002	-0.17(-0.28 to -0.06)	0.002	-0.23(-0.37 to -0.08)
Palestine	0	1.16(0.77 to 1.55)	0	0.74(0.38 to 1.1)	0.049	0.47(0 to 0.95)
Panama	0	0.85(0.72 to 0.98)	0.25	-0.18(-0.5 to 0.13)	0.137	-0.35(-0.81 to 0.11)
Papua New Guinea	0.824	0.06(-0.46 to 0.58)	0.633	0.11(-0.35 to 0.58)	0.966	0.01(-0.53 to 0.56)
Paraguay	0	0.9(0.49 to 1.3)	0	0.73(0.33 to 1.13)	0.278	0.23(-0.19 to 0.65)
Peru	0.137	0.37(-0.12 to 0.86)	0.599	-0.12(-0.56 to 0.33)	0.214	-0.34(-0.88 to 0.2)
Philippines	0	-0.33(-0.46 to -0.21)	0	-0.49(-0.7 to -0.29)	0	-0.4(-0.59 to -0.21)
Poland	0	2.22(1.96 to 2.48)	0	-2.08(-2.39 to -1.76)	0	-2.26(-2.66 to -1.85)
Portugal	0	4.41(4.02 to 4.8)	0	-2.31(-2.91 to -1.72)	0	-2.46(-2.86 to -2.06)
Puerto Rico	0	1.86(1.59 to 2.14)	0	-1.66(-1.92 to -1.41)	0	-1.91(-2.18 to -1.64)
Qatar	0	2.4(1.72 to 3.08)	0.015	-0.78(-1.4 to -0.15)	0	-1.79(-2.46 to -1.13)
Republic of Korea	0	6.24(5.8 to 6.67)	0	-1.4(-1.75 to -1.04)	0	-1.63(-1.94 to -1.32)
Republic of Moldova	0	-2.83(-3.36 to -2.3)	0	-3.63(-4.07 to -3.19)	0	-3.92(-4.41 to -3.44)
Romania	0	0.97(0.64 to 1.3)	0	-1.38(-1.61 to -1.16)	0	-2.13(-2.59 to -1.66)
Russian Federation	0.003	1.04(0.36 to 1.73)	0	-1.36(-1.84 to -0.87)	0	-1.72(-2.2 to -1.24)
Rwanda	0.847	-0.05(-0.51 to 0.42)	0.665	0.13(-0.46 to 0.73)	0.97	-0.01(-0.53 to 0.51)
Saint Kitts and Nevis	0.416	0.31(-0.44 to 1.07)	0.159	-0.51(-1.22 to 0.2)	0.099	-0.63(-1.37 to 0.12)
Saint Lucia	0.81	0.05(-0.35 to 0.45)	0.256	-0.24(-0.66 to 0.18)	0.07	-0.37(-0.77 to 0.03)
Saint Vincent and the Grenadines	0.014	0.79(0.16 to 1.42)	0.008	0.79(0.21 to 1.37)	0.258	0.36(-0.26 to 0.99)
Samoa	0	-0.52(-0.63 to -0.41)	0	-0.61(-0.64 to -0.58)	0	-0.75(-0.8 to -0.7)
San Marino	0	2.49(2.33 to 2.64)	0.014	-0.12(-0.22 to -0.02)	0.499	-0.04(-0.16 to 0.08)
Sao Tome and Principe	0	-0.73(-0.99 to -0.46)	0	-0.7(-0.95 to -0.46)	0	-1.03(-1.34 to -0.72)

Saudi Arabia	0	1.54(1.34 to 1.74)	0	-1.24(-1.32 to -1.16)	0	-1.36(-1.47 to -1.25)
Senegal	0.775	-0.2(-1.6 to 1.21)	0.872	-0.1(-1.27 to 1.09)	0.792	-0.19(-1.59 to 1.23)
Serbia	0	2.36(1.84 to 2.89)	0	-0.67(-0.96 to -0.38)	0	-1.35(-1.62 to -1.08)
Seychelles	0.436	0.7(-1.05 to 2.47)	0.51	0.51(-1 to 2.05)	0.489	0.65(-1.19 to 2.53)
Sierra Leone	0.395	0.16(-0.2 to 0.52)	0.342	0.17(-0.18 to 0.51)	0.361	0.18(-0.21 to 0.57)
Singapore	0	3.64(2.74 to 4.55)	0	-2.27(-2.66 to -1.87)	0	-2.24(-2.7 to -1.78)
Slovakia	0	2.62(2.23 to 3.02)	0	-1.3(-1.52 to -1.08)	0	-1.37(-1.6 to -1.14)
Slovenia	0	3.1(2.81 to 3.39)	0	-2.4(-2.95 to -1.84)	0	-2.28(-2.8 to -1.77)
Solomon Islands	0.134	0.16(-0.05 to 0.37)	0.164	0.14(-0.06 to 0.33)	0.094	0.23(-0.04 to 0.51)
Somalia	0.001	0.6(0.26 to 0.95)	0	0.65(0.33 to 0.96)	0	0.67(0.3 to 1.04)
South Africa	0.003	-0.62(-1.03 to -0.2)	0	-0.68(-0.89 to -0.48)	0	-1.02(-1.46 to -0.59)
South Sudan	0.542	0.09(-0.21 to 0.4)	0.504	0.1(-0.19 to 0.39)	0.944	0.01(-0.28 to 0.3)
Spain	0	3.65(3.29 to 4.01)	0	-1.48(-1.68 to -1.28)	0	-1.37(-1.55 to -1.19)
Sri Lanka	0.663	0.13(-0.44 to 0.69)	0	-0.99(-1.52 to -0.45)	0.001	-1.29(-2.04 to -0.54)
Sudan	0.001	0.28(0.12 to 0.45)	0	0.28(0.13 to 0.42)	0.628	0.06(-0.18 to 0.3)
Suriname	0.37	-0.33(-1.04 to 0.39)	0.367	-0.34(-1.07 to 0.4)	0.175	-0.37(-0.91 to 0.17)
Sweden	0.24	0.34(-0.22 to 0.9)	0	-2.58(-3.1 to -2.05)	0	-2.38(-2.69 to -2.06)
Switzerland	0.016	-0.65(-1.17 to -0.12)	0	-3.5(-3.98 to -3.02)	0	-3.66(-4.17 to -3.16)
Syrian Arab Republic	0.334	-0.2(-0.61 to 0.21)	0	-0.86(-1.2 to -0.51)	0	-1.09(-1.54 to -0.62)
Taiwan (Province of China)	0	6.46(5.63 to 7.31)	0.011	0.98(0.22 to 1.74)	0.003	1.21(0.41 to 2.02)
Tajikistan	0	-1.15(-1.58 to -0.73)	0	-1.02(-1.42 to -0.61)	0	-1.57(-2 to -1.15)
Thailand	0	2.32(2 to 2.63)	0	1.07(0.78 to 1.36)	0	1.14(0.85 to 1.43)
Timor-Leste	0.138	-0.22(-0.51 to 0.07)	0.577	-0.08(-0.34 to 0.19)	0.028	-0.54(-1.01 to -0.06)
Togo	0.549	-0.08(-0.33 to 0.18)	0.649	-0.06(-0.31 to 0.2)	0.707	-0.06(-0.36 to 0.24)
Tokelau	0	-0.38(-0.55 to -0.21)	0	-0.47(-0.62 to -0.33)	0	-0.65(-0.86 to -0.43)
Tonga	0.041	0.23(0.01 to 0.45)	0.283	0.17(-0.14 to 0.49)	0.409	0.1(-0.14 to 0.34)
Trinidad and Tobago	0	-1.16(-1.43 to -0.89)	0	-1.36(-1.91 to -0.81)	0	-1.51(-2.08 to -0.94)
Tunisia	0	0.6(0.46 to 0.75)	0	-1.16(-1.31 to -1.01)	0	-1.62(-1.77 to -1.48)
Turkey	0.476	-0.3(-1.13 to 0.53)	0	-2.14(-2.63 to -1.64)	0	-2.46(-3.11 to -1.81)
Turkmenistan	0	-0.85(-1.2 to -0.49)	0	-1(-1.34 to -0.66)	0	-1.35(-1.72 to -0.98)
Tuvalu	0	-0.98(-1.12 to -0.83)	0	-0.96(-1.08 to -0.83)	0	-1.27(-1.48 to -1.06)
Uganda	0.758	-0.08(-0.59 to 0.43)	0.589	-0.13(-0.61 to 0.35)	0.627	0.13(-0.39 to 0.66)
Ukraine	0	-1.32(-1.9 to -0.73)	0	-2.64(-3.41 to -1.86)	0	-3.06(-3.86 to -2.26)
United Arab Emirates	0.367	-0.23(-0.71 to 0.27)	0.056	-0.55(-1.11 to 0.01)	0	-0.83(-1.17 to -0.48)
United Kingdom	0	0.97(0.75 to 1.18)	0	-1.96(-2.2 to -1.72)	0	-2.17(-2.51 to -1.82)
United Republic of Tanzania	0	0.77(0.46 to 1.09)	0	0.8(0.49 to 1.11)	0	0.83(0.49 to 1.18)
United States of America	0.012	-0.34(-0.61 to -0.08)	0	-1.02(-1.15 to -0.88)	0	-1.4(-1.57 to -1.22)
United States Virgin Islands	0	-1.82(-2.45 to -1.18)	0	-2.24(-2.84 to -1.65)	0	-2.54(-3.25 to -1.81)
Uruguay	0.063	-0.37(-0.76 to 0.02)	0	-1.52(-1.8 to -1.23)	0	-1.79(-2.15 to -1.43)
Uzbekistan	0	-0.57(-0.85 to -0.29)	0	-0.79(-1 to -0.59)	0	-1(-1.22 to -0.78)
Vanuatu	0.017	0.49(0.09 to 0.89)	0.003	0.44(0.15 to 0.73)	0.067	0.52(-0.04 to 1.07)
Venezuela (Bolivarian Republic of)	0.36	0.38(-0.43 to 1.18)	0.42	-0.29(-0.99 to 0.42)	0.132	-0.74(-1.7 to 0.22)
Viet Nam	0.804	0.01(-0.1 to 0.12)	0	-0.53(-0.61 to -0.45)	0	-0.65(-0.73 to -0.56)
Yemen	0	0.63(0.41 to 0.84)	0	0.66(0.47 to 0.85)	0	0.56(0.3 to 0.82)
Zambia	0.492	-0.07(-0.26 to 0.12)	0.939	-0.01(-0.19 to 0.18)	0.472	-0.13(-0.5 to 0.23)
Zimbabwe	0	0.68(0.34 to 1.02)	0	0.64(0.32 to 0.97)	0	0.84(0.39 to 1.3)
Global	0	0.37(0.29 to 0.46)	0	0.25(0.18 to 0.32)	0.001	-0.08(-0.12 to -0.03)
SDI regions						

High SDI	0	0.35(0.27 to 0.44)	0	0.35(0.31 to 0.38)	0	-0.24(-0.28 to -0.2)
High-middle SDI	0.002	0.21(0.07 to 0.35)	0.088	0.11(-0.02 to 0.23)	0	-0.5(-0.65 to -0.35)
Middle SDI	0	0.8(0.75 to 0.86)	0	0.63(0.59 to 0.67)	0	0.33(0.24 to 0.43)
Low-middle SDI	0	0.84(0.75 to 0.94)	0	0.61(0.5 to 0.72)	0	0.37(0.27 to 0.47)
Low SDI	0	0.62(0.55 to 0.69)	0	0.44(0.39 to 0.48)	0	0.24(0.16 to 0.31)
Regions						
Andean Latin America	0	1.6(1.49 to 1.71)	0	1.4(1.26 to 1.55)	0	1.08(0.94 to 1.23)
Australasia	0.748	-0.02(-0.16 to 0.11)	0.901	-0.01(-0.11 to 0.1)	0.001	-0.42(-0.66 to -0.18)
Caribbean	0	0.48(0.27 to 0.69)	0.002	0.28(0.1 to 0.45)	0.357	0.09(-0.1 to 0.27)
Central Asia	0.38	0.04(-0.05 to 0.13)	0.083	-0.08(-0.18 to 0.01)	0	-0.44(-0.55 to -0.34)
Central Europe	0	0.75(0.64 to 0.86)	0	0.68(0.56 to 0.79)	0.767	0.01(-0.07 to 0.1)
Central Latin America	0	1.12(0.86 to 1.38)	0	0.98(0.71 to 1.25)	0	0.69(0.44 to 0.94)
Central Sub-Saharan Africa	0.135	0.1(-0.03 to 0.22)	0.425	-0.04(-0.15 to 0.06)	0.001	-0.29(-0.45 to -0.13)
East Asia	0	0.97(0.74 to 1.2)	0	0.77(0.61 to 0.93)	0	0.34(0.22 to 0.47)
Eastern Europe	0	-1.27(-1.88 to -0.67)	0	-1.24(-1.88 to -0.6)	0	-1.88(-2.51 to -1.24)
Eastern Sub-Saharan Africa	0	0.62(0.56 to 0.67)	0	0.49(0.44 to 0.54)	0.003	0.27(0.09 to 0.45)
High-income Asia Pacific	0.115	-0.09(-0.2 to 0.02)	0.336	-0.07(-0.22 to 0.08)	0	-0.82(-0.98 to -0.66)
High-income North America	0	0.59(0.49 to 0.68)	0	0.49(0.36 to 0.62)	0.972	0(-0.16 to 0.15)
North Africa and Middle East	0.908	0(-0.06 to 0.06)	0	-0.15(-0.19 to -0.11)	0	-0.5(-0.56 to -0.44)
Oceania	0.422	0.07(-0.1 to 0.23)	0.054	-0.15(-0.31 to 0)	0.091	-0.18(-0.39 to 0.03)
South Asia	0	0.68(0.53 to 0.83)	0	0.42(0.27 to 0.57)	0.001	0.21(0.08 to 0.34)
Southeast Asia	0	1.03(0.95 to 1.11)	0	0.82(0.75 to 0.88)	0	0.7(0.58 to 0.82)
Southern Latin America	0	0.52(0.42 to 0.61)	0	0.43(0.34 to 0.52)	0.541	-0.04(-0.17 to 0.09)
Southern Sub-Saharan Africa	0	0.58(0.46 to 0.71)	0	0.35(0.23 to 0.47)	0	0.3(0.15 to 0.45)
Tropical Latin America	0	0.37(0.31 to 0.42)	0	0.25(0.17 to 0.32)	0	-0.18(-0.26 to -0.1)
Western Europe	0	0.68(0.53 to 0.83)	0	0.74(0.64 to 0.84)	0.164	0.03(-0.01 to 0.07)
Western Sub-Saharan Africa	0	0.52(0.38 to 0.67)	0	0.37(0.23 to 0.51)	0.156	0.12(-0.04 to 0.28)
Countries						
Afghanistan	0	0.45(0.32 to 0.57)	0	0.2(0.1 to 0.3)	0.02	0.21(0.03 to 0.39)
Albania	0.029	0.43(0.04 to 0.81)	0.603	0.12(-0.33 to 0.56)	0.188	0.28(-0.14 to 0.71)
Algeria	0	-0.26(-0.3 to -0.21)	0	-0.41(-0.48 to -0.35)	0	-0.71(-0.8 to -0.62)
American Samoa	0	-0.76(-0.93 to -0.6)	0	-1.04(-1.22 to -0.85)	0	-0.94(-1.09 to -0.79)
Andorra	0.098	-0.08(-0.17 to 0.01)	0	-0.24(-0.32 to -0.16)	0	-0.42(-0.52 to -0.31)
Angola	0.017	0.48(0.09 to 0.88)	0.034	0.37(0.03 to 0.71)	0.729	0.08(-0.35 to 0.5)
Antigua and Barbuda	0	1.5(1.21 to 1.79)	0	1.38(1.24 to 1.52)	0	1.25(0.89 to 1.61)
Argentina	0	0.62(0.45 to 0.79)	0	0.46(0.34 to 0.58)	0.902	0.01(-0.16 to 0.18)
Armenia	0.019	0.81(0.13 to 1.49)	0.027	0.71(0.08 to 1.35)	0.54	0.24(-0.53 to 1.02)
Australia	0.936	0.01(-0.18 to 0.19)	0.544	-0.06(-0.26 to 0.14)	0	-0.46(-0.61 to -0.31)
Austria	0	1.47(1.24 to 1.71)	0	1.41(1.25 to 1.56)	0	0.67(0.44 to 0.89)
Azerbaijan	0	0.74(0.38 to 1.11)	0	0.54(0.29 to 0.8)	0.13	0.31(-0.09 to 0.72)
Bahamas	0.001	0.59(0.26 to 0.93)	0.101	0.33(-0.07 to 0.74)	0.16	0.36(-0.14 to 0.87)
Bahrain	0.03	-0.39(-0.75 to -0.04)	0.002	-0.55(-0.89 to -0.21)	0	-0.8(-1.02 to -0.57)
Bangladesh	0.767	0.03(-0.16 to 0.21)	0.209	-0.11(-0.29 to 0.06)	0	-0.37(-0.55 to -0.19)
Barbados	0.003	0.67(0.23 to 1.12)	0	0.49(0.22 to 0.76)	0.307	0.17(-0.16 to 0.51)
Belarus	0	-1.29(-1.63 to -0.96)	0	-1.35(-1.7 to -0.99)	0	-2.17(-2.49 to -1.84)
Belgium	0	1.11(0.69 to 1.54)	0	0.97(0.53 to 1.42)	0.015	0.42(0.08 to 0.76)

Belize	0	1.46(1.14 to 1.78)	0	1.25(0.94 to 1.55)	0	1.06(0.9 to 1.23)
Benin	0	0.67(0.4 to 0.95)	0	0.52(0.28 to 0.77)	0.075	0.34(-0.03 to 0.72)
Bermuda	0.002	-0.44(-0.71 to -0.16)	0	-0.64(-0.87 to -0.41)	0	-0.72(-0.93 to -0.5)
Bhutan	0	1.26(1.12 to 1.4)	0	1.12(1.01 to 1.23)	0	0.88(0.65 to 1.11)
Bolivia (Plurinational State of)	0	1.42(1.33 to 1.51)	0	1.28(1.21 to 1.35)	0	0.89(0.84 to 0.94)
Bosnia and Herzegovina	0	1.15(0.88 to 1.41)	0	0.97(0.67 to 1.27)	0	0.68(0.35 to 1.01)
Botswana	0	1.6(1.32 to 1.87)	0	1.31(1.06 to 1.57)	0	1.55(1.39 to 1.72)
Brazil	0	0.34(0.27 to 0.4)	0	0.21(0.14 to 0.29)	0	-0.21(-0.28 to -0.13)
Brunei Darussalam	0.296	0.14(-0.12 to 0.4)	0.492	-0.1(-0.38 to 0.18)	0.227	-0.14(-0.36 to 0.09)
Bulgaria	0	1.11(0.91 to 1.32)	0	0.97(0.76 to 1.17)	0	0.71(0.45 to 0.97)
Burkina Faso	0	1.13(0.96 to 1.3)	0	0.95(0.73 to 1.17)	0	0.9(0.69 to 1.11)
Burundi	0	1(0.75 to 1.25)	0	0.9(0.68 to 1.13)	0	0.65(0.37 to 0.93)
Cabo Verde	0	1.23(0.97 to 1.5)	0	1.18(0.9 to 1.46)	0	0.59(0.37 to 0.8)
Cambodia	0	1(0.93 to 1.07)	0	0.81(0.74 to 0.88)	0	0.56(0.45 to 0.66)
Cameroon	0	0.69(0.34 to 1.05)	0.001	0.46(0.18 to 0.74)	0.088	0.4(-0.06 to 0.87)
Canada	0	0.92(0.79 to 1.05)	0	0.73(0.61 to 0.85)	0	0.28(0.2 to 0.37)
Central African Republic	0.479	-0.04(-0.14 to 0.07)	0	-0.24(-0.34 to -0.15)	0	-0.3(-0.41 to -0.18)
Chad	0	1.05(0.79 to 1.32)	0	0.9(0.67 to 1.14)	0	0.75(0.39 to 1.11)
Chile	0	0.72(0.42 to 1.02)	0	0.66(0.51 to 0.82)	0.596	0.05(-0.15 to 0.25)
China	0	0.81(0.66 to 0.96)	0	0.66(0.55 to 0.77)	0	0.28(0.16 to 0.4)
Colombia	0	1.45(1.21 to 1.69)	0	1.27(1.04 to 1.51)	0	1.15(0.91 to 1.4)
Comoros	0.225	0.82(-0.5 to 2.16)	0.191	0.7(-0.35 to 1.76)	0.493	0.53(-0.97 to 2.05)
Congo	0.072	0.22(-0.02 to 0.45)	0.581	0.05(-0.14 to 0.25)	0.316	-0.15(-0.44 to 0.14)
Cook Islands	0	-0.36(-0.43 to -0.3)	0	-0.52(-0.58 to -0.46)	0	-0.74(-0.78 to -0.7)
Costa Rica	0.063	0.57(-0.03 to 1.18)	0.082	0.43(-0.05 to 0.92)	0.012	0.32(0.07 to 0.58)
Croatia	0	2.68(2.06 to 3.32)	0	2.74(2.07 to 3.41)	0	1.47(0.96 to 1.98)
Cuba	0.757	-0.05(-0.33 to 0.24)	0.399	-0.1(-0.32 to 0.13)	0.014	-0.55(-0.99 to -0.11)
Cyprus	0	0.98(0.81 to 1.15)	0	0.88(0.72 to 1.05)	0	0.38(0.2 to 0.56)
Czechia	0.004	-0.26(-0.43 to -0.08)	0	-0.37(-0.55 to -0.2)	0	-0.85(-1.05 to -0.65)
C么te d'Ivoire	0	0.46(0.26 to 0.65)	0.002	0.29(0.11 to 0.47)	0.116	0.18(-0.04 to 0.4)
Democratic People's Republic of Korea	0.603	-0.02(-0.08 to 0.05)	0	-0.18(-0.24 to -0.11)	0	-0.41(-0.49 to -0.33)
Democratic Republic of the Congo	0.25	-0.12(-0.34 to 0.09)	0.013	-0.26(-0.46 to -0.05)	0	-0.54(-0.78 to -0.29)
Denmark	0	-1.52(-1.93 to -1.11)	0	-1.29(-1.56 to -1.02)	0	-2(-2.49 to -1.51)
Djibouti	0	1.53(1.11 to 1.95)	0	1.36(1.01 to 1.72)	0	1.25(0.94 to 1.56)
Dominica	0	0.97(0.67 to 1.28)	0	0.64(0.47 to 0.81)	0	0.68(0.48 to 0.89)
Dominican Republic	0	2.63(2.19 to 3.07)	0	2.46(2.1 to 2.83)	0	2.11(1.66 to 2.56)
Ecuador	0	2.37(2.12 to 2.63)	0	2.19(1.97 to 2.41)	0	1.84(1.51 to 2.17)
Egypt	0	0.56(0.29 to 0.82)	0	0.41(0.24 to 0.59)	0.247	0.14(-0.1 to 0.39)
El Salvador	0	3.34(2.83 to 3.84)	0	3.22(2.69 to 3.75)	0	2.68(2.38 to 2.98)
Equatorial Guinea	0	1.96(1.72 to 2.21)	0	1.86(1.62 to 2.09)	0	1.45(0.96 to 1.94)
Eritrea	0	2.03(1.69 to 2.38)	0	1.82(1.6 to 2.04)	0	1.78(1.53 to 2.02)
Estonia	0.075	0.53(-0.05 to 1.12)	0.091	0.52(-0.08 to 1.12)	0.255	-0.35(-0.94 to 0.25)
Eswatini	0	1.36(1.23 to 1.49)	0	1.08(0.97 to 1.19)	0	1.3(1.18 to 1.42)
Ethiopia	0	0.28(0.22 to 0.34)	0	0.16(0.12 to 0.21)	0	-0.21(-0.3 to -0.13)
Fiji	0.038	0.36(0.02 to 0.71)	0.379	0.15(-0.18 to 0.48)	0.118	0.21(-0.05 to 0.48)
Finland	0	-0.75(-0.95 to -0.56)	0	-0.59(-0.82 to -0.36)	0	-1.04(-1.12 to -0.95)
France	0	0.95(0.81 to 1.1)	0	0.85(0.68 to 1.03)	0.027	0.13(0.01 to 0.24)

	Gabon	0.026	0.28(0.03 to 0.53)	0.179	0.15(-0.07 to 0.37)	0.683	-0.08(-0.44 to 0.29)
	Gambia	0.272	0.59(-0.46 to 1.66)	0.347	0.48(-0.52 to 1.49)	0.727	0.19(-0.89 to 1.28)
	Georgia	0.424	0.3(-0.43 to 1.03)	0.399	0.3(-0.4 to 1)	0.327	-0.41(-1.22 to 0.41)
	Germany	0	0.77(0.56 to 0.99)	0	1.04(0.77 to 1.31)	0.184	0.17(-0.08 to 0.41)
	Ghana	0.921	0.01(-0.21 to 0.24)	0.4	-0.09(-0.31 to 0.12)	0.006	-0.36(-0.62 to -0.1)
	Global	0	0.37(0.29 to 0.46)	0	0.25(0.18 to 0.32)	0.001	-0.08(-0.12 to -0.03)
	Greece	0	1.69(1.26 to 2.11)	0	1.54(1.25 to 1.84)	0	1.08(0.8 to 1.36)
	Greenland	0.015	-0.29(-0.52 to -0.06)	0	-0.45(-0.6 to -0.29)	0	-0.7(-0.88 to -0.52)
	Grenada	0	0.92(0.67 to 1.16)	0	0.73(0.48 to 0.98)	0	0.5(0.23 to 0.78)
	Guam	0	-1.91(-2.89 to -0.93)	0	-2.28(-3.34 to -1.22)	0	-1.8(-2.6 to -1)
	Guatemala	0	5.35(4.83 to 5.87)	0	4.95(4.4 to 5.5)	0	5.12(4.61 to 5.64)
	Guinea	0.026	0.36(0.04 to 0.67)	0.081	0.24(-0.03 to 0.51)	0.896	0.03(-0.35 to 0.4)
	Guinea-Bissau	0.054	0.17(0 to 0.34)	0.83	0.02(-0.19 to 0.23)	0.001	-0.38(-0.62 to -0.15)
	Guyana	0	0.76(0.5 to 1.01)	0	0.45(0.21 to 0.69)	0.003	0.59(0.21 to 0.99)
	Haiti	0.003	0.41(0.14 to 0.69)	0.057	0.23(-0.01 to 0.47)	0.784	0.04(-0.26 to 0.34)
	Honduras	0	0.73(0.67 to 0.78)	0	0.66(0.49 to 0.82)	0.31	-0.03(-0.09 to 0.03)
	Hungary	0	0.64(0.31 to 0.97)	0.003	0.5(0.17 to 0.82)	0.91	0.02(-0.33 to 0.37)
	Iceland	0.023	0.25(0.03 to 0.47)	0.906	-0.01(-0.22 to 0.19)	0	-0.4(-0.58 to -0.21)
	India	0	0.6(0.42 to 0.78)	0	0.36(0.21 to 0.52)	0.53	0.06(-0.14 to 0.26)
	Indonesia	0	1.07(1.03 to 1.12)	0	0.92(0.87 to 0.98)	0	0.56(0.51 to 0.61)
	Iran (Islamic Republic of)	0.01	-0.23(-0.4 to -0.05)	0	-0.37(-0.5 to -0.23)	0	-0.69(-0.88 to -0.49)
Acute myeloid leukemia	Iraq	0	0.54(0.39 to 0.69)	0	0.39(0.24 to 0.55)	0.009	0.2(0.05 to 0.36)
	Ireland	0	0.53(0.32 to 0.73)	0.002	0.48(0.17 to 0.79)	0.957	0.01(-0.34 to 0.36)
	Israel	0	0.34(0.23 to 0.46)	0	0.21(0.12 to 0.29)	0	-0.27(-0.39 to -0.16)
	Italy	0	1.25(1.08 to 1.42)	0	1.23(1.15 to 1.3)	0	0.41(0.28 to 0.53)
	Jamaica	0	3.25(1.56 to 4.98)	0	3.14(1.46 to 4.85)	0	2.58(1.43 to 3.75)
	Japan	0.098	-0.09(-0.2 to 0.02)	0.29	-0.11(-0.31 to 0.09)	0	-0.82(-1.01 to -0.64)
	Jordan	0	1.03(0.77 to 1.3)	0	0.88(0.7 to 1.07)	0	0.62(0.46 to 0.78)
	Kazakhstan	0	-0.6(-0.91 to -0.28)	0	-0.65(-0.84 to -0.47)	0	-1.16(-1.39 to -0.92)
	Kenya	0	0.76(0.6 to 0.93)	0	0.83(0.75 to 0.91)	0	0.6(0.36 to 0.84)
	Kiribati	0	0.23(0.14 to 0.32)	0.299	-0.05(-0.13 to 0.04)	0.658	-0.03(-0.17 to 0.11)
	Kuwait	0.063	0.93(-0.05 to 1.92)	0.094	0.86(-0.15 to 1.88)	0.57	0.19(-0.47 to 0.87)
	Kyrgyzstan	0.849	0.03(-0.32 to 0.39)	0.936	0.02(-0.42 to 0.46)	0	-0.81(-1.12 to -0.5)
	Lao People's Democratic Republic	0	0.79(0.65 to 0.93)	0	0.6(0.49 to 0.7)	0	0.34(0.28 to 0.41)
	Latvia	0.01	-0.74(-1.3 to -0.18)	0.037	-0.83(-1.6 to -0.05)	0.001	-1.25(-1.95 to -0.54)
	Lebanon	0.825	-0.01(-0.15 to 0.12)	0	-0.27(-0.39 to -0.15)	0	-0.28(-0.38 to -0.19)
	Lesotho	0	1.95(1.88 to 2.02)	0	1.64(1.56 to 1.73)	0	1.87(1.79 to 1.95)
	Liberia	0	-0.8(-1.1 to -0.5)	0	-0.71(-1.1 to -0.33)	0	-1.51(-1.84 to -1.18)
	Libya	0.882	-0.01(-0.14 to 0.12)	0.001	-0.24(-0.38 to -0.1)	0	-0.37(-0.5 to -0.24)
	Lithuania	0.974	-0.01(-0.44 to 0.42)	0.962	0.01(-0.39 to 0.41)	0.001	-0.86(-1.36 to -0.36)
	Luxembourg	0	0.31(0.15 to 0.47)	0	0.27(0.14 to 0.4)	0	-0.16(-0.24 to -0.07)
	Madagascar	0	0.18(0.1 to 0.27)	0.095	0.11(-0.02 to 0.24)	0.021	-0.27(-0.5 to -0.04)
	Malawi	0	0.37(0.19 to 0.55)	0.001	0.22(0.09 to 0.35)	0.653	0.05(-0.18 to 0.29)
	Malaysia	0	0.58(0.45 to 0.7)	0	0.39(0.27 to 0.51)	0	0.16(0.1 to 0.23)
	Maldives	0.122	-0.15(-0.33 to 0.04)	0	-0.33(-0.5 to -0.16)	0	-0.64(-0.84 to -0.43)
	Mali	0.225	0.28(-0.17 to 0.73)	0.441	0.17(-0.26 to 0.6)	0.89	-0.04(-0.58 to 0.5)
	Malta	0	0.49(0.33 to 0.66)	0.168	0.11(-0.05 to 0.27)	0.001	0.21(0.08 to 0.34)
	Marshall Islands	0	0.48(0.42 to 0.55)	0	0.26(0.2 to 0.31)	0	0.26(0.21 to 0.31)
	Mauritania	0.015	-0.28(-0.51 to -0.05)	0	-0.43(-0.64 to -0.23)	0	-0.63(-0.9 to -0.36)
	Mauritius	0	2.25(1.16 to 3.35)	0	2.01(0.91 to 3.12)	0	1.93(0.99 to 2.88)
	Mexico	0	0.97(0.77 to 1.18)	0	0.91(0.67 to 1.14)	0	0.58(0.36 to 0.8)
	Micronesia (Federated States of)	0	0.19(0.13 to 0.24)	0.002	-0.04(-0.06 to -0.01)	0.007	-0.09(-0.15 to -0.02)

Monaco	0	1.11(1.06 to 1.16)	0	0.96(0.9 to 1.01)	0	0.48(0.45 to 0.5)
Mongolia	0	0.58(0.32 to 0.85)	0.008	0.32(0.08 to 0.56)	0.015	0.25(0.05 to 0.45)
Montenegro	0.444	0.13(-0.2 to 0.46)	0.912	-0.02(-0.3 to 0.27)	0.067	-0.38(-0.79 to 0.03)
Morocco	0	0.83(0.68 to 0.99)	0	0.74(0.61 to 0.87)	0	0.51(0.36 to 0.65)
Mozambique	0	1.6(1.32 to 1.87)	0	1.37(1.14 to 1.6)	0	1.39(0.96 to 1.83)
Myanmar	0	1.31(1.24 to 1.39)	0	1.09(1.03 to 1.15)	0	0.86(0.73 to 1)
Namibia	0	1.22(0.86 to 1.59)	0	0.99(0.66 to 1.33)	0	1.08(0.63 to 1.53)
Nauru	0	-0.15(-0.19 to -0.11)	0	-0.38(-0.44 to -0.31)	0	-0.4(-0.48 to -0.32)
Nepal	0	0.52(0.42 to 0.63)	0	0.45(0.38 to 0.53)	0.543	-0.05(-0.2 to 0.11)
Netherlands	0.002	0.59(0.21 to 0.97)	0	0.57(0.29 to 0.85)	0.678	-0.11(-0.62 to 0.4)
New Zealand	0.21	-0.11(-0.28 to 0.06)	0.817	0.03(-0.2 to 0.25)	0.005	-0.28(-0.48 to -0.09)
Nicaragua	0.013	0.83(0.18 to 1.49)	0.019	0.83(0.14 to 1.53)	0.456	0.09(-0.15 to 0.33)
Niger	0.486	-0.12(-0.45 to 0.21)	0.12	-0.23(-0.52 to 0.06)	0.002	-0.57(-0.93 to -0.2)
Nigeria	0	0.56(0.28 to 0.83)	0.008	0.36(0.09 to 0.64)	0.31	0.12(-0.11 to 0.35)
Niue	0.003	0.08(0.03 to 0.13)	0	-0.14(-0.2 to -0.09)	0	-0.17(-0.23 to -0.11)
North Macedonia	0	-0.3(-0.47 to -0.14)	0	-0.53(-0.68 to -0.37)	0	-0.55(-0.74 to -0.36)
Northern Mariana Islands	0	-2.37(-2.49 to -2.25)	0	-2.66(-2.78 to -2.54)	0	-2.4(-2.55 to -2.25)
Norway	0	-0.71(-0.95 to -0.47)	0.075	-0.27(-0.58 to 0.03)	0	-1.07(-1.26 to -0.88)
Oman	0	0.51(0.39 to 0.64)	0	0.33(0.21 to 0.45)	0.024	0.16(0.02 to 0.3)
Pakistan	0	1.35(1.22 to 1.48)	0	0.96(0.82 to 1.1)	0	1.13(0.95 to 1.3)
Palau	0	0.18(0.13 to 0.23)	0.252	-0.04(-0.1 to 0.03)	0.072	-0.07(-0.15 to 0.01)
Palestine	0	1.24(1.14 to 1.34)	0	1.12(0.92 to 1.32)	0	0.85(0.72 to 0.98)
Panama	0	0.91(0.64 to 1.18)	0	0.72(0.43 to 1.02)	0.001	0.48(0.19 to 0.78)
Papua New Guinea	0.006	0.43(0.12 to 0.75)	0	0.27(0.17 to 0.37)	0.274	0.21(-0.17 to 0.59)
Paraguay	0	2.38(2.08 to 2.68)	0	2.25(1.98 to 2.52)	0	1.77(1.51 to 2.03)
Peru	0	1.15(0.85 to 1.45)	0	1.04(0.81 to 1.27)	0	0.65(0.37 to 0.93)
Philippines	0.026	0.19(0.02 to 0.35)	0.005	-0.19(-0.32 to -0.06)	0.357	0.1(-0.12 to 0.33)
Poland	0	0.92(0.82 to 1.01)	0	0.91(0.8 to 1.01)	0.846	-0.01(-0.11 to 0.09)
Portugal	0	0.77(0.56 to 0.98)	0	0.79(0.56 to 1.02)	0.558	-0.13(-0.57 to 0.31)
Puerto Rico	0.445	-0.2(-0.71 to 0.31)	0.066	-0.27(-0.56 to 0.02)	0	-0.75(-1.07 to -0.42)
Qatar	0.046	0.52(0.01 to 1.04)	0.194	0.44(-0.22 to 1.1)	0.022	-0.39(-0.73 to -0.06)
Republic of Korea	0	0.75(0.4 to 1.11)	0	0.88(0.5 to 1.27)	0.118	-0.26(-0.59 to 0.07)
Republic of Moldova	0	-1.15(-1.59 to -0.7)	0	-1.16(-1.59 to -0.72)	0	-1.62(-2 to -1.24)
Romania	0	0.9(0.68 to 1.13)	0	0.94(0.72 to 1.16)	0.032	0.16(0.01 to 0.31)
Russian Federation	0	-1.07(-1.66 to -0.48)	0.003	-1.17(-1.95 to -0.39)	0	-1.61(-2.18 to -1.03)
Rwanda	0	1.28(1.06 to 1.51)	0	1.06(0.87 to 1.25)	0	1.11(0.79 to 1.43)
Saint Kitts and Nevis	0.029	0.8(0.08 to 1.51)	0.057	0.51(-0.01 to 1.04)	0.241	0.46(-0.31 to 1.24)
Saint Lucia	0	1.05(0.9 to 1.19)	0	0.81(0.47 to 1.14)	0	0.72(0.37 to 1.08)
Saint Vincent and the Grenadines	0	2.24(2.05 to 2.43)	0	2.11(1.84 to 2.38)	0	1.7(1.22 to 2.18)
Samoa	0.678	-0.01(-0.08 to 0.05)	0	-0.27(-0.32 to -0.22)	0	-0.37(-0.4 to -0.35)
San Marino	0	0.6(0.52 to 0.69)	0	0.52(0.41 to 0.62)	0	0.34(0.25 to 0.43)
Sao Tome and Principe	0	0.4(0.2 to 0.61)	0.025	0.42(0.05 to 0.79)	0.794	0.08(-0.53 to 0.69)
Saudi Arabia	0	0.56(0.43 to 0.69)	0.043	-0.15(-0.29 to 0)	0.294	-0.07(-0.2 to 0.06)
Senegal	0.815	0.14(-1.03 to 1.32)	0.871	0.08(-0.92 to 1.09)	0.469	-0.46(-1.71 to 0.8)
Serbia	0	0.55(0.29 to 0.81)	0.001	0.45(0.19 to 0.71)	0.606	-0.08(-0.37 to 0.22)
Seychelles	0	0.54(0.32 to 0.75)	0	0.36(0.18 to 0.54)	0.087	0.24(-0.03 to 0.51)
Sierra Leone	0.001	0.55(0.23 to 0.87)	0.007	0.43(0.12 to 0.74)	0.541	0.13(-0.28 to 0.53)
Singapore	0.031	-0.63(-1.2 to -0.06)	0.018	-0.6(-1.09 to -0.1)	0	-1.3(-1.88 to -0.72)
Slovakia	0.012	-0.29(-0.52 to -0.06)	0	-0.46(-0.68 to -0.24)	0	-0.66(-0.95 to -0.37)

Slovenia	0	1.7(1.27 to 2.13)	0	1.68(1.27 to 2.09)	0.001	0.85(0.35 to 1.36)
Solomon Islands	0	0.68(0.51 to 0.84)	0	0.41(0.26 to 0.56)	0	0.44(0.29 to 0.6)
Somalia	0	0.84(0.7 to 0.98)	0	0.64(0.53 to 0.75)	0	0.72(0.49 to 0.95)
South Africa	0	0.4(0.3 to 0.51)	0	0.21(0.1 to 0.31)	0.484	0.09(-0.16 to 0.33)
South Sudan	0	0.63(0.43 to 0.83)	0	0.42(0.25 to 0.59)	0.071	0.24(-0.02 to 0.51)
Spain	0	1.65(1.52 to 1.79)	0	1.56(1.39 to 1.72)	0	0.65(0.48 to 0.82)
Sri Lanka	0	0.72(0.35 to 1.09)	0	0.54(0.24 to 0.83)	0.512	0.21(-0.41 to 0.83)
Sudan	0	1.2(1.08 to 1.33)	0	0.93(0.82 to 1.03)	0	0.9(0.77 to 1.02)
Suriname	0	0.92(0.55 to 1.28)	0.009	0.86(0.21 to 1.52)	0.007	0.5(0.14 to 0.86)
Sweden	0	0.42(0.27 to 0.57)	0	0.34(0.15 to 0.52)	0.097	-0.19(-0.41 to 0.03)
Switzerland	0.001	-0.83(-1.31 to -0.34)	0	-1.07(-1.6 to -0.54)	0	-1.8(-2.09 to -1.51)
Syrian Arab Republic	0.339	-0.06(-0.18 to 0.06)	0	-0.26(-0.36 to -0.16)	0	-0.4(-0.54 to -0.25)
Taiwan (Province of China)	0	3.79(2.64 to 4.95)	0	3.77(2.6 to 4.96)	0	2.91(2.32 to 3.5)
Tajikistan	0	-0.38(-0.56 to -0.2)	0	-0.45(-0.63 to -0.27)	0	-0.94(-1.11 to -0.77)
Thailand	0	3.13(2.87 to 3.4)	0	2.99(2.71 to 3.26)	0	2.59(2.29 to 2.9)
Timor-Leste	0	1.28(0.91 to 1.65)	0	1.03(0.75 to 1.31)	0	0.77(0.37 to 1.17)
Togo	0.011	0.41(0.09 to 0.72)	0.028	0.34(0.04 to 0.64)	0.537	-0.13(-0.55 to 0.29)
Tokelau	0.914	0.01(-0.11 to 0.13)	0.001	-0.18(-0.29 to -0.07)	0.002	-0.31(-0.51 to -0.11)
Tonga	0	0.65(0.49 to 0.8)	0	0.44(0.27 to 0.61)	0	0.49(0.36 to 0.62)
Trinidad and Tobago	0.569	0.08(-0.19 to 0.34)	0.841	-0.02(-0.2 to 0.16)	0.076	-0.17(-0.35 to 0.02)
Tunisia	0	0.17(0.1 to 0.24)	0.231	0.05(-0.03 to 0.12)	0	-0.36(-0.44 to -0.29)
Turkey	0	-0.49(-0.63 to -0.35)	0	-0.6(-0.72 to -0.48)	0	-1.12(-1.29 to -0.95)
Turkmenistan	0.437	0.25(-0.37 to 0.87)	0.668	0.14(-0.51 to 0.81)	0.277	-0.27(-0.76 to 0.22)
Tuvalu	0.732	-0.02(-0.11 to 0.08)	0	-0.16(-0.22 to -0.11)	0	-0.41(-0.53 to -0.29)
Uganda	0	1.08(0.74 to 1.42)	0	0.82(0.52 to 1.13)	0	0.97(0.59 to 1.35)
Ukraine	0	-1.51(-2.31 to -0.71)	0	-1.32(-1.99 to -0.64)	0	-2.31(-3.31 to -1.3)
United Arab Emirates	0.513	-0.09(-0.35 to 0.18)	0.127	-0.24(-0.55 to 0.07)	0	-0.33(-0.51 to -0.15)
United Kingdom	0.274	0.11(-0.09 to 0.31)	0	0.3(0.2 to 0.39)	0	-0.32(-0.43 to -0.22)
United Republic of Tanzania	0	1.45(1.24 to 1.66)	0	1.23(1.04 to 1.42)	0	1.27(0.91 to 1.64)
United States of America	0	0.57(0.46 to 0.69)	0	0.5(0.4 to 0.6)	0.833	-0.02(-0.19 to 0.16)
United States Virgin Islands	0.806	-0.01(-0.1 to 0.07)	0.207	-0.09(-0.22 to 0.05)	0	-0.52(-0.73 to -0.31)
Uruguay	0.495	0.07(-0.13 to 0.27)	0.354	-0.11(-0.33 to 0.12)	0	-0.42(-0.64 to -0.19)
Uzbekistan	0.189	0.14(-0.07 to 0.36)	0.779	-0.03(-0.24 to 0.18)	0.052	-0.23(-0.46 to 0)
Vanuatu	0	0.79(0.61 to 0.96)	0	0.49(0.32 to 0.66)	0	0.63(0.4 to 0.85)
Venezuela (Bolivarian Republic of)	0	0.82(0.44 to 1.2)	0.003	0.63(0.22 to 1.04)	0	0.5(0.26 to 0.74)
Viet Nam	0	0.86(0.76 to 0.96)	0	0.68(0.59 to 0.78)	0	0.53(0.47 to 0.58)
Yemen	0	1.2(1.05 to 1.36)	0	0.95(0.82 to 1.09)	0	1.1(0.98 to 1.21)
Zambia	0	0.87(0.77 to 0.97)	0	0.71(0.54 to 0.89)	0	0.56(0.33 to 0.79)
Zimbabwe	0.01	0.64(0.15 to 1.13)	0.14	0.35(-0.12 to 0.82)	0.048	0.61(0 to 1.22)
Global	0	0.56(0.35 to 0.77)	0	-0.28(-0.43 to -0.13)	0.004	-0.23(-0.39 to -0.07)
SDI regions						
High SDI	0.567	0.09(-0.21 to 0.39)	0	-0.95(-1.13 to -0.77)	0	-1.05(-1.2 to -0.9)
High-middle SDI	0	1.58(1.32 to 1.84)	0.225	0.13(-0.08 to 0.33)	0.723	-0.05(-0.3 to 0.21)
Middle SDI	0	2.85(2.62 to 3.08)	0	0.92(0.74 to 1.11)	0	0.89(0.69 to 1.09)
Low-middle SDI	0	1.45(1.25 to 1.65)	0.001	0.83(0.34 to 1.33)	0	0.8(0.59 to 1)
Low SDI	0	1.12(0.99 to 1.25)	0	0.84(0.68 to 1)	0	0.72(0.56 to 0.88)
Regions						

Andean Latin America	0	2.71(2.06 to 3.37)	0	1.55(0.91 to 2.2)	0	1.27(0.65 to 1.89)
Australasia	0.029	0.54(0.05 to 1.02)	0	-0.63(-0.98 to -0.28)	0	-0.78(-1.13 to -0.42)
Caribbean	0	0.84(0.69 to 0.98)	0.033	-0.13(-0.26 to -0.01)	0.552	-0.1(-0.42 to 0.23)
Central Asia	0	1.27(0.95 to 1.59)	0	0.54(0.36 to 0.72)	0.605	-0.05(-0.23 to 0.13)
Central Europe	0	3.33(3.01 to 3.65)	0	1.75(1.55 to 1.94)	0	1.59(1.45 to 1.73)
Central Latin America	0	1.37(1.09 to 1.66)	0.126	0.25(-0.07 to 0.57)	0.374	0.14(-0.17 to 0.47)
Central Sub-Saharan Africa	0	2.62(2.33 to 2.91)	0	2.47(2.21 to 2.73)	0	2.35(2.09 to 2.61)
East Asia	0	4.94(4.58 to 5.3)	0	1.75(1.53 to 1.98)	0	1.72(1.48 to 1.95)
Eastern Europe	0	1.74(0.81 to 2.67)	0.15	0.55(-0.2 to 1.3)	0.49	0.3(-0.55 to 1.16)
Eastern Sub-Saharan Africa	0	1.26(1.09 to 1.42)	0	1.08(0.95 to 1.22)	0	0.9(0.76 to 1.04)
High-income Asia Pacific	0	0.96(0.79 to 1.14)	0	-0.81(-1.14 to -0.47)	0	-0.67(-0.92 to -0.41)
High-income North America	0.216	-0.24(-0.62 to 0.14)	0	-0.75(-0.89 to -0.62)	0	-1.06(-1.24 to -0.88)
North Africa and Middle East	0	2.09(1.84 to 2.35)	0	0.54(0.4 to 0.67)	0.051	0.22(0 to 0.44)
Oceania	0.434	-0.07(-0.24 to 0.1)	0	-0.37(-0.52 to -0.23)	0.001	-0.25(-0.4 to -0.1)
South Asia	0	1.1(0.86 to 1.35)	0.073	0.49(-0.05 to 1.03)	0	0.55(0.31 to 0.79)
Southeast Asia	0	2.14(1.9 to 2.37)	0	1.2(0.98 to 1.41)	0	1.05(0.96 to 1.14)
Southern Latin America	0.001	0.68(0.29 to 1.08)	0.001	-0.43(-0.67 to -0.18)	0	-0.61(-0.78 to -0.45)
Southern Sub-Saharan Africa	0.005	0.73(0.22 to 1.24)	0.292	0.33(-0.28 to 0.94)	0.324	0.23(-0.23 to 0.69)
Tropical Latin America	0	1.26(1.14 to 1.39)	0	0.42(0.29 to 0.55)	0.006	0.17(0.05 to 0.3)
Western Europe	0	0.88(0.57 to 1.19)	0	-0.43(-0.6 to -0.26)	0	-0.68(-0.81 to -0.54)
Western Sub-Saharan Africa	0	0.86(0.73 to 1)	0	0.55(0.41 to 0.68)	0	0.55(0.43 to 0.66)
Countries						
Afghanistan	0	0.76(0.7 to 0.82)	0	0.51(0.45 to 0.57)	0	0.49(0.45 to 0.54)
Albania	0	4.82(4.45 to 5.2)	0	2.62(1.96 to 3.3)	0	2.68(2.06 to 3.3)
Algeria	0	1.26(1.13 to 1.38)	0.488	-0.04(-0.17 to 0.08)	0.008	-0.14(-0.25 to -0.04)
American Samoa	0.176	0.23(-0.1 to 0.56)	0.014	-0.32(-0.58 to -0.07)	0.388	-0.1(-0.34 to 0.13)
Andorra	0	1.29(1.11 to 1.47)	0	-0.26(-0.37 to -0.16)	0.024	-0.12(-0.22 to -0.02)
Angola	0	3.3(3.14 to 3.47)	0	3.06(2.89 to 3.24)	0	2.82(2.63 to 3.01)
Antigua and Barbuda	0	1.36(0.62 to 2.12)	0.142	0.65(-0.22 to 1.53)	0.282	0.37(-0.3 to 1.04)
Argentina	0.046	0.36(0.01 to 0.72)	0	-0.58(-0.81 to -0.36)	0	-0.73(-0.96 to -0.5)
Armenia	0.066	0.5(-0.03 to 1.04)	0.049	-0.48(-0.96 to 0)	0	-0.92(-1.43 to -0.41)
Australia	0.103	0.4(-0.08 to 0.89)	0	-0.75(-1.15 to -0.34)	0	-0.89(-1.24 to -0.54)
Austria	0.001	0.72(0.31 to 1.14)	0	-0.45(-0.67 to -0.23)	0	-0.82(-1.04 to -0.6)
Azerbaijan	0	2.04(1.73 to 2.35)	0	1.3(1.07 to 1.53)	0	0.73(0.56 to 0.9)
Bahamas	0.123	0.57(-0.15 to 1.3)	0.924	0.02(-0.49 to 0.54)	0.775	-0.08(-0.61 to 0.46)
Bahrain	0.696	0.18(-0.72 to 1.08)	0.001	-1.37(-2.18 to -0.54)	0	-1.79(-2.67 to -0.9)
Bangladesh	0.005	0.55(0.17 to 0.92)	0.406	-0.17(-0.56 to 0.23)	0.131	-0.27(-0.61 to 0.08)
Barbados	0	1.81(1.22 to 2.41)	0	0.9(0.82 to 0.97)	0	0.72(0.65 to 0.8)
Belarus	0	1.59(1.3 to 1.88)	0.121	-0.19(-0.42 to 0.05)	0	-0.4(-0.63 to -0.18)
Belgium	0.007	0.79(0.22 to 1.36)	0.146	-0.71(-1.66 to 0.25)	0.073	-0.63(-1.32 to 0.06)
Belize	0	2.22(1.44 to 3.01)	0	1.69(1.11 to 2.27)	0	1.69(1.14 to 2.24)
Benin	0	0.71(0.59 to 0.83)	0	0.52(0.41 to 0.63)	0	0.38(0.22 to 0.54)
Bermuda	0.001	0.95(0.37 to 1.53)	0.003	-0.89(-1.48 to -0.3)	0.001	-0.96(-1.5 to -0.42)
Bhutan	0	1.73(1.61 to 1.84)	0	0.9(0.83 to 0.97)	0	0.73(0.65 to 0.81)
Bolivia (Plurinational State of)	0	2.68(2.47 to 2.89)	0	2.06(2 to 2.12)	0	1.63(1.57 to 1.69)
Bosnia and Herzegovina	0	4.76(4.24 to 5.29)	0	3.17(2.65 to 3.69)	0	2.98(2.62 to 3.35)

Botswana	0	2.17(1.94 to 2.4)	0	1.44(1.26 to 1.61)	0	1.42(1.17 to 1.67)
Brazil	0	1.2(1.03 to 1.38)	0	0.37(0.24 to 0.5)	0.085	0.11(-0.02 to 0.24)
Brunei Darussalam	0	1.44(1.01 to 1.87)	0	0.46(0.23 to 0.68)	0.013	0.27(0.06 to 0.48)
Bulgaria	0	2.25(1.9 to 2.61)	0	1.21(0.93 to 1.49)	0	1.11(0.86 to 1.37)
Burkina Faso	0	0.95(0.62 to 1.29)	0	0.71(0.38 to 1.04)	0	0.69(0.39 to 0.99)
Burundi	0	0.74(0.54 to 0.94)	0	0.61(0.46 to 0.77)	0	0.52(0.34 to 0.71)
Cabo Verde	0	3.55(2.28 to 4.83)	0	2.92(2.09 to 3.77)	0	2.55(1.93 to 3.17)
Cambodia	0	1.63(1.52 to 1.75)	0	1.07(1.02 to 1.13)	0	0.84(0.78 to 0.89)
Cameroon	0	0.72(0.52 to 0.93)	0	0.42(0.25 to 0.6)	0	0.32(0.14 to 0.49)
Canada	0.072	0.59(-0.05 to 1.24)	0.037	-0.65(-1.26 to -0.04)	0.005	-0.81(-1.37 to -0.25)
Central African Republic	0	2.16(1.84 to 2.47)	0	2.16(1.87 to 2.45)	0	2.03(1.74 to 2.32)
Chad	0	0.8(0.68 to 0.91)	0	0.71(0.6 to 0.81)	0	0.63(0.51 to 0.74)
Chile	0	1.85(1.67 to 2.03)	0.001	0.24(0.09 to 0.39)	0.337	-0.07(-0.23 to 0.08)
China	0	5.07(4.72 to 5.41)	0	1.8(1.54 to 2.06)	0	1.76(1.51 to 2.01)
Colombia	0.258	0.52(-0.38 to 1.43)	0.044	-0.91(-1.78 to -0.03)	0.033	-1.09(-2.08 to -0.09)
Comoros	0	1.88(1.75 to 2.01)	0	1.58(1.43 to 1.73)	0	1.52(1.3 to 1.75)
Congo	0	2.17(1.91 to 2.42)	0	1.93(1.72 to 2.14)	0	1.78(1.5 to 2.05)
Cook Islands	0.187	-0.07(-0.17 to 0.03)	0	-1.27(-1.38 to -1.17)	0	-1.21(-1.33 to -1.08)
Costa Rica	0.234	0.93(-0.59 to 2.47)	0.979	0.02(-1.49 to 1.55)	0.858	-0.15(-1.74 to 1.47)
Croatia	0.12	0.72(-0.19 to 1.64)	0.043	-0.7(-1.38 to -0.02)	0.025	-0.94(-1.75 to -0.12)
Cuba	0	0.77(0.67 to 0.87)	0	-0.35(-0.52 to -0.18)	0.003	-0.44(-0.73 to -0.15)
Cyprus	0	3.12(2.55 to 3.69)	0.049	0.58(0 to 1.17)	0.006	0.67(0.19 to 1.16)
Czechia	0	2.68(1.81 to 3.56)	0	1.23(0.59 to 1.88)	0.008	0.79(0.2 to 1.38)
C么te d'Ivoire	0	0.46(0.31 to 0.61)	0.003	0.3(0.1 to 0.49)	0.002	0.21(0.08 to 0.35)
Democratic People's Republic of Korea	0	0.69(0.55 to 0.84)	0	0.37(0.24 to 0.51)	0	0.44(0.31 to 0.56)
Democratic Republic of the Congo	0	2.51(2.19 to 2.82)	0	2.4(2.11 to 2.7)	0	2.26(1.93 to 2.59)
Denmark	0	0.73(0.55 to 0.92)	0	-0.68(-0.88 to -0.47)	0	-1.04(-1.18 to -0.89)
Djibouti	0	1.92(1.77 to 2.07)	0	1.61(1.48 to 1.73)	0	1.67(1.49 to 1.85)
Dominica	0	1.06(0.7 to 1.42)	0	0.92(0.66 to 1.18)	0	0.8(0.44 to 1.17)
Dominican Republic	0	3.65(2.97 to 4.32)	0	2.87(2.36 to 3.37)	0	2.83(2.33 to 3.33)
Ecuador	0	3.54(2.85 to 4.24)	0	2.33(1.55 to 3.12)	0	2.24(1.64 to 2.84)
Egypt	0	2.93(1.81 to 4.06)	0	1.74(1.13 to 2.36)	0	1.54(1.36 to 1.72)
El Salvador	0	4.73(3.99 to 5.48)	0	3.45(2.76 to 4.16)	0	3.34(2.59 to 4.1)
Equatorial Guinea	0	4.49(4.02 to 4.97)	0	3.95(3.61 to 4.29)	0	3.49(3.11 to 3.88)
Eritrea	0	2.48(2.29 to 2.68)	0	2.27(2.11 to 2.43)	0	2.18(1.96 to 2.41)
Estonia	0	4.89(3.33 to 6.48)	0	2.97(1.32 to 4.64)	0.002	2.51(0.9 to 4.14)
Eswatini	0	1.24(0.85 to 1.63)	0	1.08(0.74 to 1.41)	0	0.99(0.58 to 1.39)
Ethiopia	0	0.62(0.47 to 0.76)	0	0.33(0.21 to 0.45)	0.006	0.18(0.05 to 0.31)
Fiji	0.646	0.03(-0.11 to 0.18)	0.003	-0.23(-0.38 to -0.08)	0	-0.27(-0.39 to -0.16)
Finland	0.029	0.49(0.05 to 0.93)	0	-1.1(-1.46 to -0.74)	0	-1.23(-1.6 to -0.87)
France	0	0.81(0.52 to 1.1)	0	-0.67(-0.88 to -0.47)	0	-0.95(-1.1 to -0.79)
Gabon	0	2.55(2.17 to 2.93)	0	2.22(1.88 to 2.56)	0	2.05(1.7 to 2.4)
Gambia	0.001	1.3(0.51 to 2.1)	0.006	1.07(0.31 to 1.84)	0.013	1.11(0.23 to 2)
Georgia	0	3.03(2.21 to 3.85)	0	2.98(2.31 to 3.66)	0	2.52(1.94 to 3.1)
Germany	0	1.27(0.93 to 1.62)	0.848	0.02(-0.16 to 0.2)	0.002	-0.32(-0.52 to -0.12)
Ghana	0	0.52(0.33 to 0.72)	0.002	0.15(0.05 to 0.25)	0.225	0.1(-0.06 to 0.27)
Global	0	0.56(0.35 to 0.77)	0	-0.28(-0.43 to -0.13)	0.004	-0.23(-0.39 to -0.07)
Greece	0	1.48(0.98 to 1.99)	0.256	0.29(-0.21 to 0.8)	0.213	0.29(-0.17 to 0.76)
Greenland	0.017	-0.44(-0.79 to -0.08)	0	-1.32(-1.69 to -0.94)	0	-1.29(-1.58 to -1)

Chronic lymphoid leukemia	Grenada	0	-1.39(-2.1 to -0.68)	0	-2.15(-2.91 to -1.39)	0	-1.94(-2.6 to -1.26)
	Guam	0	-2.3(-3.25 to -1.35)	0	-3.42(-4.23 to -2.61)	0	-2.02(-2.83 to -1.21)
	Guatemala	0	2.75(2.45 to 3.04)	0	2.04(1.66 to 2.42)	0	1.99(1.73 to 2.25)
	Guinea	0	0.47(0.39 to 0.56)	0	0.32(0.24 to 0.41)	0	0.31(0.24 to 0.38)
	Guinea-Bissau	0	0.45(0.38 to 0.53)	0	0.3(0.23 to 0.37)	0	0.18(0.11 to 0.25)
	Guyana	0	4.2(3.59 to 4.82)	0	3.64(3.05 to 4.23)	0	3.94(3.34 to 4.55)
	Haiti	0	0.48(0.35 to 0.61)	0	0.31(0.21 to 0.42)	0	0.18(0.09 to 0.26)
	Honduras	0	3.3(2.75 to 3.85)	0	2.81(2.33 to 3.3)	0	2.4(1.85 to 2.94)
	Hungary	0	0.96(0.73 to 1.19)	0.044	-0.55(-1.08 to -0.02)	0	-0.71(-1.08 to -0.35)
	Iceland	0	-1.53(-2.05 to -1)	0	-2.62(-3.3 to -1.93)	0	-2.65(-3.11 to -2.19)
	India	0	1.16(0.82 to 1.5)	0.193	0.5(-0.25 to 1.25)	0.001	0.59(0.24 to 0.94)
	Indonesia	0	2.62(2.48 to 2.76)	0	2.22(2.11 to 2.33)	0	1.87(1.74 to 1.99)
	Iran (Islamic Republic of)	0	1.67(1.33 to 2)	0.14	-0.12(-0.28 to 0.04)	0.002	-0.22(-0.36 to -0.08)
	Iraq	0	2.07(1.65 to 2.5)	0	0.98(0.59 to 1.38)	0	0.76(0.38 to 1.15)
	Ireland	0.001	1.29(0.55 to 2.03)	0.021	-0.63(-1.16 to -0.09)	0.03	-0.68(-1.3 to -0.06)
	Israel	0	0.87(0.67 to 1.07)	0	-0.69(-0.99 to -0.39)	0	-0.84(-1.07 to -0.6)
	Italy	0	0.81(0.68 to 0.94)	0	-0.42(-0.61 to -0.23)	0	-0.67(-0.82 to -0.51)
	Jamaica	0	5.95(5.2 to 6.71)	0	5.19(4.45 to 5.94)	0	5.28(4.53 to 6.04)
	Japan	0	0.56(0.36 to 0.76)	0	-1.03(-1.16 to -0.91)	0	-0.99(-1.14 to -0.83)
	Jordan	0	2.23(1.76 to 2.7)	0.028	0.58(0.06 to 1.1)	0.021	0.28(0.04 to 0.51)
	Kazakhstan	0	1.23(0.95 to 1.51)	0.748	0.08(-0.4 to 0.56)	0.151	-0.34(-0.79 to 0.12)
	Kenya	0	1.11(0.93 to 1.29)	0	1.15(1.02 to 1.28)	0	1.07(0.94 to 1.2)
	Kiribati	0.071	0.25(-0.02 to 0.52)	0.194	0.12(-0.06 to 0.3)	0.821	-0.02(-0.19 to 0.15)
	Kuwait	0	2.63(1.26 to 4.02)	0.219	0.91(-0.54 to 2.39)	0.208	0.84(-0.47 to 2.17)
	Kyrgyzstan	0	-1.93(-2.56 to -1.3)	0	-2.52(-3.17 to -1.87)	0	-3.16(-3.96 to -2.37)
	Lao People's Democratic Republic	0	0.72(0.65 to 0.79)	0	0.4(0.33 to 0.46)	0.055	0.09(0 to 0.18)
	Latvia	0.092	1(-0.16 to 2.17)	0.647	-0.28(-1.46 to 0.92)	0.443	-0.46(-1.63 to 0.72)
	Lebanon	0	3.17(2.88 to 3.46)	0.003	0.41(0.14 to 0.67)	0	0.52(0.26 to 0.77)
	Lesotho	0	2.77(2.59 to 2.95)	0	2.63(2.46 to 2.8)	0	2.77(2.56 to 2.98)
	Liberia	0	0.56(0.32 to 0.79)	0.044	0.3(0.01 to 0.59)	0.131	0.2(-0.06 to 0.47)
	Libya	0	1.97(1.38 to 2.55)	0.007	0.89(0.25 to 1.55)	0.005	0.9(0.28 to 1.53)
	Lithuania	0.252	1.13(-0.8 to 3.1)	0.556	0.75(-1.72 to 3.29)	0.342	0.52(-0.55 to 1.59)
	Luxembourg	0.001	0.56(0.22 to 0.89)	0	-1.12(-1.46 to -0.78)	0	-1.22(-1.47 to -0.96)
	Madagascar	0	1.9(1.75 to 2.05)	0	1.74(1.6 to 1.88)	0	1.74(1.6 to 1.89)
	Malawi	0	0.76(0.55 to 0.98)	0	0.62(0.45 to 0.79)	0	0.51(0.3 to 0.72)
	Malaysia	0	2.11(1.82 to 2.4)	0.013	1.01(0.22 to 1.82)	0	0.82(0.57 to 1.08)
	Maldives	0	1.71(1.33 to 2.1)	0.183	-0.17(-0.42 to 0.08)	0	-0.55(-0.78 to -0.31)
	Mali	0	0.41(0.27 to 0.55)	0.005	0.17(0.05 to 0.3)	0.186	0.09(-0.04 to 0.22)
	Malta	0	0.43(0.23 to 0.63)	0	-1.18(-1.45 to -0.9)	0	-1.22(-1.46 to -0.98)
	Marshall Islands	0.002	-0.25(-0.41 to -0.09)	0	-0.6(-0.76 to -0.44)	0	-0.38(-0.53 to -0.23)
	Mauritania	0.053	0.15(0 to 0.3)	0.002	-0.3(-0.48 to -0.11)	0	-0.46(-0.66 to -0.26)
	Mauritius	0.007	1.19(0.32 to 2.07)	0.896	0.06(-0.85 to 0.98)	0.372	0.35(-0.42 to 1.14)
	Mexico	0	1.75(1.44 to 2.07)	0	0.57(0.39 to 0.75)	0	0.39(0.19 to 0.6)
	Micronesia (Federated States of)	0	0.55(0.45 to 0.65)	0.112	-0.07(-0.15 to 0.02)	0.229	0.05(-0.03 to 0.14)
	Monaco	0	1.39(1.29 to 1.5)	0	0.34(0.24 to 0.44)	0	0.27(0.18 to 0.36)
	Mongolia	0	1.8(1.53 to 2.08)	0	0.94(0.65 to 1.22)	0	0.61(0.29 to 0.93)
	Montenegro	0	3.59(3.28 to 3.9)	0	2.4(2.04 to 2.76)	0	2.43(2.1 to 2.76)
	Morocco	0	1.93(1.39 to 2.48)	0	1.09(0.53 to 1.64)	0	1(0.71 to 1.28)
	Mozambique	0	2.05(1.94 to 2.16)	0	1.85(1.74 to 1.96)	0	1.88(1.75 to 2.01)
	Myanmar	0	1.08(0.99 to 1.17)	0	0.6(0.51 to 0.69)	0	0.3(0.18 to 0.42)
	Namibia	0	1.84(1.56 to 2.12)	0	1.29(1.11 to 1.47)	0	1.12(0.73 to 1.51)
	Nauru	0	0.36(0.19 to 0.54)	0	-0.3(-0.42 to -0.17)	0.006	-0.11(-0.19 to -0.03)

Nepal	0	1.8(1.71 to 1.89)	0	1.32(1.23 to 1.41)	0	1.06(0.99 to 1.14)
Netherlands	0.004	-2.79(-4.63 to -0.92)	0	-4.25(-6.09 to -2.38)	0	-4.14(-5.66 to -2.59)
New Zealand	0	1.24(0.69 to 1.8)	0.965	-0.01(-0.6 to 0.58)	0.644	-0.12(-0.64 to 0.4)
Nicaragua	0	1.32(0.72 to 1.92)	0.907	-0.08(-1.35 to 1.22)	0.091	-0.44(-0.94 to 0.07)
Niger	0	0.8(0.68 to 0.92)	0	0.61(0.49 to 0.72)	0	0.55(0.41 to 0.69)
Nigeria	0	1.11(0.92 to 1.29)	0	0.76(0.62 to 0.9)	0	0.8(0.64 to 0.96)
Niue	0	0.9(0.66 to 1.13)	0	-0.3(-0.46 to -0.14)	0	-0.29(-0.45 to -0.14)
North Macedonia	0	4.84(4.47 to 5.21)	0	3.31(3.02 to 3.6)	0	3.27(2.98 to 3.56)
Northern Mariana Islands	0.076	-0.48(-1.01 to 0.05)	0	-1.68(-1.9 to -1.47)	0	-1.02(-1.25 to -0.8)
Norway	0	0.5(0.26 to 0.73)	0	-0.72(-0.82 to -0.62)	0	-0.95(-1.06 to -0.84)
Oman	0	2.99(2.46 to 3.53)	0	1.2(0.93 to 1.46)	0	0.82(0.57 to 1.06)
Pakistan	0	1.32(1.23 to 1.4)	0	0.94(0.89 to 1)	0	0.92(0.86 to 0.98)
Palau	0	0.98(0.88 to 1.08)	0	0.13(0.08 to 0.18)	0.014	0.1(0.02 to 0.19)
Palestine	0	2.86(2.6 to 3.12)	0	1.75(1.17 to 2.34)	0	1.46(0.97 to 1.96)
Panama	0	1.08(0.5 to 1.66)	0.97	-0.01(-0.67 to 0.65)	0.244	-0.37(-1 to 0.25)
Papua New Guinea	0.002	0.22(0.08 to 0.35)	0.791	-0.02(-0.14 to 0.11)	0.734	0.03(-0.14 to 0.19)
Paraguay	0	3.67(3.31 to 4.04)	0	2.8(2.51 to 3.09)	0	2.69(2.41 to 2.97)
Peru	0	2.49(1.42 to 3.57)	0.1	1.01(-0.19 to 2.23)	0.278	0.75(-0.6 to 2.13)
Philippines	0.321	-0.22(-0.64 to 0.21)	0	-0.68(-0.96 to -0.39)	0.017	-0.39(-0.71 to -0.07)
Poland	0	6.62(6.29 to 6.95)	0	4.58(4.26 to 4.9)	0	4.4(4.04 to 4.76)
Portugal	0	2.93(2.15 to 3.71)	0.015	0.77(0.15 to 1.38)	0.061	0.62(-0.03 to 1.27)
Puerto Rico	0.929	0.04(-0.75 to 0.83)	0	-1.59(-2.4 to -0.77)	0.001	-1.41(-2.2 to -0.62)
Qatar	0.004	2.55(0.81 to 4.32)	0	1.15(0.8 to 1.5)	0.882	0.14(-1.67 to 1.98)
Republic of Korea	0	4.09(3.77 to 4.4)	0	0.5(0.24 to 0.76)	0	0.58(0.31 to 0.85)
Republic of Moldova	0.935	-0.05(-1.18 to 1.1)	0.159	-0.91(-2.17 to 0.36)	0.075	-1.2(-2.5 to 0.12)
Romania	0	3.14(2.63 to 3.65)	0	1.57(0.99 to 2.16)	0	1.15(0.54 to 1.77)
Russian Federation	0	2.4(1.27 to 3.55)	0.025	1.1(0.14 to 2.08)	0.091	0.85(-0.14 to 1.84)
Rwanda	0	1.4(1.11 to 1.7)	0	1.13(0.9 to 1.35)	0	0.86(0.6 to 1.13)
Saint Kitts and Nevis	0	1.57(1.01 to 2.14)	0.144	0.46(-0.16 to 1.08)	0.037	0.47(0.03 to 0.92)
Saint Lucia	0	0.79(0.48 to 1.11)	0.922	-0.02(-0.4 to 0.36)	0.648	-0.06(-0.29 to 0.18)
Saint Vincent and the Grenadines	0	1.84(1.55 to 2.14)	0	1.44(1.23 to 1.65)	0	1.36(1.16 to 1.57)
Samoa	0.06	0.24(-0.01 to 0.5)	0	-0.42(-0.59 to -0.24)	0.008	-0.22(-0.38 to -0.06)
San Marino	0	1.51(1.36 to 1.66)	0.621	0.04(-0.11 to 0.18)	0	0.32(0.16 to 0.48)
Sao Tome and Principe	0	1.65(1.47 to 1.82)	0	1.18(1.01 to 1.35)	0	1.14(0.96 to 1.32)
Saudi Arabia	0	3.25(2.95 to 3.55)	0	0.85(0.72 to 0.99)	0	1.08(0.97 to 1.19)
Senegal	0.006	0.81(0.23 to 1.39)	0.023	0.61(0.08 to 1.14)	0.115	0.51(-0.12 to 1.14)
Serbia	0	3.51(2.92 to 4.12)	0	1.75(1.15 to 2.36)	0	1.42(0.83 to 2.01)
Seychelles	0	0.92(0.64 to 1.2)	0.97	0(-0.23 to 0.22)	0.102	-0.16(-0.35 to 0.03)
Sierra Leone	0	1(0.91 to 1.1)	0	0.76(0.65 to 0.86)	0	0.74(0.64 to 0.84)
Singapore	0	1.68(0.98 to 2.39)	0.62	-0.23(-1.12 to 0.67)	0.03	-0.4(-0.75 to -0.04)
Slovakia	0	1.47(1.09 to 1.85)	0.254	-0.17(-0.46 to 0.12)	0.276	-0.19(-0.52 to 0.15)
Slovenia	0.051	0.84(0 to 1.69)	0	-1.01(-1.35 to -0.67)	0.006	-1.05(-1.8 to -0.3)
Solomon Islands	0	0.69(0.49 to 0.89)	0.083	0.18(-0.02 to 0.38)	0.004	0.31(0.1 to 0.53)
Somalia	0	1.54(1.44 to 1.65)	0	1.54(1.46 to 1.63)	0	1.45(1.35 to 1.55)
South Africa	0.052	0.61(-0.01 to 1.24)	0.539	0.19(-0.4 to 0.78)	0.585	-0.21(-0.96 to 0.54)
South Sudan	0	0.93(0.8 to 1.06)	0	0.82(0.72 to 0.92)	0	0.92(0.75 to 1.1)
Spain	0	1.88(1.49 to 2.27)	0.005	0.27(0.08 to 0.46)	0.872	0.02(-0.22 to 0.26)
Sri Lanka	0	1.73(1.12 to 2.34)	0.946	0.03(-0.75 to 0.81)	0.99	0(-0.59 to 0.6)
Sudan	0	2.23(2.17 to 2.29)	0	1.57(1.5 to 1.64)	0	1.33(1.28 to 1.38)

Suriname	0	2.52(1.73 to 3.33)	0	1.92(1.16 to 2.69)	0	1.98(1.12 to 2.84)
Sweden	0	0.8(0.67 to 0.93)	0.792	-0.02(-0.16 to 0.12)	0.101	-0.29(-0.63 to 0.06)
Switzerland	0	-1.5(-2.02 to -0.98)	0	-2.43(-2.79 to -2.06)	0	-2.65(-3.1 to -2.18)
Syrian Arab Republic	0	1.65(1.35 to 1.94)	0.003	0.25(0.09 to 0.42)	0.184	0.12(-0.06 to 0.3)
Taiwan (Province of China)	0	1.67(1.24 to 2.11)	0.643	-0.11(-0.6 to 0.37)	0.178	-0.32(-0.78 to 0.14)
Tajikistan	0.008	0.98(0.25 to 1.71)	0.005	0.93(0.28 to 1.58)	0.866	0.05(-0.5 to 0.59)
Thailand	0	2.45(1.79 to 3.11)	0.002	1.04(0.37 to 1.72)	0	1.05(0.79 to 1.31)
Timor-Leste	0	2.2(2.01 to 2.39)	0	1.84(1.68 to 2)	0	1.53(1.33 to 1.74)
Togo	0	0.63(0.42 to 0.84)	0	0.46(0.24 to 0.68)	0.003	0.37(0.13 to 0.61)
Tokelau	0	0.62(0.45 to 0.79)	0	-0.41(-0.53 to -0.29)	0	-0.29(-0.42 to -0.17)
Tonga	0.282	0.28(-0.23 to 0.78)	0.219	-0.24(-0.62 to 0.14)	0.749	-0.05(-0.38 to 0.27)
Trinidad and Tobago	0.003	1.02(0.35 to 1.69)	0.673	0.14(-0.52 to 0.81)	0.64	0.17(-0.53 to 0.86)
Tunisia	0	2.49(2.31 to 2.67)	0	0.68(0.55 to 0.82)	0	0.66(0.53 to 0.78)
Turkey	0	1.9(1.5 to 2.29)	0.493	0.1(-0.18 to 0.38)	0.033	-0.42(-0.81 to -0.03)
Turkmenistan	0	1.62(0.89 to 2.36)	0.001	0.87(0.37 to 1.36)	0.209	0.39(-0.22 to 1.01)
Tuvalu	0.166	0.09(-0.04 to 0.21)	0	-0.58(-0.69 to -0.46)	0	-0.42(-0.5 to -0.33)
Uganda	0	1.75(1.49 to 2)	0	1.5(1.28 to 1.71)	0	1.43(1.24 to 1.62)
Ukraine	0.003	0.56(0.19 to 0.93)	0.332	-0.28(-0.85 to 0.29)	0.232	-0.38(-1 to 0.25)
United Arab Emirates	0.015	1.27(0.25 to 2.3)	0.288	0.6(-0.5 to 1.7)	0.404	0.41(-0.55 to 1.39)
United Kingdom	0.03	0.41(0.04 to 0.78)	0.001	-0.48(-0.75 to -0.21)	0	-0.74(-0.98 to -0.5)
United Republic of Tanzania	0	1.79(1.66 to 1.92)	0	1.51(1.41 to 1.62)	0	1.55(1.42 to 1.68)
United States of America	0.069	-0.33(-0.68 to 0.03)	0	-0.78(-0.9 to -0.65)	0	-1.11(-1.24 to -0.97)
United States Virgin Islands	0	1.14(0.91 to 1.36)	0.02	0.43(0.07 to 0.79)	0.042	0.36(0.01 to 0.71)
Uruguay	0	1.19(0.58 to 1.81)	0.665	0.18(-0.64 to 1.02)	0.505	0.18(-0.34 to 0.7)
Uzbekistan	0	1.65(1.27 to 2.03)	0	1.04(0.53 to 1.55)	0.094	0.42(-0.07 to 0.91)
Vanuatu	0.15	0.16(-0.06 to 0.37)	0.234	-0.14(-0.38 to 0.09)	0.272	0.16(-0.13 to 0.45)
Venezuela (Bolivarian Republic of)	0.21	0.63(-0.35 to 1.61)	0.23	-0.35(-0.92 to 0.22)	0.154	-0.57(-1.35 to 0.22)
Viet Nam	0	2.32(2.17 to 2.46)	0	1.15(1.07 to 1.23)	0	0.89(0.81 to 0.97)
Yemen	0	1.8(1.51 to 2.09)	0	1.32(1.21 to 1.43)	0	1.25(1.15 to 1.35)
Zambia	0	1.86(1.66 to 2.05)	0	1.45(1.35 to 1.56)	0	1.47(1.27 to 1.67)
Zimbabwe	0.006	0.55(0.16 to 0.94)	0.005	0.55(0.17 to 0.94)	0.002	0.6(0.22 to 0.99)
Global	0	-0.5(-0.76 to -0.24)	0	-2.18(-2.33 to -2.04)	0	-2.35(-2.47 to -2.24)
SDI regions						
High SDI	0.083	0.29(-0.04 to 0.62)	0	-3.82(-4.02 to -3.61)	0	-4.01(-4.16 to -3.85)
High-middle SDI	0.082	0.25(-0.03 to 0.54)	0	-2.53(-2.7 to -2.37)	0	-2.82(-3.04 to -2.61)
Middle SDI	0	-0.87(-0.97 to -0.76)	0	-1.19(-1.28 to -1.1)	0	-1.51(-1.61 to -1.41)
Low-middle SDI	0	-1.53(-1.7 to -1.35)	0	-1.44(-1.61 to -1.27)	0	-1.94(-2.09 to -1.79)
Low SDI	0	-2.02(-2.1 to -1.94)	0	-1.82(-1.92 to -1.73)	0	-2.55(-2.64 to -2.45)
Regions						
Andean Latin America	0.574	-0.13(-0.59 to 0.33)	0.694	-0.09(-0.51 to 0.34)	0.021	-0.53(-0.97 to -0.08)
Australasia	0.072	-0.82(-1.7 to 0.07)	0	-4.16(-4.71 to -3.61)	0	-4.32(-4.75 to -3.88)
Caribbean	0	-0.93(-1.26 to -0.59)	0	-1.43(-1.65 to -1.22)	0	-1.61(-1.7 to -1.51)
Central Asia	0	-1.45(-1.82 to -1.08)	0	-1.42(-1.77 to -1.08)	0	-2.07(-2.45 to -1.68)
Central Europe	0.001	-0.38(-0.6 to -0.16)	0	-2.76(-3.04 to -2.49)	0	-3.32(-3.63 to -3.01)
Central Latin America	0	-1.18(-1.39 to -0.96)	0	-1.38(-1.59 to -1.17)	0	-1.69(-1.91 to -1.47)
Central Sub-Saharan Africa	0	-1.18(-1.28 to -1.08)	0	-1.02(-1.11 to -0.93)	0	-1.56(-1.69 to -1.43)

East Asia	0	1.26(0.93 to 1.59)	0	-2.34(-2.61 to -2.06)	0	-2.66(-2.96 to -2.36)
Eastern Europe	0.47	0.47(-0.8 to 1.76)	0.142	-0.79(-1.83 to 0.27)	0.031	-1.28(-2.43 to -0.12)
Eastern Sub-Saharan Africa	0	-2.55(-2.66 to -2.45)	0	-2.26(-2.35 to -2.17)	0	-3.15(-3.31 to -3)
High-income Asia Pacific	0	0.66(0.38 to 0.94)	0	-4.45(-4.72 to -4.18)	0	-4.84(-5.09 to -4.6)
High-income North America	0	-1.29(-1.57 to -1.01)	0	-3.81(-4.1 to -3.52)	0	-4.31(-4.63 to -3.99)
North Africa and Middle East	0	-1.56(-1.65 to -1.48)	0	-1.91(-2.01 to -1.8)	0	-2.2(-2.28 to -2.11)
Oceania	0	-1.07(-1.15 to -1)	0	-1.06(-1.13 to -0.99)	0	-1.1(-1.29 to -0.91)
South Asia	0	-1.69(-1.91 to -1.46)	0	-1.64(-1.96 to -1.32)	0	-2.11(-2.37 to -1.84)
Southeast Asia	0	-0.56(-0.75 to -0.37)	0	-0.6(-0.81 to -0.4)	0	-1.04(-1.23 to -0.84)
Southern Latin America	0	-2.44(-3 to -1.88)	0	-3.02(-3.4 to -2.64)	0	-3.45(-3.83 to -3.06)
Southern Sub-Saharan Africa	0	-1.05(-1.45 to -0.65)	0	-1.13(-1.58 to -0.67)	0	-1.23(-1.53 to -0.93)
Tropical Latin America	0	-2.92(-3.07 to -2.77)	0	-2.86(-3.01 to -2.71)	0	-3.57(-3.72 to -3.42)
Western Europe	0	1.52(1.06 to 1.97)	0	-3.8(-4.12 to -3.47)	0	-4.04(-4.25 to -3.82)
Western Sub-Saharan Africa	0	-0.19(-0.28 to -0.11)	0.025	-0.09(-0.16 to -0.01)	0	-0.39(-0.52 to -0.25)
Countries						
Afghanistan	0	-1.22(-1.32 to -1.12)	0	-1.1(-1.18 to -1.01)	0	-1.51(-1.71 to -1.32)
Albania	0	0.9(0.53 to 1.27)	0	-1.36(-1.73 to -0.99)	0	-0.95(-1.32 to -0.58)
Algeria	0	-1.98(-2.02 to -1.93)	0	-2.16(-2.21 to -2.11)	0	-2.57(-2.68 to -2.46)
American Samoa	0	-1.73(-2.42 to -1.04)	0	-1.83(-2.53 to -1.12)	0	-1.62(-2 to -1.24)
Andorra	0	2.67(2.31 to 3.03)	0	-2.89(-2.99 to -2.78)	0	-2.54(-2.66 to -2.41)
Angola	0.001	-0.84(-1.33 to -0.36)	0.001	-0.62(-0.97 to -0.27)	0	-1.35(-1.83 to -0.87)
Antigua and Barbuda	0	-1.21(-1.52 to -0.89)	0	-1.36(-1.81 to -0.9)	0	-1.69(-2.05 to -1.32)
Argentina	0	-2.92(-3.43 to -2.4)	0	-3.12(-3.5 to -2.73)	0	-3.49(-3.83 to -3.15)
Armenia	0	-1.56(-2.02 to -1.11)	0	-1.93(-2.41 to -1.45)	0	-2.29(-2.87 to -1.7)
Australia	0.085	-0.78(-1.65 to 0.11)	0	-4.13(-4.74 to -3.51)	0	-4.29(-4.85 to -3.73)
Austria	0	1.65(1.26 to 2.03)	0	-3.56(-3.84 to -3.28)	0	-3.68(-4.04 to -3.32)
Azerbaijan	0	-0.74(-1.09 to -0.38)	0	-0.62(-0.94 to -0.3)	0	-1.39(-1.87 to -0.91)
Bahamas	0.001	-1.12(-1.77 to -0.47)	0	-1.19(-1.79 to -0.58)	0	-1.21(-1.81 to -0.61)
Bahrain	0	-2.1(-3.02 to -1.17)	0	-2.71(-3.76 to -1.65)	0	-3.05(-3.58 to -2.52)
Bangladesh	0	-3.12(-3.45 to -2.8)	0	-2.94(-3.29 to -2.6)	0	-3.63(-3.9 to -3.36)
Barbados	0.428	-0.2(-0.71 to 0.3)	0.064	-0.42(-0.86 to 0.02)	0.035	-0.66(-1.28 to -0.05)
Belarus	0.601	0.2(-0.56 to 0.97)	0	-1.75(-2.58 to -0.91)	0	-2.33(-3.13 to -1.52)
Belgium	0	2.42(1.49 to 3.35)	0	-3.15(-3.91 to -2.38)	0	-3.14(-3.9 to -2.38)
Belize	0.55	0.1(-0.24 to 0.45)	0.683	0.07(-0.27 to 0.42)	0.835	0.04(-0.37 to 0.46)
Benin	0.005	-0.28(-0.47 to -0.08)	0	-0.23(-0.35 to -0.11)	0.006	-0.29(-0.49 to -0.08)
Bermuda	0.359	0.08(-0.09 to 0.26)	0	-2.45(-2.76 to -2.14)	0	-2.57(-2.82 to -2.32)
Bhutan	0	-2.12(-2.34 to -1.91)	0	-1.84(-2.04 to -1.63)	0	-2.8(-3.14 to -2.45)
Bolivia (Plurinational State of)	0	-0.44(-0.48 to -0.4)	0	-0.25(-0.34 to -0.17)	0	-1.01(-1.05 to -0.97)
Bosnia and Herzegovina	0	0.96(0.59 to 1.32)	0.552	0.17(-0.38 to 0.72)	0.219	-0.27(-0.71 to 0.16)
Botswana	0.818	0.03(-0.19 to 0.24)	0.568	-0.06(-0.26 to 0.14)	0.342	0.13(-0.14 to 0.41)
Brazil	0	-3(-3.15 to -2.84)	0	-2.94(-3.1 to -2.78)	0	-3.65(-3.81 to -3.49)
Brunei Darussalam	0	-1.24(-1.91 to -0.55)	0	-1.41(-2 to -0.82)	0	-1.85(-2.19 to -1.51)
Bulgaria	0	-1.63(-2.25 to -1.01)	0	-2.06(-2.52 to -1.59)	0	-2.59(-3.16 to -2.01)
Burkina Faso	0.001	0.34(0.14 to 0.54)	0	0.37(0.18 to 0.56)	0.001	0.35(0.14 to 0.56)
Burundi	0	-1.98(-2.48 to -1.47)	0	-1.79(-2.22 to -1.36)	0	-2.33(-3.04 to -1.62)
Cabo Verde	0	0.96(0.5 to 1.42)	0.003	1.13(0.39 to 1.87)	0.773	0.05(-0.3 to 0.41)
Cambodia	0	-0.62(-0.71 to -0.53)	0	-0.51(-0.67 to -0.35)	0	-1.07(-1.24 to -0.91)

Cameroon	0	-0.34(-0.41 to -0.28)	0	-0.34(-0.41 to -0.28)	0	-0.3(-0.41 to -0.2)
Canada	0	1.21(0.65 to 1.77)	0	-3.78(-4.13 to -3.42)	0	-3.77(-4.12 to -3.42)
Central African Republic	0	-0.57(-0.72 to -0.42)	0	-0.55(-0.74 to -0.37)	0	-0.6(-0.75 to -0.44)
Chad	0	0.55(0.41 to 0.69)	0	0.67(0.56 to 0.78)	0	0.43(0.3 to 0.57)
Chile	0.001	-1.37(-2.16 to -0.58)	0	-2.61(-3.12 to -2.1)	0	-3.16(-3.85 to -2.47)
China	0	1.22(0.83 to 1.62)	0	-2.44(-2.74 to -2.13)	0	-2.7(-3.01 to -2.39)
Colombia	0.013	-0.94(-1.67 to -0.2)	0	-1.62(-2.35 to -0.89)	0	-2.07(-2.39 to -1.74)
Comoros	0.406	-0.55(-1.83 to 0.75)	0.524	-0.47(-1.9 to 0.98)	0.379	-0.69(-2.22 to 0.86)
Congo	0	-1.39(-1.6 to -1.18)	0	-1.29(-1.48 to -1.11)	0	-1.6(-1.91 to -1.3)
Cook Islands	0	-0.93(-1.05 to -0.81)	0	-1.42(-1.55 to -1.29)	0	-1.54(-1.64 to -1.45)
Costa Rica	0.056	-1.49(-2.98 to 0.04)	0	-2.23(-3.15 to -1.3)	0	-2.2(-3.26 to -1.12)
Croatia	0	2.62(2.09 to 3.16)	0	-1.38(-1.9 to -0.86)	0	-2(-2.32 to -1.69)
Cuba	0.021	-0.62(-1.15 to -0.09)	0	-1.62(-1.74 to -1.51)	0	-1.92(-2.24 to -1.6)
Cyprus	0	4.11(3.2 to 5.03)	0	-3.17(-4.29 to -2.03)	0	-2.49(-3.09 to -1.88)
Czechia	0.07	1.06(-0.09 to 2.22)	0	-3.11(-3.82 to -2.39)	0	-3.16(-3.6 to -2.71)
C么te d'Ivoire	0	-0.27(-0.43 to -0.12)	0.003	-0.22(-0.37 to -0.07)	0	-0.34(-0.51 to -0.18)
Democratic People's Republic of Korea	0	-1.87(-1.96 to -1.79)	0	-1.85(-1.91 to -1.78)	0	-2.07(-2.14 to -2)
Democratic Republic of the Congo	0	-1.43(-1.66 to -1.2)	0	-1.27(-1.52 to -1.01)	0	-1.84(-2.05 to -1.63)
Denmark	0.071	-0.5(-1.05 to 0.04)	0	-5.15(-5.71 to -4.59)	0	-5.29(-5.8 to -4.78)
Djibouti	0.41	0.13(-0.17 to 0.42)	0.098	0.23(-0.04 to 0.51)	0.72	-0.06(-0.4 to 0.28)
Dominica	0.104	0.15(-0.03 to 0.33)	0.088	0.17(-0.03 to 0.36)	0.571	0.05(-0.13 to 0.24)
Dominican Republic	0	1.7(0.89 to 2.51)	0	1.71(0.88 to 2.55)	0	1.42(0.63 to 2.23)
Ecuador	0.001	0.56(0.22 to 0.9)	0.001	0.59(0.25 to 0.94)	0.226	0.23(-0.14 to 0.61)
Egypt	0	-0.84(-0.91 to -0.77)	0	-0.77(-0.91 to -0.63)	0	-1.33(-1.44 to -1.21)
El Salvador	0	1.73(1.14 to 2.33)	0	1.8(1.12 to 2.49)	0.002	1.02(0.37 to 1.68)
Equatorial Guinea	0	-1.14(-1.38 to -0.9)	0	-0.91(-1.03 to -0.78)	0	-1.75(-2.03 to -1.46)
Eritrea	0.003	-0.35(-0.58 to -0.12)	0.02	-0.24(-0.44 to -0.04)	0	-0.58(-0.83 to -0.32)
Estonia	0	1.57(1.03 to 2.1)	0.006	-1.33(-2.27 to -0.39)	0	-1.88(-2.51 to -1.24)
Eswatini	0.004	0.38(0.12 to 0.64)	0.004	0.38(0.12 to 0.65)	0.002	0.38(0.13 to 0.63)
Ethiopia	0	-3.06(-3.21 to -2.92)	0	-2.78(-2.91 to -2.64)	0	-3.76(-4.03 to -3.49)
Fiji	0	-1.81(-2.45 to -1.18)	0	-1.76(-2.37 to -1.16)	0	-1.96(-2.46 to -1.46)
Finland	0.2	-0.81(-2.03 to 0.43)	0	-5.03(-5.57 to -4.49)	0	-5.04(-5.58 to -4.5)
France	0	2.79(2.24 to 3.34)	0	-3.17(-3.45 to -2.89)	0	-3.6(-3.93 to -3.26)
Gabon	0	-1.06(-1.24 to -0.87)	0	-0.96(-1.14 to -0.77)	0	-1.33(-1.53 to -1.13)
Gambia	0.403	0.26(-0.35 to 0.88)	0.301	0.29(-0.26 to 0.85)	0.623	0.18(-0.52 to 0.88)
Georgia	0.643	-0.24(-1.27 to 0.79)	0.782	-0.13(-1.09 to 0.83)	0.159	-0.75(-1.78 to 0.29)
Germany	0	1.64(1.08 to 2.2)	0	-3.7(-4.08 to -3.32)	0	-3.92(-4.17 to -3.67)
Ghana	0	-0.88(-1 to -0.77)	0	-0.79(-0.93 to -0.66)	0	-1.16(-1.34 to -0.98)
Global	0	-0.5(-0.76 to -0.24)	0	-2.18(-2.33 to -2.04)	0	-2.35(-2.47 to -2.24)
Greece	0	2.49(1.59 to 3.4)	0	-1.52(-1.86 to -1.18)	0	-1.51(-1.96 to -1.07)
Greenland	0	-2.32(-2.69 to -1.95)	0	-2.3(-2.59 to -2.01)	0	-2.68(-2.94 to -2.41)
Grenada	0	-2.76(-3.23 to -2.28)	0	-2.75(-3.22 to -2.28)	0	-2.84(-3.3 to -2.38)
Guam	0.006	-1.63(-2.78 to -0.47)	0.001	-1.97(-3.12 to -0.8)	0.07	-1.01(-2.1 to 0.08)
Guatemala	0.008	0.67(0.17 to 1.17)	0	0.84(0.47 to 1.22)	0.001	0.63(0.25 to 1.01)
Guinea	0.007	-0.12(-0.21 to -0.03)	0.05	-0.08(-0.15 to 0)	0	-0.21(-0.32 to -0.1)
Guinea-Bissau	0	-0.81(-0.87 to -0.75)	0	-0.76(-0.81 to -0.7)	0	-0.89(-0.98 to -0.8)
Guyana	0	-1.42(-1.67 to -1.17)	0	-1.42(-1.66 to -1.18)	0	-1.4(-1.73 to -1.07)
Haiti	0	-1.76(-1.87 to -1.65)	0	-1.59(-1.67 to -1.5)	0	-2.09(-2.23 to -1.95)
Honduras	0.861	0.05(-0.53 to 0.64)	0.252	0.33(-0.23 to 0.89)	0	-0.64(-0.92 to -0.35)

Chronic myeloid leukemia	Hungary	0	-1.93(-2.54 to -1.31)	0	-4.39(-5.16 to -3.61)	0	-4.81(-5.54 to -4.08)
	Iceland	0	2.24(1.75 to 2.73)	0	-2.58(-2.84 to -2.33)	0	-2.52(-2.94 to -2.09)
	India	0	-1.67(-2.01 to -1.32)	0	-1.63(-1.98 to -1.29)	0	-2.1(-2.47 to -1.72)
	Indonesia	0	-0.48(-0.54 to -0.42)	0	-0.28(-0.34 to -0.23)	0	-1.06(-1.11 to -1)
	Iran (Islamic Republic of)	0	-1.59(-1.69 to -1.49)	0	-2.01(-2.12 to -1.9)	0	-2.41(-2.55 to -2.28)
	Iraq	0	-1.77(-1.96 to -1.58)	0	-1.77(-1.94 to -1.59)	0	-2.28(-2.43 to -2.14)
	Ireland	0	2.31(1.74 to 2.88)	0	-5.21(-5.83 to -4.58)	0	-4.74(-5.46 to -4.02)
	Israel	0	0.91(0.57 to 1.24)	0	-3.64(-3.89 to -3.4)	0	-3.82(-4.19 to -3.44)
	Italy	0	1.54(0.93 to 2.16)	0	-3.94(-4.17 to -3.71)	0	-4.26(-4.54 to -3.99)
	Jamaica	0.001	2.74(1.05 to 4.45)	0	2.92(1.42 to 4.44)	0	3.15(1.52 to 4.82)
	Japan	0.404	0.28(-0.38 to 0.95)	0	-4.91(-5.35 to -4.47)	0	-5.35(-5.8 to -4.9)
	Jordan	0	-1.31(-1.67 to -0.95)	0	-1.77(-2.09 to -1.45)	0	-2.1(-2.38 to -1.81)
	Kazakhstan	0	-1.95(-2.4 to -1.5)	0	-2.12(-2.59 to -1.65)	0	-2.75(-3.27 to -2.22)
	Kenya	0	-1.14(-1.35 to -0.94)	0	-0.68(-0.89 to -0.47)	0	-1.5(-1.81 to -1.19)
	Kiribati	0	-0.22(-0.28 to -0.16)	0	-0.19(-0.26 to -0.13)	0	-0.28(-0.35 to -0.21)
	Kuwait	0.066	1.51(-0.1 to 3.16)	0.258	-0.72(-1.97 to 0.53)	0.045	-1.18(-2.31 to -0.03)
	Kyrgyzstan	0	-2.74(-3.68 to -1.8)	0	-2.53(-3.52 to -1.53)	0	-3.47(-4.37 to -2.56)
	Lao People's Democratic Republic	0	-2.14(-2.2 to -2.09)	0	-1.96(-2.06 to -1.87)	0	-2.56(-2.64 to -2.48)
	Latvia	0.227	-0.7(-1.82 to 0.44)	0.008	-2.14(-3.7 to -0.55)	0.002	-2.36(-3.85 to -0.85)
	Lebanon	0	0.63(0.51 to 0.75)	0	-1.76(-1.84 to -1.68)	0	-1.84(-1.95 to -1.74)
	Lesotho	0	2.23(2.09 to 2.37)	0	2.22(2.05 to 2.39)	0	2.23(2.09 to 2.37)
	Liberia	0	-0.73(-0.95 to -0.5)	0	-0.6(-0.88 to -0.32)	0	-1.02(-1.28 to -0.76)
	Libya	0	-1.46(-1.93 to -0.98)	0	-1.56(-2.04 to -1.09)	0	-1.74(-2.3 to -1.18)
	Lithuania	0	-2.66(-3.59 to -1.72)	0	-3.68(-4.55 to -2.81)	0	-3.97(-5.75 to -2.15)
	Luxembourg	0	2.51(1.95 to 3.07)	0	-3.98(-4.24 to -3.71)	0	-3.84(-4.12 to -3.57)
	Madagascar	0	-1.31(-1.56 to -1.05)	0	-1.08(-1.31 to -0.86)	0	-1.76(-2.12 to -1.4)
	Malawi	0	-2.26(-2.63 to -1.89)	0	-1.95(-2.27 to -1.64)	0	-2.86(-3.39 to -2.33)
	Malaysia	0.018	-0.82(-1.5 to -0.14)	0.047	-1.03(-2.04 to -0.01)	0	-1.24(-1.83 to -0.64)
	Maldives	0	-2.83(-3.25 to -2.42)	0	-3.43(-3.75 to -3.12)	0	-4.29(-4.49 to -4.09)
	Mali	0	-0.58(-0.9 to -0.26)	0.002	-0.48(-0.79 to -0.18)	0	-0.75(-1.11 to -0.38)
	Malta	0	1.18(0.84 to 1.53)	0	-4.02(-4.21 to -3.84)	0	-3.64(-3.85 to -3.43)
	Marshall Islands	0	-0.92(-0.98 to -0.86)	0	-0.95(-1.02 to -0.89)	0	-0.82(-0.87 to -0.76)
	Mauritania	0	-1.04(-1.17 to -0.91)	0	-0.9(-1.02 to -0.79)	0	-1.25(-1.41 to -1.1)
	Mauritius	0.032	1.34(0.12 to 2.58)	0.066	1.17(-0.08 to 2.44)	0.255	0.72(-0.51 to 1.96)
	Mexico	0	-1.22(-1.68 to -0.75)	0	-1.31(-1.55 to -1.06)	0	-1.59(-2.07 to -1.1)
	Micronesia (Federated States of)	0	-1.29(-1.35 to -1.23)	0	-1.27(-1.34 to -1.21)	0	-1.41(-1.5 to -1.32)
	Monaco	0	3.02(2.67 to 3.37)	0	-1.56(-1.66 to -1.46)	0	-1.42(-1.55 to -1.28)
	Mongolia	0	-0.51(-0.76 to -0.27)	0.005	-0.37(-0.63 to -0.11)	0	-0.85(-1.21 to -0.48)
	Montenegro	0.001	0.78(0.33 to 1.22)	0	-1.08(-1.34 to -0.82)	0	-1.47(-1.83 to -1.1)
	Morocco	0	-1.06(-1.33 to -0.79)	0	-0.9(-1.17 to -0.63)	0	-1.5(-1.73 to -1.27)
	Mozambique	0.948	0.01(-0.19 to 0.2)	0.003	0.23(0.08 to 0.38)	0.002	-0.45(-0.72 to -0.17)
	Myanmar	0	-2.55(-2.62 to -2.48)	0	-2.43(-2.5 to -2.36)	0	-2.92(-3 to -2.83)
	Namibia	0.175	0.16(-0.07 to 0.4)	0.1	0.18(-0.03 to 0.4)	0.666	0.06(-0.22 to 0.35)
	Nauru	0	-1.11(-1.18 to -1.04)	0	-1.17(-1.23 to -1.11)	0	-1.1(-1.2 to -1.01)
	Nepal	0	-1.88(-1.93 to -1.84)	0	-1.61(-1.68 to -1.55)	0	-2.61(-2.69 to -2.53)
	Netherlands	0.09	0.76(-0.12 to 1.65)	0	-4.83(-5.53 to -4.12)	0	-4.67(-5.34 to -4)
	New Zealand	0	-0.91(-1.34 to -0.49)	0	-4.45(-4.83 to -4.06)	0	-4.44(-4.79 to -4.08)
	Nicaragua	0.689	0.22(-0.87 to 1.33)	0.733	0.19(-0.88 to 1.26)	0.221	-0.65(-1.67 to 0.39)
	Niger	0	-0.54(-0.79 to -0.28)	0.002	-0.38(-0.62 to -0.15)	0	-0.75(-1.07 to -0.42)
	Nigeria	0.951	0(-0.1 to 0.1)	0.035	0.11(0.01 to 0.22)	0	-0.29(-0.42 to -0.15)
	Niue	0	-1.3(-1.47 to -1.14)	0	-1.54(-1.67 to -1.4)	0	-1.66(-1.81 to -1.51)

North Macedonia	0.146	-0.24(-0.56 to 0.08)	0	-1.53(-1.85 to -1.21)	0	-1.72(-2.06 to -1.37)
Northern Mariana Islands	0	-3.21(-4.29 to -2.12)	0	-3.52(-4.21 to -2.83)	0	-3.44(-4.08 to -2.79)
Norway	0	0.63(0.36 to 0.9)	0	-4.45(-4.89 to -4)	0	-4.6(-4.96 to -4.24)
Oman	0	-1.04(-1.3 to -0.77)	0	-1.98(-2.35 to -1.62)	0	-2.54(-2.82 to -2.25)
Pakistan	0	-0.77(-0.89 to -0.64)	0	-0.77(-0.88 to -0.67)	0	-0.92(-1.08 to -0.77)
Palau	0	-0.52(-0.59 to -0.44)	0	-0.67(-0.74 to -0.6)	0	-0.83(-1 to -0.66)
Palestine	0	-0.48(-0.66 to -0.3)	0	-0.56(-0.74 to -0.39)	0	-1.03(-1.36 to -0.71)
Panama	0.034	-1.02(-1.95 to -0.08)	0.004	-1.34(-2.25 to -0.42)	0	-1.52(-2.35 to -0.69)
Papua New Guinea	0	-0.8(-1.12 to -0.49)	0	-0.75(-1.01 to -0.49)	0	-0.88(-1.23 to -0.52)
Paraguay	0.005	0.93(0.28 to 1.58)	0.005	0.92(0.27 to 1.57)	0.134	0.49(-0.15 to 1.14)
Peru	0.386	-0.49(-1.58 to 0.62)	0.352	-0.54(-1.65 to 0.59)	0.057	-1.02(-2.05 to 0.03)
Philippines	0	-1.59(-1.79 to -1.39)	0	-1.7(-1.91 to -1.5)	0	-1.57(-1.75 to -1.39)
Poland	0	-1.15(-1.46 to -0.84)	0	-3.18(-3.62 to -2.74)	0	-3.94(-4.44 to -3.43)
Portugal	0	1.7(1.24 to 2.16)	0	-4.05(-4.5 to -3.6)	0	-4.18(-4.77 to -3.59)
Puerto Rico	0.057	-1.46(-2.93 to 0.05)	0	-3.5(-4.68 to -2.3)	0	-3.6(-4.82 to -2.36)
Qatar	0.371	0.95(-1.12 to 3.07)	0.667	-0.41(-2.23 to 1.46)	0.001	-1.79(-2.88 to -0.7)
Republic of Korea	0	3.16(2.52 to 3.8)	0	-2.98(-3.21 to -2.74)	0	-3.69(-4.02 to -3.36)
Republic of Moldova	0.008	-2.57(-4.42 to -0.68)	0	-2.82(-4.33 to -1.29)	0.001	-3(-4.76 to -1.21)
Romania	0.006	-0.67(-1.15 to -0.2)	0	-1.92(-2.4 to -1.43)	0	-2.65(-3.15 to -2.16)
Russian Federation	0.301	0.91(-0.81 to 2.66)	0.788	-0.19(-1.55 to 1.19)	0.269	-0.85(-2.34 to 0.66)
Rwanda	0	-1.77(-2.12 to -1.41)	0	-1.63(-2.09 to -1.16)	0	-1.96(-2.54 to -1.37)
Saint Kitts and Nevis	0	-1.58(-1.85 to -1.3)	0	-1.84(-2.14 to -1.54)	0	-2.09(-2.45 to -1.73)
Saint Lucia	0	-1.95(-2.17 to -1.74)	0	-2.05(-2.32 to -1.79)	0	-2.12(-2.26 to -1.98)
Saint Vincent and the Grenadines	0	-3.64(-4.02 to -3.25)	0	-3.71(-4.08 to -3.33)	0	-3.84(-4.35 to -3.34)
Samoa	0	-0.89(-0.93 to -0.84)	0	-0.93(-0.98 to -0.88)	0	-0.9(-0.93 to -0.86)
San Marino	0	2.95(2.42 to 3.48)	0	-1.9(-2.12 to -1.69)	0	-1.67(-1.9 to -1.44)
Sao Tome and Principe	0.004	0.43(0.14 to 0.73)	0	0.47(0.21 to 0.73)	0.281	0.21(-0.17 to 0.6)
Saudi Arabia	0.001	0.15(0.06 to 0.23)	0	-1.31(-1.37 to -1.24)	0	-1.1(-1.19 to -1.02)
Senegal	0.096	-0.49(-1.06 to 0.09)	0.096	-0.41(-0.89 to 0.07)	0.092	-0.63(-1.37 to 0.1)
Serbia	0	1.57(1.3 to 1.84)	0.967	-0.01(-0.3 to 0.28)	0.07	-0.54(-1.11 to 0.04)
Seychelles	0	-1.1(-1.46 to -0.74)	0	-1.22(-1.6 to -0.84)	0	-1.39(-1.65 to -1.14)
Sierra Leone	0.074	-0.12(-0.26 to 0.01)	0.062	-0.13(-0.26 to 0.01)	0.133	-0.13(-0.29 to 0.04)
Singapore	0.235	0.93(-0.6 to 2.48)	0	-4.88(-5.49 to -4.27)	0	-4.95(-5.56 to -4.34)
Slovakia	0	-1.57(-2.16 to -0.98)	0	-4.1(-4.58 to -3.62)	0	-4.31(-4.8 to -3.82)
Slovenia	0	2.64(2.36 to 2.91)	0	-2.92(-3.21 to -2.63)	0	-3.3(-3.72 to -2.87)
Solomon Islands	0	-0.56(-0.77 to -0.35)	0	-0.56(-0.82 to -0.29)	0	-0.57(-0.75 to -0.39)
Somalia	0	-0.58(-0.71 to -0.46)	0	-0.43(-0.53 to -0.33)	0	-0.72(-0.86 to -0.58)
South Africa	0	-2.09(-2.68 to -1.5)	0	-2.03(-2.66 to -1.4)	0	-2.31(-2.71 to -1.9)
South Sudan	0	-0.89(-1.07 to -0.71)	0	-0.75(-0.92 to -0.58)	0	-1.28(-1.5 to -1.06)
Spain	0	2.28(1.88 to 2.67)	0	-4.2(-4.46 to -3.93)	0	-4.45(-4.83 to -4.08)
Sri Lanka	0.003	-1.01(-1.68 to -0.35)	0	-1.45(-2.03 to -0.87)	0	-1.77(-2.51 to -1.03)
Sudan	0	-1.53(-1.61 to -1.46)	0	-1.38(-1.44 to -1.33)	0	-2.15(-2.32 to -1.99)
Suriname	0.028	-0.78(-1.47 to -0.09)	0.017	-0.77(-1.39 to -0.14)	0.055	-0.88(-1.77 to 0.02)
Sweden	0.611	0.25(-0.7 to 1.2)	0	-4.51(-5.26 to -3.76)	0	-4.6(-5.38 to -3.81)
Switzerland	0.162	-0.34(-0.82 to 0.14)	0	-5.07(-5.31 to -4.84)	0	-5.31(-5.69 to -4.92)
Syrian Arab Republic	0	-2.36(-2.68 to -2.03)	0	-2.46(-2.69 to -2.23)	0	-2.82(-3.17 to -2.48)
Taiwan (Province of China)	0	2.54(1.9 to 3.18)	0	-1.01(-1.46 to -0.56)	0	-1.72(-2.21 to -1.24)

Tajikistan	0	-2.6(-2.94 to -2.26)	0	-2.36(-2.77 to -1.94)	0	-3.27(-3.48 to -3.05)
Thailand	0	1.51(0.74 to 2.28)	0.007	1.03(0.28 to 1.79)	0	1.05(0.48 to 1.63)
Timor-Leste	0	-1.16(-1.5 to -0.83)	0	-0.97(-1.26 to -0.67)	0	-1.63(-1.97 to -1.29)
Togo	0	-0.53(-0.74 to -0.32)	0	-0.5(-0.71 to -0.28)	0	-0.58(-0.82 to -0.33)
Tokelau	0	-1.85(-2 to -1.7)	0	-1.87(-2.02 to -1.72)	0	-1.95(-2.07 to -1.83)
Tonga	0	-0.86(-1.06 to -0.65)	0	-0.86(-1.08 to -0.65)	0	-0.89(-1.07 to -0.71)
Trinidad and Tobago	0	-2.29(-2.95 to -1.63)	0	-2.36(-3.02 to -1.69)	0	-2.35(-3.01 to -1.68)
Tunisia	0	-0.62(-0.76 to -0.48)	0	-1.3(-1.4 to -1.21)	0	-1.99(-2.05 to -1.93)
Turkey	0	-3.49(-3.89 to -3.1)	0	-4(-4.67 to -3.33)	0	-4.96(-5.38 to -4.54)
Turkmenistan	0.008	-1.26(-2.18 to -0.33)	0.008	-1.23(-2.13 to -0.32)	0	-1.86(-2.74 to -0.97)
Tuvalu	0	-1.87(-1.98 to -1.75)	0	-1.84(-1.95 to -1.72)	0	-1.97(-2.08 to -1.87)
Uganda	0	-1.52(-1.97 to -1.08)	0	-1.46(-1.84 to -1.07)	0	-1.69(-2.2 to -1.19)
Ukraine	0.635	-0.27(-1.37 to 0.84)	0.003	-1.39(-2.3 to -0.47)	0.006	-1.61(-2.75 to -0.46)
United Arab Emirates	0	-2.69(-3.31 to -2.06)	0	-2.78(-3.5 to -2.06)	0	-2.77(-3.23 to -2.31)
United Kingdom	0.343	0.21(-0.23 to 0.66)	0	-4.22(-4.6 to -3.84)	0	-4.3(-4.63 to -3.97)
United Republic of Tanzania	0.002	-0.33(-0.53 to -0.12)	0	-0.21(-0.32 to -0.1)	0.001	-0.54(-0.87 to -0.22)
United States of America	0	-1.73(-2.04 to -1.42)	0	-3.81(-4.11 to -3.51)	0	-4.36(-4.71 to -4.02)
United States Virgin Islands	0	-1.56(-1.82 to -1.3)	0	-1.7(-1.95 to -1.46)	0	-2.2(-2.6 to -1.79)
Uruguay	0	-2.22(-3.03 to -1.4)	0	-2.65(-3.4 to -1.9)	0	-2.91(-3.61 to -2.2)
Uzbekistan	0.008	-0.42(-0.72 to -0.11)	0.277	-0.27(-0.74 to 0.21)	0	-1(-1.49 to -0.5)
Vanuatu	0	-0.66(-0.93 to -0.39)	0	-0.68(-0.94 to -0.42)	0.001	-0.59(-0.92 to -0.25)
Venezuela (Bolivarian Republic of)	0	-2.18(-2.84 to -1.52)	0	-2.3(-2.86 to -1.73)	0	-2.5(-3.15 to -1.84)
Viet Nam	0	0.17(0.09 to 0.25)	0.037	0.07(0 to 0.14)	0	-0.24(-0.34 to -0.13)
Yemen	0	-1.03(-1.15 to -0.91)	0	-0.95(-1.05 to -0.84)	0	-1.24(-1.42 to -1.06)
Zambia	0	-1.02(-1.17 to -0.88)	0	-0.81(-0.95 to -0.66)	0	-1.49(-1.65 to -1.34)
Zimbabwe	0.027	0.31(0.04 to 0.59)	0.155	0.23(-0.09 to 0.54)	0.014	0.54(0.11 to 0.98)
Global	0	-2.21(-2.26 to -2.16)	0	-1.9(-1.98 to -1.82)	0	-2.77(-2.87 to -2.67)
SDI regions						
High SDI	0	-1.76(-1.85 to -1.67)	0	-1.31(-1.37 to -1.26)	0	-2.2(-2.28 to -2.12)
High-middle SDI	0	-2.44(-2.53 to -2.35)	0	-2.22(-2.36 to -2.09)	0	-3.23(-3.33 to -3.12)
Middle SDI	0	-2.46(-2.6 to -2.31)	0	-2.21(-2.29 to -2.13)	0	-3.13(-3.19 to -3.07)
Low-middle SDI	0	-2.1(-2.23 to -1.96)	0	-1.83(-1.89 to -1.77)	0	-2.63(-2.73 to -2.53)
Low SDI	0	-1.26(-1.34 to -1.17)	0	-1.08(-1.1 to -1.06)	0	-1.53(-1.61 to -1.45)
Regions						
Andean Latin America	0	-1.17(-1.58 to -0.77)	0	-1.19(-1.73 to -0.65)	0	-1.79(-2.24 to -1.34)
Australasia	0.868	-0.03(-0.32 to 0.27)	0.097	0.38(-0.07 to 0.83)	0.481	-0.1(-0.38 to 0.18)
Caribbean	0	-0.81(-0.92 to -0.69)	0	-0.61(-0.78 to -0.45)	0	-0.99(-1.12 to -0.86)
Central Asia	0	-2.4(-2.62 to -2.17)	0	-1.65(-1.88 to -1.41)	0	-2.6(-2.79 to -2.4)
Central Europe	0	-2(-2.26 to -1.74)	0	-1.73(-1.96 to -1.5)	0	-2.45(-2.65 to -2.25)
Central Latin America	0	-2.01(-2.2 to -1.82)	0	-1.78(-1.98 to -1.58)	0	-2.39(-2.55 to -2.23)
Central Sub-Saharan Africa	0	-2.05(-2.15 to -1.94)	0	-1.73(-1.87 to -1.59)	0	-2.16(-2.29 to -2.04)
East Asia	0	-2.66(-2.83 to -2.49)	0	-2.86(-3.04 to -2.68)	0	-3.7(-3.86 to -3.55)
Eastern Europe	0	-2.1(-3.24 to -0.95)	0.004	-1.41(-2.36 to -0.45)	0	-2.23(-3.42 to -1.03)
Eastern Sub-Saharan Africa	0	-1.62(-1.76 to -1.47)	0	-1.43(-1.56 to -1.31)	0	-1.99(-2.17 to -1.82)
High-income Asia Pacific	0	-2.96(-3.15 to -2.78)	0	-2.34(-2.51 to -2.17)	0	-3.69(-3.89 to -3.48)
High-income North America	0	-1.43(-1.59 to -1.26)	0	-0.97(-1.14 to -0.8)	0	-1.63(-1.87 to -1.39)

North Africa and Middle East	0	-1.35(-1.46 to -1.25)	0	-1.08(-1.18 to -0.99)	0	-1.7(-1.77 to -1.62)
Oceania	0.406	-0.1(-0.35 to 0.14)	0.002	-0.22(-0.37 to -0.08)	0.011	-0.33(-0.58 to -0.08)
South Asia	0	-2.1(-2.24 to -1.95)	0	-2.05(-2.28 to -1.82)	0	-2.63(-2.83 to -2.43)
Southeast Asia	0	-1.48(-1.53 to -1.43)	0	-1.29(-1.34 to -1.24)	0	-1.91(-1.95 to -1.87)
Southern Latin America	0	-0.82(-1.03 to -0.6)	0	-0.68(-0.84 to -0.52)	0	-1.24(-1.41 to -1.08)
Southern Sub-Saharan Africa	0	-0.9(-1.1 to -0.7)	0	-0.79(-1.04 to -0.54)	0	-1.36(-1.74 to -0.97)
Tropical Latin America	0	-1.93(-2.06 to -1.81)	0	-1.29(-1.43 to -1.15)	0	-2.17(-2.27 to -2.06)
Western Europe	0	-1.79(-2.09 to -1.5)	0	-1.24(-1.42 to -1.07)	0	-2.29(-2.43 to -2.14)
Western Sub-Saharan Africa	0	-0.22(-0.31 to -0.12)	0	-0.3(-0.39 to -0.2)	0	-0.44(-0.55 to -0.34)
Countries						
Afghanistan	0	-0.78(-0.99 to -0.58)	0	-0.43(-0.56 to -0.31)	0	-1.03(-1.2 to -0.86)
Albania	0.456	-0.33(-1.19 to 0.54)	0	-1.5(-2.19 to -0.81)	0	-1.6(-2.32 to -0.88)
Algeria	0	-2.02(-2.1 to -1.95)	0	-1.89(-1.93 to -1.85)	0	-2.52(-2.59 to -2.45)
American Samoa	0.91	0.02(-0.27 to 0.31)	0.85	-0.02(-0.25 to 0.21)	0.992	0(-0.3 to 0.3)
Andorra	0	-1.57(-1.66 to -1.47)	0	-1.13(-1.24 to -1.02)	0	-1.52(-1.6 to -1.43)
Angola	0	-2.31(-2.52 to -2.09)	0	-2.01(-2.41 to -1.61)	0	-2.67(-2.95 to -2.4)
Antigua and Barbuda	0.006	-0.26(-0.44 to -0.07)	0.159	-0.27(-0.64 to 0.11)	0	-0.71(-0.81 to -0.62)
Argentina	0	-0.78(-1.03 to -0.53)	0	-0.49(-0.74 to -0.24)	0	-1.13(-1.32 to -0.94)
Armenia	0	-1.62(-2.36 to -0.87)	0	-1.49(-2.03 to -0.95)	0	-1.97(-2.75 to -1.19)
Australia	0.036	0.37(0.02 to 0.71)	0.071	0.65(-0.05 to 1.37)	0.653	0.08(-0.26 to 0.41)
Austria	0	-2.03(-2.63 to -1.42)	0	-1.75(-2.02 to -1.47)	0	-2.45(-2.84 to -2.05)
Azerbaijan	0.001	-1.2(-1.9 to -0.51)	0	-1.03(-1.44 to -0.62)	0	-1.66(-2.36 to -0.95)
Bahamas	0	-0.92(-1.2 to -0.65)	0	-0.94(-1.17 to -0.72)	0	-1.09(-1.36 to -0.82)
Bahrain	0.142	-0.52(-1.22 to 0.18)	0.011	-0.7(-1.23 to -0.16)	0	-1.14(-1.65 to -0.63)
Bangladesh	0	-3.25(-3.43 to -3.06)	0	-2.78(-3.18 to -2.38)	0	-3.72(-4.01 to -3.44)
Barbados	0	-0.89(-1.29 to -0.49)	0	-0.67(-0.92 to -0.43)	0	-1.19(-1.58 to -0.8)
Belarus	0	-4.06(-4.52 to -3.6)	0	-2.83(-3.12 to -2.55)	0	-4.1(-4.73 to -3.47)
Belgium	0	-2.47(-2.98 to -1.96)	0	-2.19(-2.86 to -1.52)	0	-2.85(-3.31 to -2.4)
Belize	0	-0.87(-1.33 to -0.4)	0.137	-0.21(-0.48 to 0.07)	0.006	-0.6(-1.02 to -0.17)
Benin	0.128	0.14(-0.04 to 0.32)	0.527	0.04(-0.08 to 0.16)	0.918	-0.01(-0.18 to 0.16)
Bermuda	0	-2.25(-2.48 to -2.02)	0	-2.68(-2.9 to -2.46)	0	-2.94(-3.16 to -2.72)
Bhutan	0	-1.82(-2.15 to -1.49)	0	-1.4(-1.53 to -1.27)	0	-2.59(-2.93 to -2.26)
Bolivia (Plurinational State of)	0	-1.37(-1.51 to -1.23)	0	-1.08(-1.13 to -1.03)	0	-1.95(-2.02 to -1.88)
Bosnia and Herzegovina	0	-1.71(-2.3 to -1.11)	0	-1.92(-2.37 to -1.48)	0	-2.21(-2.76 to -1.66)
Botswana	0.069	0.24(-0.02 to 0.49)	0	-0.45(-0.67 to -0.24)	0.004	-0.28(-0.46 to -0.09)
Brazil	0	-1.92(-2.05 to -1.79)	0	-1.27(-1.44 to -1.1)	0	-2.16(-2.26 to -2.06)
Brunei Darussalam	0.004	-0.5(-0.84 to -0.16)	0	-0.63(-0.94 to -0.31)	0	-0.99(-1.28 to -0.7)
Bulgaria	0.098	-0.81(-1.77 to 0.15)	0.89	0.05(-0.71 to 0.82)	0.038	-0.91(-1.76 to -0.05)
Burkina Faso	0	0.83(0.62 to 1.04)	0	0.4(0.24 to 0.57)	0	0.63(0.43 to 0.83)
Burundi	0	-0.92(-1.31 to -0.53)	0.028	-0.62(-1.17 to -0.07)	0	-1.26(-1.71 to -0.81)
Cabo Verde	0.693	0.08(-0.32 to 0.48)	0.034	0.49(0.04 to 0.94)	0.047	-0.36(-0.72 to -0.01)
Cambodia	0	-1.75(-1.94 to -1.56)	0	-1.58(-1.67 to -1.5)	0	-2.28(-2.46 to -2.09)
Cameroon	0	0.42(0.26 to 0.58)	0.058	0.1(0 to 0.21)	0.006	0.21(0.06 to 0.36)
Canada	0	-1.81(-2.19 to -1.43)	0	-1.74(-1.85 to -1.63)	0	-2.26(-2.56 to -1.95)
Central African Republic	0	-0.93(-1.05 to -0.8)	0	-0.97(-1.14 to -0.81)	0	-0.97(-1.21 to -0.72)
Chad	0	0.88(0.77 to 1)	0	0.8(0.69 to 0.9)	0	0.8(0.66 to 0.95)
Chile	0	-1(-1.37 to -0.62)	0	-0.99(-1.19 to -0.79)	0	-1.7(-1.94 to -1.47)

	China	0	-2.66(-2.83 to -2.48)	0	-2.9(-3.09 to -2.72)	0	-3.75(-3.91 to -3.59)
	Colombia	0	-2.29(-2.64 to -1.94)	0	-2.62(-2.98 to -2.26)	0	-3.04(-3.38 to -2.7)
	Comoros	0.049	-0.9(-1.79 to 0)	0.037	-0.97(-1.87 to -0.06)	0.015	-1.35(-2.42 to -0.26)
	Congo	0	-2.16(-2.53 to -1.8)	0	-2.07(-2.32 to -1.82)	0	-2.4(-2.84 to -1.96)
	Cook Islands	0	-1.87(-1.94 to -1.79)	0	-1.57(-1.63 to -1.51)	0	-2.02(-2.07 to -1.98)
	Costa Rica	0	-1.23(-1.9 to -0.56)	0	-1.22(-1.74 to -0.69)	0	-1.34(-1.94 to -0.73)
	Croatia	0	-4.34(-4.63 to -4.05)	0	-3.9(-4.23 to -3.56)	0	-4.84(-5.16 to -4.51)
	Cuba	0	-1.92(-2.1 to -1.73)	0	-1.66(-1.83 to -1.48)	0	-2.11(-2.52 to -1.7)
	Cyprus	0	-1.28(-1.63 to -0.93)	0	-1.17(-1.67 to -0.67)	0	-1.76(-2.04 to -1.47)
	Czechia	0	-1.69(-2.25 to -1.13)	0	-1.49(-2.06 to -0.91)	0	-2.08(-2.52 to -1.64)
	C么te d'Ivoire	0.666	0.03(-0.11 to 0.18)	0.593	-0.04(-0.19 to 0.11)	0.575	-0.06(-0.28 to 0.15)
	Democratic People's Republic of Korea	0	-2.68(-2.8 to -2.55)	0	-1.53(-1.62 to -1.45)	0	-2.16(-2.22 to -2.1)
	Democratic Republic of the Congo	0	-2.08(-2.22 to -1.93)	0	-1.71(-1.83 to -1.59)	0	-2.11(-2.29 to -1.92)
	Denmark	0	-1.62(-1.98 to -1.27)	0	-1.1(-1.44 to -0.77)	0	-2.16(-2.5 to -1.83)
	Djibouti	0.332	-0.17(-0.51 to 0.17)	0	-0.33(-0.48 to -0.18)	0	-0.66(-0.92 to -0.4)
	Dominica	0.001	-0.37(-0.6 to -0.14)	0	-0.55(-0.71 to -0.4)	0	-0.6(-0.78 to -0.41)
	Dominican Republic	0	-0.91(-1.32 to -0.49)	0.16	-0.43(-1.03 to 0.17)	0	-1.11(-1.58 to -0.64)
	Ecuador	0	-1.38(-2.01 to -0.76)	0	-1.5(-1.93 to -1.06)	0	-1.99(-2.63 to -1.35)
	Egypt	0	-0.79(-1.17 to -0.42)	0.009	-0.45(-0.79 to -0.11)	0	-1.22(-1.58 to -0.85)
	El Salvador	0	-1.5(-2.03 to -0.97)	0.003	-1.08(-1.8 to -0.36)	0	-1.98(-2.6 to -1.36)
	Equatorial Guinea	0	-3.09(-3.66 to -2.52)	0	-2.99(-3.22 to -2.77)	0	-3.79(-4.28 to -3.29)
	Eritrea	0	-0.5(-0.74 to -0.26)	0	-0.6(-0.79 to -0.41)	0	-0.97(-1.16 to -0.78)
	Estonia	0	-3.74(-4.81 to -2.65)	0	-2.77(-3.93 to -1.6)	0	-3.85(-5.02 to -2.67)
	Eswatini	0.002	-0.26(-0.42 to -0.09)	0	-0.46(-0.62 to -0.31)	0	-0.34(-0.5 to -0.17)
	Ethiopia	0	-3.02(-3.24 to -2.79)	0	-2.65(-2.76 to -2.54)	0	-3.75(-3.99 to -3.5)
	Fiji	0	-1.02(-1.29 to -0.75)	0	-1.28(-1.55 to -1.02)	0	-1.37(-1.78 to -0.96)
	Finland	0	-0.78(-1.08 to -0.48)	0	-0.79(-1.21 to -0.37)	0	-1.37(-1.7 to -1.04)
	France	0	-1.62(-1.94 to -1.29)	0	-1.17(-1.45 to -0.88)	0	-2.01(-2.22 to -1.8)
	Gabon	0	-2.01(-2.22 to -1.8)	0	-1.85(-1.97 to -1.73)	0	-2.21(-2.44 to -1.99)
	Gambia	0.719	-0.15(-0.98 to 0.68)	0.808	0.06(-0.45 to 0.58)	0.599	-0.25(-1.15 to 0.67)
	Georgia	0	-2.21(-3.02 to -1.39)	0.003	-1.24(-2.06 to -0.41)	0	-2.13(-3 to -1.26)
	Germany	0	-1.37(-1.74 to -0.99)	0.009	-0.72(-1.25 to -0.18)	0	-1.77(-2.01 to -1.53)
	Ghana	0	-1.18(-1.27 to -1.08)	0	-1.18(-1.29 to -1.06)	0	-1.48(-1.57 to -1.4)
	Global	0	-2.21(-2.26 to -2.16)	0	-1.9(-1.98 to -1.82)	0	-2.77(-2.87 to -2.67)
	Greece	0	-1.47(-1.98 to -0.96)	0	-1.23(-1.8 to -0.66)	0	-1.78(-2.22 to -1.34)
	Greenland	0	-0.99(-1.29 to -0.69)	0	-0.96(-1.13 to -0.79)	0	-1.27(-1.52 to -1.03)
	Grenada	0	-0.91(-1.1 to -0.72)	0.001	-0.61(-0.98 to -0.24)	0	-1.31(-1.46 to -1.17)
	Guam	0.446	0.24(-0.37 to 0.85)	0.573	-0.28(-1.25 to 0.7)	0.141	0.33(-0.11 to 0.77)
	Guatemala	0	-0.9(-1.3 to -0.5)	0.035	-0.76(-1.46 to -0.05)	0	-1.38(-1.81 to -0.95)
	Guinea	0.025	-0.23(-0.43 to -0.03)	0.001	-0.17(-0.28 to -0.07)	0	-0.32(-0.46 to -0.18)
	Guinea-Bissau	0	-0.35(-0.54 to -0.17)	0	-0.3(-0.39 to -0.21)	0.001	-0.57(-0.9 to -0.23)
	Guyana	0	-0.62(-0.94 to -0.29)	0.001	-0.66(-1.04 to -0.29)	0	-0.81(-1.2 to -0.42)
	Haiti	0	-1.57(-1.71 to -1.42)	0	-1.28(-1.39 to -1.17)	0	-1.86(-2.02 to -1.71)
	Honduras	0	-1.51(-1.74 to -1.28)	0	-0.71(-0.96 to -0.47)	0	-2.08(-2.15 to -2.02)
	Hungary	0	-1.26(-1.67 to -0.85)	0	-1.19(-1.62 to -0.77)	0	-1.77(-2.2 to -1.33)
	Iceland	0.639	0.08(-0.26 to 0.43)	0.01	0.61(0.15 to 1.07)	0.07	-0.34(-0.7 to 0.03)
	India	0	-2.35(-2.6 to -2.09)	0	-2.16(-2.53 to -1.8)	0	-2.86(-3.17 to -2.54)
	Indonesia	0	-1.56(-1.63 to -1.48)	0	-0.97(-1.01 to -0.92)	0	-1.88(-1.95 to -1.81)
	Iran (Islamic Republic of)	0	-1.9(-2.13 to -1.66)	0	-1.23(-1.37 to -1.09)	0	-2.15(-2.32 to -1.98)
Other	Iraq	0	-0.89(-1.19 to -0.59)	0	-0.61(-0.9 to -0.32)	0	-1.3(-1.6 to -1)

leukemia	Ireland	0	-2.43(-2.95 to -1.89)	0	-2.49(-2.83 to -2.14)	0	-2.84(-3.37 to -2.31)
	Israel	0.032	-0.93(-1.78 to -0.08)	0.022	-0.65(-1.2 to -0.09)	0.002	-1.32(-2.16 to -0.48)
	Italy	0	-2.94(-3.41 to -2.46)	0	-2.22(-2.52 to -1.92)	0	-3.21(-3.58 to -2.84)
	Jamaica	0.236	0.66(-0.43 to 1.77)	0.096	1.17(-0.21 to 2.57)	0.112	1(-0.23 to 2.26)
	Japan	0	-2.32(-2.45 to -2.18)	0	-1.64(-1.83 to -1.45)	0	-2.57(-2.86 to -2.28)
	Jordan	0	-0.67(-0.85 to -0.48)	0	-0.98(-1.35 to -0.61)	0	-1.43(-1.6 to -1.25)
	Kazakhstan	0	-2.93(-3.16 to -2.7)	0	-1.69(-1.91 to -1.46)	0	-3.17(-3.56 to -2.77)
	Kenya	0	-1.22(-1.37 to -1.07)	0	-0.72(-0.84 to -0.59)	0	-1.24(-1.36 to -1.13)
	Kiribati	0	-0.64(-0.88 to -0.4)	0	-0.57(-0.72 to -0.42)	0	-0.77(-1 to -0.54)
	Kuwait	0	-2.75(-4.14 to -1.35)	0	-2.41(-3.59 to -1.22)	0	-3.36(-4.74 to -1.97)
	Kyrgyzstan	0	-3.74(-4.12 to -3.36)	0	-2.61(-2.87 to -2.34)	0	-4.16(-4.55 to -3.77)
	Lao People's Democratic Republic	0	-2.36(-2.58 to -2.15)	0	-2.08(-2.18 to -1.97)	0	-2.79(-2.97 to -2.62)
	Latvia	0	-2.97(-4 to -1.94)	0.002	-2(-3.23 to -0.75)	0	-2.89(-4.04 to -1.73)
	Lebanon	0	-0.91(-1.12 to -0.7)	0	-1.21(-1.3 to -1.11)	0	-1.53(-1.58 to -1.48)
	Lesotho	0	0.64(0.57 to 0.71)	0	0.53(0.46 to 0.59)	0	0.61(0.53 to 0.7)
	Liberia	0	-0.8(-1.11 to -0.49)	0	-0.43(-0.58 to -0.27)	0	-0.95(-1.12 to -0.77)
	Libya	0	-0.94(-1.22 to -0.65)	0.001	-0.66(-1.05 to -0.26)	0	-1.15(-1.55 to -0.75)
	Lithuania	0	-3.84(-4.74 to -2.93)	0	-3.02(-3.88 to -2.15)	0	-3.93(-4.78 to -3.07)
	Luxembourg	0	-2.37(-2.66 to -2.08)	0	-1.92(-2.22 to -1.62)	0	-2.63(-2.98 to -2.28)
	Madagascar	0	-1.98(-2.09 to -1.87)	0	-1.46(-1.64 to -1.29)	0	-2.19(-2.43 to -1.94)
	Malawi	0	-2.34(-2.72 to -1.97)	0	-2.02(-2.23 to -1.8)	0	-2.58(-2.87 to -2.29)
	Malaysia	0	-2.15(-2.49 to -1.81)	0	-1.74(-2.16 to -1.32)	0	-2.48(-2.65 to -2.3)
	Maldives	0	-2.87(-3.37 to -2.36)	0	-3.25(-3.49 to -3)	0	-4.26(-4.6 to -3.91)
	Mali	0.012	-0.49(-0.87 to -0.11)	0.001	-0.47(-0.76 to -0.19)	0.001	-0.74(-1.17 to -0.31)
	Malta	0	-1.59(-1.88 to -1.31)	0	-1.98(-2.24 to -1.71)	0	-2(-2.27 to -1.74)
	Marshall Islands	0	-0.37(-0.54 to -0.2)	0	-0.51(-0.63 to -0.38)	0	-0.47(-0.53 to -0.41)
	Mauritania	0	-0.63(-0.86 to -0.41)	0	-0.77(-0.95 to -0.59)	0	-1(-1.23 to -0.77)
	Mauritius	0	-1.39(-2.04 to -0.74)	0	-1.2(-1.77 to -0.63)	0	-1.64(-2.31 to -0.95)
	Mexico	0	-2.38(-2.64 to -2.12)	0	-1.82(-2.04 to -1.59)	0	-2.64(-2.91 to -2.37)
	Micronesia (Federated States of)	0	-0.61(-0.67 to -0.55)	0	-0.6(-0.63 to -0.57)	0	-0.87(-0.92 to -0.82)
	Monaco	0	-0.93(-1.02 to -0.84)	0.154	-0.06(-0.15 to 0.02)	0	-0.61(-0.68 to -0.54)
	Mongolia	0	-1.42(-1.66 to -1.18)	0	-0.91(-1.17 to -0.65)	0	-1.8(-2.1 to -1.51)
	Montenegro	0	-2.39(-2.83 to -1.95)	0	-1.84(-2.39 to -1.29)	0	-2.26(-2.6 to -1.92)
	Morocco	0	-0.58(-0.85 to -0.31)	0.014	-0.33(-0.59 to -0.07)	0	-0.94(-1.12 to -0.76)
	Mozambique	0	-0.97(-1.45 to -0.48)	0	-1.03(-1.32 to -0.74)	0	-1.38(-1.83 to -0.92)
	Myanmar	0	-1.95(-2.07 to -1.83)	0	-1.87(-1.96 to -1.78)	0	-2.52(-2.64 to -2.4)
	Namibia	0.142	-0.33(-0.77 to 0.11)	0	-0.67(-0.88 to -0.46)	0	-0.73(-1.06 to -0.4)
	Nauru	0	-0.41(-0.51 to -0.31)	0	-0.44(-0.51 to -0.37)	0	-0.6(-0.76 to -0.44)
	Nepal	0	-2.55(-2.69 to -2.41)	0	-1.65(-1.73 to -1.57)	0	-2.94(-3.08 to -2.8)
	Netherlands	0.009	1.3(0.33 to 2.29)	0	2.19(1.09 to 3.31)	0.02	1.13(0.18 to 2.09)
	New Zealand	0.005	-0.86(-1.46 to -0.26)	0.21	-0.32(-0.81 to 0.18)	0.003	-0.75(-1.25 to -0.25)
	Nicaragua	0	-1.51(-1.88 to -1.14)	0	-0.91(-1.42 to -0.41)	0	-2.13(-2.6 to -1.66)
	Niger	0.543	-0.09(-0.39 to 0.2)	0.867	0.02(-0.19 to 0.23)	0.045	-0.34(-0.67 to -0.01)
	Nigeria	0	-0.61(-0.72 to -0.51)	0	-0.61(-0.7 to -0.52)	0	-0.91(-1.08 to -0.74)
	Niue	0	-0.39(-0.49 to -0.29)	0	-0.8(-0.88 to -0.71)	0	-0.94(-1.02 to -0.86)
	North Macedonia	0	-0.88(-1.02 to -0.75)	0.001	-0.89(-1.4 to -0.37)	0	-1.13(-1.75 to -0.5)
	Northern Mariana Islands	0.841	-0.04(-0.46 to 0.38)	0.009	-0.43(-0.75 to -0.11)	0.084	-0.28(-0.59 to 0.04)
	Norway	0	-1.24(-1.86 to -0.63)	0.01	-0.61(-1.07 to -0.15)	0	-1.41(-1.86 to -0.96)
	Oman	0	-1.03(-1.31 to -0.75)	0	-0.91(-1.1 to -0.71)	0	-1.66(-1.85 to -1.47)
	Pakistan	0	-0.73(-0.93 to -0.53)	0	-0.98(-1.08 to -0.89)	0	-1.22(-1.42 to -1.01)

Palau	0	-0.68(-0.77 to -0.59)	0	-0.68(-0.75 to -0.61)	0	-0.86(-0.95 to -0.78)
Palestine	0	-1.32(-1.66 to -0.97)	0	-1.19(-1.4 to -0.98)	0	-1.65(-1.87 to -1.43)
Panama	0.22	-0.35(-0.91 to 0.21)	0.042	-0.53(-1.03 to -0.02)	0.034	-0.63(-1.22 to -0.05)
Papua New Guinea	0.449	-0.12(-0.42 to 0.19)	0.016	-0.12(-0.21 to -0.02)	0.028	-0.39(-0.73 to -0.04)
Paraguay	0	-2.04(-2.36 to -1.72)	0	-1.41(-1.78 to -1.04)	0	-2.29(-2.62 to -1.95)
Peru	0.009	-1.01(-1.75 to -0.26)	0.047	-1.05(-2.07 to -0.01)	0	-1.59(-2.33 to -0.84)
Philippines	0	-1.55(-1.81 to -1.28)	0	-1.69(-1.82 to -1.56)	0	-1.68(-1.91 to -1.45)
Poland	0	-3.68(-4.1 to -3.26)	0	-3.47(-3.86 to -3.08)	0	-4.17(-4.69 to -3.64)
Portugal	0	-3.24(-3.96 to -2.51)	0	-2.23(-2.84 to -1.6)	0	-3.78(-4.56 to -2.98)
Puerto Rico	0.003	-0.71(-1.19 to -0.24)	0	-0.79(-1.22 to -0.36)	0	-1.25(-1.76 to -0.73)
Qatar	0	-1.96(-2.79 to -1.13)	0.006	-1.61(-2.76 to -0.46)	0	-2.88(-3.69 to -2.07)
Republic of Korea	0	-3.88(-4.36 to -3.41)	0	-3.68(-4.07 to -3.3)	0	-5.19(-5.66 to -4.73)
Republic of Moldova	0	-3.29(-4.38 to -2.18)	0	-2.54(-3.05 to -2.02)	0	-3.44(-3.92 to -2.96)
Romania	0	-0.99(-1.1 to -0.88)	0	-0.47(-0.72 to -0.22)	0	-1.37(-2.05 to -0.68)
Russian Federation	0.018	-1.62(-2.93 to -0.28)	0.068	-1.16(-2.38 to 0.09)	0.007	-1.97(-3.37 to -0.55)
Rwanda	0.054	-0.93(-1.87 to 0.01)	0	-1.47(-1.88 to -1.07)	0	-1.66(-2.5 to -0.81)
Saint Kitts and Nevis	0	-1.67(-2.3 to -1.05)	0	-1.64(-1.86 to -1.43)	0	-2.17(-2.84 to -1.5)
Saint Lucia	0	-1.1(-1.27 to -0.93)	0	-1.27(-1.53 to -1.02)	0	-1.48(-1.6 to -1.36)
Saint Vincent and the Grenadines	0	-1(-1.34 to -0.66)	0	-0.34(-0.51 to -0.17)	0	-0.95(-1.38 to -0.51)
Samoa	0	-0.55(-0.61 to -0.48)	0	-0.47(-0.54 to -0.4)	0	-0.7(-0.78 to -0.63)
San Marino	0	-0.59(-0.68 to -0.5)	0	-0.36(-0.44 to -0.29)	0	-0.72(-0.8 to -0.64)
Sao Tome and Principe	0.193	-0.08(-0.2 to 0.04)	0.882	0.03(-0.34 to 0.4)	0.127	-0.19(-0.43 to 0.05)
Saudi Arabia	0	-1.21(-1.36 to -1.05)	0	-1.79(-1.95 to -1.63)	0	-1.95(-2.16 to -1.75)
Senegal	0.77	0.12(-0.67 to 0.92)	0.405	0.23(-0.31 to 0.77)	0.977	0.01(-0.79 to 0.82)
Serbia	0	-1.59(-2.32 to -0.86)	0	-0.82(-1.22 to -0.41)	0	-2(-2.66 to -1.34)
Seychelles	0.002	-0.8(-1.3 to -0.29)	0	-1.07(-1.35 to -0.79)	0	-1.26(-1.7 to -0.82)
Sierra Leone	0.001	0.42(0.18 to 0.66)	0	0.35(0.21 to 0.48)	0.032	0.25(0.02 to 0.48)
Singapore	0	-3.81(-4.68 to -2.93)	0	-3.16(-3.8 to -2.51)	0	-4.06(-4.76 to -3.35)
Slovakia	0.001	-0.69(-1.08 to -0.29)	0.028	-0.47(-0.89 to -0.05)	0	-1.09(-1.49 to -0.69)
Slovenia	0	-3.1(-3.61 to -2.59)	0	-2.8(-3.19 to -2.4)	0	-3.31(-3.88 to -2.73)
Solomon Islands	0.319	-0.09(-0.27 to 0.09)	0.007	-0.2(-0.34 to -0.05)	0.003	-0.24(-0.4 to -0.08)
Somalia	0	-0.68(-0.89 to -0.48)	0	-0.6(-0.75 to -0.44)	0	-0.69(-0.9 to -0.47)
South Africa	0	-1.03(-1.42 to -0.64)	0	-0.83(-1.23 to -0.43)	0	-1.53(-1.96 to -1.09)
South Sudan	0	-1.27(-1.42 to -1.12)	0	-1.01(-1.23 to -0.79)	0	-1.47(-1.64 to -1.29)
Spain	0	-2.81(-3.03 to -2.59)	0	-2.07(-2.31 to -1.83)	0	-3.34(-3.5 to -3.17)
Sri Lanka	0	-1.29(-1.72 to -0.86)	0	-1.26(-1.61 to -0.91)	0	-1.92(-2.48 to -1.35)
Sudan	0	-1.25(-1.53 to -0.98)	0	-0.66(-0.77 to -0.56)	0	-1.62(-1.82 to -1.42)
Suriname	0.165	-0.43(-1.03 to 0.18)	0.2	-0.29(-0.73 to 0.15)	0.083	-0.58(-1.24 to 0.08)
Sweden	0	-1.27(-1.82 to -0.7)	0.003	-0.58(-0.97 to -0.2)	0	-1.42(-1.75 to -1.1)
Switzerland	0	-2.12(-3.24 to -0.98)	0	-1.23(-1.77 to -0.68)	0	-2.28(-3.08 to -1.47)
Syrian Arab Republic	0	-1.04(-1.48 to -0.61)	0	-1.12(-1.26 to -0.98)	0	-1.63(-1.88 to -1.38)
Taiwan (Province of China)	0	-2.32(-3.27 to -1.36)	0	-1.92(-2.37 to -1.46)	0	-2.89(-3.7 to -2.08)
Tajikistan	0	-2.37(-2.64 to -2.1)	0	-1.36(-1.5 to -1.22)	0	-2.66(-3.11 to -2.21)
Thailand	0	-0.87(-1.13 to -0.62)	0	-1.18(-1.31 to -1.05)	0	-1.51(-1.77 to -1.24)
Timor-Leste	0	-1.61(-1.88 to -1.34)	0	-1.11(-1.33 to -0.89)	0	-1.96(-2.31 to -1.61)
Togo	0.431	0.12(-0.18 to 0.42)	0.071	0.2(-0.02 to 0.41)	0.555	0.1(-0.23 to 0.43)
Tokelau	0	-1.1(-1.29 to -0.91)	0	-1.02(-1.06 to -0.99)	0	-1.38(-1.49 to -1.27)
Tonga	0.006	-0.32(-0.55 to -0.09)	0	-0.35(-0.54 to -0.16)	0	-0.46(-0.64 to -0.27)

Trinidad and Tobago	0	-1.52(-2.02 to -1.02)	0	-1.57(-2.31 to -0.83)	0	-1.73(-2.47 to -0.99)
Tunisia	0	-1.92(-2.11 to -1.72)	0	-1.2(-1.31 to -1.08)	0	-2.08(-2.22 to -1.94)
Turkey	0	-3.1(-3.36 to -2.84)	0	-2.75(-3.05 to -2.44)	0	-3.95(-4.12 to -3.77)
Turkmenistan	0	-3.05(-3.9 to -2.2)	0	-1.95(-2.59 to -1.31)	0	-3.02(-3.78 to -2.26)
Tuvalu	0	-1.63(-1.76 to -1.5)	0	-1.15(-1.21 to -1.1)	0	-1.82(-1.93 to -1.7)
Uganda	0.203	-0.32(-0.81 to 0.17)	0	-0.54(-0.83 to -0.24)	0.004	-0.66(-1.09 to -0.22)
Ukraine	0	-2.12(-3.16 to -1.06)	0.005	-1.27(-2.15 to -0.38)	0	-2.15(-3.24 to -1.05)
United Arab Emirates	0.005	-1.22(-2.07 to -0.36)	0.005	-1.18(-2 to -0.36)	0	-1.44(-2.2 to -0.68)
United Kingdom	0	-0.6(-0.9 to -0.3)	0	-0.77(-1.05 to -0.5)	0	-1.42(-1.71 to -1.12)
United Republic of Tanzania	0	-0.63(-0.95 to -0.31)	0	-0.83(-1.08 to -0.58)	0	-0.99(-1.24 to -0.73)
United States of America	0	-1.35(-1.56 to -1.14)	0	-0.9(-1.08 to -0.73)	0	-1.56(-1.84 to -1.28)
United States Virgin Islands	0	-0.88(-1.11 to -0.64)	0	-0.68(-0.82 to -0.54)	0	-1.32(-1.79 to -0.84)
Uruguay	0	-0.87(-1.02 to -0.73)	0	-0.71(-0.82 to -0.6)	0.001	-1.11(-1.76 to -0.46)
Uzbekistan	0	-2.51(-2.85 to -2.17)	0	-1.5(-1.88 to -1.11)	0	-2.75(-3.05 to -2.45)
Vanuatu	0.849	0.03(-0.24 to 0.29)	0.221	-0.11(-0.29 to 0.07)	0.676	-0.04(-0.24 to 0.15)
Venezuela (Bolivarian Republic of)	0	-1.61(-2.15 to -1.07)	0	-1.3(-1.79 to -0.81)	0	-1.93(-2.51 to -1.35)
Viet Nam	0	-1.09(-1.15 to -1.03)	0	-1.22(-1.28 to -1.17)	0	-1.59(-1.64 to -1.53)
Yemen	0	-0.36(-0.51 to -0.22)	0.78	-0.01(-0.09 to 0.07)	0	-0.62(-0.8 to -0.43)
Zambia	0	-1.87(-2 to -1.73)	0	-1.73(-1.88 to -1.58)	0	-2.24(-2.44 to -2.04)
Zimbabwe	0	-1.85(-2.25 to -1.44)	0	-1.81(-2.02 to -1.6)	0	-1.82(-2.26 to -1.39)

Note: AAPC average annual percent change, CI confidence interval, DALYs disability-adjusted life years, GBD global burden of disease, SDI socio-demographic index.

TableS5: Decomposition analysis of leukemia in 2019.

location	Proportion of Total Change Attributed to Incidence							Proportion of Total Change Attributed to Deaths							Proportion of Total Change Attributed to DALYs						
	Aging	Population	Other leukemia	ALL	CLL	AML	CML	Aging	Population	Other leukemia	ALL	CLL	AML	CML	Aging	Population	Other leukemia	ALL	CLL	AML	CML
Global	38	120	-91	27	7	5	-6	87	155	-101	-13	-4	5	-29	-19	-411	378	64	5	8	76
Socio-demographic factor																					
High SDI	46	35	-12	20	1	7	4	97	57	-23	-7	-13	16	-26	263	290	-183	-75	-53	5	-147
High-middle SDI	37	56	-64	50	15	3	1	180	148	-170	-24	0	6	-40	-19	-97	155	28	1	10	23
Middle SDI	51	351	-462	98	45	27	-12	124	193	-219	-6	8	15	-14	8	-174	247	17	-4	-7	13
Low-middle SDI	-14	741	-581	-43	44	54	-100	52	187	-105	-17	7	11	-34	68	-613	446	98	-11	-17	129
Low SDI	-28	266	-85	-16	5	6	-48	-12	220	-47	-17	5	3	-52	-43	397	-106	-44	3	1	-109
Region																					
Andean Latin America	10	104	-41	8	4	15	-1	29	83	-28	4	2	12	-1	-9	160	-68	3	2	16	-3
Australasia	42	46	0	8	6	1	-4	69	66	3	-9	-7	2	-25	79	135	0	-38	-12	-16	-49
Caribbean	25	109	-46	6	7	8	-10	54	89	-25	-4	-1	5	-17	-23	359	-152	-27	-2	3	-58
Central Asia	15	-158	212	24	-8	4	12	-95	-794	633	213	-6	53	96	13	-125	130	47	1	18	15
Central Europe	59	-15	-25	20	49	13	-1	166	-32	-60	-21	43	34	-29	-172	68	152	81	-64	-31	67
Central Latin America	14	114	-67	25	4	16	-5	38	84	-36	8	1	11	-7	-20	203	-99	12	0	17	-13
Central Sub-Saharan Africa	-41	430	-262	-15	14	-4	-23	-12	232	-102	-10	12	-4	-17	-36	436	-229	-27	9	-12	-41
East Asia	13	231	-581	332	79	19	8	-248	-209	612	-25	-23	-24	17	14	-37	128	-3	-3	-2	2
Eastern Europe	148	-80	-112	55	136	-58	11	-127	52	61	61	-18	54	18	-12	17	25	38	-2	27	7
Eastern Sub-Saharan Africa	-109	687	-259	-56	11	7	-182	-55	500	-123	-53	11	4	-184	####	7295	-2192	-995	48	-5	-2917
High-income Asia Pacific	53	12	-23	46	2	5	6	159	22	-49	-12	-2	13	-32	-99	-38	121	35	2	20	58
High-income North America	55	57	-15	3	-4	12	-8	70	66	-18	-5	-10	17	-22	111	156	-63	-26	-26	12	-63
North Africa and Middle East	12	191	-88	-5	9	-4	-15	43	145	-46	-16	1	-6	-20	2	508	-209	-91	1	-41	-69
Oceania	0	105	-2	-1	0	1	-3	7	107	-7	-1	0	-2	-4	-1	116	-8	-1	0	-2	-4
South Asia	16	194	-74	-21	13	15	-43	46	139	-39	-19	4	6	-39	-90	1157	-390	-245	20	16	-367
Southeast Asia	28	318	-279	-7	13	35	-7	63	117	-84	-7	3	12	-5	250	-5446	5007	563	-60	-458	244
Southern Latin America	34	74	-24	15	4	10	-14	62	87	-23	-6	-4	11	-27	57	330	-126	-62	-10	3	-93
Southern Sub-Saharan Africa	32	104	-49	0	12	5	-2	42	86	-33	-1	5	2	-2	36	160	-93	-4	-1	6	-4
Tropical Latin America	48	134	-75	-4	8	9	-21	66	85	-29	-7	1	4	-19	22	656	-297	-113	2	-35	-135
Western Europe	32	19	-8	25	9	9	13	92	36	-20	-6	-5	30	-27	345	257	-244	-94	-49	99	-214
Western Sub-Saharan Africa	-9	114	-6	-2	3	2	-1	-9	118	-7	-3	2	1	-2	-8	123	-9	-4	1	-1	-3

Note: ALL acute lymphoblastic leukemia, AML acute myeloid leukemia, CLL chronic lymphocytic leukemia, CML chronic myeloid leukemia, DALYs disability-adjusted life years.

TableS6: Decomposition analysis of leukemia's subtypes in 2019.

Cause	Location	Incidence			Deaths			DALYs (Disability-Adjusted Life Years)		
		Aging	Population	Epidemiological change	Aging	Population	Epidemiological change	Aging	Population	Epidemiological change
Acute lymphoid leukemia	Global	3.7	43.7	52.5	-11.6	256.3	-144.6	518.8	-1462.6	1043.8
	High SDI	6.9	23.8	69.3	-581.6	-1049.8	1731.3	23.1	-90.8	167.6
	High-middle SDI	4.7	19.8	75.5	-97.3	-1754.8	1952.2	69.3	-91.7	122.4
	Middle SDI	-0.8	38.7	62.1	-12.6	137.8	-25.1	-4825.8	8592.1	-3666.3
	Low-middle SDI	-96.2	317.4	-121.2	-96.8	400.5	-203.7	504.5	-1183.8	779.4
	Low SDI	-23.0	195.3	-72.3	-21.2	194.9	-73.7	-29.2	222.3	-93.1
	Andean Latin America	-18.7	89.6	29.1	-12.6	97.6	15.0	-52.1	145.6	6.5
	Australasia	15.7	43.2	41.1	-124.6	-412.0	636.6	18.6	-123.2	204.5
	Caribbean	-28.9	95.7	33.2	-68.4	233.4	-65.0	-1129.6	1765.4	-535.8
	Central Asia	169.8	-858.8	789.0	36.4	-252.5	316.1	43.3	-128.2	184.9
	Central Europe	14.7	-11.8	97.1	-24.1	16.9	107.3	15.9	10.6	73.5
	Central Latin America	-16.4	69.1	47.3	-11.9	86.4	25.5	-68.5	148.1	20.4
	Central Sub-Saharan Africa	-13.4	171.0	-57.5	-11.9	161.7	-49.8	-16.3	181.7	-65.4
	East Asia	4.8	12.2	83.0	6.1	52.6	41.3	-474.6	392.2	182.4
	Eastern Europe	13.9	-45.8	131.9	-6.9	16.2	90.6	12.9	10.2	76.9
	Eastern Sub-Saharan Africa	-44.3	272.5	-128.3	-40.6	262.0	-121.4	-52.7	303.3	-150.6
	High-income Asia Pacific	10.6	7.9	81.5	-106.5	-52.4	258.9	27.0	-17.3	90.3
	High-income North America	12.3	56.2	31.5	93.9	216.5	-210.4	18.3	-233.8	315.6
	North Africa and Middle	-18.7	144.8	-26.1	-49.2	471.1	-322.0	382.2	-1325.7	1043.5
	Oceania	-6.7	115.3	-8.6	-4.6	113.5	-8.8	-10.0	117.7	-7.7
	South Asia	-1008.7	2820.3	-1711.6	32102.9	-108422.0	76419.0	207.0	-465.5	358.5
	Southeast Asia	-71.2	211.2	-40.1	-57.9	253.2	-95.2	580.5	-982.3	501.8
	Southern Latin America	-5.4	51.8	53.7	-7.2	176.8	-69.6	416.0	-993.3	677.3
	Southern Sub-Saharan Africa	-25.9	133.2	-7.3	-16.0	129.7	-13.7	-51.5	181.7	-30.1
	Tropical Latin America	-136.4	278.3	-41.9	-154.4	452.8	-198.4	244.5	-339.9	195.5
	Western Europe	2.4	14.2	83.4	-116.5	-146.9	363.4	18.4	-47.0	128.5
	Western Sub-Saharan Africa	-7.5	125.0	-17.5	-7.0	123.2	-16.2	-8.3	127.0	-18.7
Acute myeloid leukemia	Global	31.6	54.7	13.6	37.2	54.6	8.2	19.3	89.0	-8.2
	High SDI	45.1	30.3	24.6	47.3	30.2	22.5	47.1	50.7	2.2
	High-middle SDI	44.2	41.8	14.0	52.0	40.5	7.5	44.9	106.5	-51.5
	Middle SDI	23.3	46.3	30.4	32.4	45.0	22.6	6.4	73.3	20.3
	Low-middle SDI	11.2	60.8	28.0	19.3	61.3	19.4	-3.6	89.5	14.1
	Low SDI	-5.0	90.0	15.0	-3.9	95.8	8.1	-9.3	107.5	1.9
	Andean Latin America	14.5	47.3	38.2	20.3	46.4	33.3	2.8	63.3	33.9
	Australasia	46.7	49.2	4.1	48.0	49.1	2.8	44.6	74.0	-18.6
	Caribbean	26.3	51.8	21.9	34.1	52.5	13.3	9.7	86.4	3.9
	Central Asia	13.4	99.1	-12.5	23.5	102.9	-26.5	-0.5	227.8	-127.3
	Central Europe	57.8	-14.6	56.8	64.2	-14.4	50.2	83.7	-36.2	52.5
	Central Latin America	19.9	47.6	32.5	27.3	45.6	27.1	5.8	67.5	26.8
	Central Sub-Saharan Africa	-4.4	115.2	-10.8	-3.8	120.1	-16.2	-8.0	142.2	-34.2
	East Asia	28.7	28.3	42.9	39.7	26.4	33.9	-3.2	60.9	42.2
	Eastern Europe	-45.8	30.2	115.5	-60.5	30.9	129.6	-7.8	15.5	92.2
	Eastern Sub-Saharan Africa	-10.0	98.0	12.1	-8.7	101.9	6.8	-15.1	115.9	-0.8
	High-income Asia Pacific	72.3	10.8	16.9	75.5	10.5	14.0	111.7	33.8	-45.6
	High-income North America	36.0	35.7	28.3	37.2	36.8	26.0	37.5	52.0	10.5

	North Africa and Middle	21.6	87.4	-9.0	28.8	87.2	-16.0	12.6	132.2	-44.8
	Oceania	4.3	92.9	2.8	7.4	99.3	-6.8	0.8	105.5	-6.3
	South Asia	14.1	63.3	22.7	23.5	64.4	12.2	0.0	94.0	6.1
	Southeast Asia	22.2	43.7	34.1	29.5	43.8	26.6	9.7	59.4	30.9
	Southern Latin America	24.6	47.7	27.8	29.5	47.9	22.6	14.3	82.5	3.2
	Southern Sub-Saharan Africa	12.5	59.2	28.3	19.0	62.6	18.4	7.9	71.4	20.7
	Tropical Latin America	33.4	54.0	12.6	41.9	51.2	6.9	18.7	97.4	-16.1
	Western Europe	37.1	18.2	44.7	39.0	17.8	43.2	37.3	29.9	32.9
	Western Sub-Saharan Africa	-6.4	97.5	8.9	-7.4	103.7	3.8	-8.2	110.3	-2.1
Chronic lymphoid leukemia	Global	42.1	39.4	18.6	61.1	50.9	-12.0	55.2	56.4	-11.6
	High SDI	62.3	33.3	4.4	112.4	49.6	-62.0	128.0	70.1	-98.0
	High-middle SDI	39.7	20.3	40.1	68.7	30.5	0.8	68.2	36.7	-5.0
	Middle SDI	30.4	20.7	48.9	48.9	29.1	22.1	44.2	32.7	23.1
	Low-middle SDI	32.4	35.5	32.0	40.4	40.9	18.7	36.8	44.0	19.2
	Low SDI	3.5	67.3	29.3	5.1	73.2	21.7	2.8	76.4	20.8
	Andean Latin America	27.1	29.7	43.1	35.6	35.2	29.2	32.6	40.1	27.4
	Australasia	43.5	39.1	17.4	73.6	53.5	-27.1	72.3	65.2	-37.5
	Caribbean	43.6	31.4	25.1	63.9	42.7	-6.6	60.4	46.1	-6.5
	Central Asia	24.5	39.7	35.8	32.4	59.8	7.8	49.8	76.1	-25.9
	Central Europe	35.1	-6.2	71.0	55.7	-8.3	52.6	52.3	-9.4	57.1
	Central Latin America	42.3	29.5	28.2	57.1	36.6	6.3	55.1	41.4	3.5
	Central Sub-Saharan Africa	-0.6	54.0	46.5	0.2	55.9	44.0	-0.7	57.1	43.6
	East Asia	24.5	10.2	65.3	45.8	15.4	38.8	36.3	17.6	46.1
	Eastern Europe	39.3	-11.4	72.1	85.8	-21.7	35.9	101.1	-30.5	29.3
	Eastern Sub-Saharan Africa	0.5	68.2	31.3	1.5	72.1	26.4	0.1	76.2	23.6
	High-income Asia Pacific	65.3	7.8	26.9	119.9	10.7	-30.6	122.0	14.9	-36.9
	High-income North America	60.3	49.4	-9.7	84.9	64.9	-49.8	110.1	90.8	-101.0
	North Africa and Middle	23.9	37.3	38.7	36.7	54.3	9.0	37.0	60.1	2.9
	Oceania	15.2	82.9	1.9	17.5	92.3	-9.8	15.6	90.2	-5.9
	South Asia	36.4	38.6	25.1	45.0	43.8	11.2	39.9	47.1	13.1
	Southeast Asia	34.7	25.0	40.3	44.0	30.0	26.0	42.9	32.9	24.2
	Southern Latin America	40.8	37.1	22.1	70.3	54.8	-25.1	72.6	71.2	-43.8
	Southern Sub-Saharan Africa	35.6	45.1	19.3	39.6	51.0	9.4	44.7	56.7	-1.4
	Tropical Latin America	46.3	27.9	25.8	59.4	32.9	7.7	59.5	37.9	2.6
	Western Europe	44.8	17.6	37.5	91.2	27.1	-18.3	107.5	42.8	-50.3
	Western Sub-Saharan Africa	-15.7	88.6	27.1	-18.4	99.6	18.9	-15.5	96.7	18.8
	Global	57.9	84.7	-42.6	-667.7	-1038.4	1806.0	-32.1	-156.9	289.1
	High SDI	46.9	31.5	21.6	-76.7	-46.3	223.0	-32.3	-33.0	165.4
	High-middle SDI	54.8	33.8	11.5	-258.5	-150.5	509.0	-65.9	-63.4	229.3
	Middle SDI	82.1	88.0	-70.1	109.3	99.3	-108.5	247.7	449.6	-597.3
	Low-middle SDI	197.5	610.2	-707.7	134.8	308.7	-343.5	-31.0	-310.5	441.5
	Low SDI	45.6	-754.1	808.5	93.3	-2378.2	2384.9	28.2	-301.2	373.0
	Andean Latin America	41.4	68.4	-9.8	45.0	63.3	-8.3	38.5	101.4	-40.0
	Australasia	75.2	69.8	-45.0	-109.0	-97.2	306.2	-44.7	-65.7	210.4
	Caribbean	106.0	126.7	-132.7	221.2	225.3	-346.5	-158.0	-393.9	652.0
	Central Asia	-177.1	-413.4	690.5	-206.5	-437.4	744.0	-40.2	-121.5	261.6
	Central Europe	173.2	-37.3	-35.9	-95.6	17.8	177.8	-35.8	10.9	124.9
	Central Latin America	88.7	91.3	-80.0	102.1	92.2	-94.2	150.5	221.6	-272.1
[illegible]	Central Sub-Saharan Africa	-17.2	377.2	-260.0	-8.8	277.3	-168.5	-74.6	1104.2	-929.6

Chronic myeloid leukemia	East Asia	37.9	20.7	41.4	-664.3	-283.0	1047.3	-51.3	-52.3	203.6
	Eastern Europe	83.7	-31.1	47.3	-315.0	103.7	311.2	-54.2	28.1	126.2
	Eastern Sub-Saharan Africa	26.2	-256.6	330.4	27.9	-337.9	410.0	23.8	-182.7	258.9
	High-income Asia Pacific	57.4	9.8	32.8	-123.3	-16.4	239.8	-27.0	-8.7	135.7
	High-income North America	132.2	136.0	-168.2	-55.0	-54.4	209.4	-26.4	-38.2	164.5
	North Africa and Middle	81.2	212.6	-193.8	125.3	279.8	-305.1	1258.0	5417.5	-6575.5
	Oceania	19.3	148.2	-67.5	21.1	144.5	-65.6	14.9	156.0	-70.8
	South Asia	141.5	450.0	-491.5	120.0	282.6	-302.6	-40.4	-438.4	578.7
	Southeast Asia	67.6	79.7	-47.3	73.1	75.5	-48.6	89.3	161.8	-151.1
	Southern Latin America	-152.8	-185.5	438.3	-98.7	-105.4	304.1	-36.2	-62.2	198.4
	Southern Sub-Saharan Africa	61.0	171.6	-132.6	68.6	157.3	-125.9	52.7	248.0	-200.6
	Tropical Latin America	-464.7	-423.1	987.9	-1652.5	-1315.0	3067.6	-84.7	-109.6	294.2
	Western Europe	29.9	14.4	55.7	-63.5	-25.0	188.5	-27.5	-18.5	146.0
	Western Sub-Saharan Africa	-3.0	121.8	-18.8	-3.5	118.6	-15.1	-1.8	128.3	-26.5
Other leukemia	Global	-3.2	-135.4	238.6	-841.3	-1582.4	2523.7	-1.1	-87.1	188.2
	High SDI	379.3	325.9	-605.2	132.2	71.5	-103.7	-231.3	-229.3	560.6
	High-middle SDI	-8.9	-61.2	170.1	-236.2	-196.7	532.9	-6.0	-39.7	145.7
	Middle SDI	3.7	-84.1	180.4	-175.6	-252.6	528.1	0.6	-60.8	160.1
	Low-middle SDI	26.3	-136.1	209.8	-165.8	-588.0	853.8	13.0	-102.7	189.7
	Low SDI	-74.6	503.0	-328.4	-19.7	328.3	-208.6	-136.6	1126.9	-890.3
	Andean Latin America	28.4	306.9	-235.3	70.2	135.9	-106.1	4445.2	45953.4	-50298.6
	Australasia	45.9	50.2	3.9	48.2	37.7	14.1	46.0	53.8	0.2
	Caribbean	8.5	623.0	-531.5	66.9	100.3	-67.3	51.3	-1624.7	1673.5
	Central Asia	15.1	-55.3	140.2	-6.3	-104.1	210.4	8.5	-55.8	147.3
	Central Europe	-89.7	24.6	165.1	-403.3	69.1	434.2	-56.2	17.0	139.2
	Central Latin America	-5.8	-209.1	315.0	207.0	302.5	-409.4	-12.6	-158.5	271.1
	Central Sub-Saharan Africa	-1156.5	9926.5	-8670.0	-35.9	625.9	-489.9	2078.1	-22490.5	20512.4
	East Asia	11.6	-31.8	120.2	-61.6	-49.9	211.5	7.7	-22.8	115.1
	Eastern Europe	-25.2	16.2	109.0	-96.0	31.3	164.6	-20.2	14.0	106.2
	Eastern Sub-Saharan Africa	-475.5	2343.9	-1768.5	-326.3	2372.8	-1946.5	157.6	-874.9	817.3
	High-income Asia Pacific	-169.1	-38.3	307.4	292.2	35.1	-227.3	-61.5	-17.2	178.7
	High-income North America	162.1	199.5	-261.7	96.0	85.7	-81.6	425.8	540.9	-866.8
	North Africa and Middle	-133.3	2126.4	-1893.1	57.7	167.2	-124.9	-45.6	-3673.5	3819.1
	Oceania	-2.8	106.6	-3.8	6.9	106.0	-12.9	-2.2	118.4	-16.2
	South Asia	42.7	-206.3	263.7	-10956.0	-33193.9	44249.9	18.9	-135.6	216.8
	Southeast Asia	6.2	-305.8	399.6	160.4	269.8	-330.2	-0.4	-166.4	266.8
	Southern Latin America	74.6	150.3	-124.9	76.4	82.2	-58.6	186.5	429.9	-516.4
	Southern Sub-Saharan Africa	55.2	364.4	-319.6	68.0	156.5	-124.5	423.3	2437.9	-2761.2
	Tropical Latin America	-83.7	-375.8	559.5	114.6	106.9	-121.5	-68.9	-248.0	416.9
	Western Europe	1096.6	558.1	-1554.7	149.6	52.0	-101.6	-130.2	-82.1	312.3
	Western Sub-Saharan Africa	-11.4	124.8	-13.3	-11.1	132.5	-21.4	-9.0	137.7	-28.6

Note: DALYs disability-adjusted life years, SDI socio-demographic index.

TableS7: Predicted Analysis of Global Leukemia Disease Burden, 2020-2030.

Year	Deaths									Incidence								
	ASR			ASR_prediction			number_prediction			ASR			ASR_prediction			number_prediction		
	val	upper	lower	pred_val	pred_low	pred_up	pred_val	pred_low	pred_up	val	upper	lower	pred_val	pred_low	pred_up	pred_val	pred_low	pred_up
1990	5.8	6.4	5.2	5.8	0.7	32.0	318211.8	315873.6	320550.1	9.6	11.0	8.1	9.6	1.1	34.4	521119.6	518165.6	524073.7
1991	5.8	6.4	5.2	5.8	0.7	32.0	320372.3	318087.6	322657.1	9.6	11.0	8.2	9.6	1.1	34.5	529317.6	526371.6	532263.6
1992	5.7	6.3	5.2	5.7	0.7	32.0	322621.4	320337.8	324905.0	9.6	10.9	8.2	9.6	1.1	34.5	536872.6	533909.6	539835.6
1993	5.7	6.2	5.2	5.7	0.7	32.1	325733.5	323441.6	328025.5	9.6	10.9	8.3	9.7	1.1	34.5	547463.8	544470.6	550456.9
1994	5.7	6.2	5.2	5.7	0.7	32.1	328685.3	326386.3	330984.3	9.7	10.9	8.4	9.7	1.1	34.6	556404.7	553387.2	559422.2
1995	5.6	6.1	5.1	5.6	0.7	32.1	331077.0	328772.3	333381.8	9.7	10.8	8.4	9.7	1.2	34.6	564483.9	561444.2	567523.6
1996	5.6	6.1	5.1	5.6	0.7	32.1	331822.5	329517.8	334127.3	9.6	10.7	8.4	9.6	1.2	34.5	569192.9	566141.0	572244.8
1997	5.5	6.0	5.0	5.5	0.7	32.1	333027.9	330721.4	335334.4	9.6	10.7	8.4	9.6	1.2	34.5	575789.4	572720.7	578858.2
1998	5.5	5.9	5.0	5.5	0.7	32.1	334981.8	332670.7	337292.9	9.6	10.6	8.5	9.6	1.3	34.6	584238.0	581147.2	587328.8
1999	5.4	5.9	5.0	5.4	0.7	32.2	337182.6	334866.2	339499.0	9.6	10.6	8.5	9.6	1.3	34.6	591195.5	588087.4	594303.5
2000	5.4	5.8	4.9	5.4	0.7	32.2	338196.8	335880.0	340513.7	9.5	10.5	8.5	9.5	1.3	34.5	592606.4	589497.2	595715.7
2001	5.3	5.7	4.8	5.3	0.7	32.1	338102.1	335788.9	340415.4	9.4	10.3	8.4	9.4	1.3	34.4	592973.3	589865.4	596081.1
2002	5.2	5.6	4.8	5.2	0.7	32.0	337159.3	334852.7	339465.9	9.2	10.1	8.3	9.3	1.3	34.3	592561.8	589457.4	595666.2
2003	5.1	5.5	4.7	5.1	0.7	32.0	335930.8	333632.4	338229.3	9.2	10.0	8.3	9.2	1.4	34.3	594117.4	591012.1	597222.7
2004	5.0	5.4	4.6	5.0	0.7	31.9	333988.4	331702.0	336274.7	9.0	9.8	8.2	9.0	1.3	34.2	591623.4	588530.5	594716.2
2005	5.0	5.3	4.6	5.0	0.7	32.0	334297.2	332014.7	336579.6	8.9	9.7	8.1	8.9	1.3	34.1	593515.0	590422.5	596607.6
2006	4.9	5.2	4.5	4.9	0.7	31.9	332856.6	330584.7	335128.4	8.8	9.4	8.0	8.8	1.3	34.0	590368.9	587290.5	593447.3
2007	4.8	5.1	4.4	4.8	0.7	31.8	332457.2	330192.5	334722.0	8.7	9.3	8.0	8.7	1.3	33.9	592195.4	589118.4	595272.4
2008	4.8	5.1	4.4	4.8	0.7	31.9	334158.0	331892.8	336423.1	8.7	9.3	8.0	8.7	1.3	34.0	600400.3	597308.3	603492.4
2009	4.7	5.0	4.3	4.7	0.7	32.0	334060.3	331801.4	336319.1	8.7	9.2	8.0	8.7	1.3	34.0	606010.0	602910.3	609109.8
2010	4.7	5.0	4.3	4.7	0.7	31.9	334129.5	331876.1	336382.8	8.6	9.2	8.0	8.6	1.3	33.9	609011.5	605910.5	612112.5
2011	4.6	4.9	4.2	4.6	0.7	31.9	334654.4	332405.1	336903.7	8.5	9.1	7.9	8.5	1.4	33.9	611760.9	608659.2	614862.6
2012	4.6	4.8	4.2	4.6	0.7	31.8	334455.1	332212.8	336697.4	8.4	9.0	7.9	8.4	1.4	33.8	613039.8	609942.0	616137.5
2013	4.5	4.8	4.1	4.5	0.7	31.8	334239.3	332003.7	336474.8	8.4	8.9	7.8	8.4	1.4	33.8	616307.3	613208.2	619406.5
2014	4.5	4.7	4.1	4.5	0.6	31.8	334139.1	331910.1	336368.1	8.3	8.9	7.7	8.3	1.3	33.8	620161.9	617060.2	623263.6
2015	4.4	4.7	4.1	4.4	0.6	31.9	335341.9	333114.6	337569.2	8.3	8.9	7.7	8.3	1.4	33.8	626007.9	622898.5	629117.2
2016	4.4	4.6	4.0	4.4	0.6	31.9	335633.3	333411.3	337855.3	8.3	8.8	7.7	8.3	1.3	33.8	627665.7	624559.9	630771.5
2017	4.3	4.6	4.0	4.3	0.6	32.0	335013.1	332800.3	337225.9	8.2	8.9	7.5	8.2	1.3	33.8	631609.0	628502.1	634716.0
2018	4.3	4.6	3.9	4.3	0.6	32.2	336150.9	333937.8	338364.0	8.2	8.9	7.6	8.2	1.3	33.8	639717.0	636596.2	642837.8
2019	4.3	4.6	3.9	4.3	0.6	32.2	336955.4	334708.1	339202.7	8.2	8.9	7.5	8.2	1.3	33.8	644390.4	641248.5	647532.3
2020				4.3	0.4	43.2	342426.2	334852.9	349999.6				8.3	1.1	41.9	663167.5	645542.4	680792.6
2021				4.2	0.3	53.1	343236.8	332190.9	354282.8				8.3	0.8	50.0	669658.7	644066.1	695251.4
2022				4.2	0.1	90.7	343885.2	328373.4	359396.9				8.3	0.3	80.1	676024.1	640052.2	711996.0
2023				4.2	0.0	158.6	344408.9	323682.3	365135.5				8.3	0.1	137.2	682358.3	634098.4	730618.3
2024				4.1	0.0	237.3	344794.8	318231.7	371358.0				8.3	0.0	207.6	688568.4	626369.1	750767.6
2025		NA		4.1	0.0	309.2	345011.4	312069.1	377953.7		NA		8.3	0.0	275.8	694527.0	616893.2	772160.8
2026				4.0	0.0	368.2	345038.0	305250.9	384825.0				8.3	0.0	334.0	700158.1	605744.7	794571.5
2027				4.0	0.0	413.9	344904.3	297857.5	391951.0				8.3	0.0	380.4	705596.2	593137.6	818054.9
2028				3.9	0.0	448.2	344649.1	289959.6	399338.7				8.3	0.0	415.8	710957.4	579223.5	842691.3
2029				3.9	0.0	473.5	344263.2	281574.8	406951.7				8.3	0.0	442.1	716166.6	563968.5	868364.7
2030				3.9	0.0	491.9	343712.9	272700.3	414725.5				8.3	0.0	461.3	721091.5	547299.6	894883.3

Note: ASR age-standardized rate.

TableS8: Frontiers analysis of ASDR and ASR-DALYs in leukemia across 204 countries.

Location	SDI	Age-standardized Deaths rank				Age-standardized DALYs rank			
		Age-standardized Deaths	Frontier Deaths	Effective difference	Effective difference rank	Age-standardized DALYs	Frontier DALYs	Effective difference	Effective difference rank
Afghanistan	0.3	10.01(6.95 to 14.38)	2.4	7.6	204 (204)	365.49(241.81 to 546.42)	87.6	277.9	204 (204)
Albania	0.7	4.19(3.17 to 5.55)	2.3	1.9	96 (97)	178.59(136.15 to 226.78)	76.2	102.4	159 (160)
Algeria	0.7	3.02(2.35 to 3.71)	2.3	0.7	30 (28)	97.91(75.39 to 121.05)	76.5	21.5	23 (17)
American Samoa	0.7	2.96(2.42 to 3.61)	2.3	0.6	29 (27)	100.01(79.41 to 128.46)	76.1	23.9	27 (22)
Andorra	0.9	6.28(4.76 to 7.97)	2.3	4.0	184 (183)	174.45(130.55 to 226.22)	74.4	100.1	156 (152)
Angola	0.5	3.31(2.45 to 4.35)	2.3	1.0	44 (41)	124.63(92.38 to 156.71)	80.4	44.2	54 (57)
Antigua and Barbuda	0.7	4.27(3.63 to 5.01)	2.3	2.0	105 (103)	147.26(121.85 to 179.55)	73.9	73.3	118 (109)
Argentina	0.7	5.16(4.8 to 5.5)	2.3	2.8	158 (160)	175.47(164.63 to 186.96)	76.2	99.3	154 (154)
Armenia	0.7	4.34(3.69 to 5.07)	2.3	2.0	106 (107)	177.19(151.68 to 205.45)	76.4	100.7	157 (158)
Australia	0.8	4.79(4.3 to 5.18)	2.3	2.5	139 (139)	121.38(112.21 to 129.57)	74.3	47.1	58 (52)
Austria	0.8	4.64(4.18 to 5.02)	2.3	2.4	132 (132)	121.22(110.75 to 130.23)	73.5	47.7	59 (51)
Azerbaijan	0.7	4.62(3.56 to 5.91)	2.3	2.3	127 (130)	223.73(166.71 to 293.15)	76.2	147.5	190 (189)
Bahamas	0.8	3.67(3.03 to 4.48)	2.3	1.4	72 (70)	139.95(113.69 to 173.17)	74.3	65.6	99 (89)
Bahrain	0.8	5.49(4.29 to 6.84)	2.3	3.2	171 (169)	147.82(116.51 to 183.74)	74.2	73.6	120 (111)
Bangladesh	0.5	2.55(1.86 to 3.39)	2.3	0.2	9 (7)	95.25(70.98 to 124.22)	76.4	18.8	16 (11)
Barbados	0.7	5.14(4.27 to 6.15)	2.3	2.9	161 (159)	176.96(145.53 to 217.34)	74.3	102.7	161 (157)
Belarus	0.7	4.58(3.61 to 5.84)	2.3	2.3	128 (127)	157.62(123.87 to 201.98)	73.8	83.9	135 (132)
Belgium	0.9	5.26(4.73 to 5.72)	2.3	3.0	163 (163)	131.09(120.62 to 141.58)	73.7	57.4	75 (70)
Belize	0.6	3.34(2.91 to 3.77)	2.3	1.0	48 (48)	129.92(113.14 to 146.61)	76.2	53.7	70 (68)
Benin	0.4	3.55(2.57 to 4.75)	2.4	1.1	51 (55)	139.27(94.34 to 199.94)	87.9	51.4	65 (86)
Bermuda	0.8	3.59(3.03 to 4.35)	2.3	1.3	63 (59)	119.38(98.86 to 144.4)	73.9	45.4	56 (49)
Bhutan	0.5	3.32(2.34 to 4.69)	2.3	1.0	45 (43)	113.8(78 to 165.14)	82.6	31.3	38 (42)
Bolivia (Plurinational State of)	0.6	6.84(5.31 to 8.58)	2.3	4.5	197 (197)	253.39(188.66 to 323.15)	76.2	177.2	199 (199)
Bosnia and Herzegovina	0.7	4.22(3.22 to 5.28)	2.3	1.9	100 (99)	136.72(99.41 to 173.8)	75.1	61.6	87 (82)
Botswana	0.6	4.19(2.94 to 5.58)	2.3	1.9	97 (98)	136.01(95.41 to 182.11)	76.2	59.9	82 (80)
Brazil	0.6	3.96(3.68 to 4.16)	2.3	1.6	84 (84)	146.35(136.71 to 156.49)	76.5	69.9	109 (107)
Brunei Darussalam	0.8	5.63(4.82 to 6.39)	2.3	3.3	174 (175)	208.63(171.43 to 239.39)	73.8	134.8	183 (182)
Bulgaria	0.8	4.75(3.8 to 5.86)	2.3	2.5	137 (137)	172.8(137.35 to 213.26)	73.7	99.1	153 (149)
Burkina Faso	0.3	3.73(2.8 to 4.91)	2.5	1.3	61 (73)	150.62(102.15 to 212.98)	90.9	59.7	80 (120)
Burundi	0.3	3.26(2.26 to 4.63)	2.5	0.8	36 (39)	133.18(85.63 to 204.4)	89.3	43.9	53 (73)
Cabo Verde	0.5	4.04(3.41 to 4.93)	2.3	1.7	87 (86)	138.88(112.69 to 171.79)	76.1	62.7	90 (85)
Cambodia	0.5	5.92(4.7 to 7.24)	2.3	3.6	180 (180)	219.1(170.08 to 275.68)	81.1	138.0	186 (188)
Cameroon	0.5	3.81(2.67 to 5.11)	2.3	1.5	78 (77)	142.57(95.11 to 199.78)	76.2	66.4	103 (98)
Canada	0.9	4.96(4.5 to 5.38)	2.3	2.7	148 (148)	129.88(120.23 to 139.96)	74.1	55.8	73 (67)
Central African Republic	0.3	3.78(2.63 to 5.15)	2.5	1.3	69 (75)	153.27(98.53 to 235.37)	89.5	63.8	93 (125)
Chad	0.2	3.62(2.69 to 4.66)	2.5	1.2	55 (64)	141.27(100 to 189.18)	91.6	49.7	63 (93)
Chile	0.8	4.13(3.82 to 4.46)	2.3	1.9	95 (93)	145.89(134.13 to 158.86)	74.4	71.5	112 (105)
China	0.7	3.67(3.07 to 4.28)	2.3	1.3	71 (69)	163.82(136.6 to 189.48)	76.4	87.4	138 (138)
Colombia	0.6	4.39(3.42 to 5.5)	2.3	2.1	109 (111)	190.35(148.43 to 237.81)	76.2	114.2	171 (171)
Comoros	0.5	3.11(2.22 to 4.23)	2.3	0.8	33 (33)	123.89(77.95 to 188.66)	83.5	40.4	48 (55)
Congo	0.6	3.27(2.43 to 4.29)	2.3	0.9	43 (40)	111.4(82.42 to 148.22)	76.2	35.3	41 (38)
Cook Islands	0.8	2.34(1.82 to 2.91)	2.3	0.1	2 (2)	73.45(53.36 to 95.34)	73.4	0.0	2 (2)
Costa Rica	0.7	5.11(3.96 to 6.37)	2.3	2.8	154 (157)	183.69(143.26 to 231.04)	76.2	107.5	167 (166)
Croatia	0.8	4.81(3.84 to 5.98)	2.3	2.5	142 (140)	131.14(104.73 to 163.04)	74.2	57.0	74 (71)
Cuba	0.7	4(3.28 to 4.87)	2.3	1.7	86 (85)	140.97(115.25 to 169.72)	76.3	64.7	95 (92)
Cyprus	0.8	5.61(4.02 to 6.75)	2.3	3.3	173 (173)	139.78(106.19 to 164.92)	73.5	66.2	102 (88)
Czechia	0.8	5.07(4.2 to 6.07)	2.3	2.8	156 (153)	131.07(108.23 to 159.33)	73.5	57.6	76 (69)
Democratic People's Republic of Korea	0.6	4.49(3.4 to 5.81)	2.3	2.2	120 (121)	199.98(150.52 to 266.09)	76.2	123.8	177 (177)
Democratic Republic of the	0.4	2.81(1.91 to 3.96)	2.3	0.5	16 (15)	102.46(72.74 to 136.04)	84.2	18.3	15 (30)
Denmark	0.9	5.06(4.56 to 5.59)	2.3	2.8	153 (152)	127.35(115.71 to 139.43)	73.9	53.4	68 (61)

Djibouti	0.5	3.66(2.51 to 5.27)	2.3	1.3	66 (68)	150.81(93.43 to 230.31)	83.3	67.5	105 (121)
Dominica	0.7	6.39(5.12 to 7.83)	2.3	4.1	186 (186)	245.07(190.33 to 308.46)	74.2	170.9	197 (197)
Dominican Republic	0.6	4.05(2.93 to 5.51)	2.3	1.7	88 (87)	157.64(114.91 to 216.49)	76.3	81.4	133 (133)
Ecuador	0.6	5.75(4.34 to 7.21)	2.3	3.4	176 (177)	231.51(172.78 to 289.73)	76.2	155.3	193 (194)
Egypt	0.7	3.78(2.63 to 5.33)	2.3	1.5	75 (76)	136.07(99.78 to 183.29)	76.3	59.7	81 (81)
El Salvador	0.6	4.84(3.51 to 6.28)	2.3	2.5	141 (142)	188.95(137.16 to 248.1)	76.3	112.6	170 (170)
Equatorial Guinea	0.7	2.92(1.79 to 4.39)	2.3	0.6	27 (25)	94.23(55.78 to 147.54)	76.4	17.9	13 (9)
Eritrea	0.4	3.59(2.49 to 5.03)	2.3	1.3	59 (58)	145.33(93.17 to 223.93)	84.4	60.9	85 (102)
Estonia	0.8	5.62(4.38 to 6.96)	2.3	3.3	175 (174)	169.16(135.61 to 208.38)	73.5	95.6	148 (142)
Eswatini	0.6	4.11(2.87 to 5.46)	2.3	1.8	92 (92)	134.04(90.64 to 183.76)	76.3	57.8	77 (77)
Ethiopia	0.3	7.74(4.51 to 10.92)	2.4	5.3	200 (200)	290.14(163.46 to 397.21)	89.1	201.1	202 (202)
Fiji	0.7	6.47(5.07 to 8.1)	2.3	4.1	190 (190)	247.57(191.7 to 315.24)	76.4	171.2	198 (198)
Finland	0.9	3.6(3.29 to 3.92)	2.3	1.3	65 (61)	101.13(92.61 to 110.5)	73.6	27.5	32 (27)
France	0.8	5.32(4.7 to 5.82)	2.3	3.0	166 (166)	134.7(123.72 to 145.71)	73.9	60.8	84 (78)
Gabon	0.7	3.33(2.35 to 4.27)	2.3	1.0	46 (44)	112.17(78.67 to 146.34)	76.4	35.8	42 (40)
Gambia	0.4	2.67(2.04 to 3.3)	2.3	0.3	12 (11)	91.98(66.73 to 118.06)	84.4	7.6	5 (7)
Georgia	0.7	4.82(4.06 to 5.68)	2.3	2.5	138 (141)	197.88(167.99 to 230.27)	76.3	121.6	176 (176)
Germany	0.9	4.88(4.38 to 5.31)	2.3	2.6	145 (143)	131.29(120.84 to 142.41)	73.4	57.9	78 (72)
Ghana	0.6	2.91(2.3 to 3.67)	2.3	0.6	26 (24)	106.22(78.54 to 138.08)	76.3	30.0	36 (33)
Global	0.7	4.26(3.91 to 4.58)	2.3	1.9	102 (102)	150.51(135.71 to 164.14)	76.2	74.4	121 (119)
Greece	0.8	6.59(5.95 to 7.05)	2.3	4.3	194 (194)	168.83(155.89 to 181.27)	73.9	94.9	146 (141)
Greenland	0.8	3.2(2.57 to 3.94)	2.3	0.9	40 (35)	86.03(67.38 to 108.77)	73.8	12.2	9 (6)
Grenada	0.7	4.28(3.84 to 4.73)	2.3	2.0	103 (104)	149.56(131.65 to 168.13)	76.2	73.4	119 (115)
Guam	0.8	3.34(2.72 to 4.08)	2.3	1.0	49 (45)	126.31(102.61 to 155.09)	73.5	52.8	67 (58)
Guatemala	0.5	4.89(3.8 to 6.1)	2.3	2.6	143 (144)	211.04(164.15 to 264.2)	76.2	134.9	184 (184)
Guinea	0.3	2.8(2.11 to 3.53)	2.5	0.4	13 (14)	103.63(76.48 to 133.59)	89.3	14.3	11 (31)
Guinea-Bissau	0.4	4.09(3.07 to 5.44)	2.4	1.7	85 (90)	150.17(111.08 to 201.49)	87.5	62.7	89 (118)
Guyana	0.6	3.87(3.01 to 4.89)	2.3	1.6	81 (82)	156.01(120.56 to 200.3)	76.2	79.8	129 (130)
Haiti	0.4	6.4(4.09 to 9.58)	2.3	4.1	187 (187)	294.1(168.51 to 475.78)	84.2	209.9	203 (203)
Honduras	0.5	6.28(4.83 to 7.97)	2.3	4.0	183 (184)	216.06(161.02 to 281.01)	76.2	139.9	187 (186)
Hungary	0.8	5.27(4.4 to 6.28)	2.3	3.0	164 (164)	152.94(126.72 to 184.02)	74.2	78.7	128 (124)
Iceland	0.9	3.67(3.17 to 4.16)	2.3	1.4	73 (71)	101.56(88.27 to 115.57)	73.7	27.8	34 (28)
India	0.6	2.87(2.44 to 3.42)	2.3	0.5	19 (18)	100.48(85.3 to 122.29)	76.2	24.3	30 (23)
Indonesia	0.7	4.93(3.99 to 5.95)	2.3	2.6	146 (146)	180.52(145.51 to 220.76)	76.3	104.2	162 (161)
Iran (Islamic Republic of)	0.7	6.04(4.31 to 6.85)	2.3	3.7	181 (181)	205.3(140.29 to 237.81)	76.2	129.1	181 (181)
Iraq	0.7	6.95(5.4 to 8.75)	2.3	4.6	198 (198)	227.6(176.92 to 283.88)	76.2	151.4	192 (192)
Ireland	0.9	4.25(3.81 to 4.67)	2.3	2.0	104 (101)	109.9(99.9 to 120.52)	73.6	36.3	43 (36)
Israel	0.8	6.24(5.5 to 6.8)	2.3	4.0	182 (182)	154.42(141.13 to 166.92)	73.9	80.5	132 (128)
Italy	0.8	5.1(4.61 to 5.38)	2.3	2.8	157 (156)	146.68(137.03 to 155.08)	73.4	73.3	117 (108)
Jamaica	0.7	4.45(3.47 to 5.57)	2.3	2.1	116 (118)	170.67(132.24 to 217.06)	76.2	94.5	143 (144)
Japan	0.9	3.09(2.79 to 3.25)	2.3	0.8	37 (32)	97.51(92.15 to 102.16)	73.6	24.0	28 (16)
Jordan	0.7	6.62(5.29 to 7.99)	2.3	4.3	195 (195)	191.83(155.83 to 229.54)	74.7	117.2	173 (173)
Kazakhstan	0.7	3.56(3.13 to 4.04)	2.3	1.2	58 (57)	140.32(123.18 to 159.52)	75.0	65.4	97 (91)
Kenya	0.5	2.53(1.97 to 3.19)	2.3	0.2	7 (5)	95.22(70.82 to 122.24)	76.2	19.0	17 (10)
Kiribati	0.5	4.43(3.51 to 5.63)	2.3	2.1	115 (116)	171.26(131.59 to 219.89)	76.4	94.9	145 (147)
Kuwait	0.9	3.62(3.01 to 4.39)	2.3	1.3	70 (63)	110.42(92.39 to 133.64)	73.5	37.0	44 (37)
Kyrgyzstan	0.6	2.85(2.5 to 3.24)	2.3	0.5	18 (17)	113.98(100.39 to 129.02)	76.3	37.7	45 (43)
Lao People's Democratic	0.5	5.5(4.09 to 7.01)	2.3	3.2	169 (170)	214.01(153.26 to 280.79)	76.4	137.6	185 (185)
Latvia	0.8	5.02(4.13 to 6.05)	2.3	2.7	150 (149)	162.15(132.78 to 196.97)	73.5	88.6	139 (136)
Lebanon	0.7	6.52(5.15 to 8.53)	2.3	4.2	191 (191)	203.88(160.37 to 254.39)	76.2	127.7	179 (179)
Lesotho	0.5	4.53(3.18 to 6.11)	2.3	2.2	124 (124)	145.15(100.02 to 199.82)	76.3	68.8	108 (101)
Liberia	0.4	3.15(2.18 to 4.23)	2.4	0.8	35 (34)	113.35(77.85 to 153.04)	84.0	29.3	35 (41)
Libya	0.7	5.18(4 to 6.49)	2.3	2.9	160 (161)	168.55(131.72 to 214.29)	76.2	92.4	141 (140)
Lithuania	0.8	5.13(4.24 to 6.13)	2.3	2.9	159 (158)	157.12(130.46 to 187.5)	73.6	83.5	134 (131)
Luxembourg	0.9	5.46(4.78 to 6.25)	2.3	3.2	168 (168)	148.27(129.07 to 171.25)	73.6	74.7	122 (113)
Madagascar	0.4	2.63(1.95 to 3.48)	2.3	0.3	11 (10)	105.44(75.6 to 141.53)	84.2	21.3	22 (32)
Malawi	0.4	2.55(1.86 to 3.46)	2.4	0.2	6 (8)	102.3(68.87 to 153.46)	84.4	17.9	14 (29)

Malaysia	0.7	5.1(4.01 to 6.64)	2.3	2.8	155 (155)	180.99(141.59 to 232.52)	74.1	106.9	166 (164)
Maldives	0.6	3.51(2.86 to 4.17)	2.3	1.2	56 (53)	118.38(97.05 to 146.06)	76.2	42.2	49 (47)
Mali	0.3	2.62(1.99 to 3.43)	2.4	0.2	5 (9)	99.78(72.45 to 136.89)	90.4	9.4	6 (21)
Malta	0.8	3.86(3.34 to 4.38)	2.3	1.6	82 (81)	116.95(102.36 to 131.47)	74.5	42.5	51 (46)
Marshall Islands	0.5	5.77(4.09 to 8.12)	2.3	3.4	177 (178)	210.32(144.75 to 299.86)	76.3	134.1	182 (183)
Mauritania	0.5	2.89(2.05 to 3.82)	2.3	0.6	23 (22)	96.98(63.45 to 138.51)	76.2	20.8	21 (15)
Mauritius	0.7	3.39(2.79 to 4.07)	2.3	1.1	50 (49)	141.89(116.02 to 171.18)	76.3	65.6	100 (95)
Mexico	0.6	4.37(3.84 to 4.95)	2.3	2.1	108 (109)	194.13(170.74 to 217.87)	76.2	117.9	174 (174)
Micronesia (Federated States of)	0.6	5.78(3.65 to 8.28)	2.3	3.5	178 (179)	204.27(109.23 to 298.4)	76.2	128.1	180 (180)
Monaco	0.9	9.66(7.42 to 11.85)	2.3	7.4	203 (203)	273.49(215.35 to 341.21)	73.4	200.1	201 (201)
Mongolia	0.6	2.88(2.2 to 3.74)	2.3	0.6	22 (21)	114(85.4 to 151.89)	76.2	37.8	46 (44)
Montenegro	0.8	4.58(3.77 to 5.52)	2.3	2.3	130 (129)	144.73(115.67 to 177.2)	73.5	71.2	111 (100)
Morocco	0.5	2.47(1.91 to 3.1)	2.3	0.2	4 (4)	77.29(57.99 to 98.82)	76.2	1.1	3 (3)
Mozambique	0.3	4.32(3.22 to 5.69)	2.4	1.9	99 (106)	180.87(118.93 to 283.85)	89.4	91.5	140 (162)
Myanmar	0.5	5.53(4.19 to 7.15)	2.3	3.2	172 (172)	225.56(157.1 to 311.15)	76.2	149.4	191 (190)
Namibia	0.6	2.53(1.95 to 3.23)	2.3	0.2	8 (6)	80.87(57.28 to 107.56)	76.2	4.7	4 (4)
Nauru	0.6	6.46(4.62 to 8.8)	2.3	4.1	189 (189)	242.73(166.99 to 346.34)	76.2	166.5	196 (196)
Nepal	0.4	3.08(2.36 to 3.88)	2.3	0.8	31 (30)	98.37(76.25 to 124.12)	84.5	13.9	10 (19)
Netherlands	0.9	5.43(4.87 to 5.9)	2.3	3.1	167 (167)	134(123.11 to 144.95)	73.7	60.3	83 (76)
New Zealand	0.8	5.05(4.63 to 5.4)	2.3	2.8	151 (151)	137.31(129.6 to 144.69)	74.3	63.0	92 (84)
Nicaragua	0.5	4.41(3.53 to 5.33)	2.3	2.1	112 (114)	174.41(129.13 to 220.56)	76.2	98.2	150 (151)
Niger	0.2	3.32(2.31 to 4.5)	2.7	0.6	25 (42)	129.05(85.74 to 188.43)	102.9	26.2	31 (64)
Nigeria	0.5	2.87(2.24 to 3.58)	2.3	0.5	20 (19)	96.97(72.25 to 128.36)	76.2	20.8	20 (14)
Niue	0.7	4.4(3.17 to 5.8)	2.3	2.1	110 (112)	162.66(111.96 to 221.55)	76.1	86.6	137 (137)
North Macedonia	0.7	4.57(3.55 to 5.73)	2.3	2.3	126 (126)	154.1(111.29 to 197.31)	73.8	80.3	130 (127)
Northern Mariana Islands	0.8	4.39(3.65 to 5.38)	2.3	2.1	114 (110)	146.35(118.6 to 187.16)	73.4	72.9	116 (106)
Norway	0.9	3.07(2.84 to 3.25)	2.3	0.8	34 (29)	84.02(79.07 to 89.3)	73.5	10.6	7 (5)
Oman	0.8	4.45(3.65 to 5.19)	2.3	2.2	119 (117)	128.84(101.38 to 153.55)	73.8	55.0	71 (63)
Pakistan	0.4	3.94(3.28 to 4.72)	2.3	1.6	83 (83)	164.17(134.44 to 198.88)	83.7	80.4	131 (139)
Palau	0.7	2.28(1.72 to 2.85)	2.3	0.0	1 (1)	73.42(56.55 to 91.37)	73.4	0.0	1 (1)
Palestine	0.6	7.27(6.07 to 8.56)	2.3	4.9	199 (199)	203.13(170.77 to 240.13)	76.3	126.8	178 (178)
Panama	0.7	4.46(3.47 to 5.59)	2.3	2.1	117 (119)	181.64(139.65 to 229.35)	76.2	105.5	165 (165)
Papua New Guinea	0.4	4.5(3.15 to 6.45)	2.3	2.2	121 (122)	185.1(125.31 to 275.56)	83.9	101.2	158 (167)
Paraguay	0.6	4.52(3.43 to 5.84)	2.3	2.2	123 (123)	161.69(122.22 to 210.7)	76.2	85.5	136 (135)
Peru	0.6	4.4(3.03 to 6)	2.3	2.1	111 (113)	180.89(119.2 to 253.41)	76.2	104.7	164 (163)
Philippines	0.6	4.55(3.81 to 5.42)	2.3	2.2	125 (125)	191.18(161.76 to 223.59)	76.2	115.0	172 (172)
Poland	0.8	5.21(4.43 to 6.07)	2.3	2.9	162 (162)	145.41(124.73 to 167.9)	73.8	71.7	113 (103)
Portugal	0.7	4.65(4.19 to 5.03)	2.3	2.4	134 (133)	133.39(121.96 to 145.39)	74.4	59.0	79 (75)
Puerto Rico	0.8	4.06(3.22 to 5.08)	2.3	1.8	89 (88)	124.19(97.87 to 156.45)	73.4	50.8	64 (56)
Qatar	0.8	8.54(5.58 to 12.24)	2.3	6.3	202 (202)	172.5(126.62 to 229.3)	74.1	98.4	151 (148)
Republic of Korea	0.9	2.69(2 to 3.15)	2.3	0.4	14 (12)	96.63(72.15 to 116.25)	73.8	22.8	24 (13)
Republic of Moldova	0.7	2.9(2.58 to 3.25)	2.3	0.6	24 (23)	118.39(104.99 to 132.69)	76.2	42.2	50 (48)
Romania	0.8	4.16(3.43 to 4.97)	2.3	1.9	98 (96)	142.37(118.98 to 169.26)	73.8	68.6	107 (97)
Russian Federation	0.8	3.55(3.11 to 4.03)	2.3	1.3	60 (56)	122.63(107.65 to 138.69)	73.8	48.9	62 (54)
Rwanda	0.4	3.25(2.42 to 4.34)	2.3	0.9	41 (38)	133.26(90.54 to 199.49)	84.4	48.9	61 (74)
Saint Kitts and Nevis	0.7	4.06(3.22 to 4.92)	2.3	1.8	91 (89)	136.94(99.49 to 175.74)	74.5	62.5	88 (83)
Saint Lucia	0.7	4.15(3.48 to 4.85)	2.3	1.8	94 (95)	153.87(127.26 to 182.31)	76.2	77.7	126 (126)
Saint Vincent and the Grenadines	0.6	4.72(4.11 to 5.43)	2.3	2.4	136 (136)	173.08(147.1 to 203.75)	76.6	96.5	149 (150)
Samoa	0.6	4.63(3.42 to 5.97)	2.3	2.3	131 (131)	152.23(106.93 to 203.33)	76.2	76.1	123 (123)
San Marino	0.9	8.22(5.48 to 11.55)	2.3	5.9	201 (201)	230.29(156.84 to 327.24)	73.5	156.8	194 (193)
Sao Tome and Principe	0.5	2.82(2.08 to 3.67)	2.3	0.5	17 (16)	100.53(71.87 to 136.25)	76.3	24.2	29 (24)
Saudi Arabia	0.8	3.43(2.67 to 4.59)	2.3	1.1	52 (50)	107.27(82.58 to 141.53)	73.4	33.8	40 (35)
Senegal	0.4	3.54(2.58 to 4.55)	2.3	1.2	57 (54)	127.35(88.49 to 171.31)	84.5	42.8	52 (60)
Serbia	0.8	4.93(3.81 to 6.16)	2.3	2.6	147 (147)	142.07(106.73 to 177.95)	74.3	67.8	106 (96)
Seychelles	0.7	6.36(5.48 to 7.36)	2.3	4.0	185 (185)	219.06(186.64 to 260.91)	74.9	144.1	189 (187)
Sierra Leone	0.3	3.6(2.55 to 4.96)	2.4	1.2	54 (60)	149.82(98.35 to 220.85)	87.1	62.7	91 (117)

Singapore	0.9	2.75(2.49 to 3.01)	2.3	0.5	15 (13)	93.82(85.02 to 103.7)	73.7	20.1	19 (8)
Slovakia	0.8	4.46(3.29 to 5.68)	2.3	2.2	122 (120)	135.1(101.55 to 173.11)	73.8	61.4	86 (79)
Slovenia	0.8	4.58(3.59 to 5.85)	2.3	2.3	129 (128)	121.97(96.76 to 154.07)	73.5	48.5	60 (53)
Solomon Islands	0.4	6.83(4.66 to 10.67)	2.3	4.5	196 (196)	266.25(170.17 to 435.5)	84.6	181.7	200 (200)
Somalia	0.1	3.49(2.24 to 4.9)	3.3	0.2	10 (52)	149.58(96.56 to 212.99)	138.7	10.9	8 (116)
South Africa	0.7	3.34(2.66 to 3.76)	2.3	1.0	47 (46)	99.42(83.2 to 113.33)	76.4	23.0	25 (20)
South Sudan	0.4	3.34(2.38 to 4.56)	2.4	0.9	42 (47)	148.89(104.37 to 203.18)	83.9	65.0	96 (114)
Spain	0.8	4.42(4.01 to 4.78)	2.3	2.1	118 (115)	127.21(117.29 to 137.62)	73.7	53.6	69 (59)
Sri Lanka	0.7	3.84(2.79 to 4.99)	2.3	1.5	80 (80)	140.23(99.67 to 185.72)	76.1	64.1	94 (90)
Sudan	0.5	6.44(4.4 to 8.69)	2.3	4.1	188 (188)	234.93(155.79 to 329.67)	76.2	158.8	195 (195)
Suriname	0.6	3.65(2.95 to 4.43)	2.3	1.3	64 (66)	143.5(114.88 to 175.81)	76.2	67.3	104 (99)
Sweden	0.9	4.32(3.98 to 4.58)	2.3	2.0	107 (105)	111.92(105.3 to 118.13)	74.1	37.8	47 (39)
Switzerland	0.9	3.69(3.34 to 4.01)	2.3	1.4	74 (72)	100.94(92.72 to 110.84)	73.4	27.5	33 (26)
Syrian Arab Republic	0.6	15.82(11.96 to 20.41)	2.3	13.5	205 (205)	456.25(348.86 to 606.33)	76.3	380.0	205 (205)
Taiwan (Province of China)	0.9	3.76(2.99 to 4.79)	2.3	1.5	76 (74)	129.5(103.22 to 164.03)	74.1	55.4	72 (65)
Tajikistan	0.5	3.82(3.09 to 4.91)	2.3	1.5	79 (79)	154.51(118.3 to 217.7)	76.2	78.4	127 (129)
Thailand	0.7	4.69(2.95 to 6.35)	2.3	2.4	133 (135)	171.17(119.67 to 224.21)	76.2	95.0	147 (146)
Timor-Leste	0.5	5.04(3.64 to 6.42)	2.3	2.7	149 (150)	186.29(117.62 to 239.52)	76.2	110.1	169 (169)
Togo	0.4	3.23(2.4 to 4.22)	2.3	0.9	38 (37)	116.63(85.14 to 154.06)	84.5	32.1	39 (45)
Tokelau	0.6	4.09(2.88 to 5.62)	2.3	1.8	90 (91)	141.83(95.97 to 201.22)	76.2	65.7	101 (94)
Tonga	0.6	3.09(2.39 to 3.93)	2.3	0.8	32 (31)	106.72(79.93 to 140.62)	76.2	30.5	37 (34)
Trinidad and Tobago	0.8	3.61(2.75 to 4.65)	2.3	1.3	68 (62)	150.84(113.33 to 194.85)	73.7	77.2	124 (122)
Tunisia	0.7	2.93(2.14 to 3.92)	2.3	0.6	28 (26)	95.35(69.88 to 127.03)	76.2	19.2	18 (12)
Turkey	0.7	5.28(4.21 to 6.68)	2.3	3.0	165 (165)	176.71(143.76 to 214.02)	74.3	102.4	160 (156)
Turkmenistan	0.7	3.22(2.63 to 3.97)	2.3	0.9	39 (36)	147.98(122.05 to 180.79)	76.2	71.8	114 (112)
Tuvalu	0.6	4.91(3.37 to 6.87)	2.3	2.6	144 (145)	175.06(117.54 to 248.92)	76.2	98.9	152 (153)
Uganda	0.4	2.4(1.77 to 3.17)	2.3	0.1	3 (3)	98.31(66.58 to 140.9)	84.0	14.3	12 (18)
Ukraine	0.7	4.78(4.07 to 5.69)	2.3	2.5	140 (138)	194.66(166.43 to 227.72)	74.1	120.5	175 (175)
United Arab Emirates	0.9	6.56(4.33 to 8.71)	2.3	4.3	193 (192)	177.7(130 to 229.86)	73.4	104.3	163 (159)
United Kingdom	0.8	4.67(4.33 to 4.87)	2.3	2.4	135 (134)	119.88(114.88 to 124.72)	73.9	46.0	57 (50)
United Republic of Tanzania	0.4	3.65(2.73 to 4.85)	2.3	1.3	67 (67)	161.63(112.08 to 236.39)	84.3	77.4	125 (134)
United States of America	0.9	5.74(5.36 to 6.01)	2.3	3.5	179 (176)	145.44(139.75 to 150.82)	73.5	72.0	115 (104)
United States Virgin Islands	0.8	4.37(3.62 to 5.19)	2.3	2.1	113 (108)	139.32(112.21 to 172.56)	73.8	65.5	98 (87)
Uruguay	0.7	5.51(5.06 to 6)	2.3	3.2	170 (171)	169.89(156.11 to 182.93)	76.3	93.6	142 (143)
Uzbekistan	0.6	4.24(3.66 to 4.93)	2.3	1.9	101 (100)	175.86(150.42 to 206.78)	76.3	99.5	155 (155)
Vanuatu	0.5	5.1(3.4 to 7.23)	2.3	2.8	152 (154)	185.97(121.8 to 265.95)	76.6	109.4	168 (168)
Venezuela (Bolivarian Republic of)	0.6	4.13(3.21 to 5.32)	2.3	1.8	93 (94)	170.84(131.89 to 219.71)	76.2	94.7	144 (145)
Viet Nam	0.6	3.81(2.95 to 4.83)	2.3	1.5	77 (78)	128.66(98.87 to 163.62)	76.2	52.5	66 (62)
Yemen	0.4	6.57(4.47 to 9.14)	2.3	4.2	192 (193)	226.14(148.08 to 326.31)	84.7	141.4	188 (191)
Zambia	0.5	3.63(2.82 to 4.56)	2.3	1.3	62 (65)	147.43(106.74 to 205.82)	76.3	71.2	110 (110)
Zimbabwe	0.5	2.88(2.19 to 3.89)	2.3	0.6	21 (20)	100.62(77.14 to 131.14)	77.2	23.4	26 (25)

Note: ASR age-standardized rate, ASDR age-standardized death rate, DALYs disability-adjusted life years, SDI socio-demographic index.
